# Prognosis of Non-small-cell Lung Cancer Patients With Lipid Metabolism Pathway Alternations to Immunotherapy

**DOI:** 10.3389/fgene.2021.646362

**Published:** 2021-07-14

**Authors:** Tianli Cheng, Jing Zhang, Danni Liu, Guorong Lai, Xiaoping Wen

**Affiliations:** ^1^Thoracic Medicine Department I, Hunan Cancer Hospital, Changsha, China; ^2^Thoracic Medicine Department I, Affiliated Tumor Hospital of Xiangya Medical School of Central South University, Changsha, China; ^3^HaploX Biotechnology, Shenzhen, China

**Keywords:** immune checkpoint inhibitors, non-small-cell lung cancer, predictive marker, lipid metabolism pathway, immune microenvironment

## Abstract

Immune checkpoint inhibitors (ICIs) significantly improve the survival of patients with non-small-cell lung cancer (NSCLC), but only some patients obtain clinical benefits. Predictive biomarkers for ICIs can accurately identify people who will benefit from immunotherapy. Lipid metabolism signaling plays a key role in the tumor microenvironment (TME) and immunotherapy. Hence, we aimed to explore the association between the mutation status of the lipid metabolism pathway and the prognosis of patients with NSCLC treated with ICIs. We downloaded the mutation data and clinical data of a cohort of patients with NSCLC who received ICIs. Univariate and multivariate Cox regression models were used to analyze the association between the mutation status of the lipid metabolism signaling and the prognosis of NSCLC receiving ICIs. Additionally, The Cancer Genome Atlas (TCGA)–NSCLC cohort was used to explore the relationships between the different mutation statuses of lipid metabolism pathways and the TME. Additionally, we found that patients with high numbers of mutations in the lipid metabolism pathway had significantly enriched macrophages (M0- and M1-type), CD4 + T cells (activated memory), CD8 + T cells, Tfh cells and gamma delta T cells, significantly increased expression of inflammatory genes [interferon-γ (IFNG), CD8A, GZMA, GZMB, CXCL9, and CXCL10] and enhanced immunogenic factors [neoantigen loads (NALs), tumor mutation burden (TMB), and DNA damage repair pathways]. In the local-NSCLC cohort, we found that the group with a high number of mutations had a significantly higher tumor mutation burden (TMB) and PD-L1 expression. High mutation status in the lipid metabolism pathway is associated with significantly prolonged progression-free survival (PFS) in NSCLC, indicating that this marker can be used as a predictive indicator for patients with NSCLC receiving ICIs.

## Introduction

Lung cancer is a malignant tumor with the highest morbidity and mortality worldwide (Bray et al., 2018). Non-small-cell lung cancer (NSCLC) is the most common pathological type of lung cancer, and the 5-year survival rate is less than 15% (Herbst et al., 2018; Siegel et al., 2018). Immune checkpoint inhibitors (ICIs) have an antitumor effect by restoring T cell-mediated antitumor immune function and have become a novel clinical treatment tool for NSCLC; however, growing evidence suggests that not all NSCLC patients benefit from ICIs. In the unscreened NSCLC populations, the objective response rate (ORR) to ICIs is commonly less than 20% (Garon et al., 2015). Thus, predicting the effectiveness of ICIs, identifying patients who can benefit from ICIs (Gibney et al., 2016), and maximizing the efficacy of immunotherapy are of great significance for the precise treatment of NSCLC.

PD-L1 expression and tumor mutation burden (TMB) are commonly used markers of immune efficacy. Additionally, high microsatellite instability (MSI-H), deficient mismatch repair (dMMR), tumor-infiltrating lymphocytes (TILs), and the intestinal microbial flora have also show certain predictive value. Although the research conclusions are constantly evolving, some limitations remain (Herbst et al., 2016; Langer et al., 2016; Brody et al., 2017; Chen et al., 2019). For example, a small number of NSCLC patients with low PD-L1 expression seem to be “biomarker negative” but still respond to ICI-based treatment. In contrast, not all patients with high PD-L1 expression can obtain clinical benefit from ICIs (Langer et al., 2016). Additionally, there are many challenges for detecting TMB in clinical practice, including determining the ideal approach for detecting TMB, determining the appropriate cutoff for high or low TMB, and reaching a consensus regarding the different numbers of genes detected by different platforms (Chowell et al., 2018). Moreover, the incidence of MSI-H in NSCLC is very low, so the values of MSI-H and dMMR for predicting the efficacy of ICIs in NSCLC remain to be verified (Warth et al., 2016; Vanderwalde et al., 2018). Hence, how to identify which patients with NSCLC should be treated with ICIs has become an urgent problem in clinical practice.

Metabolic reprogramming processes, such as lipid metabolism, play an important role in the tumor microenvironment (TME) and immunotherapy (DeBerardinis et al., 2008; Yoshida, 2015; Sun, 2016; Baek et al., 2017; Ma et al., 2019). Tumor cells produce large amounts of fatty acids through *de novo* synthesis, and a fatty acid-enriched TME affects the function of effector T cells and M1-type macrophages and is conducive to the production of Tregs and M2 macrophages (Gaber et al., 2017), causing an immunosuppressive TME. Jiang et al. (2018) found that the overexpression of fatty acid synthase (FASN) in ovarian cancer contributed to lipid accumulation in tumor-infiltrating dendritic cells (DCs), causing T cell dysfunction, which in turn induced an impaired antitumor immune response and thus inhibited the ability of fatty acid synthesis to enhance antitumor immunity. Lin et al. (Lin R. et al., 2020) found that tissue-resident memory T (Trm) cells in gastric adenocarcinoma do not use glucose but rather rely on fatty acid oxidation for energy. Cancer cells and Trm cells compete for lipid metabolism, leading to Trm cell death. Blocking PD-L1 can regulate Trm cell metabolism, promote lipid uptake, and further enhance antitumor immune ability. Moreover, several studies have suggested that alterations in specific signaling pathways are associated with the prognosis of patients receiving ICIs and can be used as novel markers to identify patients who will gain benefit from immunotherapy (Teo et al., 2018; Wang et al., 2018). Hence, based on the above results, we aimed to illustrate the association between the mutation status of the lipid metabolism pathway and the prognosis of NSCLC patients treated with ICIs to identify a means to further predict which population of patients with NSCLC will respond to ICIs.

## Materials and Methods

### Immunotherapy Cohort, The Cancer Genome Atlas Cohort, and Local Cohort

One cohort of NSCLC patients who received ICIs [anti-PD-(L)1 monotherapy or anti-PD-(L)1 in combination with anti-CTLA-4 therapy] was derived from a published study reported by Rizvi et al. (2018). This immunotherapy cohort included a total of 240 NSCLC patients with clinical data and mutation data. Additionally, we used the TCGAbiolinks R package to download mutation data, expression data and clinical data from the LUAD and LUSC cohorts in The Cancer Genome Atlas (TCGA) database (Colaprico et al., 2016). The TCGA-LUAD and TCGA-LUSC cohorts were combined into one cohort in the subsequent analysis, called the TCGA cohort (Tomczak et al., 2015). We collected 115 formalin-fixed paraffin-embedded (FFPE) NSCLC samples from the Thoracic Medicine Department I, Hunan Cancer Hospital and Thoracic Medicine Department I, Affiliated Tumor Hospital of Xiangya Medical School of Central South University and performed panel sequencing. The human NSCLC tumor specimens, panel sequencing, data processing, and pathological diagnosis are detailed in the Supplementary Methods.

### Mutation Data Preprocessing

To explore the association between the mutation status of the lipid metabolism pathway and the prognosis of NSCLC patients receiving ICIs, we downloaded the lipid metabolism gene set from MSigDB (Liberzon et al., 2011). First, we filtered the mutation data and retained only the non-synonymous mutation data. Next, we counted the non-synonymous mutations in the lipid metabolism pathway in each sample. According to the median number of non-synonymous mutations that occurred in this pathway in each dataset, each sample was divided into a group with a high number of mutations and a group with a low number of mutations in lipid metabolism molecules (Supplementary Table 1). In the subsequent analysis, we will refer to these two groups as the high mutation group and the low mutation group for short. Additionally, in the mutation frequency analysis, we only compared the top 20 mutations in each cohort.

### Immune Microenvironment Analysis

We used the CIBERSORT algorithm and LM22.txt to estimate the proportions of 22 types of TILs from the expression data of NSCLC patients (Newman et al., 2015). Additionally, immune-related genes, immune checkpoint genes and immune-related scores were obtained from published studies (Rooney et al., 2015; Thorsson et al., 2018). The gene set enrichment analysis (GSEA) algorithm was used to determine the pathways that were significantly enriched or downregulated in the high mutation and low mutation groups (Subramanian et al., 2007). We analyzed and compared the gene ontology (GO) terms, Kyoto Encyclopedia of Genes and Genomes (KEGG) pathways and Reactome pathways enriched in the high and low mutation groups. The enrichment score (ES) and *P*-value were used to evaluate the activity of the pathway and whether there was a significant difference.

### Statistical Analysis

The Mann–Whitney *U* test and Fisher’s exact test were applied to the comparison of the difference between the continuous and categorical variables between high-mut and low-mut groups, respectively. We used the Kaplan–Meier (KM) curve, univariate and multivariate Cox model, and the log-rank test to evaluate the effect of the mutation status of lipid metabolism on the prognosis of NSCLC receiving ICIs. Also, the “ggpubr” R package was used to visualize boxplots (Kassambara, 2018). *P* less than 0.05 was regarded as statistically significant.

## Results

### A Higher Number of Mutations in the Lipid Metabolism Pathway Was Associated With Favorable Prognosis in Patients Treated With ICIs

**FIGURE 1:**
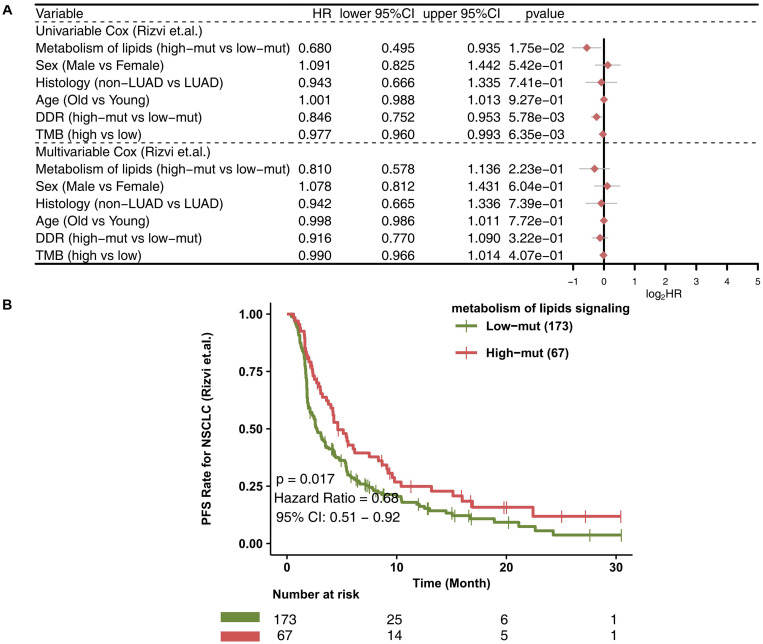
The value of clinical characteristics and the number of mutations in the lipid metabolism pathway for predicting ICI efficacy. **(A)** Forest plot displaying the results of the univariate and multivariate Cox regression analyses in the ICI-treated cohort (Rizvi et al., 2018). The main portion of the forest plot presents the hazard ratio (HR) and 95% confidence interval (95% CI), and red dots indicate *P* < 0.05. Predictors of favorable outcomes have an HR < 1, and predictors of poor outcome have an HR > 1. **(B)** KM survival curves for PFS in 240 NSCLC patients from the ICI-treated cohort (Rizvi et al., 2018).

In the ICI-treated cohort, we used a univariate-Cox model to analyze the effects of a high number of mutations in the lipid metabolism pathway, sex, histological type, and age at the time of prognosis of NSCLC patients receiving immunotherapy (Figure 1A). We found that a high number of mutations in the lipid metabolism pathway, a high TMB and a high number of alterations in DNA damage repair (DDR) signaling were associated with prolonged progression-free survival (PFS) in the ICI-treated cohort; however, the results of the multivariate Cox analysis showed that a high number of mutations in the lipid metabolism pathway, a high TMB, or a high number of mutations in DDR signaling could not be used as an independent predictor of the prognosis of patients with NSCLC receiving ICIs. Similarly, NSCLC patients with a high number of mutations in the lipid metabolism pathway had significantly prolonged PFS than those with a low number of mutations [*P* = 0.017; HR = 0.68; 95% confidence interval (95% CI): 0.51–0.92; Figure 1B]. Moreover, we found that the PFS time of the high number of mutations in the lipid metabolism pathway combined with the high TMB group was significantly prolonged than that of the low number of mutations in the lipid metabolism pathway combined with the low TMB group (Supplementary Figure 1; *P* = 0.008; HR = 0.548). Also, we found that the high-TMB group had significantly prolonged PFS time compared with the low-TMB group (Supplementary Figure 2; *P* = 0.024; HR = 0.73).

### Comparison of Mutated Genes Between the High and low Mutation Groups

**FIGURE 2:**
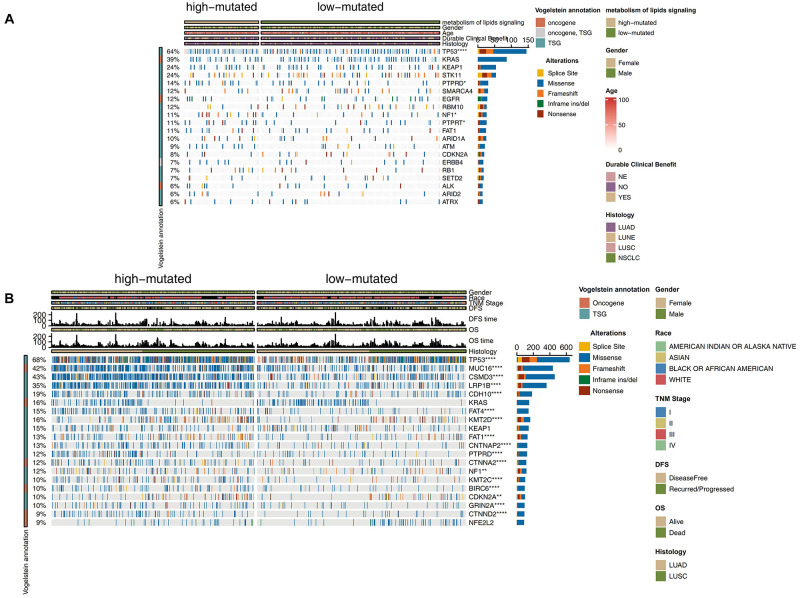
Genomic profiles of NSCLC patients in the ICI-treated cohort (Rizvi et al., 2018) **(A)** and TCGA-NSCLC **(B)** cohorts. The top 20 genes with the highest mutation frequencies and the corresponding clinical information are shown. The top five genes with the highest mutation frequencies in the ICI-treated cohort (Rizvi et al., 2018) were TP53, KRAS, KEAP1, STK11, and PTPRD. The top five genes with the highest mutation frequencies in the TCGA cohort were TP53, TTN, MUC16, CSMD3, and RYR2. The mutation types are indicated as follows: yellow indicates splice site mutations, blue indicates missense mutations, orange indicates frameshift mutations, green indicates in-frame insertions/deletions, and brown indicates nonsense mutations. The clinical characteristics are shown as patient annotations.

To compare the differences in known cancer driver genes between the high and low mutation groups, we visualized the top 20 mutated driver genes in each group and used Fisher’s exact test to calculate the statistical differences. In the ICI-treated cohort, the high mutation group had more gene mutations than the low mutation group. Compared with the low mutation group, the high mutation group had significantly increased TP53 mutations (79.1% vs. 50.9%; *P* < 0.05), PTPRD mutations (20.9% vs. 9.2%; *P* < 0.05), NF1 mutations (17.9% vs. 7.5%; *P* < 0.05), and PTPRT mutations (17.9% vs. 7.5%; *P* < 0.05; Figure 2A). Among the above-mentioned genetic mutations with significant differences, most of the mutations were missense mutations, followed by frameshift mutations. In the TCGA cohort, the high mutation group had a higher frequency of driver genes than the low mutation group (*P* < 0.05; Figure 2B), while three genes (KRAS, KEAP1, and NFE2L2) showed no significant difference between high and low mutation groups. The results of the mutual exclusivity analysis of the lipid metabolism genes in the high mutation group compared to the low mutation group showed no significant difference (Supplementary Figure 3). We also compared lipid metabolism mutation frequency differences between the high mutation group and the low mutation group (Supplementary Figure 4). Compared with the low mutation group, the high mutation group had significantly increased mutations in PIK3CG (15.0% vs. 5.20%), PIK3CA (15.0% vs. 2.31%), PIK3C2G (13.4% vs. 1.73%), PIK3C3 (11.9% vs. 1.16%), INPP4B (10.4% vs. 1.16%), NCOR1 (10.4% vs. 1.16%), EP300 (10.4% vs. 1.58%), PTEN (8.96% vs. 1.16%), INPP4A (7.46% vs. 0%), and PIK3R2 (5.97% vs. 0.98%).

### Comparison of the Immune Microenvironment Between the High- and Low-Mutation Groups

**FIGURE 3:**
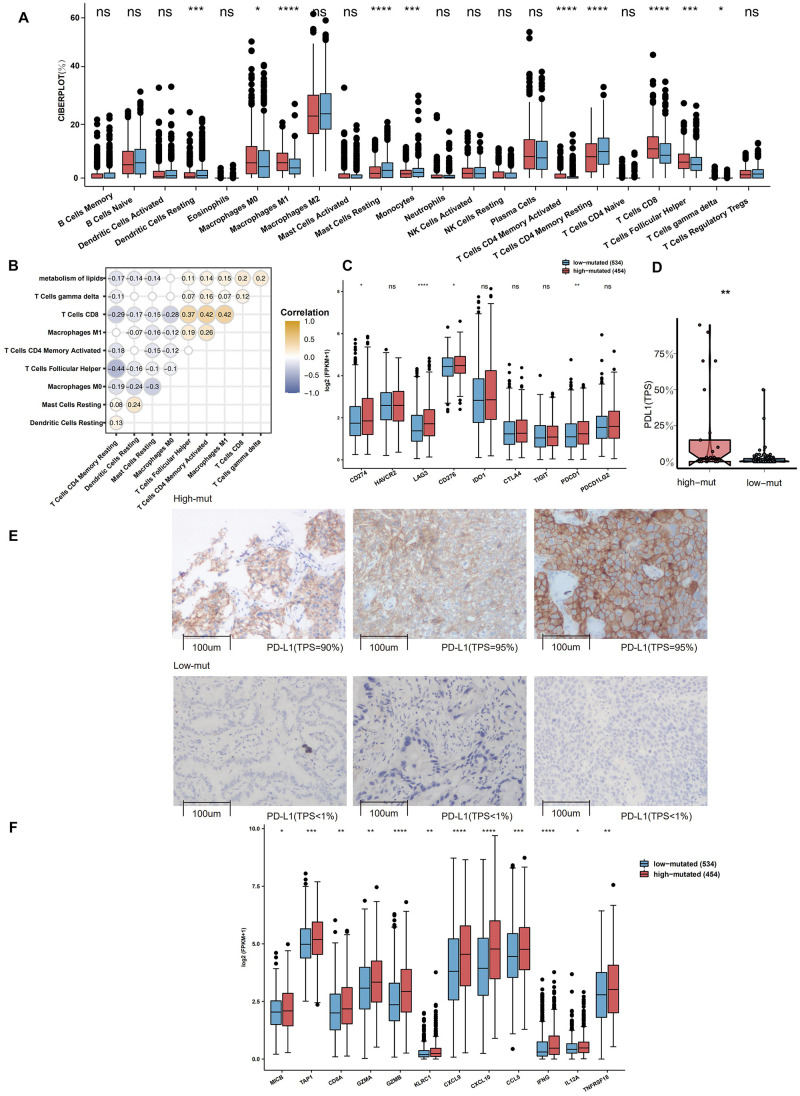
**(A)** Comparison of the fractions of 22 types of immune cells as estimated by the CIBERSORT algorithm between the high and low mutation groups in the TCGA cohort. **(B)** The correlations between the number of mutations in the lipid metabolism pathway and the proportions of immune cells. **(C)** Comparison of the expression of immune-related genes between the high and low mutation groups in the TCGA cohort. **(D)** Comparison of the expression of PD-L1 (TPS) between the high and low mutation groups in the Local-NSCLC cohort. **(E)** The typical cases for each TPS level between the high (three samples; high PD-L1 TPS) and low mutation (three samples; no PD-L1 TPS) groups in the Local-NSCLC. Using HE and PD-L1 stained slides, we manually assessed the number of tumor cells, the sample size (diameter), the crush rate with a cut-off value of <1% (no PD-L1 TPS), 1–50% (low PD-L1 TPS), 50% < (high PD-L1 TPS), and the TPS for each biopsy sample using the slide that contained the most tumor cells. The TPS level was evaluated by pathologists who completed training courses in TPS estimation. **(F)** Heatmap depicting the mean differences in the expression of proinflammatory and antigen presentation genes between the high and low mutation groups in the TCGA cohort. Each square represents the fold change or the mean difference in the expression of these genes between the high and low mutation groups in the TCGA cohort. Red indicates upregulation.

To explore differences in the TME between the high-mutation group and the low-mutation group, the CIBERSORT algorithm was applied to evaluate the proportions of twenty-two different immune cells in the TME. Compared with the low-mutation group, the high-mutation group had significantly enriched macrophages (M0- and M1-type), CD4 + T cells (activated memory), CD8 + T cells, Tfh cells, and gamma delta T cells (all *P* < 0.05; Figure 3A). Additionally, as shown in Figure 3B, the number of mutations in the lipid metabolism pathway had a significantly positive correlation with the proportion of macrophages (M1-type), CD4 + T cells (activated memory), CD8 + T cells, Tfh cells, and gamma delta T cells (*R* > 0, *P* < 0.05). A high proportion of CD8 + T cells was significantly correlated with a high proportion of Tfh cells, macrophages (M1-type) and CD4 + T cells (activated memory) (*R* > 0, *P* < 0.05; Figure 3B). In contrast, some activated immune cells had a significantly negative correlation with the ratio of resting/suppressive immune cells (*R* < 0, *P* < 0.05; Figure 3B). Moreover, we found that the high-mutation group had higher expression levels of immune checkpoint molecules (Figure 3C), such as CD274 (PD-L1), LAG3, CD276, and PDCD1 (PD-1), than the low-mutation group. In the local-NSCLC cohort, patients with a high number of mutations in the lipid metabolism pathway had high levels of PD-L1 (*P* < 0.05; Figure 3D). Figure 3E shows typical cases for each TPS level (lipid metabolism: 3 high-mutation vs. 3 low-mutation cases). Similarly, the expression of inflammatory genes, such as cytotoxicity markers (CD8A, GZMA, and GZMB), antigen processing and presentation markers (MICB and TAP1), and inflammatory cytokines (CXCL9, CXCL10, CCL5, IFNG, IL12A, and TNFRSF18), was significantly higher in the high-mutation group than in the low-mutation group (all *P* < 0.05, Figure 3F).

### Comparison of Immunogenicity Between the High and Low Mutation Groups

**FIGURE 4:**
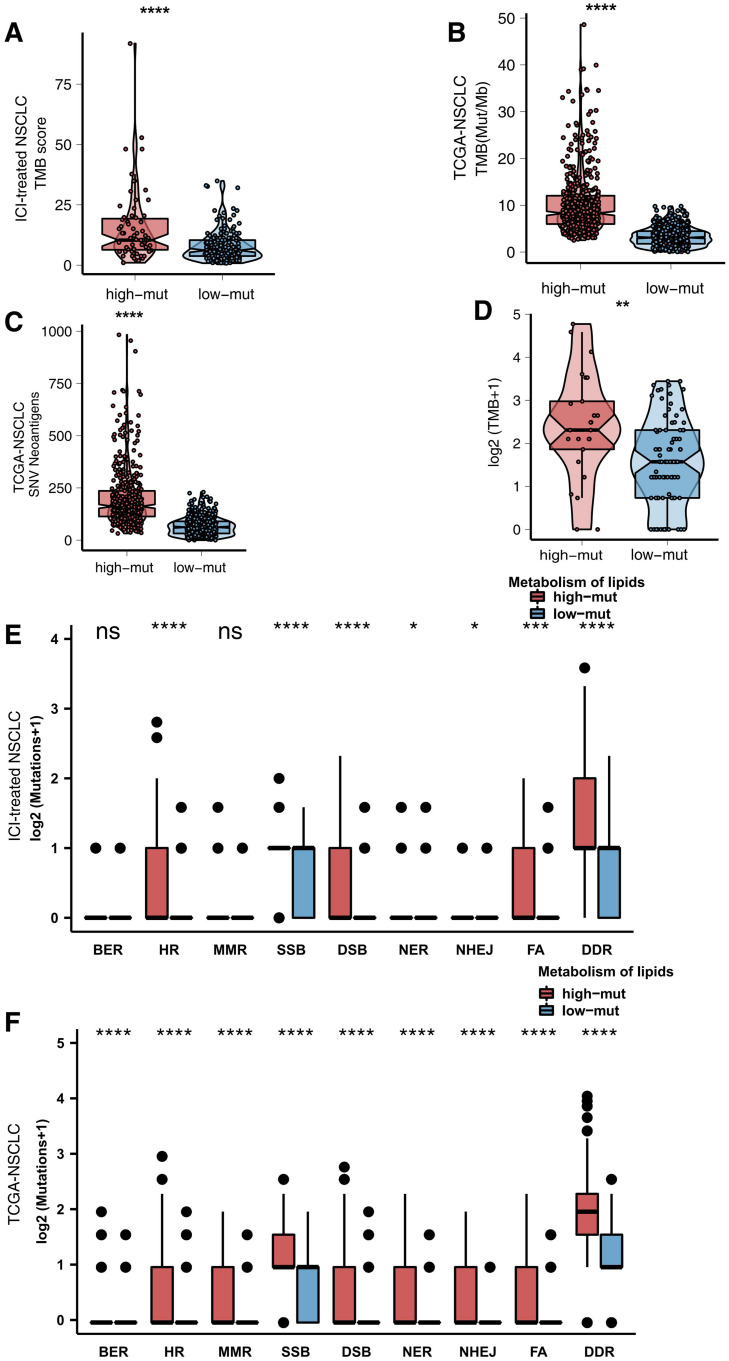
**(A)** Comparison of TMB scores between the high and low mutation groups in the ICI-treated cohort (Rizvi et al., 2018). **(B)** Comparison of TMB between the high and low mutation groups in the TCGA cohort. **(C)** Comparison of TMB between the high and low mutation groups in the Local-NSCLC cohort. **(D)** Comparison of NAL between the high and low mutation groups in the TCGA cohort. **(E)** Comparison of DNA damage-related gene set alterations between the high and low mutation groups in the ICI-treated cohort (Rizvi et al., 2018). **(F)** Comparison of DNA damage-related gene set alterations between the high and low mutation groups in the TCGA cohort.

Immunogenicity is a vital factor affecting the prognosis of patients with NSCLC receiving ICIs and the efficacy of ICIs. We determined the differences in immunogenicity between the high and low mutation groups. For TMB, in both the ICI-treated cohort and the TCGA cohort, compared with the low mutation group, the high mutation group had a significantly enhanced TMB (all *P* < 0.05; Figures 4A,B). In the Local-NSCLC cohort, we found that patients with a high number of mutations in the lipid metabolism pathway had high levels of TMB (*P* < 0.05; Figure 4C). Additionally, the high mutation group had a higher neoantigen load (NAL) than the low mutation group (*P* < 0.05; Figure 4D). DDR signaling pathways play a key role in correcting DNA damage. We downloaded eight DDR signaling pathway gene sets from MSigDB and merged these gene sets into one (the merged DDR pathway gene set). In the ICI-treated cohort, in most DDR pathways such as homologous recombination (HR), single-strand break (SSB), double-strand break (DSB), nucleotide excision repair (NER), non-homologous end joining (NHEJ), Fanconi anemia (FA), and merged DDR pathways, the high mutation group had a significantly increased number of mutations (*P* < 0.05; Figure 4E). In the TCGA cohort, the high mutation group had a higher number of non-synonymous mutations in all DDR pathways than the low mutation group (all *P* < 0.05; Figure 4F).

### Differences in Pathway Activity Between the High and Low Mutation Groups

**FIGURE 5:**
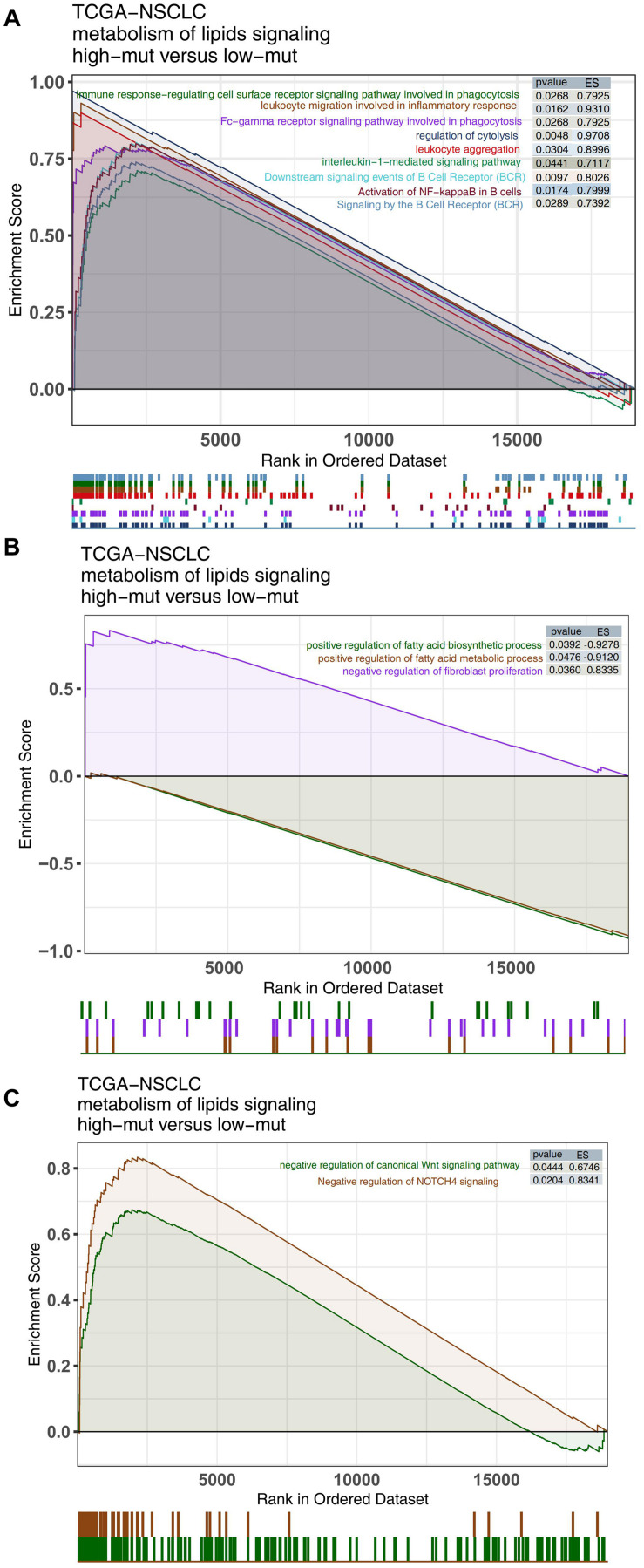
Comparison of GSEA results between the high and low mutation groups in the TCGA cohort. GSEA-identified differences in immune cell **(A)**, exhaustion-related **(B)**, and oncogenic pathway activities **(C)** between the high and low mutation groups in the TCGA cohort.

Alterations in functional pathway activity also have impacts on the efficacy of ICIs and the prognosis of NSCLC patients receiving ICIs. We used the ClusterProfiler R package to perform GSEA with the NSCLC expression data from the high and low mutation groups. Immune-related pathway terms, such as lymphocyte recruitment and participation in the inflammatory response, lymphocyte aggregation, interleukin 1, and BCR pathway activation, were significantly enriched in the high mutation group (Figure 5A). In contrast, some pathway terms related to immune depletion, such as fatty acid synthesis, fatty acid metabolism and regulation of fibroblast proliferation, were significantly downregulated in the high mutation group (Figure 5B). Additionally, some carcinogenic pathways, such as the canonical WNT pathway and the NOTCH pathway, were significantly upregulated in the low mutation group compared with the high mutation group (Figure 5C).

## Discussion

To date, with the gradual increase in in-depth research on immune checkpoints, breakthroughs have been made in the research of ICIs, which have revolutionized the diagnosis and treatment of NSCLC; however, many challenges remain in clinical application, such as the limited population that benefits and the lack of effective biomarkers (Garon et al., 2019; Garassino et al., 2020). In the TME, both tumor cells and immune cells can undergo metabolic reorganization to adapt to a microenvironment with low oxygen, acidity and low nutrition (Wu and Dai, 2017). The activity of the lipid metabolism pathway can affect the recruitment, infiltration and activation of TILs (Yang et al., 2016; Saleh and Elkord, 2019; Jiang et al., 2020). Novel treatments that regulate lipid metabolism may effectively improve the immunotherapy efficacy and patient prognosis. In this study, we found that a high number of mutations in the lipid metabolism pathway was related to a favorable prognosis in patients with NSCLC receiving ICIs. Next, we analyzed the potential relationships between the number of mutations in the lipid metabolism pathway and immunogenicity and the TME. Patients with a high number of mutations in the lipid metabolism pathway had significantly enhanced immunogenic factors (such as TMB, NAL, and DDR pathway mutations) and enriched activated immune cells with upregulated inflammatory gene expression profiles.

The inflammatory TME in patients with a high number of mutations in the lipid metabolism pathway may be related to a better prognosis with ICI treatment. Compared with patients with a low number of mutations in the lipid metabolism pathway, patients with a high number of mutations had significantly increased proportions of infiltrating activated immune cells [macrophages (M0- and M1-type), CD4 + T cells (activated memory), CD8 + T cells, Tfh cells, and gamma delta T cells] and upregulated inflammatory expression profiles (IFNG, CD8A, GZMA, GZMB, CXCL9, and CXCL10). Tumor cell necrosis induced by the perforin-granzyme pathway and tumor cell apoptosis induced by the Fas-FasL pathway are regarded as two vital mechanisms by which CD8 + T cells exert antitumor immunity. Additionally, CD8 + T cells can also induce iron-mediated tumor cell death by secreting IFN-γ, which is a newly identified method of cell death that differs from apoptosis and necrosis (Dixon et al., 2012). IFN-γ can downregulate the expression of two subunits of the glutamate-cystine antiporter on the surface of tumor cells, namely, solute carrier family 3 member 2 (SLC3A2) and SLC7A11, thereby inhibiting tumors. Cystine uptake by the cell reduces glutathione synthesis and ultimately leads to insufficient synthesis of glutathione peroxidase 4 (GPX4), which inhibits the cell from effectively removing peroxide. Lipids cause iron-induced death in cells under iron-dependent conditions (Friedmann Angeli et al., 2019; Wang et al., 2019). IFNγ is mainly derived from CD8 + T cells and is also an important cytokine for CD8 + T cells to complete immune-mediated killing. In addition to mediating iron-induced cell death, IFN-γ can also promote antigen presentation and tumor cell killing. IFN-γ can activate the JAK-STAT signaling pathway through interferon receptors acting on tumor cells, thereby upregulating the expression of interferon-stimulated genes (ISGs) and enhancing major histocompatibility complex I (MHC-I) expression on the cell membrane. The expression of MHC-I molecules and intracellular immune proteasomes promotes the recognition of tumor cells by immune cells and simultaneously sensitizes tumor cells to apoptosis signals, which ultimately leads to tumor cell death (Quail and Joyce, 2013; Schneider et al., 2014). M1-type macrophages highly express TNF, inducible nitric oxide synthase (iNOS), MHCII and other proteins, which play an antitumor effect. Chemokines (CXCL9 and CXCL10) play an important role in recruiting CD8 + T cells and NK cells to the TME. The above results suggest the presence of an inflammatory immune microenvironment in the high mutation group (Lin et al., 2019).

The significantly enhanced immunogenicity in patients with a high number of mutations in the lipid metabolism signaling may be associated with a favorable prognosis with ICIs. Mutations in the DDR signaling can contribute to the up-regulation of genome instability and cause accumulated DNA damage, which may be a biomarker for identifying potential ICI responders in multiple cancer types (Teo et al., 2018; Wang et al., 2018). Patients with advanced urothelial cancers with mutations in the DDR pathway had a significantly increased ORR to immunotherapy (67.9% vs. 18.8%; *P* < 0.001) (Teo et al., 2018). Additionally, Wang et al. (2018) found that patients with co-mutations in the DDR pathway had significantly prolonged OS and PFS compared with patients without co-mutations. The TMB has been regarded as a potential molecular marker for predicting ICI response, and its utility has been gradually confirmed (Jessurun et al., 2017). An increased TMB can promote the production of more tumor neoantigens (McGranahan et al., 2016). Neoantigens are presented to DCs, which can promote the transformation of T cells into mature and activated T cells, and high NAL is associated with sensitivity to anti-PD-1/CTLA-4 treatments (Lin A. et al., 2020). In this study, we found that patients with a high number of mutations in the lipid metabolism pathway had a significantly increased TMB, NAL, and mutations of the DDR pathway. Therefore, the above results suggest that up-regulated immunogenicity may be a strategy generating favorable prognoses for NSCLC patients with a high number of mutations in the lipid metabolism pathway. This study analyzed the prognosis of ICI treatment and mutation status of lipid metabolism in patients with non-small cell lung cancer and attempted to elucidate the potential role of a high number of lipid metabolism mutations as a biomarker for screening the predominant population of NSCLC preferred for immunotherapy; however, this study still has several limitations. First, this work included only one ICI-treated cohort of NSCLC, which may introduce bias when screening biomarkers for the prognosis of ICIs of NSCLC. Second, targeted sequencing (MSK-IMPACT) was used to detect somatic mutations in the ICI-treated cohort and included significantly fewer gene mutations compared to whole-exome sequencing (WES). Third, this study cannot separate the effect of the TMB or the mutation counts of DDR signaling from the effect of the mutation status of lipid metabolism on the prognosis of NSCLC patients receiving ICIs. We hope to conduct relevant cell or animal experiments in the future to verify how a high number of lipid metabolism mutations affect the efficacy of immunotherapy and explore their relationship with the TME. We also hope to study NSCLC patients receiving ICIs to separate the effect of the TMB or the mutation counts of DDR signaling.

## Conclusion

Our study provided solid evidence that high-mutated lipid metabolism signaling was associated with prolonged PFS in NSCLC patients receiving ICIs. Hence, high-mutated lipid metabolism signaling can act as a potential biomarker for ICIs among NSCLC.

## Data Availability Statement

The original contributions presented in the study are included in the article/Supplementary Material, further inquiries can be directed to the corresponding author.

## Ethics Statement

Ethical review and approval was not required for the study on human participants in accordance with the local legislation and institutional requirements. Written informed consent for participation was not required for this study in accordance with the national legislation and the institutional requirements. Written informed consent was not obtained from the individual(s) for the publication of any potentially identifiable images or data included in this article.

## Author Contributions

XW: conceptualization. TC: formal analysis, visualization, and writing – original draft. TC, JZ, DL, and GL: writing – review and editing. All authors contributed to the article and approved the submitted version.

## Conflict of Interest

JZ, DL, and GL were employed by the company, HaploX Biotechnology. The remaining authors declare that the research was conducted in the absence of any commercial or financial relationships that could be construed as a potential conflict of interest.

## Supplementary Material

Supplementary Figure 1 KM survival curves for PFS in NSCLC patients (ICI-treated cohort) grouped according to TMB levels and mutation status of lipids metabolism.

Supplementary Figure 2 KM survival curves for PFS in NSCLC patients (ICI-treated cohort) grouped according to TMB levels.

Supplementary Figure 3 The mutual exclusivity analysis of lipid metabolism genes in the high and low mutation groups.

Supplementary Figure 4 Comparison of mutation frequencies of lipid metabolism genes between the high and low mutation groups in the ICI-treated cohort (Rizvi et al., 2018).

Supplementary Table 1 The mutation counts of lipid metabolism signaling of NSCLC patients in the TCGA-NSCLC cohort.
