# Supplementary figures and images for: Prognosis of Non-small-cell Lung Cancer Patients With Lipid Metabolism Pathway Alternations to Immunotherapy
Source: Front Genet. 2021 Jul 14;12:646362. doi: 10.3389/fgene.2021.646362 (PMC8317604; doi:10.3389/fgene.2021.646362)

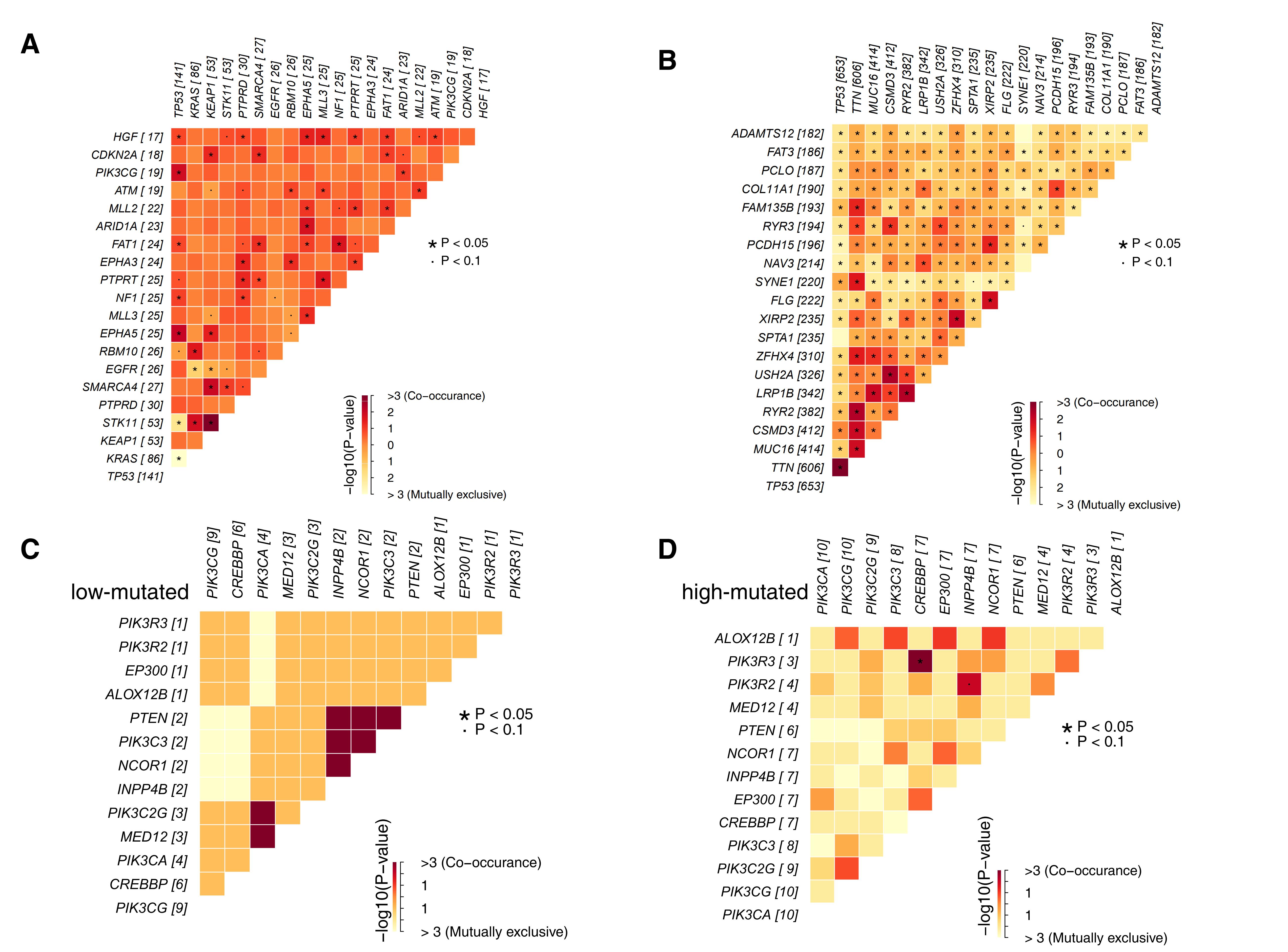

Supplement: Supplementary Figure 1 — KM survival curves for PFS in NSCLC patients (ICI-treated cohort) grouped according to TMB levels and mutation status of lipids metabolism. [file Image_1.JPEG]

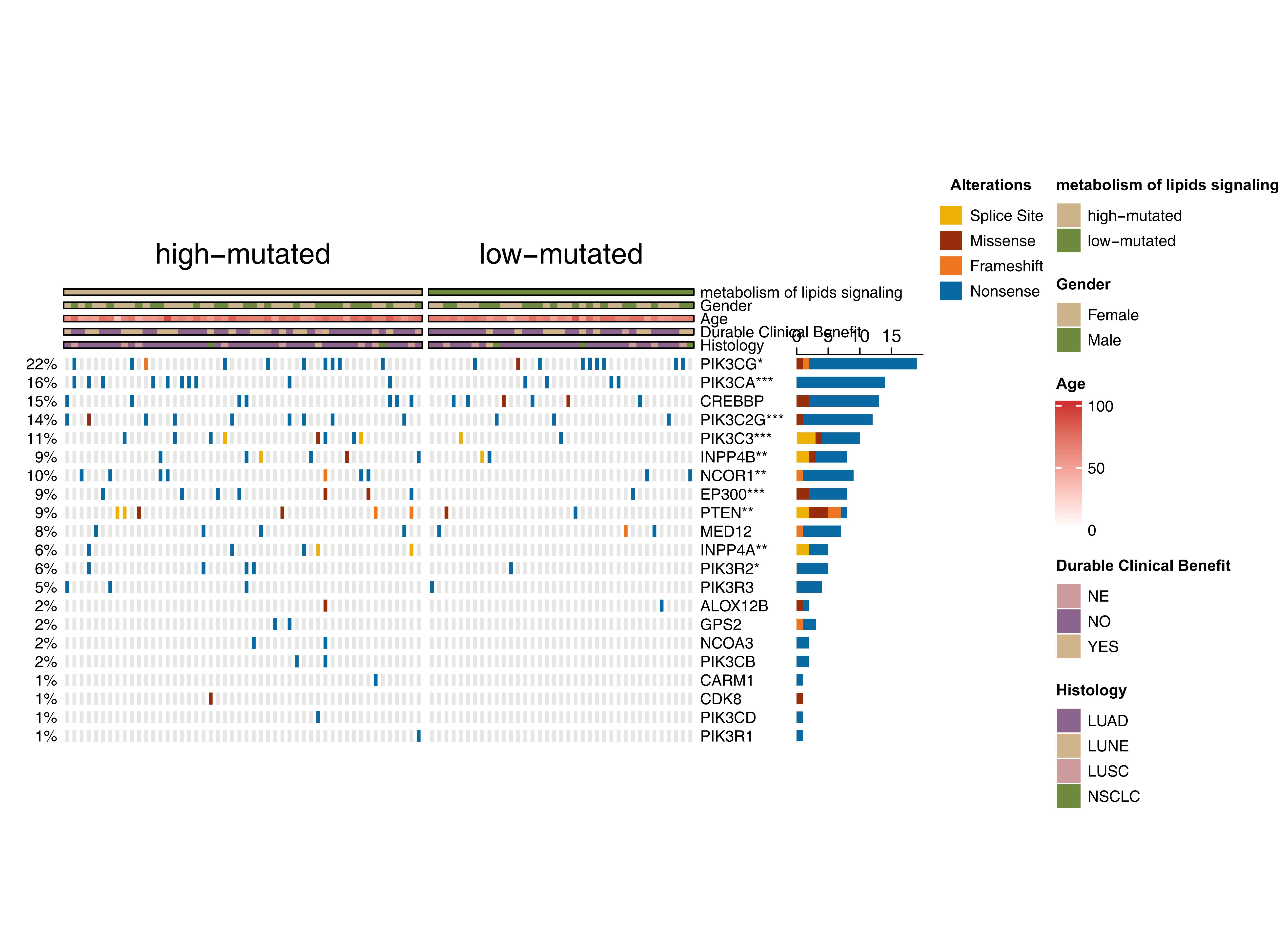

Supplement: Supplementary Figure 2 — KM survival curves for PFS in NSCLC patients (ICI-treated cohort) grouped according to TMB levels. [file Image_2.JPEG]

Survival Rate for OS

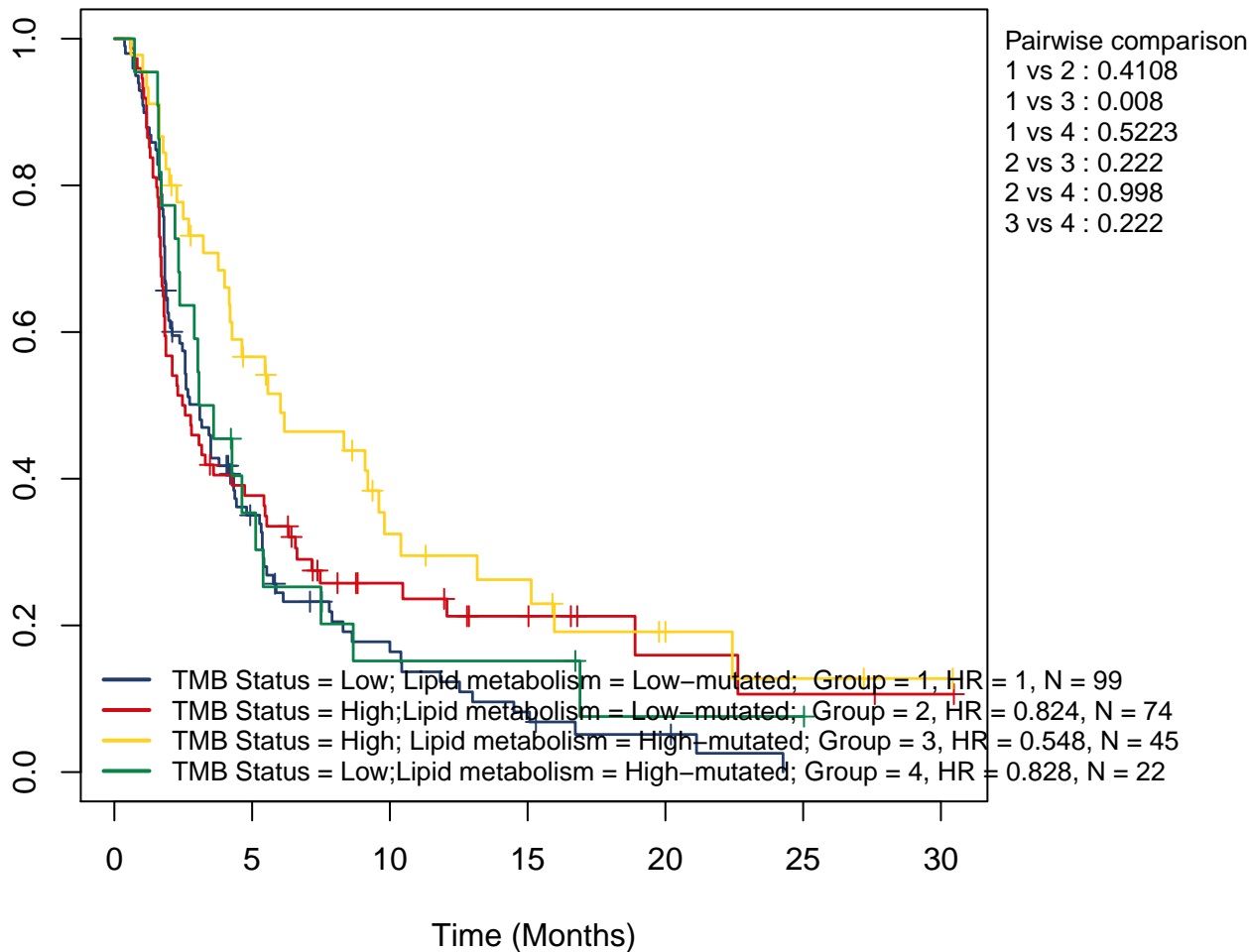

Supplement: Supplementary Figure 3 — The mutual exclusivity analysis of lipid metabolism genes in the high and low mutation groups. [file Data_Sheet_2.PDF]

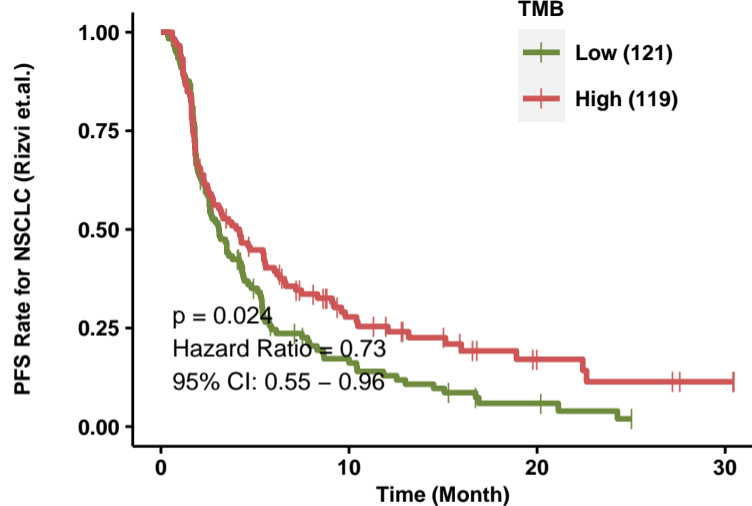

**Number at risk**

|     |    |   |   |
|-----|----|---|---|
| 121 | 16 | 4 | 0 |
| 119 | 23 | 7 | 2 |

Supplement: Supplementary Figure 4 — Comparison of mutation frequencies of lipid metabolism genes between the high and low mutation groups in the ICI-treated cohort (Rizvi et al., 2018). [file Data_Sheet_3.PDF]
