# Supplementary material for: Prognosis of Non-small-cell Lung Cancer Patients With Lipid Metabolism Pathway Alternations to Immunotherapy
Source: Front Genet. 2021 Jul 14;12:646362. doi: 10.3389/fgene.2021.646362 (PMC8317604; doi:10.3389/fgene.2021.646362)
Supplement: Supplementary file 7 [file Data_Sheet_1.PDF]

Pathways	Gene List	logPvalue	HR
path_1	ALOX12B,ARID1B,ATM,AXIN1,CDC42,CSF1R,CSF3R,FGF3,FGF4,GATA1,HIST1H3D,HIST1H3I,HNF1A,IKZF1,NOTCH2,PIK3CG,PRDM1,PTPRS,RAC2,SOS1	0.574693567	1.083462933
path_2	CASP8,CCNE1,EGFR,ESR1,H3F3C,HIST1H3E,KDM5C,KLF4,MALT1,NBN,NOTCH4,PIK3C3,PIK3CB,PIK3CD,PMS1,PPP6C,PTCH1,STK40,TCF3,TP53	0.785006136	0.948392863
path_3	ATRX,BCL2L11,CALR,CDH1,DAXX,EED,ICOSLG,MCL1,MET,MYCN,PIK3C2G,POLD1,RAD50,RARA,RHOA,SDHAF2,SHQ1,SOCS1,SOX9,STK40	0.332460933	1.147372554
path_4	CARD11,CD276,CEBPA,CREBBP,EPHA5,FBXW7,IDH1,IKZF1,INHBA,INSRR,MAP2K4,MAP3K1,MLL,MPL,NCOR1,NUF2,PHOX2B,SUFU,TNFAIP3,TSC1	0.17621576	1.253213108
path_5	ASXL1,AXL,BRD4,CDKN1A,E2F3,ERBB2,GLI1,INPP4A,KDM5C,MLL2,MST1R,PHOX2B,PIK3CB,PIK3R3,PIM1,PLCG2,PNRC1,PTPRS,PTPRT,SF3B1	0.054953975	1.367393427
path_6	ALOX12B,AR,ARID1A,ARID2,BRAF,CD79B,EIF4E,EPHA3,HIST1H3A,INPP4B,NKX3-1,NRAS,NTRK2,PAK7,PARP1,RAD51,SMAD3,SOCS1,STAG2,STAT5B	0.074684266	1.318706391
path_7	BCOR,CALR,CDC73,ELF3,FANCC,FBXW7,FGFR1,GATA2,GNAQ,HIST1H3A,INSR,MAP2K1,MDM2,MST1R,MUTYH,NOTCH4,PIK3CG,SETD2,SRC,TERT	0.789526566	1.044010981
path_8	ARID1A,ATM,BRCA2,CASP8,CTCF,CYSLTR2,FGF19,IRF4,JUN,KDM5C,KNSTRN,LATS1,MALT1,MAP2K1,MSH2,POLD1,PTCH1,RAD51C,SRC,SUFU	0.322907513	1.180518157
path_9	AKT3,CDC73,DIS3,EGFR,ELF3,FOXA1,HIST1H3E,HNF1A,HOXB13,IKZF1,IL10,JAK2,KDR,PPM1D,RHEB,SDHC,SOCS1,SOX2,STK40,TET2	0.776980895	0.960946523
path_10	BCL2,BLM,BMPR1A,CDK4,E2F3,HIST1H3B,HIST1H3C,HNF1A,KDM6A,MSH6,MST1,NFE2L2,NOTCH3,PIK3R1,PMS2,PPP6C,PRDM1,SDHA,SMAD3,TGFBR2	0.357896766	1.140813454
path_11	BRAF,CDKN2A,EED,FANCC,FGF19,FGFR4,FLT1,GRIN2A,HIST1H1C,HIST1H3I,MYOD1,NTRK1,PHOX2B,PTCH1,PTPRD,RAD54L,SMAD2,SOX2,SRC,STK11	0.18060577	1.214637234
path_12	CENPA,CHEK2,EIF4E,ERCC5,FBXW7,FGFR3,GNAS,HNF1A,IGF1R,IKZF1,KNSTRN,MEF2B,MST1R,MTOR,NFE2L2,PPP2R1A,RPTOR,SHQ1,SOX9,SUFU	0.055908806	1.316389002
path_13	BRCA2,DNMT3A,FLT3,GRIN2A,INHBA,KDM5C,KEAP1,MDM4,MYOD1,NF2,NPM1,PAK1,PIK3CB,PIK3R3,PTPN11,RBM10,RPTOR,SMARCD1,TAP1,TCF7L2	0.682662584	1.060445049
path_14	BCL2,CARM1,CXCR4,FOXL2,FUBP1,HIST1H3C,INPP4B,KRAS,LATS2,MAP3K1,MET,MTOR,NOTCH2,PAK1,RAC2,SH2B3,SLX4,SOCS1,STAG2,TP53BP1	0.104870126	1.279911735

path_15	BCOR,BMPR1A,CCND1,CCNE1,CDC42,CDH1,FANCA,FAT1,FGF3,FGFR1,FOXP1,INHBA,KMT2D,NOTCH1,NTRK2,PDCD1,PNRC1,RASA1,SHQ1,TAP1	0.620861053	1.088406578
path_16	AKT3,AMER1,BMPR1A,CDK12,DIS3,EIF1AX,EPHA3,KDR,MET,MITF,MSH3,NF1,PBRM1,PIK3CG,PMAIP1,PREX2,RAD51,RTEL1,SMAD3,SMARCD1	0.085835883	1.299343497
path_17	ABL1,CEBPA,DAXX,DNMT3A,EIF4E,EPHA3,FGF19,GNAQ,HIST1H3D,HLA-A,IFNGR1,KEAP1,MAP3K13,MGA,NTRK1,PIK3R3,PNRC1,POLE,TBX3,TGFBR2	0.33388848	1.152507835
path_18	ARID1B,ERCC2,FBXW7,FLT4,HIST1H1C,HIST1H3I,INSR,MLL2,MRE11A,PDGFRA,PGR,PIK3C3,PIK3R3,POLD1,PRDM1,RBM10,SLX4,SMARCA4,TGFBR1,TP63	0.533819245	1.095506181
path_19	ATR,AXIN1,CD274,CDKN1B,DNMT3B,ERCC4,ESR1,H3F3C,HIST1H3A,IGF2,INHA,INSRR,JAK2,NCOA3,PAK7,PIK3CA,PNRC1,RAF1,TP53,TP63	0.838678318	1.029487041
path_20	ABL1,CENPA,CHEK2,CTCF,DNMT3B,EIF1AX,ELF3,EZH2,FANCA,GATA2,ICOSLG,KNSTRN,MAX,MDM4,MYC,POLE,RARA,RB1,RPS6KA4,STAG2	0.434676892	1.159407138
path_21	ACVR1,CDK6,ERG,FGFR3,GATA2,GNAQ,IDH1,INHBA,IRF4,MRE11A,NCOA3,NEGR1,NOTCH2,PALB2,PIK3C3,PPP2R1A,RBM10,RECQL4,TCF3,TP53BP1	0.07627331	1.381538042
path_22	ABL1,ALK,CDK12,CRLF2,DDR2,EIF4A2,EPHB1,ERBB2,FGFR1,GLI1,GNAS,ICOSLG,KEAP1,LATS1,MAP2K2,MYCL1,NTRK3,PAK1,PIK3CG,RAF1	0.99976873	0.999957919
path_23	ASXL1,AXIN1,BCL2,CALR,CD79B,EED,EPHA5,EPHA7,IGF2,JAK1,MET,MITF,MST1R,NEGR1,NFKBIA,PTPRT,RHOA,SMARCB1,TAP1,TGFBR1	0.070942248	1.327305334
path_24	ARAF,ASXL2,BCL10,BCL2,BCL6,CTCF,CXCR4,EPHA3,ERG,FYN,GATA2,HIST1H3A,INPP4B,KMT2D,KNSTRN,NOTCH1,PIK3C3,RAD54L,SMARCA4,SRC	0.085337663	1.283683242
path_25	ASXL2,ATR,ATRX,CDC42,EIF1AX,ERRFI1,FOXL2,HIST1H3A,INSRR,NUF2,PARK2,PDCD1,PIK3R3,PPP6C,PTPRT,RAD54L,RARA,RFWD2,SDHA,SDHAF2	0.055779428	1.397319062
path_26	BTK,CEBPA,CYSLTR2,DDR2,DNMT3A,FBXW7,GATA1,GNAS,HIST1H1C,IGF2,KLF4,MALT1,MITF,MLL3,NCOR1,NOTCH1,PIK3CG,RAD54L,RB1,SDHC	0.737193631	1.051576332
path_27	BRAF,CDH1,CDK4,DICER1,E2F3,FAM46C,FANCC,FGFR2,GATA1,ICOSLG,MDM2,MYC,NOTCH2,NUF2,PHOX2B,PLCG2,PMAIP1,PMS2,PTEN,RAD51B	0.059374768	1.315913559
path_28	BRAF,CBL,CD276,CD79B,CDK4,CDK8,CTLA4,DAXX,ERBB2,ERBB3,ERCC5,FGF3,HIST1H3G,IGF2,INHBA,JAK3,NFE2L2,NKX2-1,SOX9,TET1	0.173466373	1.211777898

path_29	ANKRD11,AXL,BRCA1,CSF3R,DNAJB1,EP300,ERBB3,HIST1H3A,JAK2,KMT2D,NKX2-1,PIK3CA,PIK3R1,PMS2,RAD21,RAD51D,RHEB,SMARCB1,SMO,SPEN	0.59459314	0.918276029
path_30	AXL,BMPR1A,DDR2,DNMT1,DOT1L,EED,EPHA5,HIST1H3C,INPP4A,INSR,KMT2A,PAX5,PNRC1,PTCH1,PTPN11,PTPRD,RET,RHEB,SETD2,SOS1	0.099258113	1.316515846
path_31	CALR,CARM1,CDC42,CHEK1,FLT4,GATA2,GSK3B,HIST1H3J,MLL,MLL3,MYCN,NCOR1,NF1,NOTCH2,PDGFRA,PTCH1,RBM10,REL,RET,SPEN	0.069712596	1.311920952
path_32	BRAF,BRD4,CD276,DNMT1,ERCC4,FLT3,FOXP1,INHBA,KEAP1,KMT2D,MAP3K13,MED12,MLL2,RPS6KB2,RYBP,SDHAF2,STAT5B,TCF3,TP53BP1,TRAF2	0.20070718	1.226817163
path_33	CDK4,FANCA,FBXW7,FLT3,FOXA1,IGF1R,MEN1,MPL,MRE11A,MYOD1,NFE2L2,NRAS,NSD1,PAK1,PDCD1,PIK3R2,RYBP,SDHA,SMARCB1,SPEN	0.1162814	1.247551232
path_34	ATRX,BCL2L1,CCND3,CDK8,DNMT3B,FAM46C,FAT1,FOXP1,GNAQ,IGF1R,IKZF1,KRAS,MYC,MYCN,NCOR1,NKX3-1,NOTCH2,RASA1,RNF43,TEK	0.080344597	1.286008375
path_35	AKT2,ASXL1,BARD1,CIC,CTCF,EGFR,FAM46C,IGF2,JAK2,MET,PHOX2B,PIK3C3,PLK2,PMAIP1,PTEN,REL,RFWD2,RHEB,RNF43,SMARCB1	0.663774478	1.071781985
path_36	AKT2,ARID2,BRCA2,CCND1,CCNE1,CXCR4,DICER1,E2F3,EIF4E,ERCC5,ETV1,FANCA,FH,JUN,MAP2K2,MYCL1,NTRK2,NTRK3,PARP1,PGR	0.065392715	1.298165178
path_37	AKT3,ALOX12B,ARID2,ATM,BLM,CSF1R,DOT1L,DROSHA,EIF4A2,FAM175A,FGF3,FUBP1,HOXB13,LATS2,MDM2,MLL,NBN,NOTCH1,PIK3CG,POLD1	0.840632386	1.031863174
path_38	AKT3,ATM,CALR,CD79B,FANCA,FGFR2,FLT4,FOXP1,HIST1H3A,HIST1H3E,HIST1H3G,INPP4B,MEF2B,NOTCH1,PAK7,PARK2,PAX5,RECQL4,RUNX1,RYBP	0.411544642	1.147096661
path_39	ARID5B,AXIN2,CDC73,CEBPA,EGFR,EIF4E,ERBB3,FUBP1,FYN,GLI1,HIST1H3D,IFNGR1,IGF2,MAP3K13,PBRM1,PIK3R1,PNRC1,PPP6C,PTPRS,RAD50	0.34730247	0.876437788
path_40	ARAF,CCND3,CDK12,CRLF2,CTCF,DNMT3B,FGFR1,HIST1H3C,HIST1H3D,HOXB13,JUN,KMT2D,MST1R,NF1,PNRC1,PPP2R1A,PPP6C,RBM10,SMAD3,SMARCA4	0.58270428	1.090422111
path_41	AMER1,ATM,BARD1,CDH1,CDKN1A,CTCF,GSK3B,HIST3H3,ICOSLG,MYCN,NOTCH2,PALB2,PAX5,RPS6KA4,RTEL1,SDHAF2,SOX2,TCF7L2,TET1,U2AF1	0.324445278	1.150003184
path_42	AMER1,ATR,BMPR1A,CDKN2C,CYSLTR2,DAXX,DROSHA,ERCC4,ETV1,FOXL2,HLA-A,JUN,KLF4,MST1R,NRAS,PALB2,PGR,PPP6C,SH2D1A,SUFU	0.114569424	1.251594367

path_43	ARAF,AURKA,BRCA1,CARM1,CD276,CUL3,DROSHA,ERG,EZH2,FGFR2,FGFR3,GRIN2A,HIST1H3H,KDM6A,KEAP1,MAP3K13,MRE11A,PIK3R3,TBX3,TNFAIP3	0.719974112	1.061322993
path_44	CCNE1,CHEK2,CYSLTR2,DDR2,EPHB1,FLT1,FOXP1,KLF4,MPL,MYCN,NEGR1,NF1,NOTCH1,NOTCH3,NSD1,PIK3C3,PLK2,RAD21,RPS6KB2,SYK	0.133678988	1.283187931
path_45	APC,BCL2L1,CDKN1B,DNMT3B,ERRFI1,ETV1,FGFR1,FGFR3,FOXL2,FYN,HIST1H3I,IKBKE,INSRR,IRF4,MYC,PARK2,PDGFRB,REL,RHEB,TAP1	0.746211592	1.047899345
path_46	AKT3,BCOR,BRCA1,BRCA2,CENPA,ERBB4,FBXW7,HIST1H3G,HNF1A,JAK3,KDM5C,PDGFRA,PIK3CD,RAD51,SF3B1,SH2B3,SOX17,STK11,TERT,TP53	0.12542487	1.268754218
path_47	BARD1,BRCA1,BTK,CENPA,ETV1,HIST3H3,IGF2,IRS1,MEF2B,NFKBIA,NOTCH1,NRAS,PIK3CA,PTPRT,RAD50,SH2B3,SMARCB1,SRC,TBX3,TET2	0.490255213	1.11556987
path_48	AXIN1,CD79B,CDC73,DNMT3B,ERBB4,ERCC5,EZH2,GATA1,H3F3C,IDH1,KNSTRN,MEF2B,MSH3,NKX3-1,NOTCH2,NUF2,PIK3CD,SMAD4,SMARCA4,STK11	0.307397307	1.165042171
path_49	BARD1,BBC3,CDK4,CDKN2A,EPHA5,FGF3,FOXA1,HIST1H3J,MAP3K1,MAP3K13,MRE11A,NEGR1,NKX2-1,PDCD1,PIK3C3,PIK3R3,PTCH1,RNF43,SDHAF2,SUFU	0.172994535	1.238995187
path_50	AKT1,APC,BCL2,BMPR1A,CTLA4,ERBB4,ERCC2,IFNGR1,IL10,JAK1,MITF,PHOX2B,SDHC,SF3B1,SOX17,STK11,SUFU,SYK,TET2,YES1	0.557651222	1.09998288
path_51	ALK,AXIN1,BCL6,CDKN2C,CTNNB1,FH,GATA2,GNA11,MDM4,MGA,MSH3,MYC,NCOA3,NRAS,NUF2,PDGFRB,PNRC1,SPEN,STAG2,STAT5B	0.488376215	1.13364656
path_52	AKT1,AKT2,AURKA,CDC73,CENPA,CHEK1,CHEK2,EED,GATA1,KDM5A,MAP2K1,MGA,NKX2-1,PARP1,SDHA,SMAD2,TGFBR1,TOP1,TSC1,TSC2	0.095316519	1.268453672
path_53	ALOX12B,APC,BAP1,BRD4,CDC73,CDK4,CHEK1,CYSLTR2,FGF3,FGF4,GLI1,HLA-A,JAK1,MDM2,MEF2B,MGA,NF1,PIK3CB,RAD21,RET	0.170082166	1.299446348
path_54	ASXL2,BMPR1A,BRIP1,CD276,DDR2,ERRFI1,FBXW7,H3F3C,IGF2,INSR,KLF4,KNSTRN,MLL2,NEGR1,RPTOR,SHQ1,SOX17,SOX9,TMPRSS2,TSC2	0.417749651	1.121270303
path_55	ANKRD11,BCL2L11,CDKN1A,EIF1AX,ERCC4,ETV1,HIST1H3D,HIST3H3,HOXB13,KDR,MAX,MGA,MUTYH,NFKBIA,PAK7,PARP1,PBRM1,RAD51C,SUFU,SYK	0.463633003	1.14682677
path_56	ALK,ATR,AXL,CDK12,ERCC4,KDM5C,KMT2D,MAX,MEF2B,MST1,MST1R,NCOR1,NPM1,PALB2,PMS2,RHEB,SDHAF2,SDHB,SMARCB1,TCF7L2	0.467738814	0.902594021

path_57	ATRX,B2M,BAP1,CCND1,CDK12,EIF1AX,ETV6,GNAS,KMT2A,KNSTRN,MAP2K1,MAP2K2,MDC1,NSD1,SOCS1,SPEN,SUFU,TGFBR1,TNFAIP3,TRAF2	0.159226588	1.217884007
path_58	ATR,AURKA,B2M,CCND1,CHEK1,DOT1L,EED,ERG,FANCC,IL7R,JAK1,KRAS,NCOR1,PNRC1,PPP6C,SH2D1A,SLX4,TBX3,TGFBR2,TSHR	0.163089377	1.232491855
path_59	AKT2,ALK,AMER1,BCOR,CDKN2A,CHEK1,CIC,CREBBP,EZH2,HIST1H3I,JUN,KMT2A,MAP3K13,MEF2B,PAK7,PAX5,PREX2,RBM10,SETD2,SUFU	0.731082945	1.051429494
path_60	ASXL1,BMPR1A,CIC,ERG,ETV6,FLT4,FOXA1,FOXO1,GRIN2A,GSK3B,HGF,KMT2A,MITF,MUTYH,PDGFRA,PIK3CA,POLE,PPP6C,RBM10,TP53BP1	0.142339975	1.25595796
path_61	ANKRD11,BRAF,CCNE1,DNMT3B,DOT1L,ERBB3,GATA3,HIST1H3B,INHBA,IRS1,MTOR,NFE2L2,NTRK1,RAD21,RHEB,ROS1,SH2B3,TCF3,TSC2,TSHR	0.06925226	1.407756877
path_62	AMER1,ATM,BCL2,BCL2L11,CD274,CDC42,CDKN1B,CHEK2,FGF19,HLA-A,INPP4A,IRS2,KLF4,MGA,MYC,NKX3-1,PAX5,PLCG2,RECQL4,SDHA	0.116226796	1.320682292
path_63	ASXL2,CSF1R,CXCR4,EIF4E,EPHB1,GATA1,GNAS,GRIN2A,HIST1H3A,IRS1,KMT2D,MAX,MDM2,NOTCH1,NOTCH2,PIK3R3,PLCG2,PTPRD,SDHA,SMO	0.110794188	1.303722752
path_64	AXIN2,BARD1,CALR,CTLA4,CUL3,DOT1L,E2F3,GATA3,HIST1H3I,IFNGR1,KDM5A,MCL1,MYCL1,NOTCH4,NRAS,PTCH1,RNF43,ROS1,RTEL1,SH2B3	0.36418626	1.182883028
path_65	AKT2,AURKA,CENPA,DAXX,EGFR,EIF4A2,EPHA7,EPHB1,HIST1H3E,IFNGR1,JAK1,KNSTRN,MRE11A,PARK2,PGR,PNRC1,PPM1D,RBM10,RHEB,TCF3	0.392812026	1.149546608
path_66	APC,BBC3,BRAF,EP300,FUBP1,FYN,GNAQ,IKBKE,MAX,MDC1,NBN,NUF2,PMS1,RAD51D,RHOA,RPS6KA4,SDHC,STAG2,SYK,TGFBR1	0.724072743	1.051214976
path_67	ANKRD11,ARID1B,ARID2,ASXL1,CDK12,CUL3,DOT1L,ERCC5,FOXO1,GATA1,GRIN2A,H3F3C,HIST1H3I,NFKBIA,PAK1,PHOX2B,PIK3CA,PIK3R1,RB1,RBM10	0.653920345	1.072339217
path_68	AMER1,BCL2,BCOR,BRCA1,CD276,CDK12,CDKN1B,CDKN2A,CUL3,DROSHA,EPHA3,FOXA1,FYN,GATA1,KMT2D,PAK7,PPM1D,RAD21,SETD2,U2AF1	0.058498974	1.344786248
path_69	ARID1B,ATR,BARD1,BMPR1A,CARD11,CD274,CEBPA,CRLF2,ESR1,FUBP1,GPS2,IFNGR1,KMT2A,MAP3K1,NTRK2,PHOX2B,RPTOR,STK11,STK40,SUFU	0.180948347	1.251994863
path_70	ABL1,ANKRD11,CDKN1B,EGFR,ERBB3,FANCC,FOXL2,H3F3C,INSRR,MAP2K4,MST1R,NTRK3,PGR,PIK3R3,PTPRS,RAD51,RAD51C,RB1,RECQL4,SPEN	0.078918148	1.353123494

path_71	BCL2L1,CHEK1,CTNNB1,DNMT3B,GLI1,GPS2,GRIN2A,H3F3C,HIST1H3D,LATS1,MAP3K13,MDC1,NCOR1,NPM1,PARK2,PGR,PPP6C,RFWD2,RICTOR,SYK	0.345383518	1.170404371
path_72	ABL1,BCL6,BRCA2,CDK4,CSF3R,EIF4E,FOXP1,GNA11,GNAQ,GPS2,HNF1A,KLF4,KMT2A,LATS2,MYC,NSD1,PTPRD,RAD51D,RECQL4,TGFBR1	0.438488706	1.137010576
path_73	BAP1,CCND2,CDK12,CENPA,CYSLTR2,DAXX,DNMT3B,DOT1L,FGFR1,GLI1,HIST1H3B,KLF4,KNSTRN,MLL,NKX2-1,PAK1,PTEN,RAC2,SDHB,TSHR	0.308881526	1.154181577
path_74	ASXL2,BBC3,CALR,ERCC4,ETV6,FGF4,FGFR4,INSRR,KDM5C,MEF2B,NUF2,PAX5,PMS2,PNRC1,POLE,SOX17,TGFBR1,TMPRSS2,TNFAIP3,TRAF2	0.208081159	1.200719396
path_75	AMER1,CCND3,CDK6,CHEK2,EGFR,ERBB4,FANCC,FGFR4,GPS2,KDM5C,MALT1,MAP2K4,MAX,MTOR,NRAS,NUF2,PBRM1,PHOX2B,PLK2,PTCH1	0.912850017	0.982353882
path_76	CHEK2,DNMT3A,ERBB2,FBXW7,INHBA,MAP2K4,NBN,PAK1,PALB2,PDGFRA,RAD51B,RB1,SMARCB1,SPOP,SRC,STK40,TCF7L2,TEK,TMPRSS2,TP63	0.758500216	1.054565773
path_77	ALOX12B,ARAF,AXIN1,BCL2L11,DDR2,ESR1,FAT1,FUBP1,HNF1A,IGF2,MGA,MITF,MRE11A,MST1R,NCOR1,NOTCH2,NTRK1,RPS6KB2,STAT3,TMPRSS2	0.505720694	1.117603716
path_78	ALOX12B,BRIP1,GPS2,HIST1H3G,HNF1A,KMT2A,KRAS,MITF,NCOA3,NOTCH3,PALB2,PIK3R3,PLCG2,POLD1,POLE,PTCH1,RPS6KA4,SMARCB1,SOX9,SRC	0.152507204	1.238354054
path_79	ASXL1,BAP1,BLM,ELF3,EP300,FYN,GRIN2A,HIST1H3G,IGF1R,INPP4B,INSR,KDM5C,MST1,NSD1,RAD51C,RARA,RUNX1,SDHC,SMAD3,SOX17	0.320929017	1.150077452
path_80	AMER1,AXIN2,BCOR,BTK,CARD11,CCNE1,CDK8,CDKN1A,CHEK1,CSF1R,CSF3R,HIST1H3I,KRAS,MYOD1,NOTCH2,RARA,SMAD2,SMAD4,STK11,TRAF2	0.251746046	1.213863844
path_81	ALOX12B,BAP1,CASP8,CDH1,CREBBP,EGFR,ERBB3,ERCC4,ERCC5,GSK3B,MAP2K1,MSH3,PIK3CB,SDHC,SOS1,STK40,TAP2,TCF3,TET1,TP53	0.20156183	1.200847248
path_82	BCOR,CUL3,FGF19,FGFR4,FOXO1,IRS2,KDR,KEAP1,MEF2B,NUP93,PARP1,POLD1,RECQL4,RNF43,ROS1,RTEL1,SETD2,TGFBR1,TGFBR2,TP53	0.077456507	1.321353366
path_83	AKT2,ASXL1,ATR,CDK12,CDK4,CDKN2C,CSF1R,CTCF,DROSHA,E2F3,EIF1AX,ERBB3,MGA,MLL,MYOD1,NFKBIA,PAX5,PMS2,PPP2R1A,RAD50	0.742709319	1.047551428
path_84	AMER1,CDK8,CSF3R,ERRFI1,GRIN2A,HIST1H3B,HNF1A,HOXB13,INHBA,MITF,NCOA3,PAK1,PAK7,PIK3CB,PIK3R1,PTEN,RET,RHOA,SETD2,SH2B3	0.317445248	1.189386413

path_85	ACVR1,ALOX12B,ANKRD11,BCL2L11,CARM1,CDK12,EPHA3,FGFR2,GATA2,IGF1R,JAK2,MCL1,MST1R,NOTCH1,PDGFRA,PLCG2,PMS2,PPP2R1A,PTPRS,SH2B3	0.073967774	1.395367684
path_86	ARID1B,AXIN1,BCL6,CCND1,CCND2,DNMT3A,DROSHA,EPHA5,ERCC4,ETV1,FANCC,FBXW7,FH,HNF1A,PGR,PREX2,PTPRT,RAD50,RAF1,SDHC	0.245835603	1.221435714
path_87	B2M,BTK,CENPA,GATA1,IRS1,JUN,KLF4,KRAS,MDM2,NKX3-1,NOTCH4,NRAS,NSD1,NTRK1,NUF2,PLK2,SDHC,SH2D1A,SRC,STK11	0.968745115	1.006912539
path_88	CALR,CARD11,CBL,CDKN1A,CTNNB1,EGFR,EIF1AX,ELF3,EPHA3,FAT1,FOXA1,GRIN2A,INHBA,JAK1,KMT2A,MED12,NPM1,NUP93,PTEN,RFWD2	0.113601583	1.25977928
path_89	ANKRD11,BRAF,BTK,CBL,CDH1,CENPA,CIC,EIF1AX,IKBKE,JUN,MLL3,MYCN,NFE2L2,NFKBIA,POLE,PPP6C,RBM10,RET,SMARCD1,SOX9	0.128554232	1.283813613
path_90	ARAF,BLM,BRAF,BRD4,ESR1,GLI1,HIST1H1C,IFNGR1,JAK1,KDR,MPL,MRE11A,NSD1,PPP2R1A,PTPRT,RAF1,ROS1,SDHA,SRSF2,TP53BP1	0.107923973	1.325582942
path_91	AKT3,AMER1,AXL,BCOR,CEBPA,ERCC2,GNAQ,HIST1H3B,IFNGR1,INHA,INHBA,IRS1,KNSTRN,MST1,NUP93,PHOX2B,RAD21,SMARCB1,SOS1,TERT	0.711635333	1.054894343
path_92	ARID5B,BRCA2,BTK,CASP8,CDK4,CTCF,DICER1,EGFR,EPHA3,FGFR4,FOXO1,GPS2,IRS2,NCOR1,NF2,NUF2,RECQL4,RHEB,RPS6KA4,RUNX1	0.229489863	1.206979501
path_93	ABL1,CDK8,CTCF,DNMT1,ERBB2,FAT1,FGFR1,FLT4,FOXO1,IFNGR1,IL10,MED12,MPL,RAD51B,RASA1,RBM10,ROS1,SMAD3,SMARCB1,TCF3	0.149275141	1.259284112
path_94	ABL1,BAP1,BRCA2,EPHA5,ETV1,GATA1,GLI1,KDM5A,KLF4,MAP2K2,MAX,NPM1,NRAS,RAD54L,RECQL4,RHOA,RPS6KB2,RPTOR,SMARCB1,TMPRSS2	0.176435603	1.276359707
path_95	ATR,CCND1,CD79B,CDK6,FUBP1,HOXB13,KMT2D,NBN,NCOR1,NKX3-1,NOTCH3,NRAS,NTRK1,NUP93,PIK3C3,RAD51,RHOA,SMAD2,SMARCB1,TCF3	0.541576582	1.090737748
path_96	BCOR,CD276,CENPA,CTCF,EGFR,EIF1AX,FBXW7,FGF4,FOXL2,FYN,HGF,HIST1H3A,HIST1H3J,IRS1,KRAS,NOTCH2,NTRK1,RAD54L,TBX3,TCF3	0.364851865	1.137552797
path_97	AKT2,BMPR1A,CSF3R,CXCR4,DAXX,ERCC5,FAM46C,FGF3,FLT4,GNA11,GNAQ,MUTYH,NOTCH2,NUP93,PAK7,PGR,PLK2,SHQ1,SMAD4,TCF3	0.117274317	1.246542005
path_98	BARD1,BLM,CASP8,CSF1R,ERG,GLI1,IRS1,MAP2K1,MCL1,MLL3,MSH6,MYCL1,NFE2L2,NPM1,PGR,PTPRD,SMARCB1,SOX17,SYK,TBX3	0.167481068	1.256752502

path_99	ACVR1,AKT2,ASXL1,CTLA4,CYSLTR2,GNAQ,IKBKE,IRS2,JUN,KEAP1,KRAS,MED12,MSH6,NEGR1,NTRK3,PAK1,PLCG2,RAD51,RICTOR,SOS1	0.221982327	1.18952205
path_100	CASP8,CCND2,CDKN1A,DDR2,EPHB1,ERRFI1,FANCC,FGF3,HIST1H3D,HIST1H3H,KDM5C,MAP3K13,NTRK2,NUF2,PBRM1,PPM1D,PTPRS,RAD51C,RAD51D,TEK	0.521816473	1.095507937
path_101	ALK,BARD1,BCL6,BLM,CD274,CEBPA,EGFR,EIF4E,EPHA3,FLT1,JAK2,MALT1,MDC1,NKX3-1,PAK1,PTEN,RARA,RBM10,SOX2,STAT3	0.708268642	1.056301216
path_102	AXIN1,B2M,CCNE1,CDC42,CSF3R,EIF4A2,EPHA3,ERCC2,ERCC5,ERRFI1,IL7R,KDM5C,KMT2A,LATS1,MAP2K1,NTRK3,PALB2,RAD51B,REL,SLX4	0.259000035	1.228553384
path_103	BCOR,BRD4,CDK4,DNAJB1,EGFR,ELF3,ERCC2,HIST1H3C,HIST1H3G,IFNGR1,JAK3,MAX,MET,MRE11A,PARP1,PIM1,PTEN,SRSF2,STAT5B,TP53BP1	0.148018429	1.317636251
path_104	ABL1,ALOX12B,CALR,CARM1,EIF4A2,EZH2,FYN,IL7R,JAK1,JUN,NOTCH2,NTRK1,NUF2,PREX2,SDHC,SETD2,SMARCA4,TAP1,TEK,TET1	0.230342164	1.25072849
path_105	ARID1A,AXIN1,BCOR,CEBPA,CRLF2,DNMT3B,ERCC4,ERCC5,FOXO1,HIST1H3D,HIST3H3,NCOA3,NFKBIA,PDGFRB,PIK3R3,PNRC1,RAF1,RBM10,RECQL4,SMARCB1	0.072437166	1.351999222
path_106	ATR,CSF1R,CSF3R,EPHB1,ERG,FANCA,FYN,HIST3H3,IL10,INSR,JAK3,KNSTRN,MGA,MYCL1,NUF2,PIK3R3,REL,RYBP,SMARCB1,SYK	0.157063377	1.22408892
path_107	AXIN1,BRIP1,CTCF,DNAJB1,EZH2,FLT3,IKBKE,INHA,KRAS,MDM4,MED12,NEGR1,NRAS,NTRK3,PBRM1,PDGFRB,RAD51D,STK11,TEK,TOP1	0.258911498	1.221628612
path_108	ASXL1,BRAF,CALR,CREBBP,EGFR,ERCC5,FGF3,FH,GSK3B,ICOSLG,JAK1,MDM2,MSH2,PIK3CA,PLCG2,RAD21,SH2B3,SOS1,SOX17,TGFBR1	0.589052374	1.094554918
path_109	ANKRD11,ATM,ATR,AURKA,AXL,CHEK2,EPHA7,FGFR2,FGFR3,HGF,HIST1H3A,HIST1H3C,HNF1A,INHBA,JAK2,NOTCH3,PPP6C,RB1,RICTOR,TRAF2	0.834709387	1.034038083
path_110	ALK,ALOX12B,BCL6,CDKN2C,DNMT3B,EPHB1,ERCC2,GLI1,HIST1H3I,KDM6A,MALT1,PPP6C,RAD51B,RAD51D,RASA1,RNF43,SOX9,SYK,TBX3,TP53BP1	0.326003018	0.871529594
path_111	ARID1A,BCOR,CALR,CDC73,DNMT3A,ERCC2,FAM175A,GNAS,MAP2K4,MAX,NOTCH1,NRAS,PMS2,PPP6C,PTPRD,SMAD2,SMARCB1,SOCS1,STK11,TP53	0.392906785	1.13993248
path_112	ANKRD11,ARID2,CXCR4,DAXX,E2F3,EPHA3,ETV1,FANCA,GNA11,INPP4B,MSH3,MYOD1,NOTCH3,NOTCH4,NUF2,PDGFRA,PMAIP1,PPP6C,SH2B3,SMAD4	0.102926882	1.300469616
path_113	ALOX12B,BRAF,CASP8,CDC42,CDC73,EP300,ETV1,FAM46C,GATA2,HNF1A,MLL3,NSD1,PARP1,PBRM1,PIK3CB,RET,RNF43,RTEL1,TAP2,TSC2	0.423259022	1.119717372

path_114	AKT2,AMER1,CCND1,CDK8,CUL3,DICER1,EP300,ERBB2,ERCC5,FOXL2,HIST1H3E,KDM5C,LATS1,MAP3K13,NCOR1,PDGFRA,PIM1,RAD51C,RARA,RPS6KB2	0.130650762	1.330426611
path_115	ANKRD11,B2M,CALR,CEBPA,CIC,DAXX,IKZF1,IL7R,LATS2,MSH2,NRAS,PIK3CA,PTPRD,RAD51C,RB1,RFWD2,RPS6KA4,RTEL1,SMAD2,STAT3	0.929561729	1.015396385
path_116	AKT1,ALOX12B,BAP1,CALR,CHEK1,EGFR,JUN,MITF,MRE11A,NOTCH1,PHOX2B,PIK3CG,PIK3R1,RAD21,RET,RNF43,SMAD2,SYK,TCF7L2,TNFAIP3	0.510405729	1.128065432
path_117	ASXL2,CARD11,CCND3,EGFR,EPHA3,ERCC5,FYN,GATA2,MEF2B,NSD1,NTRK2,PBRM1,PIK3R2,PPP6C,PREX2,RBM10,RNF43,RPTOR,STAG2,TRAF2	0.108742472	1.278611886
path_118	ARID1A,ARID5B,BLM,BRD4,CARD11,CSF3R,CUL3,CXCR4,EPHA7,FGFR2,FH,HIST1H3J,IFNGR1,INPP4B,JAK1,MED12,MLL2,NTRK1,PIK3CD,RHEB	0.062000899	1.343815212
path_119	ALK,ATRX,CBL,CDK4,CIC,DICER1,ESR1,FGF19,FOXO1,H3F3C,HLA-A,KDM5C,MDM4,MET,PMS1,POLD1,PPM1D,PPP6C,RAF1,SMO	0.162557634	1.269200335
path_120	ASXL2,B2M,BBC3,BMPR1A,CCND1,CSF1R,CTLA4,EED,ERCC2,ERCC5,FGFR4,FH,HGF,HOXB13,NRAS,POLE,PRDM1,RAC2,REL,TP63	0.138558402	1.233197097
path_121	ACVR1,ANKRD11,ASXL2,CUL3,FANCC,FLT3,HIST1H3B,HIST1H3J,IGF2,JAK2,MAP3K13,MLL2,MPL,MYC,PDCD1,PMS2,SETD2,SMAD4,SMARCB1,TERT	0.411145573	1.141397484
path_122	AKT3,ALOX12B,AXIN1,CDK8,CDKN2A,CENPA,CHEK1,DICER1,DNAJB1,E2F3,ETV6,FYN,IRF4,MDC1,MEF2B,MPL,PPP6C,SLX4,SMARCD1,SRC	0.386691845	1.131920056
path_123	AKT1,BCL10,CDH1,CDK6,ERBB2,FGF19,FGFR1,FH,FOXP1,IGF1R,LATS2,NF2,PIK3R2,PMAIP1,PTPN11,RHEB,SH2D1A,SMAD3,SRC,TBX3	0.70281451	1.05532562
path_124	ALOX12B,CDH1,CTLA4,FLT4,FYN,HIST1H3E,MALT1,MEF2B,NCOR1,NFE2L2,PAX5,PIK3R2,PLK2,PREX2,RECQL4,SF3B1,SMAD2,STK11,TGFBR1,TRAF2	0.314019582	1.169365023
path_125	AKT2,APC,ARID5B,ATRX,CCND3,CEBPA,DNMT1,FOXO1,GLI1,GNA11,KDR,PDGFRB,PLK2,PTCH1,RBM10,RNF43,SPOP,TBX3,TET1,U2AF1	0.076859153	1.326671494
path_126	AXIN1,AXIN2,CDK12,CSF1R,HIST1H3I,IL10,MAP3K13,MLL2,MTOR,PRDM1,PTCH1,RAC2,RAD21,RAD50,RAD51C,RARA,RHEB,SUFU,TOP1,YAP1	0.762778713	0.958130724
path_127	AKT2,AKT3,AXIN1,CASP8,CHEK2,CIC,ERBB4,FH,GNA11,HIST1H3B,IL7R,KLF4,MDM4,PTPRD,RAD51,RNF43,RPS6KB2,SDHB,SMAD4,TBX3	0.065475905	1.354211274
path_128	ALOX12B,ARAF,CARD11,CDK8,CDKN2A,CYSLTR2,DROSHA,ELF3,EPHA7,FAM175A,HLA-A,JAK3,JUN,MCL1,MLL3,NKX2-1,PIK3C3,PMAIP1,RET,STK11	0.155449416	1.229836204

path_129	ARID5B,BAP1,CARD11,CDK6,CDK8,CYSLTR2,DNMT1,EED,EIF4E,FYN,MDM2,NUP93,PALB2,PIK3CG,PTPRS,RAD50,REL,ROS1,SH2B3,SOX2	0.053142707	1.313653964
path_130	AKT1,ATM,AXIN2,BARD1,BCL10,CIC,FAM175A,FGF19,MAP3K13,MPL,MSH6,NBN,NEGR1,PARP1,PDCD1,PDGFRA,PREX2,RAD21,SOX17,STAG2	0.814398765	0.967097325
path_131	BLM,BRCA1,ELF3,HIST1H1C,HIST1H3G,INHA,IRS1,JUN,KDM5C,KLF4,MDM2,NPM1,PDGFRA,PGR,PIK3C3,PIK3R3,ROS1,RYBP,TERT,WT1	0.123594336	1.242450627
path_132	ARID5B,CTNNB1,FAM175A,FGFR1,GATA3,HIST1H3D,IFNGR1,IL7R,JUN,KDM6A,MRE11A,NCOR1,PARP1,PTCH1,PTEN,PTPRS,SDHAF2,SOX2,TGFBR2,TMPRSS2	0.301183581	1.218775646
path_133	AR,CDK12,CDKN1A,CDKN2C,ERBB4,FAM46C,FGFR2,HIST1H3I,HNF1A,IDH1,MLL2,MPL,NF1,PIK3R1,PPM1D,RB1,RET,SOS1,SOX9,SRSF2	0.203500428	1.220034389
path_134	BAP1,BCL2L1,CDK12,CDKN2C,ESR1,FAM46C,FGF3,FGF4,GNAQ,HGF,HIST1H3J,IL10,KMT2A,MDM2,MEN1,MSH3,NOTCH1,PLCG2,PLK2,RAD54L	0.136505805	1.234718984
path_135	ARID2,ATRX,BCL6,BLM,CALR,CCND3,CENPA,CTLA4,EIF1AX,HLA-A,KLF4,KRAS,MITF,MST1R,NBN,NF2,PARP1,RHEB,SOX17,STAT3	0.096463361	1.274299347
path_136	ARID1B,ATRX,CALR,CD79B,DNMT3A,EPHB1,ERRFI1,FANCA,FGFR3,FH,HIST1H3D,INPP4A,MLL2,MRE11A,NF2,PDGFRA,RAD51B,RAF1,SLX4,SPEN	0.050533026	1.398413599
path_137	ABL1,ALOX12B,BCL2L11,BCOR,BRIP1,CDC42,EPHB1,FLT3,JAK1,MEN1,MET,MYC,NRAS,NTRK2,PDCD1,POLE,RAD51B,SHQ1,SMAD4,SUFU	0.236297054	1.220368359
path_138	AKT3,CCND3,CD274,CENPA,EPHA5,ETV6,GLI1,JAK2,MAP3K13,MITF,NSD1,PDGFRA,PHOX2B,PIK3C2G,PIK3R1,POLE,PPP6C,RAF1,RECQL4,TCF3	0.292354439	1.16098682
path_139	AXIN2,CEBPA,CHEK1,CYSLTR2,DROSHA,EIF4E,EP300,FLT1,HIST1H3A,NCOA3,NCOR1,NTRK3,PAX5,RBM10,RTEL1,SMAD3,SOX2,TCF3,TEK,TNFAIP3	0.18472819	1.264453743
path_140	ALK,AXIN1,BCL10,BRIP1,CCND2,CHEK2,CSF1R,CTCF,FANCC,GATA3,HIST1H3I,MLL3,MRE11A,MYOD1,NF1,PIK3CG,PIK3R3,PMS2,SETD2,TRAF2	0.54644418	1.096016838
path_141	ALK,AMER1,AR,ARAF,B2M,BBC3,CD79B,EPHA7,INHBA,MALT1,MDM4,MED12,NBN,NSD1,PARP1,PGR,PHOX2B,RASA1,RET,TBX3	0.051448655	1.392219499
path_142	BRAF,BRIP1,CARD11,CEBPA,CYSLTR2,DNMT1,EPHB1,HIST1H3A,ICOSLG,KDR,MAP2K4,MSH6,NSD1,PIK3C2G,PMS1,RAD21,RPTOR,SRC,STK40,YAP1	0.502523357	1.100006087

path_143	ALOX12B,BLM,BMPR1A,CD276,CSF1R,EPHB1,ETV6,INPP4B,KDM5A,MSH6,MYC,NFE2L2,NFKBIA,PIK3C2G,PNRC1,RARA,RPTOR,SOS1,SRC,STK40	0.152429007	1.225896884
path_144	BAP1,BTK,EPHA5,ERCC5,FAT1,FOXA1,IFNGR1,IRF4,KDM6A,KEAP1,MTOR,MYOD1,NF1,NFE2L2,PPP2R1A,RTEL1,SYK,TAP2,TNFAIP3,TP53BP1	0.216620431	1.19655478
path_145	ABL1,ARID2,CCNE1,CHEK2,DICER1,ERCC5,HIST1H3D,IGF2,MAP2K4,MDC1,NTRK1,PALB2,POLD1,RAD51C,SETD2,SMAD3,SMARCA4,SMARCD1,SOX2,SPEN	0.135297289	1.271870679
path_146	BCL2L11,BCOR,CDKN1B,CTLA4,E2F3,FANCA,FOXO1,HIST1H1C,HIST3H3,IDH1,MDM4,MLL,MYOD1,PIK3R3,POLD1,POLE,PTPRD,PTPRS,SHQ1,STK11	0.658066861	1.068730442
path_147	ABL1,APC,ARID5B,BCL10,CCND1,CD274,DNAJB1,FANCC,H3F3C,JAK1,KMT2D,MDM2,MLL3,NTRK2,PBRM1,PIM1,PTEN,RPTOR,SLX4,SOS1	0.301626019	1.210542787
path_148	AKT3,BCL2,CDH1,DICER1,ERBB2,FGF3,GNAS,HIST1H3B,IKBKE,LATS2,MAP3K1,MITF,PDCD1,PIK3R1,PMS2,PTPRD,RPS6KB2,RUNX1,SOX9,STAG2	0.082610415	1.363161487
path_149	CARM1,CDK4,CDK8,CSF1R,DAXX,FANCC,FGF19,FGFR4,FUBP1,HIST1H3E,KDR,LATS2,NOTCH1,PIK3C2G,PREX2,RAD51C,RYBP,SMAD2,SRSF2,STK40	0.306532952	1.160353328
path_150	ARID1B,CARD11,DDR2,ERBB2,FGFR4,GLI1,HIST1H3A,HIST1H3D,KDM6A,MCL1,MEF2B,MLL3,MUTYH,NTRK3,PHOX2B,PIK3R2,RAD51,RNF43,ROS1,SMO	0.077997353	1.33567979
path_151	APC,CARM1,DNAJB1,ESR1,HIST1H3H,IGF2,KDM5C,MAP2K4,MLL,MPL,MST1R,NFKBIA,NOTCH3,RAC2,RHEB,SOX17,SOX2,STAT3,TOP1,TSC1	0.627806443	1.07115159
path_152	AKT1,ARID5B,BAP1,BCL2L1,CDK4,CSF1R,EPHB1,GLI1,IFNGR1,IL7R,INPP4A,KDM5A,MALT1,PIK3R2,PNRC1,RAF1,RECQL4,RET,SDHA,TAP2	0.90825646	0.983440679
path_153	ATRX,BCL6,BLM,BRCA1,DDR2,DNAJB1,EGFR,EIF1AX,ERBB2,ETV6,FOXA1,GATA1,GATA3,GPS2,HIST1H1C,IKBKE,INSRR,MAP3K1,POLE,PPP2R1A	0.104259254	1.283673257
path_154	ABL1,ALOX12B,ASXL2,ATM,BRCA1,BRD4,CEBPA,CUL3,EIF4A2,FOXA1,FOXP1,HIST1H3D,IGF2,INHA,NUP93,PAK1,PMS2,POLD1,RB1,RTEL1	0.6460023	0.923190987
path_155	ABL1,BRCA1,CBL,CTCF,FGF4,FGFR2,FH,HIST1H3I,KLF4,MAP2K1,MRE11A,MUTYH,MYCL1,NFE2L2,PDGFRB,PIK3C2G,PTEN,RB1,RET,SF3B1	0.497623454	1.126507796
path_156	ARAF,ARID5B,BRCA1,CDK6,CDKN1B,CTNNB1,GNAS,HIST1H3B,IGF1R,IKZF1,PIK3CD,PIK3R2,PRDM1,RAD51B,SMARCB1,SMARCD1,SRC,TERT,TRAF7,WT1	0.678225772	1.061576027

path_157	ASXL1,EED,EIF4A2,EPHB1,FAT1,FGF3,FGFR1,IDH1,INSRR,MDM4,MLL,MRE11A,NRAS,NTRK2,NUF2,PDGFRA,RAD21,SDHC,SMARCD1,TAP2	0.419299779	1.120637828
path_158	ALK,ANKRD11,BCOR,CD274,CDKN1A,CDKN1B,CIC,CRLF2,DNAJB1,HIST1H3E,MCL1,NFKBIA,NPM1,NTRK1,PAK1,PIM1,RAD51B,RET,SOX2,TET2	0.442797425	1.117236642
path_159	B2M,BMPR1A,BRIP1,BTK,CD276,CDK4,CDKN1B,CTLA4,EIF4E,EPHA5,FLT3,GLI1,GPS2,KIT,MYCL1,PIK3CD,RAD21,RASA1,RPTOR,RTEL1	0.82738421	1.031239857
path_160	ALK,APC,ARID1A,ARID2,BBC3,CD276,CDK4,FGF4,GPS2,KIT,MRE11A,MYCL1,NCOA3,NTRK1,PIM1,PTPN11,RAD50,REL,RPS6KA4,RPS6KB2	0.912382764	1.020119711
path_161	AR,BTK,CASP8,CD274,CDKN1B,DAXX,FAM175A,GNAS,INHA,KDM6A,MRE11A,NOTCH4,PRDM1,PTPRD,SPOP,STAT5B,TAP1,TCF3,TRAF2,TRAF7	0.088797504	1.363107421
path_162	AKT3,BRCA2,CBL,CDK6,DDR2,EPHB1,FAM175A,FANCC,FBXW7,FGFR2,HIST1H3H,INHBA,KDM5C,KRAS,MUTYH,MYCL1,NFKBIA,PTPRD,STK11,SYK	0.354487615	1.160975767
path_163	AKT1,BAP1,BCL6,CASP8,CCNE1,CTLA4,CUL3,ERBB4,ERCC4,HGF,HIST1H3J,KIT,MALT1,MAP3K1,MCL1,NOTCH4,NPM1,NTRK2,PALB2,SLX4	0.407752566	1.150729261
path_164	AXIN2,BRIP1,CCND3,CHEK1,CTLA4,CTNNB1,DDR2,FGF3,GPS2,HIST1H3A,KMT2D,MPL,MSH3,NPM1,PHOX2B,RAC2,RAD51B,RICTOR,RPS6KB2,SUFU	0.277308783	1.17222389
path_165	AKT1,ATR,BCL2L11,BRCA2,CDK8,CTCF,DAXX,FH,FOXP1,HIST1H3G,KDM5C,MEF2B,MGA,MYOD1,PAK7,PRDM1,RAD51C,TERT,TP53,TP53BP1	0.500953696	1.100752022
path_166	ALOX12B,AMER1,ANKRD11,AR,ASXL1,AXIN2,CDH1,CDK8,DROSHA,EGFR,HOXB13,IKBKE,IL7R,IRF4,MITF,MLL,MUTYH,NFKBIA,PARK2,PNRC1	0.470827526	1.141146708
path_167	BARD1,CARM1,CDK6,CDKN1A,CTLA4,EPHB1,FAT1,FLT1,GPS2,HGF,MAX,NRAS,NTRK2,PAK7,PIK3R2,SHQ1,SMARCB1,SOCS1,SRC,TGFBR1	0.459387123	1.131564632
path_168	CDH1,CHEK2,H3F3C,ICOSLG,INHBA,MLL,MSH2,MYCN,NOTCH4,NSD1,PMS2,PTPRT,RAD50,RB1,ROS1,RYBP,SMARCB1,SRSF2,TMPRSS2,TNFAIP3	0.100345465	1.307314604
path_169	BRCA2,CCND3,CDK4,CDKN1B,CHEK1,DIS3,DNMT3A,E2F3,EIF4A2,ELF3,EPHB1,FAM175A,FGF3,JAK3,MITF,MLL,MPL,REL,RET,SDHAF2	0.222059308	1.191229688
path_170	AKT3,CCND1,CCNE1,CDKN2C,CTLA4,DNMT3B,EIF4E,EPHA3,ESR1,EZH2,FANCC,FLT4,FUBP1,HIST3H3,INHA,MYOD1,NTRK1,RUNX1,SRSF2,STAT5B	0.168375932	1.276100385

path_171	AXL,BRCA1,CENPA,DNMT1,FLT1,FLT3,HIST1H1C,HIST1H3J,INPP4B,JAK1,LATS1,MAP2K4,MITF,NEGR1,NOTCH3,NPM1,PIM1,RPS6KB2,SH2B3,TGFBR1	0.139504666	1.230607945
path_172	ARAF,CHEK2,FGF19,FGFR2,GNAS,H3F3C,HIST3H3,LATS2,MLL,MST1,NEGR1,NF2,NPM1,PIK3C2G,RAD51D,RPS6KA4,SRC,TBX3,TET1,TGFBR2	0.284649398	1.16370237
path_173	AURKA,BCL6,CCND1,CCNE1,EZH2,FGFR2,HIST1H3H,IGF1R,MDM4,MGA,MLL,MLL2,MPL,NTRK2,PLCG2,RAD21,RARA,RTEL1,SH2B3,SOX9	0.47133992	1.107221677
path_174	ALK,ARAF,BCL2L11,BRCA1,ESR1,FBXW7,FOXL2,HIST1H3E,INSRR,MITF,PDGFRB,PLK2,PNRC1,RAC2,RAD54L,RHEB,SMAD2,SOX2,SRC,SUFU	0.903461687	1.017611623
path_175	AXIN1,CDK12,CTLA4,E2F3,ERBB2,ESR1,FOXL2,INPP4B,JAK1,MAP3K1,NFKBIA,NOTCH3,NTRK1,RAF1,RET,SDHB,SOCS1,TMPRSS2,TRAF7,XPO1	0.38791333	1.129937933
path_176	ARAF,BAP1,BRIP1,CCND3,CDK12,CDKN1B,CHEK2,CUL3,DNMT3B,ERCC2,FOXO1,IRS1,MAP3K1,MSH2,PAK1,RAC2,RBM10,RECQL4,RICTOR,SHQ1	0.320525589	1.19902517
path_177	AKT1,CARD11,CCND2,CDC73,EPHA3,FAM46C,FANCA,HOXB13,INSRR,KDM6A,LATS2,MCL1,MDM2,MITF,PARK2,PTEN,RPS6KB2,SMARCA4,TAP1,TGFBR2	0.162808194	1.2522309
path_178	ABL1,B2M,CTLA4,DIS3,EIF4A2,ETV6,FBXW7,FGFR2,HIST1H1C,KDM5A,LATS2,MCL1,NCOR1,NF1,PAX5,PIM1,PREX2,RAD50,SUFU,WT1	0.146287847	1.284009642
path_179	CD274,EIF1AX,EPHA3,ERG,FAT1,KEAP1,LATS1,LATS2,MLL2,MSH2,NTRK2,PALB2,PARK2,PIK3CB,PTCH1,RAC2,RICTOR,SMARCA4,SPOP,SRSF2	0.641571866	1.068261888
path_180	BCL2,CCND2,CDKN2C,CSF3R,FLT3,IL10,IRF4,KNSTRN,MAP2K4,MDM2,PAX5,PMAIP1,PPM1D,PPP2R1A,RNF43,SETD2,SF3B1,SMAD3,SPOP,TAP2	0.116460568	1.251035264
path_181	B2M,BBC3,BRD4,CASP8,DNMT3B,E2F3,FBXW7,FOXL2,IL7R,KRAS,MALT1,MITF,MSH2,MSH6,NEGR1,PLK2,PNRC1,PPP2R1A,SDHC,SMO	0.329302257	1.18325856
path_182	DDR2,ERBB3,ETV6,FAT1,GNA11,ICOSLG,IDH1,KIT,MPL,MST1R,MTOR,MYCN,NKX2-1,PALB2,PARK2,PTCH1,RHEB,RICTOR,SDHA,SMAD2	0.84706073	1.033488594
path_183	ARID1B,BARD1,DDR2,DICER1,ELF3,EPHB1,ERBB2,GLI1,HIST1H3I,KDM5C,LATS2,MAP3K13,NRAS,PARK2,PDCD1,PDGFRA,PMS1,PTPRD,RAD51D,RBM10	0.668580189	1.065065434
path_184	AMER1,B2M,EGFR,ELF3,EP300,FGFR3,FOXL2,GRIN2A,INPP4A,JAK2,KDM6A,MLL,MLL3,MYC,NKX3-1,PDGFRA,PPP6C,PTPN11,RNF43,SDHB	0.990942422	0.998273197

path_185	ASXL1,BAP1,DNAJB1,DNMT3A,ERBB3,H3F3C,HIST1H3C,HIST1H3I,HNF1A,IFNGR1,KMT2A,LATS1,LATS2,MTOR,NUP93,PAX5,PIK3CD,PIM1,RPS6KA4,RUNX1	0.620040703	1.074263134
path_186	BCL6,BRCA1,CDH1,CDKN1A,CTLA4,CXCR4,EPHA5,EPHA7,FANCA,INPP4A,MITF,NFE2L2,PARP1,RAD51B,SMAD3,SOCS1,SOX17,SOX9,SRSF2,TGFBR1	0.236335053	1.238543757
path_187	ANKRD11,BTK,CBL,CDH1,CENPA,DNAJB1,EPHB1,GATA2,HIST1H3G,IL7R,JAK1,MYC,NOTCH4,PDGFRB,POLE,PTPRD,RARA,SDHA,SDHB,TSHR	0.419109079	1.138998131
path_188	AXIN2,BAP1,BCL10,DNMT3B,ELF3,GNA11,HIST1H3I,HNF1A,HOXB13,KIT,MAP2K4,MEF2B,MLL3,MYC,NUF2,PTPRD,SHQ1,SMAD3,SOCS1,STK40	0.087249929	1.337238568
path_189	CD79B,DNMT3A,ELF3,GPS2,HOXB13,KDM5A,LATS1,NKX3-1,NOTCH1,NTRK1,NTRK2,PAK1,PGR,PIK3C3,PTEN,RAC2,RASA1,RBM10,SMAD3,TCF7L2	0.068387829	1.354092613
path_190	AKT1,ASXL1,CD79B,CDKN2C,CREBBP,DROSHA,ERCC5,FANCA,GLI1,IGF2,INPP4B,NKX2-1,NTRK1,RFWD2,RICTOR,RNF43,SETD2,SMAD3,TCF7L2,TERT	0.114126671	1.319064282
path_191	ANKRD11,ARID1A,CCND2,CCND3,CDK12,CENPA,CUL3,FGF19,FYN,GLI1,HGF,HIST1H3H,PALB2,PARK2,RPS6KA4,RPS6KB2,SH2D1A,SPEN,TAP1,TP53BP1	0.321848063	1.150716445
path_192	AKT3,BRCA1,CDC73,CSF1R,E2F3,ERCC4,ETV1,GATA2,HGF,HIST1H3A,NKX2-1,NOTCH3,PIK3CB,PLCG2,PTPRS,RASA1,RFWD2,SDHA,SMAD3,WT1	0.089152168	1.270946968
path_193	ARID2,CALR,CRLF2,EPHA3,ERCC2,FGF19,HOXB13,IGF1R,MYC,NFE2L2,PIK3C3,PIK3CA,POLD1,POLE,RAD50,SLX4,SPOP,STAT5B,TERT,TP53	0.141700157	1.288172792
path_194	AKT3,CARD11,CTCF,CXCR4,GATA2,KMT2A,KNSTRN,MDM4,MST1,NEGR1,PIK3C3,PNRC1,RNF43,SDHB,SLX4,SOX2,SPOP,SYK,TCF3,TRAF7	0.157861791	1.23103144
path_195	ACVR1,ALK,BCL6,CASP8,CHEK2,DDR2,EIF1AX,ERBB2,FGF19,FGFR3,IFNGR1,INHBA,INPP4B,MCL1,MED12,MYC,NOTCH3,NSD1,PIK3R2,RHOA	0.072732818	1.359764409
path_196	ARID5B,CARD11,CDKN1A,CTCF,EGFR,EIF4E,ERBB2,FBXW7,HIST1H3A,HIST1H3I,HIST3H3,HLA-A,IRS1,KNSTRN,MAX,MLL,PAK1,PIK3R1,PTCH1,SMAD3	0.481434015	1.13674208
path_197	ASXL2,BAP1,BTK,CARM1,CDC42,CREBBP,DICER1,ERCC2,FAM46C,GLI1,IL7R,IRS1,KMT2D,MLL,MST1R,NFKBIA,PARP1,PIK3R1,SH2D1A,TET1	0.339100017	1.144537162

path_198	AXIN2,BLM,CDK4,EPHB1,ERCC2,FGF3,FOXP1,GNAQ,GNAS,INHBA,KMT2D,MYCL1,NKX2-1,PIK3C3,PIK3R2,PIM1,RAD50,RUNX1,SLX4,SUFU	0.192886147	1.201282909
path_199	ABL1,BRD4,CARD11,CDKN1A,CHEK2,DNMT3B,ERBB2,FGFR2,HIST1H3G,INSR,IRS2,JAK1,MDM4,PIK3CG,PMS2,SH2D1A,SMAD2,SOS1,TBX3,TCF3	0.912196483	0.982032516
path_200	AKT2,ALK,BTK,CDH1,CDK6,ERBB2,ERCC4,HOXB13,INHA,IRS2,KMT2D,MAP3K1,MEF2B,MET,MLL2,PIK3C2G,POLE,PPM1D,RB1,RYBP	0.473728243	1.118108581
path_201	ARAF,AXIN1,CALR,FANCA,FGF3,FGFR1,FOXO1,FOXP1,HIST1H3E,IKBKE,JAK1,KEAP1,NKX2-1,NOTCH4,PMS1,RAD51C,RAF1,STAT3,TAP1,TNFAIP3	0.382674842	0.869480592
path_202	APC,ATM,BAP1,BCL2,BMPR1A,CD274,CDK6,CSF1R,GRIN2A,GSK3B,HIST1H3C,INSR,JAK2,KEAP1,MCL1,MDM4,MYC,PIK3C2G,SRSF2,WT1	0.100499588	0.782897848
path_203	ATR,AURKA,AXL,CD274,EGFR,EPHA3,EPHA7,ETV1,FOXL2,HIST1H3J,KDM5A,KEAP1,MALT1,MAP3K1,PPP6C,SDHA,SH2B3,SOX17,SYK,TAP2	0.280322286	1.169552502
path_204	AKT1,BCL2L11,CARD11,CD276,CDK12,CDK8,CHEK1,CRLF2,DIS3,FH,FLT4,FOXP1,GLI1,KDM6A,MST1R,MYOD1,NOTCH2,PAX5,PIK3CB,RAD54L	0.356668002	1.138837338
path_205	ARID5B,AXIN1,AXL,BLM,CCND3,EIF1AX,ESR1,IGF2,INHA,KDM5A,KRAS,MGA,NCOA3,NPM1,PAK7,PNRC1,RAD51D,RAF1,SMARCB1,TCF7L2	0.158327768	1.231819709
path_206	AR,DROSHA,EP300,EPHA5,FGFR3,GNA11,GNAS,HIST1H3J,ICOSLG,IL10,INHA,KDM5C,KMT2A,LATS1,PBRM1,PIK3CB,PIK3CD,PIM1,PREX2,SDHB	0.051651825	1.455040393
path_207	BTK,CARD11,CXCR4,DNMT1,ERBB4,FANCC,FGFR2,FH,FYN,HGF,HIST1H3B,INHA,JAK2,MAP3K1,MEN1,MSH3,MTOR,PLK2,PMS2,RFWD2	0.070509523	1.397837983
path_208	ALK,APC,ARID2,B2M,CDK12,CHEK2,CREBBP,CTCF,CXCR4,KNSTRN,KRAS,MUTYH,PHOX2B,PRDM1,PREX2,PTPRS,SMARCA4,SMARCD1,SPOP,TCF7L2	0.243210114	1.180600827
path_209	ASXL1,BBC3,BRAF,CCNE1,CDKN2A,DAXX,DNMT3A,ERBB3,FOXP1,INPP4B,MET,MST1R,MYC,NF2,NSD1,PDGFRB,PIK3R1,RARA,STAT5B,TGFBR1	0.456847414	1.130285527
path_210	ASXL2,AXIN1,AXIN2,DNMT3B,ERCC4,FGFR2,HNF1A,INPP4A,MAX,MRE11A,MUTYH,NF1,NUF2,NUP93,PTPN11,RPS6KA4,RPS6KB2,SDHB,SMAD2,TET1	0.263503607	1.234758403
path_211	CCND2,CDK12,CDKN2C,CHEK2,CRLF2,E2F3,EPHA5,KIT,MED12,MEF2B,MLL3,MYC,MYOD1,NF2,PARK2,PARP1,SLX4,SPEN,TAP1,TCF7L2	0.069372493	1.350124557

path_212	ARID1B,BRD4,CASP8,EGFR,ERBB4,ERCC5,FGF19,GATA1,HIST1H3E,KDR,NF1,PDGFRB,PMAIP1,PRDM1,RAD51D,RBM10,SMARCB1,TEK,TET2,TGFBR1	0.084345559	1.291355096
path_213	BCOR,CBL,CD276,FGFR4,FOXA1,HIST1H1C,IGF1R,IL7R,JAK2,MAX,MDM4,MLL2,PIK3C3,PLK2,POLD1,RBM10,RHEB,SH2B3,TERT,TET2	0.43473975	1.128767229
path_214	CDKN1B,CTLA4,DDR2,GSK3B,HIST1H3E,IKZF1,KDM6A,MAP2K4,MSH6,MYCN,PAK7,RB1,REL,RFWD2,SF3B1,SOS1,SOX9,STK11,TET1,TRAF7	0.092121887	1.300414702
path_215	CARM1,CD79B,DIS3,EPHA7,ERCC4,FAM175A,H3F3C,HIST1H3D,IGF2,IKBKE,IKZF1,IRF4,MYCN,NFKBIA,NKX2-1,NPM1,RAD51D,SOS1,SPOP,TP63	0.606751476	1.076091811
path_216	ARID1A,CCND2,CXCR4,FGFR1,FOXO1,HIST1H3J,MDC1,MTOR,NRAS,PAK7,PPP6C,PTPRS,RAD51,RHOA,SMARCA4,SOX17,SOX2,SYK,TET1,TGFBR1	0.225481934	1.206634288
path_217	APC,EGFR,ERBB2,FGFR1,GLI1,KDR,KLF4,MEF2B,MITF,MLL3,MSH2,MSH6,MYCN,NOTCH2,PARP1,PIK3CD,PIK3R2,POLD1,PRDM1,SOCS1	0.785614674	0.958947486
path_218	AKT1,ALK,ATR,BBC3,CARM1,CASP8,CDC42,CENPA,DNMT1,ERCC5,FBXW7,HIST1H1C,JAK1,MLL3,MYCN,NF2,NOTCH1,PIK3R1,SDHB,SDHC	0.052557463	1.417247696
path_219	AKT3,ARID1A,ARID5B,CCNE1,CDKN1B,CHEK1,CIC,CRLF2,CSF3R,EGFR,FAM46C,FANCC,FH,GLI1,HGF,HIST1H3E,LATS2,NRAS,PDGFRA,SOCS1	0.500663041	1.118259452
path_220	ACVR1,ARID5B,BCL2L11,CALR,CDC42,CEBPA,DAXX,DNMT1,EZH2,FAM175A,FGFR4,HIST1H3A,IL7R,JAK3,JUN,NCOA3,PDCD1,PHOX2B,SRSF2,STK11	0.438410902	1.157495132
path_221	ANKRD11,ATR,AXIN1,BCL2L11,CRLF2,ESR1,KDM5C,MALT1,MAP2K2,MDC1,MSH6,NTRK1,PARK2,PIK3CD,POLE,TAP1,TBX3,TGFBR1,TOP1,ZFHX3	0.060363083	1.393693163
path_222	AKT3,ANKRD11,DDR2,DNMT3B,ELF3,ESR1,IKZF1,JUN,MLL,NBN,PDGFRB,PHOX2B,PTCH1,RAD51,RB1,RPS6KB2,RYBP,SPEN,SUFU,TBX3	0.391198506	1.173900286
path_223	ALK,AXIN2,CDC73,CDK8,CIC,DAXX,FGF4,FH,FOXP1,GATA2,GPS2,IKZF1,NOTCH2,PIK3C3,PMS1,PMS2,SETD2,SMAD2,SOX2,STAT5B	0.629054508	1.090369645
path_224	AKT1,ALOX12B,CDH1,CSF3R,DIS3,ERRFI1,FYN,IDH1,IRF4,KMT2D,LATS1,MCL1,MITF,MSH2,MUTYH,NFKBIA,PIK3R3,PTEN,PTPRD,TET2	0.069301405	1.291438243
path_225	AXIN1,BAP1,BCL2L11,BCL6,CCND3,CHEK1,EPHA5,ERCC2,ETV6,ICOSLG,KRAS,MALT1,MGA,MSH6,PDGFRA,PLCG2,ROS1,SLX4,STAT3,TAP1	0.308475207	1.162614307
path_226	CIC,CXCR4,EGFR,EIF1AX,EIF4A2,EP300,ERBB2,FOXA1,FOXL2,GNA11,H3F3C,KDM5C,KNSTRN,MAP3K1,NOTCH3,PBRM1,PPM1D,PTPRT,RICTOR,SMARCD1	0.109885396	1.297968617

path_227	ARID1B,CBL,CDC73,CXCR4,ELF3,FGF3,FGFR1,GRIN2A,HIST1H3G,JAK3,LATS1,MET,MRE11A,MYCN,NOTCH3,NSD1,PIK3CA,ROS1,SOS1,TAP1	0.105973647	1.337815909
path_228	ARAF,BTK,FANCC,FGF19,FGFR4,HIST1H3E,HLA-A,LATS2,NCOR1,PALB2,PIK3C3,PMAIP1,PREX2,RAD50,RAD51,SHQ1,STK11,TAP2,TGFBR1,TNFAIP3	0.734577224	0.946064847
path_229	ARID1A,ATR,CTLA4,DROSHA,EP300,EPHA3,FAT1,INSRR,MPL,NFKBIA,PAK1,RHEB,RPS6KA4,SH2B3,SMARCD1,SPEN,SRSF2,STAT5B,STK40,TRAF2	0.11627124	1.304405136
path_230	APC,ARID1B,BBC3,CARM1,CDK6,FGFR3,INHBA,JAK1,MED12,MET,NFE2L2,NOTCH1,NPM1,PARP1,PAX5,PIM1,RAD50,RPS6KA4,SLX4,STAT3	0.188234593	1.274866448
path_231	ALK,ARID1A,AURKA,CDKN1A,ELF3,ERBB3,FANCA,FLT3,FOXL2,HLA-A,IFNGR1,IRS2,MLL,MYCL1,NF2,PALB2,REL,STK40,TERT,TMPRSS2	0.315865349	1.180795595
path_232	ASXL1,ATR,CCND2,CDC42,CRLF2,CTNNB1,DNMT3A,EP300,FYN,GNA11,KNSTRN,MET,NRAS,PAK7,PAX5,SDHB,SHQ1,SLX4,SOX2,TAP1	0.303377247	0.842324517
path_233	APC,ATRX,CIC,CRLF2,CUL3,EED,FAM46C,FBXW7,IRF4,KDR,MPL,MTOR,MUTYH,MYCL1,NCOA3,NOTCH3,NUP93,PIK3R1,RHEB,SOX9	0.066576493	1.401059823
path_234	ARID5B,ATR,CD276,CDKN2A,CDKN2C,CRLF2,CUL3,FAT1,HGF,HIST1H1C,IRF4,MAP2K1,PAK7,POLD1,PTPRT,RAD51C,RBM10,RNF43,SOX2,SPEN	0.109254068	1.260828169
path_235	AKT2,CBL,CDH1,CENPA,DDR2,DICER1,HIST1H3E,HIST1H3I,HOXB13,IKBKE,INPP4A,IRS2,PAK1,PBRM1,PIK3R3,POLD1,RASA1,RBM10,SDHAF2,TEK	0.149499804	1.337483892
path_236	ALK,BCL2L11,BRAF,CIC,DIS3,FBXW7,HIST1H3B,HIST1H3I,INHBA,KDM6A,KRAS,LATS1,PALB2,PIK3R2,PMS2,RB1,SF3B1,SMAD3,SMARCA4,TCF7L2	0.365314927	1.13603959
path_237	BAP1,CSF3R,DOT1L,ETV1,FGFR3,FUBP1,GATA2,GSK3B,KDM5A,KLF4,MCL1,MITF,NRAS,PAK1,PDGFRB,PTPRS,RAD50,RAD54L,SMARCB1,SPEN	0.191440389	1.201725152
path_238	BCL6,CCND1,CDC73,CDKN2C,CSF1R,DNMT3A,E2F3,FGF19,FUBP1,GPS2,HIST1H3B,HIST1H3I,IFNGR1,IL7R,MEF2B,MYCL1,NTRK1,PLK2,SOX9,SYK	0.818979124	1.034060823
path_239	ACVR1,APC,BCL6,CD276,DOT1L,EIF1AX,GATA1,IRS2,KMT2A,MCL1,MED12,MRE11A,MST1,PDCD1,PHOX2B,PIK3CA,PIK3R3,RAD50,SMAD3,TAP2	0.298481914	1.157890449
path_240	BCL2,CARM1,CDKN2C,CHEK1,DAXX,FANCC,FBXW7,FGFR4,HGF,MAP3K13,NF1,NPM1,PARP1,PIK3CD,PPP6C,PRDM1,RAC2,RHEB,RPS6KA4,SMAD2	0.241083769	1.181657362

path_241	AKT3,AXL,BBC3,CCNE1,CDKN2A,CTCF,ERG,FGFR4,GLI1,GNA11,IDH1,INSRR,JAK1,KDR,NEGR1,PNRC1,RBM10,RHOA,STK40,TAP2	0.2548252	1.231193425
path_242	CBL,CD274,CSF1R,CTCF,CTNNB1,DIS3,EPHA5,GNA11,HIST1H3D,HIST1H3H,HLA-A,IKBKE,INPP4A,MSH6,NF1,PGR,PPM1D,RET,RPTOR,SETD2	0.282479502	1.192077333
path_243	ARID1A,BARD1,CSF3R,EIF4E,FANCA,FGFR4,GATA2,IFNGR1,IGF2,MAP2K2,MCL1,MDC1,MSH2,MYC,NRAS,PBRM1,PIK3CA,PIK3R2,SH2B3,SHQ1	0.607382425	0.923627381
path_244	ABL1,BLM,BRIP1,CBL,CDH1,CRLF2,CSF1R,ERBB3,ETV6,FGF4,H3F3C,IGF2,KMT2A,MEF2B,MSH3,PARP1,PDGFRA,RBM10,RFWD2,STAG2	0.838411839	1.035567647
path_245	CDK12,CXCR4,DNAJB1,ESR1,GATA1,GSK3B,HIST1H3I,IGF2,JAK1,MEF2B,MST1,MUTYH,NKX3-1,NTRK1,PARK2,PMAIP1,PMS2,RAD51B,SPEN,TRAF2	0.214385598	1.194745409
path_246	ARID1A,CARD11,CBL,CHEK1,CTNNB1,ELF3,GATA1,IDH1,IGF2,NEGR1,NFKBIA,NKX3-1,PIK3C3,PIK3R2,PLCG2,PRDM1,SPOP,TBX3,TCF3,TP53	0.168956723	1.215668328
path_247	AMER1,ASXL2,ATR,BRAF,CDKN1B,ERBB4,FH,IDH1,IL7R,MGA,NBN,NOTCH4,NUP93,POLD1,POLE,RHEB,RNF43,SMAD4,SOX2,SYK	0.291864997	1.185347411
path_248	ARID2,BARD1,CDKN1A,DAXX,DROSHA,EIF4E,EP300,FBXW7,HIST1H3I,HLA-A,IGF1R,IL7R,IRS1,MITF,NTRK3,PAK7,RAC2,SETD2,SMAD4,SMARCD1	0.456144535	1.130582228
path_249	AKT3,APC,BCL2L1,CCNE1,CDK8,CSF1R,CTLA4,DROSHA,EIF1AX,GRIN2A,HIST1H3D,HNF1A,ICOSLG,IGF1R,RAD51B,SMAD3,SMO,TCF7L2,TERT,TRAF2	0.551890752	1.088483082
path_250	AMER1,CCND2,CDKN1B,DNAJB1,ETV1,FANCA,FGF3,FGFR1,GPS2,GRIN2A,H3F3C,HIST1H1C,HIST1H3G,PAX5,PDCD1,PIK3C2G,PNRC1,RET,SOCS1,TAP2	0.077181991	1.280556508
path_251	ARAF,BARD1,CD79B,CDKN1A,DOT1L,FGF4,FOXO1,GATA3,HIST1H1C,JAK1,KDM5C,KNSTRN,MCL1,MST1R,MYOD1,PARP1,PDGFRA,PIK3CD,PRDM1,RECQL4	0.775417316	0.960492621
path_252	BRAF,CBL,CDH1,FAM46C,FGFR2,FOXA1,GSK3B,HIST1H3A,HIST1H3J,IL10,IRF4,IRS1,MEF2B,NEGR1,NF1,PDCD1,PRDM1,PTEN,RAD51B,RHOA	0.332348216	1.146834098
path_253	ARAF,AXIN2,CREBBP,EED,ERBB3,EZH2,GATA1,HIST1H3E,JAK3,KDM6A,MST1,NFKBIA,PIK3CA,PTEN,RB1,SDHB,SOCS1,TERT,TNFAIP3,TP53BP1	0.191957059	1.27584376
path_254	ARID2,CDK12,ESR1,ETV6,GNA11,HIST1H3D,KRAS,MALT1,MAP2K2,MAP3K13,MLL2,MYCL1,NFKBIA,NUF2,POLD1,POLE,RHOA,RTEL1,RYBP,SMAD4	0.37452469	0.880274826

path_255	ARID5B,BAP1,BCL2L1,BRCA1,CBL,CD274,CSF1R,EGFR,ERBB4,EZH2,FOXO1,GNAS,HIST1H3D,IRS1,MSH6,SMAD3,STAG2,TAP2,TERT,TRAF2	0.24646582	0.812921744
path_256	ABL1,ACVR1,BCL6,BRAF,CSF1R,CUL3,DNMT3A,ERBB3,FBXW7,FLT3,HIST1H3H,INHBA,KMT2D,MAP3K1,MAP3K13,MLL,NBN,PAK7,PGR,PIK3C2G	0.201184709	1.23772771
path_257	ARID1A,BCL6,E2F3,EPHA5,FOXO1,GNAS,HIST1H3D,ICOSLG,INSRR,KDR,MRE11A,MST1,PIK3CB,PLCG2,RASA1,RNF43,RYBP,SHQ1,SMAD3,SUFU	0.471887407	1.128703296
path_258	ANKRD11,AR,B2M,CCND2,CDKN2A,CTNNB1,FOXA1,HIST1H1C,IKZF1,INPP4A,JUN,KDM6A,MED12,MST1,MST1R,NEGR1,PLK2,PPM1D,RET,RICTOR	0.257532568	1.211316211
path_259	AKT2,BRCA2,BRIP1,CIC,DNMT3A,DNMT3B,GATA2,HIST1H3E,IDH1,IRF4,JAK2,KMT2D,MEN1,MSH3,NF1,NOTCH3,NTRK2,PIK3R3,PMS2,SMARCA4	0.065740894	1.323465892
path_260	ASXL2,CBL,CCND2,CHEK2,FGFR3,FLT4,FOXP1,FYN,MDC1,MST1,NOTCH4,PDGFRB,PIK3R3,PIM1,PMAIP1,PTEN,PTPRT,SHQ1,STK11,TAP1	0.830439777	1.031204161
path_261	ABL1,CDKN1A,E2F3,EP300,FLT4,GNAQ,INHBA,KLF4,MST1R,MTOR,NCOA3,PDGFRA,PIK3CA,RAD51D,RHOA,RPTOR,SH2D1A,SOCS1,TCF3,TMPRSS2	0.325977115	1.148730543
path_262	AKT1,ATRX,AURKA,CASP8,CD79B,FAM46C,HIST1H3I,IKBKE,MDM2,MST1R,MYOD1,NEGR1,NRAS,NUP93,PIK3CB,PTPRD,RAD51D,SMAD2,SMARCB1,TEK	0.90675895	1.019208066
path_263	CD274,CDK6,CTCF,CXCR4,DNMT3A,DNMT3B,DROSHA,EPHB1,FGFR4,HIST1H3C,JAK1,JAK2,KIT,PBRM1,PIK3R3,PMS2,PREX2,PTPRT,SMAD4,STK11	0.517756135	1.102605479
path_264	ARID1B,BARD1,CARD11,CCND1,CD274,CREBBP,CUL3,EIF4E,ERCC5,EZH2,GATA3,GRIN2A,GSK3B,NF2,NUF2,RAD51,RARA,RB1,REL,TAP1	0.233254539	1.240528241
path_265	ALOX12B,ASXL1,BTK,FGF19,HIST1H3B,HIST1H3H,HLA-A,IRS1,JAK2,MAX,NEGR1,NSD1,PARP1,PIK3R2,RAF1,RBM10,RHEB,SRSF2,STAT5B,STK11	0.659438381	1.068374865
path_266	AKT2,ASXL1,BRCA1,CCND2,CTNNB1,DNAJB1,ERBB2,ESR1,GLI1,INSR,KDR,NF1,PIK3C2G,PIK3CB,PRDM1,RAF1,RFWD2,RICTOR,RYBP,YAP1	0.905185036	1.019389819
path_267	AKT1,AXL,CEBPA,DNMT1,DNMT3B,ERBB3,FGFR2,FGFR3,GLI1,HIST1H3B,INSR,PARP1,PDGFRB,PIK3CB,PLK2,RBM10,RET,RPS6KA4,SOCS1,TNFAIP3	0.437573223	1.169768999
path_268	APC,ASXL2,ATM,BRCA1,CDK8,ERBB4,FGF3,IRF4,KDM5C,NCOR1,NSD1,PDCD1,PHOX2B,PIM1,PPM1D,RAD54L,RBM10,ROS1,SOX17,TCF7L2	0.922108916	0.985761416

path_269	ABL1,ARAF,BRIP1,CDC73,CHEK1,ERG,FGFR3,GATA1,IDH1,INHA,KNSTRN,MDM2,MEN1,NBN,NF2,NUF2,RPS6KB2,SOS1,STAG2,TAP1	0.089138004	1.272810459
path_270	AKT1,BAP1,BLM,BMPR1A,CARD11,CD79B,EED,EZH2,GATA1,HGF,HIST3H3,KMT2A,KMT2D,KNSTRN,MALT1,PBRM1,RUNX1,SMAD3,SMAD4,SRC	0.902563691	1.017437153
path_271	ALK,CARD11,EP300,FGF4,ICOSLG,INSRR,KDM6A,MEN1,MGA,MLL2,NFE2L2,PAK7,PDGFRB,PMAIP1,PNRC1,RAD51B,RAD54L,RARA,RBM10,TSHR	0.819911322	1.034450886
path_272	AMER1,ARAF,ARID5B,BCL2L1,BRD4,CDC73,DIS3,DNMT3B,ERCC4,KDR,KLF4,MAP2K4,MLL2,MST1R,NPM1,NRAS,PDCD1,RET,SOS1,STK40	0.22810292	1.185545615
path_273	ALOX12B,ARID1B,CREBBP,CTCF,DNMT1,EIF1AX,EIF4A2,FGF19,HIST1H3C,MAP3K1,NCOA3,NF2,NFKBIA,PPP2R1A,PTCH1,RAD51D,RPS6KA4,SH2D1A,STAT5B,TGFBR2	0.191086529	1.306948367
path_274	ARID1B,BRIP1,CD274,CDK12,CIC,CTCF,FLT4,HNF1A,KDM5C,KIT,LATS2,MLL2,PIK3CG,RAD21,RAD51C,RASA1,RTEL1,SOX9,TNFAIP3,XPO1	0.728991317	1.059678416
path_275	ARID2,ATM,BCL2,CARD11,CTNNB1,ERCC2,EZH2,GNA11,GPS2,IKBKE,MST1R,NEGR1,NTRK2,PAK7,PIK3R2,RPS6KB2,SMAD2,TCF3,TRAF2,U2AF1	0.965299237	1.007305235
path_276	ARAF,ARID2,CBL,CCND3,EIF4A2,ETV1,GATA2,GSK3B,MLL,MYOD1,NCOR1,PAK1,PDCD1,PMAIP1,PNRC1,POLE,PPM1D,PTCH1,RAF1,SOS1	0.107201751	1.340395229
path_277	ARID2,AXIN2,BCL2L1,DNMT1,FGF19,GNAS,HGF,HIST1H3B,HIST3H3,NTRK3,PAK7,PIK3CB,PIK3CD,PREX2,RAD21,RAD51D,RHEB,SMARCD1,TAP1,TGFBR2	0.862284185	1.035270223
path_278	BCOR,CARD11,CD274,CDK12,DAXX,FANCA,FOXA1,GNAS,INHBA,IRF4,KDM5A,MPL,NBN,PIK3CB,PRDM1,RARA,REL,SF3B1,SMO,STAT3	0.081692961	1.280433015
path_279	ABL1,ALK,BLM,CCND1,DNMT1,DOT1L,EPHB1,FANCA,GATA2,GNA11,GNAQ,JAK3,KMT2D,MDM4,PPP2R1A,RAD21,RARA,RYBP,TAP1,TET1	0.458054081	1.111507412
path_280	ANKRD11,ASXL2,CCND3,CIC,DAXX,EZH2,FH,GATA1,IGF1R,NFE2L2,NKX3-1,NOTCH1,NOTCH2,NPM1,POLD1,RAD51B,SMAD4,SOCS1,SRSF2,TNFAIP3	0.105556997	1.385934185
path_281	AKT3,ALK,ANKRD11,ASXL2,BRIP1,CCNE1,CDKN1A,CSF3R,ERBB4,FLT3,HLA-A,MEF2B,NOTCH4,RHOA,RNF43,RPS6KB2,SDHB,SOX9,SYK,TCF7L2	0.251329939	1.230580652
path_282	APC,ARID5B,AURKA,CDH1,CDKN1B,CIC,EPHA5,ERG,FGFR1,FOXO1,HIST1H3E,PMAIP1,PTEN,RAF1,RFWD2,ROS1,SDHB,STAT5B,SYK,TCF3	0.197099732	1.275592505

path_283	APC,BCL6,BRCA1,CCND1,CCND3,CD276,CTLA4,DNAJB1,DOT1L,FGF3,FLT3,GRIN2A,HIST3H3,IKZF1,MAP2K4,NOTCH1,NOTCH3,PHOX2B,RPS6KB2,SUFU	0.140986654	1.311536128
path_284	AKT1,ARID1A,ATR,BMPR1A,CARM1,CASP8,CDKN2A,CENPA,CTLA4,ERRFI1,FGFR2,IRF4,MALT1,MDM2,MLL,MST1,PNRC1,RB1,SLX4,SMARCA4	0.090987715	1.319622214
path_285	ALK,CCNE1,CD79B,ERG,FANCA,GNA11,HIST1H3I,HOXB13,IFNGR1,IRF4,KDM5C,KDR,MALT1,MAP2K4,MAX,MRE11A,NPM1,PHOX2B,RYBP,SDHC	0.140042103	1.381672336
path_286	CDK4,CDKN2A,CHEK1,CHEK2,DROSHA,EPHA3,FGFR1,FLT4,GRIN2A,HIST1H3B,KLF4,MAP2K2,MLL2,MLL3,RAD21,RET,SLX4,SOCS1,SPOP,STK40	0.492561277	1.112265062
path_287	BBC3,CD276,DDR2,ERCC2,FGF3,FLT4,FOXA1,FUBP1,HIST1H3H,MTOR,MUTYH,MYOD1,NCOR1,NKX2-1,RHEB,SF3B1,SPOP,SRC,TGFBR1,TP53	0.061091267	1.30735823
path_288	CDK6,EZH2,FYN,GRIN2A,HIST1H3B,IKBKE,MAP2K1,MAP2K4,MLL,MLL3,PDGFRB,PHOX2B,PIK3R2,RUNX1,SDHA,SPOP,SRSF2,STAT5B,STK40,TEK	0.514103382	1.097151452
path_289	BAP1,BCL6,CCNE1,CDKN1B,CHEK2,EIF1AX,EIF4E,ERCC4,EZH2,FANCA,FGF4,FOXP1,GNA11,KLF4,NF1,PALB2,SDHB,SHQ1,SMO,TCF3	0.594779163	1.077379006
path_290	ALOX12B,ARID1A,ARID2,BBC3,CARM1,DIS3,DNMT1,FAT1,HIST1H3D,HIST1H3E,ICOSLG,KIT,MAX,MSH3,MST1,PIK3CD,PMS1,RECQL4,TET2,TP53	0.157374757	1.268137673
path_291	AMER1,AXL,CDKN1B,CTLA4,ERBB3,ERBB4,FGF19,JAK1,JAK3,LATS2,MEF2B,MLL3,PALB2,PARK2,PARP1,RAF1,SMAD2,SMARCD1,SOX17,TGFBR1	0.230047078	1.254477142
path_292	ALOX12B,ARAF,BCL2L11,BLM,BRD4,CD276,CUL3,DDR2,FGFR3,GATA1,GSK3B,HIST1H3B,KEAP1,LATS1,MAP3K13,PIK3C3,PNRC1,PREX2,SETD2,STK40	0.428956852	1.137250796
path_293	AXIN2,EIF4A2,HIST1H3D,JAK2,KLF4,KRAS,MALT1,NEGR1,NF1,NFKBIA,NTRK2,PIK3C2G,PTCH1,REL,RFWD2,STAG2,TBX3,TP53,TRAF7,YAP1	0.311777887	1.158422467
path_294	BCL2L1,BCOR,CDKN2A,CDKN2C,DROSHA,GRIN2A,IFNGR1,IL7R,JAK3,MALT1,MAP3K1,MAP3K13,NCOA3,NTRK3,PARP1,PPM1D,PRDM1,SOCS1,SPEN,STAT5B	0.323818956	1.190842849
path_295	AKT1,BCOR,CARM1,CHEK2,FBXW7,GATA3,HIST1H3I,IL7R,INHBA,JAK3,MCL1,NTRK1,NUP93,PARK2,PHOX2B,POLE,RAD54L,RUNX1,SH2B3,SMAD2	0.674482887	1.061433936
path_296	ANKRD11,BBC3,CCNE1,CDH1,CDK6,CTNNB1,DIS3,ERBB4,HIST1H3C,HIST1H3D,IRS1,JAK1,PARP1,PAX5,PBRM1,RAD54L,ROS1,RUNX1,SLX4,SOX2	0.089889632	1.414743263

path_297	B2M,BCL2,CARM1,CASP8,CCND1,CD79B,CDK8,CDKN2C,GATA3,HIST1H3D,HNF1A,JAK3,KNSTRN,MEF2B,MLL3,PARP1,PDCD1,PHOX2B,SMAD3,SOX2	0.201490902	1.202197073
path_298	BCL2L1,BRAF,CDK12,CYSLTR2,DNMT3B,DROSHA,FOXO1,HIST3H3,IKBKE,IRS2,JAK1,MAP2K4,MED12,NEGR1,PAK1,RARA,RTEL1,STAG2,TET1,TSC2	0.908688073	0.983870138
path_299	ARID2,AXIN1,BCL2L1,BTK,CD79B,CDC42,CDKN1B,CTLA4,ERBB2,EZH2,FAM46C,FGFR2,MSH2,NF2,NUF2,PDCD1,PGR,RB1,REL,RYBP	0.557527982	1.110686624
path_300	ANKRD11,BRCA1,CCND2,CDK12,CRLF2,EPHA5,FGFR4,GATA3,HIST1H3D,MET,MSH6,NFE2L2,NRAS,NTRK3,PGR,PIK3CD,RAD50,SMARCA4,SMO,SPOP	0.138356183	1.25111015
path_301	AXL,CCND1,CCND3,CDK12,DNMT1,GATA1,GATA3,HIST1H3H,HOXB13,IKBKE,MPL,NRAS,PAK1,PIK3CA,RAF1,RET,SMAD3,SMARCA4,STAG2,TCF7L2	0.70054877	1.070581062
path_302	ALOX12B,ARID1B,BCL2,CASP8,CXCR4,DIS3,DROSHA,ERG,GRIN2A,HOXB13,ICOSLG,IDH1,INPP4B,JAK3,MUTYH,NF2,PHOX2B,SDHB,SH2D1A,SMARCA4	0.128116233	1.285804676
path_303	BCL2,BRD4,CD276,CDKN1A,CTCF,ERBB3,ERRFI1,ETV6,FGFR3,FOXO1,HIST1H3C,HOXB13,KRAS,MYCN,PALB2,PPP6C,RECQL4,SDHA,SMARCA4,SMARCD1	0.12825326	1.259475979
path_304	AKT1,CCND1,CCNE1,CDC42,CDKN1A,ERBB3,FH,FOXL2,HIST1H3G,JAK1,KIT,MDC1,MEF2B,NFE2L2,NOTCH1,PIK3C2G,RAC2,RBM10,SOS1,TCF7L2	0.167086554	1.254683232
path_305	AKT2,BCL2L1,BCL6,CARD11,CDKN2A,ERCC4,FAM46C,FBXW7,GATA1,KEAP1,KMT2A,MED12,MEN1,NEGR1,NOTCH1,PAX5,PIK3CD,REL,RTEL1,SMAD2	0.078122389	1.305387982
path_306	CDK8,CHEK1,EIF1AX,ERCC4,GATA1,MEN1,MLL2,MPL,NCOA3,NEGR1,NOTCH2,NUF2,NUP93,PIK3R3,PRDM1,RAD21,ROS1,RPS6KA4,SUFU,SYK	0.592181158	1.079863271
path_307	B2M,CENPA,DROSHA,EIF4E,ETV6,H3F3C,HNF1A,ICOSLG,IFNGR1,IKZF1,INSRR,JUN,KDM5A,KRAS,MSH2,MUTYH,NOTCH3,PMS2,RASA1,RHEB	0.159640884	1.264464327
path_308	BRCA1,CTNNB1,DDR2,FANCC,FGF3,FOXL2,H3F3C,IKZF1,JUN,KIT,KMT2A,MALT1,MAP2K4,NFKBIA,PDCD1,PIK3C3,RAF1,RBM10,ROS1,SDHB	0.45449908	1.142952287
path_309	BCL2L11,CDH1,CDK6,CHEK2,CUL3,DOT1L,FGF3,FOXO1,GATA1,GNAS,HLA-A,IGF1R,IRS1,IRS2,MLL3,NFKBIA,PALB2,PAX5,PTCH1,SETD2	0.287729493	1.192787135
path_310	AMER1,AXIN1,DNMT3B,DOT1L,FAT1,FGFR2,FOXL2,FOXO1,GNA11,IFNGR1,INHBA,KLF4,NOTCH2,PTPN11,PTPRS,RAD51C,SDHB,STAG2,STAT3,TBX3	0.059713021	1.393378232

path_311	BARD1,CCND3,CCNE1,CD79B,CDK4,CDK6,EIF1AX,HIST1H3H,MAP3K1,MDM4,MRE11A,MYC,NFKBIA,NOTCH1,PIK3R2,RAF1,RECQL4,RPS6KB2,SETD2,SUFU	0.276373421	1.167084072
path_312	ARAF,CARD11,CBL,CD276,CDH1,CXCR4,FGFR2,HIST3H3,MST1R,MYCN,NEGR1,PIK3R2,POLD1,RFWD2,RICTOR,RPTOR,SH2B3,SMARCD1,SOX9,TP63	0.346973929	1.142458647
path_313	ALOX12B,CALR,CD79B,CDK6,CENPA,CYSLTR2,DOT1L,ERRFI1,FGF3,FLT1,HIST1H3B,HIST1H3D,HLA-A,IRS2,MDM2,MSH2,PDGFRB,PLK2,RAD54L,SMARCB1	0.585225876	1.080780864
path_314	AKT2,ARID5B,ASXL1,BMPR1A,CENPA,DNMT3A,EIF4A2,ELF3,FOXL2,HLA-A,INHBA,JAK3,LATS2,NTRK2,PIK3CA,PPP2R1A,RAD50,RAD51D,RPTOR,RYBP	0.474220103	1.136952646
path_315	BCL10,BRAF,CD79B,CEBPA,CTCF,CTNNB1,FGF3,FGFR4,GLI1,HIST1H3I,HLA-A,INPP4B,KRAS,LATS2,MGA,PIK3CA,PTPRT,RB1,SMAD3,SOX17	0.295134508	1.187957681
path_316	ABL1,AKT3,ARID5B,AURKA,AXIN2,CASP8,CDKN1B,CEBPA,EGFR,FOXP1,HLA-A,MDC1,MLL2,MLL3,MYCN,POLD1,RAF1,RPS6KB2,SMAD4,SOX2	0.600665202	1.082932471
path_317	ATM,CSF1R,CUL3,FGFR1,FLT3,FOXL2,HIST1H1C,LATS1,MAP2K4,MLL,MSH6,NF2,NFKBIA,NOTCH1,PIK3R2,PTPRS,SF3B1,SH2D1A,SHQ1,SLX4	0.341351923	1.168071526
path_318	B2M,BCL2,BCL6,CDK12,CTNNB1,DROSHA,EGFR,GNAS,HIST1H3A,HIST1H3C,INPP4A,MET,MLL,NOTCH3,NUP93,PDGFRA,PTEN,RPS6KB2,SPEN,SRC	0.315706294	1.183639318
path_319	AMER1,ATR,CASP8,CD276,CTCF,MLL2,NOTCH4,NTRK1,NUP93,PPP2R1A,RAD21,RARA,RNF43,RPS6KA4,RTEL1,SPOP,TAP1,TAP2,TCF3,TET1	0.616938775	1.093851219
path_320	CARM1,CBL,CCND3,EED,FAM175A,KDR,MAP2K1,MAP2K2,NKX2-1,NTRK1,PDGFRB,PMAIP1,RAD51,RASA1,RBM10,ROS1,SH2D1A,SLX4,TET1,TNFAIP3	0.289267178	1.162217734
path_321	ALOX12B,ANKRD11,APC,AURKA,CARM1,DNMT3A,FANCA,GNAQ,HIST1H3I,MGA,MLL2,MPL,MYC,NOTCH3,PIK3R1,POLE,PTEN,RAD21,RAD51D,RPS6KB2	0.268473113	1.196533351
path_322	ALOX12B,ASXL2,CCND2,E2F3,ELF3,MET,MITF,MUTYH,MYCL1,NCOR1,NFE2L2,PIK3R1,PIK3R2,PMS2,PNRC1,REL,SH2D1A,SPOP,STK40,TOP1	0.620425931	1.072478289
path_323	ARAF,ARID1A,B2M,BRCA1,BRD4,CALR,CTCF,CYSLTR2,DROSHA,EIF1AX,IGF1R,IL10,NF2,PARP1,PDGFRA,PLK2,POLD1,RASA1,RECQL4,SLX4	0.11005008	1.346548309
path_324	ALOX12B,ARID1B,CD274,CD79B,CDC42,CENPA,CSF3R,EIF1AX,HIST1H3A,IL7R,JAK3,MAX,MYCN,NSD1,PARK2,PIK3R1,RAD51D,RET,ROS1,TET2	0.478439837	1.105032329

path_325	AMER1,ANKRD11,AURKA,AXIN1,BCOR,FYN,GLI1,MAP2K1,MAP2K2,MDM2,MRE11A,MYCN,MYOD1,NTRK1,PPP2R1A,RBM10,RICTOR,STAG2,TAP2,TMPRSS2	0.09642777	1.334869665
path_326	BARD1,BMPR1A,CEBPA,CXCR4,ELF3,FAT1,HIST1H3C,IRF4,IRS1,JUN,KEAP1,MST1,MST1R,NKX2-1,NUF2,PAK7,PAX5,PMAIP1,TGFBR1,TP53	0.124096948	1.277359754
path_327	ARID5B,CARM1,CCND1,CDC73,CDK6,CHEK2,CRLF2,CUL3,EPHA5,FGF19,GNA11,IRF4,MLL,NPM1,PIK3R1,PRDM1,RAD50,RAD54L,TCF7L2,TERT	0.272041649	1.168661135
path_328	AKT3,ANKRD11,ETV6,HIST1H3B,HLA-A,IGF1R,INSRR,KDM5A,LATS2,MEN1,MSH2,MTOR,NEGR1,NOTCH2,PIK3CD,RPTOR,SH2B3,SHQ1,TP53,TP53BP1	0.253958025	1.191155439
path_329	CCNE1,FGF3,GATA2,HGF,HIST1H3D,IKZF1,KDM5A,MAP3K13,MAX,MRE11A,MST1R,MYC,PBRM1,PTEN,RAC2,RNF43,RTEL1,RYBP,TGFBR1,U2AF1	0.102246274	1.344947802
path_330	AKT1,ATR,BARD1,BTK,CDK4,CDKN1A,CDKN2C,ERG,FAM175A,FGFR2,IDH1,IKZF1,INHBA,MET,NEGR1,PARP1,SMARCD1,TP53,TP63,TSC1	0.153911607	1.229730666
path_331	ARID1A,B2M,BRCA2,CHEK1,CYSLTR2,DROSHA,EED,FAM46C,MAP2K4,MCL1,MET,MLL,NOTCH4,PARK2,PBRM1,PTPRT,RICTOR,SDHAF2,SDHC,STK40	0.056835146	1.366896452
path_332	AMER1,AXL,DIS3,ERBB4,FANCA,FH,GNAS,IFNGR1,IKZF1,INPP4B,KDM6A,MAP3K13,NEGR1,NKX2-1,NSD1,PIK3CD,PTPRD,REL,SH2D1A,STK11	0.621954049	1.073361873
path_333	APC,ARAF,AXL,CDC42,CDC73,CXCR4,ERBB2,FOXP1,JAK2,KNSTRN,MED12,MLL,NOTCH4,POLD1,PPP6C,RAD51D,RB1,RBM10,RPTOR,TAP2	0.361536826	1.158309502
path_334	CTNNB1,EED,ETV1,FBXW7,FH,FOXO1,IDH1,IGF1R,INHA,IRS2,MAP2K1,MAP3K1,MTOR,PIK3CA,PTEN,RAD50,RET,SETD2,SYK,TEK	0.136386691	1.276593646
path_335	AKT2,ARID1B,BCOR,CTCF,DDR2,DNMT3B,EED,FANCC,FOXL2,GPS2,HIST1H3D,IGF1R,IGF2,MGA,NOTCH2,NTRK3,RARA,SDHC,STAG2,STAT3	0.174004776	1.264081278
path_336	ARID5B,CALR,CENPA,CUL3,DNAJB1,DNMT3B,HIST1H3I,IFNGR1,KDM5A,KMT2D,MDM2,NFE2L2,NPM1,NUF2,RICTOR,SH2B3,SOX9,SRC,TP63,TRAF2	0.431642505	1.121673929
path_337	AURKA,BLM,BTK,CDKN1B,CDKN2C,CXCR4,HIST1H3C,HIST1H3J,ICOSLG,JAK2,KDM5A,MALT1,MAP2K2,NF2,PIK3CB,POLE,PTEN,PTPRD,RAD54L,REL	0.08573928	1.272216181
path_338	CCND1,CD274,CSF3R,CTNNB1,CYSLTR2,EIF4A2,FGFR3,H3F3C,MEF2B,MGA,MST1,MST1R,NF2,NOTCH3,PIK3CB,PLCG2,PLK2,PPP6C,STAT3,STK11	0.902639615	1.019470344

path_339	CASP8,CDK4,CDKN1A,CREBBP,INSR,JAK3,KNSTRN,MRE11A,MSH2,MST1R,NTRK2,PAK1,PTCH1,RPTOR,SMARCD1,SMO,SOX2,SPEN,STAT3,TET1	0.335275549	1.145449213
path_340	ATRX,AXL,BRIP1,BTK,CENPA,FGFR1,HIST1H3B,HLA-A,MAP2K2,MAP3K13,MCL1,MITF,MLL3,NCOR1,PGR,PPP6C,REL,SDHC,SMARCA4,SMARCD1	0.066059432	1.327551779
path_341	AKT2,ATM,CHEK1,CIC,ERCC4,FANCC,FGF4,FGFR1,FLT3,GATA1,GNAS,GRIN2A,KDM5A,LATS1,MLL,NUF2,PARK2,RAD21,SOX9,TGFBR2	0.87867995	0.978716423
path_342	AR,BAP1,BTK,CASP8,CDC42,CDKN1A,CDKN2C,ELF3,GATA1,HIST1H3D,HOXB13,JAK1,MDC1,MLL2,MST1R,MYC,MYOD1,SDHA,SUFU,TAP1	0.532278905	1.092251719
path_343	AKT1,APC,FAT1,IKBKE,KDR,KIT,PDGFRA,PIK3CA,PTPRS,RARA,RNF43,RPTOR,RTEL1,SDHC,SH2D1A,STAG2,STAT3,TBX3,TET1,TMPRSS2	0.459697203	1.122257241
path_344	ALK,APC,ARID2,BCL10,BCL2,EPHB1,FOXA1,HNF1A,IDH1,KDM6A,KMT2D,NKX3-1,NSD1,PAK1,PIM1,POLE,SMAD3,SOX17,SRSF2,TET2	0.068705032	1.360596574
path_345	CCND2,CD276,CRLF2,ERCC5,FOXO1,HGF,HIST1H3H,HIST1H3J,KDM5C,LATS2,MED12,MSH6,NFKBIA,PAK1,PAX5,PDCD1,PIK3CA,PIK3R3,RB1,SMARCD1	0.489936292	1.122992317
path_346	ABL1,CREBBP,CUL3,DAXX,E2F3,GATA1,HIST1H3B,HIST3H3,KDM5C,PLK2,PMAIP1,PMS1,PMS2,RB1,SDHAF2,SHQ1,SMAD3,STAG2,STAT3,STK40	0.698267179	0.946721735
path_347	ATM,BCL2,FAM175A,IFNGR1,IKBKE,MAX,MEN1,MLL2,MRE11A,MYCN,MYOD1,NPM1,NTRK1,PDGFRA,RAD21,RHEB,SDHAF2,TNFAIP3,TP63,TSHR	0.493376087	1.129274021
path_348	AKT3,ALOX12B,ETV6,FLT4,IGF2,INHA,KMT2D,KRAS,LATS1,MAP2K1,MAX,MGA,MLL,NSD1,PARP1,PIK3CG,PLCG2,RAC2,RASA1,TGFBR2	0.076971393	1.285611348
path_349	ALK,BCOR,CCND1,CDK4,CSF3R,EIF4E,FGF3,FOXA1,GLI1,GNAS,GSK3B,HIST1H3H,KMT2A,MDC1,MRE11A,MUTYH,PMAIP1,POLE,STAT5B,TET1	0.243680481	1.178952763
path_350	ALK,BRIP1,EED,ERG,ETV6,FGFR1,GATA2,GRIN2A,HIST1H3A,HIST1H3D,IGF1R,MEN1,MLL2,PDGFRA,PIK3C2G,RAD51,SDHC,SH2B3,SMAD4,SMARCA4	0.55700553	1.091189488
path_351	ATR,AURKA,CD276,CENPA,CHEK2,EPHA3,IGF2,IL7R,INHA,JAK2,JUN,KMT2A,LATS2,MRE11A,NOTCH2,NTRK2,PDGFRA,PIK3CD,POLE,RAD51D	0.554804845	1.109406455
path_352	AMER1,ASXL2,BCL2,CREBBP,EGFR,ESR1,FGF3,FGFR1,IKBKE,JAK2,KEAP1,KMT2A,MED12,MGA,NSD1,PIK3CD,PTEN,RASA1,ROS1,SHQ1	0.410797519	1.141908494

path_353	ASXL1,CDK12,ERCC5,ERRFI1,GSK3B,HIST1H3H,HIST3H3,ICOSLG,IKBKE,IRS2,KNSTRN,KRAS,MALT1,MRE11A,PALB2,PIK3CA,PLCG2,RB1,RECQL4,TOP1	0.528028204	1.097685804
path_354	AKT3,ATR,DROSHA,ERCC4,FOXA1,GPS2,HGF,HIST1H3D,HLA-A,IL7R,JAK1,KRAS,MLL2,MSH3,RAC2,RNF43,SDHA,SF3B1,SOCS1,TSHR	0.362091637	1.141495474
path_355	ARID2,ATR,BARD1,BLM,CCND3,CSF1R,E2F3,EPHB1,ESR1,FGF3,FGFR3,HNF1A,KDR,MET,MGA,MRE11A,NCOR1,RAD21,SDHC,STK40	0.056880826	1.363786691
path_356	BMPR1A,BRD4,CCND1,ELF3,ERBB3,FAM175A,FAT1,FBXW7,GLI1,GNAQ,H3F3C,HIST1H3D,NKX2-1,NOTCH4,PIK3CG,RAF1,RPS6KA4,SDHB,SUFU,TAP1	0.340191911	1.144119038
path_357	CREBBP,DAXX,EIF4A2,HIST1H1C,IRF4,IRS2,KDR,MALT1,MAP2K2,MAP3K1,MED12,MSH2,NCOR1,NFKBIA,NOTCH2,NPM1,NTRK3,PTPRD,ROS1,SMARCD1	0.357862296	1.149973116
path_358	AURKA,BRAF,CDKN2C,CHEK1,DROSHA,EZH2,FLT1,FOXO1,HIST1H3H,IKZF1,KRAS,NFE2L2,NKX2-1,PBRM1,PIK3R2,PTPN11,RECQL4,REL,STK40,TGFBR2	0.826126651	1.032242197
path_359	AXL,BTK,CDK12,FGF19,HIST1H3D,HOXB13,INHA,MAX,MEF2B,MUTYH,MYCL1,NEGR1,NFKBIA,PARK2,PHOX2B,PREX2,RAD51,RPS6KB2,SOCS1,SOX9	0.258137537	1.192656733
path_360	ALK,CCND1,CYSLTR2,ELF3,ERG,HIST1H3D,INSRR,IRS1,MDM4,MSH6,NCOR1,NOTCH4,PDCD1,PTPN11,RECQL4,RHOA,ROS1,STK40,TBX3,TGFBR1	0.170813123	1.215377393
path_361	ASXL2,ATRX,BRAF,BTK,CASP8,CENPA,CYSLTR2,EGFR,EIF4E,EZH2,FGF19,GLI1,HIST1H3E,MAX,MYC,PMS1,PREX2,RPS6KA4,SMARCD1,SOX2	0.594829092	1.102316757
path_362	ARID1A,ASXL2,BARD1,BCL2L1,DICER1,EPHB1,ERRFI1,FGF19,GNAQ,HIST1H3A,INHA,KDM5A,MDM4,MITF,NOTCH4,NPM1,PLK2,POLD1,SMARCD1,TET2	0.28029548	1.200468515
path_363	BRIP1,CARM1,CDH1,CSF1R,CTCF,ELF3,FANCA,FGFR3,IRS1,MCL1,MDM2,MYCL1,NF1,NTRK1,PDGFRA,PMS2,PNRC1,RAD54L,SDHC,SETD2	0.084646686	1.367466166
path_364	ALOX12B,BRIP1,CDC73,DICER1,EED,ERBB2,ESR1,FANCC,FAT1,FUBP1,NCOA3,NOTCH1,PDGFRA,PMS1,RAC2,RAD51,RET,RYBP,SH2D1A,TCF7L2	0.848470697	1.032485099
path_365	AMER1,AR,AXIN1,CDC42,CDC73,FAT1,FLT4,HIST1H3A,HIST1H3G,HIST3H3,NBN,NOTCH3,PMS2,PREX2,PTCH1,RBM10,RICTOR,SDHAF2,SMO,TERT	0.134070974	1.274596282
path_366	ALOX12B,ATM,AURKA,BCL2,CDK4,DOT1L,EIF4A2,FANCC,GSK3B,HIST1H3I,ICOSLG,IL7R,KMT2A,MALT1,MUTYH,PGR,RHEB,TBX3,TMPRSS2,TRAF2	0.79952146	0.955950905

path_367	ACVR1,ASXL2,BRIP1,BTK,CCNE1,DDR2,ERCC4,ETV1,FOXO1,GNAQ,IGF1R,KDM5C,MLL,NTRK1,PDGFRB,PTPRD,PTPRS,RAD51C,RFWD2,ROS1	0.067617169	1.33139422
path_368	ABL1,AKT2,BARD1,BRCA2,CD79B,ERBB4,ERCC4,FANCC,HIST1H3G,HIST3H3,MAP2K1,MDC1,MSH3,MYOD1,NF2,NOTCH1,NOTCH4,NTRK2,PIK3R2,RAD51	0.915952284	1.017669628
path_369	ARAF,ARID5B,ATRX,AXIN2,BCL6,CD274,ELF3,FUBP1,GSK3B,HNF1A,MALT1,MDM2,MET,MSH6,NFKBIA,NOTCH4,PREX2,RAF1,SH2B3,TGFBR1	0.559276077	1.108017732
path_370	ARID1A,ARID2,AURKA,BCL2L11,CARD11,CCND3,CHEK2,DOT1L,ESR1,FLT1,FOXL2,HIST1H3J,KMT2D,MRE11A,NEGR1,PBRM1,PDCD1,PMAIP1,RAD21,RPTOR	0.535298368	1.10991042
path_371	ARID1B,CD276,CD79B,CREBBP,DIS3,E2F3,FGFR1,FOXL2,HGF,HIST1H3C,HIST1H3D,NKX3-1,PARK2,PAX5,PTPRS,RAD51C,RARA,SMO,TERT,XPO1	0.547762166	1.088535655
path_372	AURKA,CSF1R,DROSHA,EPHB1,ERBB3,ERBB4,FUBP1,GNAQ,HGF,HLA-A,KNSTRN,MAP2K4,MSH6,MST1,MYC,NOTCH2,PLCG2,RARA,RFWD2,RYBP	0.082395313	1.27627618
path_373	BCL10,CDC42,CENPA,DDR2,DNMT3A,ERBB4,ERCC2,EZH2,FGF19,FLT3,GATA3,GPS2,HIST1H3A,HIST1H3J,MCL1,MDM2,MLL2,MTOR,PPP2R1A,RHOA	0.760525138	1.058676816
path_374	ARID5B,BBC3,BMPR1A,CD276,CHEK1,EPHA5,ERCC4,HIST1H3A,IGF1R,MTOR,NF1,NRAS,NSD1,PAK1,PALB2,PHOX2B,PPP6C,SDHB,SDHC,SPEN	0.762838566	1.05354649
path_375	BRAF,BRCA1,CD274,CREBBP,CTLA4,ERCC5,ESR1,GATA1,HIST1H3G,MAX,MGA,MST1R,NKX2-1,PALB2,PBRM1,PIK3CG,PIM1,PMAIP1,PPP2R1A,PPP6C	0.707409007	0.944539167
path_376	ASXL2,CSF1R,DNMT3B,DOT1L,ERCC4,FLT1,FLT3,GNA11,IDH1,KIT,MLL2,MLL3,MUTYH,NF1,NOTCH3,PAK7,PIK3CA,SDHB,SMO,SRSF2	0.7722209	1.04185167
path_377	ACVR1,ATR,B2M,BBC3,CREBBP,CRLF2,CSF3R,CYSLTR2,EGFR,HIST1H3G,KMT2D,MDM2,MYC,PDGFRA,PHOX2B,PIK3R3,PIM1,PPP6C,SMAD2,STAG2	0.817920119	1.044048213
path_378	ANKRD11,ARAF,AXIN1,CD79B,ERCC2,FAM175A,FGFR3,GRIN2A,HIST1H3H,MYCL1,NKX3-1,PIK3CG,PPP6C,RAD50,RYBP,SDHA,STK11,SYK,TCF7L2,TGFBR1	0.935949113	0.987119884
path_379	ACVR1,CASP8,CCND3,ERBB4,FANCC,FAT1,FOXL2,GLI1,HIST1H3C,IFNGR1,JUN,MGA,MITF,MSH2,NEGR1,NF2,NOTCH2,PTCH1,RASA1,TOP1	0.054729921	1.366200303

path_380	AURKA,CARM1,CCND2,CDKN1B,CREBBP,CTCF,FANCC,FLT3,FUBP1,INHA,INSR,KRAS,MSH6,PIK3R2,PLCG2,RAD21,RAD51,RECQL4,RICTOR,SMARCB1	0.701717886	1.059643557
path_381	ALOX12B,ARID2,ATM,CDC73,CENPA,DNAJB1,FGF3,GATA2,GATA3,HIST1H1C,HIST1H3B,KRAS,NFKBIA,NTRK1,NTRK3,PARK2,PTPN11,RAD54L,RPTOR,SH2B3	0.312364584	1.15867661
path_382	CCNE1,CD274,DDR2,DNMT3B,EED,FBXW7,FOXP1,H3F3C,HIST1H3G,HIST3H3,IGF2,IL7R,IRS2,MDC1,NUF2,PAX5,PLCG2,REL,RFWD2,SDHC	0.3728031	1.134595972
path_383	ASXL1,DAXX,DNAJB1,DROSHA,ETV1,EZH2,FANCC,INPP4B,MAP2K4,MEN1,MSH2,MTOR,NTRK1,PDGFRA,PIK3CD,PIK3R3,POLD1,RARA,RFWD2,SLX4	0.368719889	0.880857001
path_384	AKT1,ASXL2,BCL6,BRCA1,CDK4,FBXW7,FGF3,FOXA1,GRIN2A,IGF1R,KMT2D,MALT1,MAP2K4,MYC,NCOR1,PAK7,PIK3CG,SLX4,SUFU,SYK	0.798789694	1.043841906
path_385	ANKRD11,ASXL1,AXL,BRAF,EIF4A2,ERCC5,GNAQ,GPS2,INHA,MAP3K13,MET,MYCN,NTRK1,PBRM1,PHOX2B,PIK3C3,PPP2R1A,RFWD2,SOX9,TAP2	0.154530245	1.313140213
path_386	ARAF,BRD4,CARD11,CHEK2,DNMT1,ERCC5,GPS2,HIST1H3B,HIST3H3,IFNGR1,MALT1,MDM4,MLL3,MYCL1,NTRK1,PIK3CG,PRDM1,RPTOR,STAT5B,STK11	0.606430083	1.0827734
path_387	BCOR,CARD11,CENPA,ERBB3,HIST1H3C,MCL1,MSH2,MYC,NFE2L2,PAK1,PIK3CB,PMAIP1,RFWD2,RYBP,SDHAF2,SDHB,SOX2,SRC,STAT3,STK11	0.900255855	1.020580042
path_388	AURKA,AXIN1,CUL3,DNMT1,EZH2,FGFR2,GRIN2A,H3F3C,HIST1H3J,IRS1,KDM6A,MAP3K1,MST1R,NEGR1,NTRK1,PDCD1,POLD1,RAD21,SMAD3,SRSF2	0.148499003	1.225375788
path_389	CXCR4,DNMT3B,EED,EGFR,FUBP1,HIST1H3C,HIST3H3,IRF4,KIT,KNSTRN,MLL,NEGR1,NF1,NUF2,PBRM1,POLD1,PPP2R1A,RAC2,RAD51B,SRSF2	0.209686039	1.229167089
path_390	AURKA,BCL2,BRAF,CDK6,EGFR,ERCC2,FANCC,FLT4,HGF,MAP2K2,MLL2,NTRK3,PAX5,PREX2,RASA1,SDHB,SF3B1,SH2D1A,TAP2,TNFAIP3	0.256467768	1.191130115
path_391	AKT1,AXIN2,CIC,EIF1AX,EPHA7,FANCA,FGF19,HIST1H1C,HIST1H3I,IL7R,INPP4A,MTOR,NPM1,PAK1,PHOX2B,PTPRD,PTPRS,RNF43,RUNX1,TAP1	0.363107133	1.161434243
path_392	BCL2L1,BRD4,CCNE1,CIC,CREBBP,EGFR,EP300,FANCC,GNAS,INHBA,INSRR,MDM4,NOTCH3,NUF2,PAK1,PARK2,PIK3C3,PIK3CB,PLK2,RAD51	0.573497061	1.098709021
path_393	AXIN2,BCL2L1,CALR,CDK4,CDK6,CXCR4,DIS3,HIST1H1C,HIST1H3C,HIST1H3E,HIST1H3I,IRS1,IRS2,MAP3K1,MST1R,PHOX2B,POLD1,POLE,RFWD2,RICTOR	0.196216813	1.199772804

path_394	CHEK1,DAXX,DIS3,DROSHA,EIF1AX,FGFR4,HGF,HIST1H1C,HIST1H3B,HOXB13,JAK3,KDM5C,KIT,MSH3,MTOR,NEGR1,PDGFRB,PIK3CB,PIK3R2,RECQL4	0.82639361	0.969472629
path_395	ALK,BCL2L11,CDKN2A,CDKN2C,CTLA4,DNMT3A,EIF1AX,ERCC4,FANCA,FLT1,INHA,KDM5C,MST1R,MYCN,NFE2L2,NOTCH3,PBRM1,SMAD4,SMARCB1,STK40	0.558722263	1.095872313
path_396	ABL1,BMPR1A,CHEK1,CSF1R,GNAQ,IFNGR1,INPP4B,MDC1,MSH3,NFE2L2,NKX3-1,NOTCH2,PBRM1,PNRC1,PPM1D,PTPN11,RAD50,RARA,SDHB,SF3B1	0.132836748	1.332086558
path_397	AKT3,ALOX12B,BARD1,BCL2,CDKN1A,CDKN2A,ERBB3,ERCC5,FOXO1,HOXB13,IKZF1,INPP4A,MAP2K2,MLL2,PHOX2B,RAD50,SHQ1,SMAD2,TAP1,TOP1	0.145153082	1.330419582
path_398	ASXL1,BCL2L11,BCL6,BCOR,CDKN2C,DNMT3A,GRIN2A,INHA,KIT,KMT2A,MDM4,MITF,NBN,PIK3R3,RAD21,RAF1,RHEB,RICTOR,RYBP,SOX9	0.899029028	0.982215542
path_399	APC,ARID1A,BCOR,BMPR1A,CDH1,DNAJB1,ERCC2,ESR1,LATS2,PHOX2B,PTEN,RAC2,RHEB,RYBP,SETD2,SHQ1,SMARCA4,SOX17,STAT5B,TMPRSS2	0.723188798	1.057799025
path_400	ATRX,BCL2L11,EPHB1,ERCC4,FAM46C,FGF4,ICOSLG,IGF1R,IGF2,JAK3,KRAS,MEF2B,MST1,NCOA3,PAK1,PPM1D,PPP6C,RFWD2,SRSF2,TET2	0.099579867	1.30339995
path_401	ARID5B,BARD1,CALR,CCND2,DNMT1,ELF3,ESR1,FBXW7,KDM5C,MLL,MLL2,MUTYH,NCOA3,NFE2L2,PPP2R1A,RAD21,SH2D1A,SLX4,STAT5B,YAP1	0.130324795	1.284687581
path_402	ASXL1,ASXL2,BBC3,CD79B,CTNNB1,DAXX,DNMT3A,ERRFI1,FOXL2,GATA2,IDH1,IFNGR1,MUTYH,MYOD1,NF2,NSD1,PPP6C,RET,SH2D1A,SMARCA4	0.597615805	1.088817258
path_403	ACVR1,CARD11,DNMT3B,EED,FAM175A,FAM46C,IKBKE,INHBA,MSH2,PDCD1,POLD1,PTPRT,RAD51,RPS6KB2,RTEL1,SHQ1,SMARCD1,STAT5B,SUFU,SYK	0.185536911	1.206021801
path_404	BCL2,CDK4,CDKN1A,DNMT3B,HIST1H3B,INPP4B,MALT1,NBN,NCOR1,NEGR1,NFE2L2,NOTCH1,NOTCH3,NTRK2,PTPRS,PTPRT,RB1,SH2B3,SH2D1A,SMARCD1	0.051827477	1.364444731
path_405	ABL1,BAP1,CD274,CIC,CYSLTR2,ERRFI1,FGFR1,GRIN2A,HIST3H3,IGF2,MLL2,MYC,NBN,PALB2,PBRM1,PTPN11,RAD51C,RFWD2,SDHB,SDHC	0.672525996	1.061513021
path_406	B2M,CCND2,CDKN1A,CHEK1,DDR2,E2F3,EGFR,EPHA7,FAT1,FOXO1,GLI1,MLL,MPL,NEGR1,PDGFRB,PGR,PIM1,RAD51,RICTOR,STAT5B	0.383877981	1.154936946
path_407	AKT1,AKT2,AXIN2,CDC42,DOT1L,EPHA5,ERBB3,ERG,FLT1,KDM6A,KMT2A,MGA,MSH2,MST1,NCOR1,NOTCH1,PIK3CA,RUNX1,SF3B1,SMARCB1	0.508297348	1.10734292

path_408	BMPR1A,CDK6,CDKN1A,CTLA4,EIF4A2,EPHB1,HIST1H3I,IGF1R,INPP4B,MAP2K4,MCL1,MITF,NRAS,PIK3CB,PLK2,POLE,RARA,TAP1,TNFAIP3,YES1	0.6785074	1.061753257
path_409	AKT3,CCND1,CHEK2,DOT1L,ERBB4,EZH2,FGF19,FOXA1,HLA-A,IFNGR1,JAK2,KDM5A,KRAS,MAP3K13,MLL3,NCOA3,POLD1,RET,RFWD2,SMAD4	0.507950816	1.110673081
path_410	AKT1,BRAF,CCND3,CDKN2A,CSF1R,EED,FOXP1,IL7R,JAK2,MCL1,MRE11A,MYCN,NCOR1,NEGR1,PHOX2B,RAD51C,RAF1,SDHA,SOX17,TMPRSS2	0.291370909	1.161207265
path_411	ATR,BBC3,BRIP1,CDKN2C,CTNNB1,DNMT3B,DOT1L,EIF4A2,ESR1,GATA2,KDM5A,MAP2K4,MEF2B,MLL3,PIK3C2G,PIK3CB,RPS6KB2,RUNX1,SPEN,SYK	0.129032766	1.287163444
path_412	ABL1,CARM1,CUL3,EIF1AX,EPHA3,FBXW7,FYN,HIST1H3A,HIST1H3I,IL10,INHA,IRF4,MYOD1,NF2,NRAS,PTPRS,RAD21,RAD50,RARA,TSC2	0.496334986	1.130874986
path_413	AKT1,ASXL1,BARD1,BRD4,DNMT1,DROSHA,HIST1H3I,HLA-A,INPP4B,KIT,MST1R,MUTYH,MYC,NBN,PARP1,PIK3R3,RHEB,RNF43,SLX4,SOCS1	0.135574446	1.234103908
path_414	BCL2L1,BRAF,CDK4,CSF1R,EPHA5,ERCC2,FBXW7,FGFR1,IGF2,MAP3K13,MET,MYOD1,NEGR1,NOTCH4,PAK7,PIK3CG,PTCH1,PTPRS,RB1,RUNX1	0.411949333	1.128788439
path_415	ACVR1,ALOX12B,ATRX,CSF1R,ERBB2,ESR1,FLT4,FOXL2,FYN,HIST3H3,IGF2,LATS1,MET,MTOR,NKX3-1,NOTCH1,PDGFRA,PMS1,POLE,PPM1D	0.222837643	1.214279541
path_416	ALK,AMER1,B2M,CCND2,CD276,EIF1AX,ERG,FANCC,KDM5A,KRAS,MTOR,NCOR1,NOTCH4,NTRK3,PIK3CB,RAD51C,RB1,ROS1,SDHAF2,TCF7L2	0.127975752	1.253652858
path_417	B2M,CD79B,CEBPA,CHEK1,EPHA5,ERBB4,FGFR1,GATA3,HIST1H3D,JAK3,KDM5A,MCL1,MITF,MRE11A,MYC,PIM1,RASA1,RB1,RPS6KB2,SMARCD1	0.109888423	1.316796473
path_418	AKT2,ARID5B,BMPR1A,CDK6,CTCF,CYSLTR2,FBXW7,HNF1A,JUN,KDR,MDM2,NBN,PDGFRA,PIK3CB,PIK3R1,PIK3R3,POLE,RUNX1,SPOP,TAP2	0.327551487	1.150155668
path_419	ALK,ASXL2,CTCF,E2F3,FGF3,FOXP1,GSK3B,H3F3C,HGF,HIST3H3,IFNGR1,IGF1R,JAK3,MITF,MST1,NKX3-1,PARP1,POLD1,SDHB,YAP1	0.326838203	1.148780622
path_420	AKT1,ATR,CDH1,DAXX,DIS3,EPHA3,ERCC4,ERG,FGFR1,FLT1,FUBP1,IKZF1,MAP3K13,MDM2,NF1,PIK3R1,RAF1,RTEL1,SH2B3,SMO	0.852133436	1.033664181
path_421	ANKRD11,CALR,CD274,CDKN2A,CHEK1,EP300,FAM46C,FBXW7,FYN,HNF1A,HOXB13,MSH3,MTOR,MYCL1,MYOD1,NKX2-1,RAD54L,REL,SETD2,SMARCD1	0.661047709	1.085561632
path_422	AXL,BBC3,CCND1,CSF1R,CSF3R,DAXX,DDR2,FGF19,FGF4,FOXL2,MALT1,MAX,MLL2,MST1R,PPM1D,PTPN11,PTPRD,RAD54L,RICTOR,SF3B1	0.118691358	1.276368496

path_423	ANKRD11,AR,ATM,BCL2,CCND2,EPHB1,GLI1,KIT,KLF4,KMT2A,LATS1,MET,MSH3,MYOD1,PDGFRA,RHOA,SMAD3,SOX17,SPEN,STAT5B	0.318827883	1.188559296
path_424	CDC42,CTLA4,DNAJB1,ERBB3,FAM46C,FANCC,FGFR4,H3F3C,HIST1H3A,IDH1,KMT2A,MRE11A,NPM1,PMS2,RAD50,RAD54L,RB1,RTEL1,SMAD2,SRSF2	0.607201716	1.077478271
path_425	ANKRD11,BMPR1A,CASP8,CDK12,CREBBP,GRIN2A,GSK3B,HIST1H3G,IKZF1,KIT,MAP2K2,MSH6,MTOR,NOTCH3,NRAS,PDGFRB,PPP6C,RASA1,ROS1,RUNX1	0.1744931	1.248291718
path_426	ASXL2,AXL,CCND1,CD276,CREBBP,DDR2,EGFR,ETV6,FAT1,FGF19,IDH1,IL10,KRAS,MAP3K1,MED12,NKX2-1,PMAIP1,RAF1,SMAD2,TP53	0.305986865	1.159356009
path_427	AKT1,APC,ASXL1,CTLA4,EGFR,EPHB1,ERCC4,FOXA1,GSK3B,HLA-A,IGF2,KMT2D,NF1,NTRK2,PLK2,PPM1D,RAD21,RAD50,RASA1,SMAD2	0.881367938	1.025463967
path_428	ABL1,ATRX,BCL10,CDC42,FAM175A,FANCC,GATA2,HOXB13,KDM6A,MAP2K2,MED12,MPL,NCOR1,NFKBIA,PIK3C2G,PRDM1,RICTOR,SOX9,TERT,TGFBR1	0.076987466	1.370410916
path_429	AMER1,ARID1A,CARD11,CD276,CDH1,CTLA4,DNAJB1,FANCC,FGF4,FGFR2,FOXP1,GATA3,HIST1H1C,HIST1H3D,MAX,MLL3,PALB2,PDGFRA,RAD51,RYBP	0.12076448	1.312372388
path_430	AR,AXIN1,BRCA2,CTLA4,DNMT3A,EIF4A2,ERCC2,FAM46C,FOXP1,GNAQ,H3F3C,HIST1H3C,HLA-A,MLL3,MSH2,PLCG2,PLK2,RASA1,RNF43,SETD2	0.271052682	1.201713094
path_431	ACVR1,BRCA2,CD274,CHEK1,FLT1,HIST1H3G,HIST1H3I,KIT,MAP2K2,MAX,PBRM1,PIK3CA,PIM1,POLD1,PPM1D,PRDM1,PTPN11,SMAD4,SOS1,STAT3	0.760027931	1.056890288
path_432	AKT3,ALOX12B,AXIN2,BARD1,BRCA2,CENPA,EIF4A2,ERRFI1,FH,HIST1H3E,IFNGR1,JAK2,JAK3,KRAS,PIK3CA,PLCG2,PTPRT,RASA1,RICTOR,ROS1	0.128739877	1.246425199
path_433	AR,AURKA,BTK,ERCC4,HIST1H3E,IL7R,INHA,MAP3K1,MSH3,NCOR1,NRAS,PAK7,PHOX2B,RAC2,RAD21,RPS6KB2,SDHC,SH2D1A,SMARCA4,TP53	0.269017614	1.170375379
path_434	BRCA2,CDK8,DNMT3B,DROSHA,FGF4,FGFR1,GRIN2A,HIST1H1C,HIST1H3B,HIST1H3G,INSR,KMT2D,MAP3K1,MYC,NOTCH4,PNRC1,POLE,RTEL1,SMAD3,STK11	0.551029838	1.099783598
path_435	BBC3,BRAF,CASP8,DAXX,EGFR,EPHA3,ESR1,FLT3,KDM6A,MSH6,NBN,NF2,NKX3-1,PIM1,PMS2,POLD1,ROS1,SETD2,SMAD2,SMARCD1	0.090792014	1.302630793
path_436	BBC3,CARM1,CD79B,CDH1,CDK8,DICER1,ERCC5,ERG,ETV6,FOXA1,GATA2,GATA3,GRIN2A,IKBKE,NFKBIA,NOTCH4,PAK7,PPM1D,PTPRS,STAT5B	0.140574198	1.231470563

path_437	AR,CD276,CDC42,CTLA4,EGFR,GNAQ,HIST3H3,IL7R,INSRR,JAK3,KEAP1,MSH3,NPM1,PAK1,PARP1,PAX5,PGR,PTPRT,RAD54L,RHEB	0.735254131	1.052336576
path_438	ARID5B,ASXL1,BRCA2,CDK12,CHEK1,CHEK2,EPHA5,FBXW7,FLT1,FOXL2,IDH1,MET,MYCN,NPM1,PHOX2B,PLCG2,POLE,RAD51B,SETD2,TP63	0.750216356	1.049845382
path_439	AKT3,BCL2L11,BRD4,CTLA4,EPHA5,EPHB1,EZH2,FGFR3,FOXP1,GNAS,MAP3K13,MCL1,MITF,PDCD1,PLK2,PPP2R1A,PTEN,RAC2,RYBP,SLX4	0.345339163	1.142560395
path_440	BRD4,CREBBP,CSF1R,DICER1,EED,EIF4E,ERRFI1,FGF3,FH,FYN,HIST1H3B,HIST1H3D,IL7R,INSRR,KMT2A,MRE11A,MSH6,RPS6KA4,SHQ1,STAG2	0.27134668	1.177573819
path_441	ABL1,ARAF,BCL2L1,CD276,CTCF,CTLA4,FAM46C,FGF3,GLI1,HIST1H1C,IRF4,JAK3,KLF4,MAP3K13,MSH3,MST1R,NOTCH4,PLK2,RBM10,SMARCB1	0.453522765	1.126135322
path_442	ASXL1,ASXL2,AXIN2,EPHA5,HIST1H3J,KDR,MPL,NFKBIA,NUP93,PIK3C3,RAD50,RAF1,REL,RNF43,ROS1,SOX2,STAT3,TAP2,TOP1,TRAF7	0.433085251	1.150496927
path_443	ARID1A,ASXL2,BCL2L1,CDC42,EPHA7,ERCC2,ETV1,GPS2,HIST1H1C,IKZF1,IRS2,KEAP1,MLL2,NOTCH3,POLE,RAD51B,RAD51C,RASA1,RICTOR,STAT5B	0.202274943	1.202439336
path_444	APC,ARAF,BRIP1,CALR,CARD11,CDK6,DOT1L,EP300,ETV6,H3F3C,NBN,NCOR1,NTRK2,PIK3C3,PIK3R3,PMAIP1,PTPN11,PTPRT,RASA1,RBM10	0.250288315	1.186130344
path_445	AURKA,CCND2,CDC73,CDKN1B,DAXX,ERCC5,FGFR2,FLT3,FOXL2,GRIN2A,HIST1H3G,INSR,INSRR,IRF4,KMT2D,KRAS,MALT1,PNRC1,RAC2,RAD54L	0.552028741	0.911426525
path_446	ALOX12B,ATR,BBC3,BLM,CDK8,CSF3R,DAXX,ERCC4,FBXW7,FLT3,FUBP1,HIST1H3B,IGF2,IKZF1,MLL,PAK7,PTCH1,RICTOR,SDHAF2,STK11	0.788841358	1.045460077
path_447	APC,ATM,CDK6,CDKN1A,CREBBP,DICER1,ELF3,FGFR4,GATA3,INSR,KRAS,NF2,NUP93,PBRM1,PIK3CG,RAD54L,RHOA,SHQ1,SPEN,TET2	0.600873286	1.097076794
path_448	ATM,BRCA1,CHEK1,EIF4A2,ERRFI1,FAT1,FUBP1,GNAQ,HIST1H1C,HOXB13,IFNGR1,MED12,MPL,MTOR,MYOD1,NF2,NFE2L2,NOTCH4,PLK2,SOS1	0.081771817	1.318648727
path_449	CDC42,DAXX,FLT3,GNAS,HLA-A,IKBKE,INPP4A,INSRR,IRF4,MCL1,NCOR1,NOTCH3,NTRK1,PLCG2,REL,RET,SHQ1,TEK,TET2,TOP1	0.118043447	1.247691854
path_450	BRIP1,CBL,CDC73,ERBB3,ERG,FH,KDM5C,KRAS,MAP3K13,MEF2B,NTRK2,PALB2,PMS2,POLD1,POLE,PTPRD,RAD51D,REL,SHQ1,TCF7L2	0.21835099	1.19870064

path_451	BCL6,CALR,CDC42,CDK12,CHEK1,ERG,ESR1,FGF4,INPP4A,NCOR1,NOTCH4,PAK7,PIK3C3,PIK3CA,PIK3R1,PTPRS,SDHAF2,SMARCB1,SOX17,STAG2	0.897715225	1.022056926
path_452	AR,BAP1,BBC3,CALR,CTNNB1,ERCC2,FAM46C,FGFR4,FOXL2,HIST1H3I,HOXB13,IRS1,MDM4,MTOR,NSD1,PMAIP1,PTCH1,RET,SDHA,SHQ1	0.989001525	0.998048981
path_453	BRCA1,CARM1,CBL,E2F3,EIF4E,EPHA7,ETV6,FGFR2,GPS2,INHA,IRS1,KRAS,MLL3,NCOA3,NF1,PDGFRB,PIK3R3,RNF43,ROS1,RUNX1	0.156094758	1.221988862
path_454	AMER1,CASP8,CXCR4,DNMT3B,EPHA3,ERCC4,FAM46C,FAT1,FGF3,FLT1,FOXO1,HIST3H3,IDH1,MAP2K2,MYOD1,PIK3R1,PTCH1,RUNX1,SRC,STAT5B	0.612577529	1.082033104
path_455	ARID1A,AXIN1,AXIN2,BMPR1A,CEBPA,ERG,FOXL2,FUBP1,GATA1,GATA3,HIST1H3C,IGF1R,LATS1,MAP3K13,MSH6,PHOX2B,SYK,TGFBR1,TP53,WT1	0.154897706	1.229144094
path_456	AKT3,ATM,AXIN1,BCL10,FANCC,FGF19,FGF4,GRIN2A,HIST1H3I,KDM5C,MDM4,MED12,MEF2B,PPM1D,PTPRS,RAC2,RHEB,RICTOR,SMAD2,TEK	0.096228747	1.264863272
path_457	AR,ARID1B,ASXL1,BAP1,BRCA2,CSF1R,EP300,ETV1,FAM175A,HIST1H3A,HIST1H3G,INPP4B,INSR,MDM2,PMAIP1,RAD51,RET,SLX4,SUFU,TAP2	0.071022721	1.291670387
path_458	ARID5B,BRCA1,CARD11,DNMT3A,ERBB3,ETV6,FGF19,GSK3B,HIST1H3J,HOXB13,IKBKE,INHBA,MALT1,MAP2K1,MLL,MYCN,NKX3-1,PAK1,RB1,TET2	0.231482563	1.22677426
path_459	AXIN2,BARD1,BBC3,CDK12,CDK4,CHEK2,DNAJB1,FAM175A,FGF19,HLA-A,HOXB13,NKX2-1,NRAS,PBRM1,PMS1,RAC2,RAD51D,RBM10,RTEL1,SRC	0.645713202	0.937226208
path_460	ABL1,ASXL2,BCL2L1,CDK8,CDKN2A,DROSHA,ETV6,FGFR3,GLI1,IKBKE,INPP4B,IRF4,JAK3,MAP3K13,MITF,NOTCH3,PIK3CB,RAD51D,RASA1,STAT5B	0.159790675	1.218577655
path_461	AR,ATM,ATR,B2M,BRAF,CCND3,CDKN1B,ERCC5,HIST1H1C,INHBA,INPP4B,PAK7,PDGFRB,PNRC1,PPP2R1A,PRDM1,PTPRS,RFWD2,RYBP,SDHAF2	0.062909304	1.362573672
path_462	AKT1,ALOX12B,CD274,ESR1,ETV1,GSK3B,HIST1H3J,IRS2,NFKBIA,NOTCH2,NSD1,PAK7,PIK3C3,PIK3CD,PIK3CG,POLD1,PTPRD,RAD54L,RB1,SMARCA4	0.157531702	1.226260177
path_463	ANKRD11,BMPR1A,BRAF,BTK,CDK12,FGFR3,INSR,JAK2,MAX,MTOR,MYCL1,NOTCH3,PARP1,PIK3C2G,PIK3R3,PMS1,POLE,RYBP,SOX2,STAT5B	0.543902082	1.090888832
path_464	ANKRD11,ATR,BRCA1,CASP8,DDR2,EIF4A2,FANCC,FOXP1,HIST1H3J,KDM5C,NOTCH1,PARP1,PAX5,PGR,PIK3R2,RAF1,SMARCD1,STK11,SYK,TP53BP1	0.494577946	1.108541629

path_465	CARD11,CDC42,CSF3R,EGFR,EIF4A2,EIF4E,ERBB4,FLT1,HIST3H3,IGF1R,IRF4,JAK2,MEN1,MPL,NBN,NEGR1,NKX2-1,PDCD1,PIK3CA,RBM10	0.629055085	1.075037495
path_466	AKT1,BMPR1A,CBL,CCND1,CDK12,CDKN2A,CENPA,DNMT3B,EPHB1,FAM175A,FOXL2,MAP2K1,MYCL1,NOTCH3,PREX2,RAD51D,RPS6KA4,SETD2,SH2D1A,STAG2	0.206133619	1.263030829
path_467	ASXL1,ATR,BBC3,CDK8,DNAJB1,ERG,HIST3H3,IL10,MAP3K1,MAP3K13,MCL1,MLL3,PALB2,PIK3CD,PPP6C,PREX2,PTPN11,RHEB,ROS1,SDHAF2	0.262110565	1.176837441
path_468	AKT3,ATR,CHEK2,CYSLTR2,DNMT3A,EPHA7,ERBB4,FGF4,FGFR4,FOXL2,HGF,ICOSLG,IGF1R,INSRR,KDR,MYC,PTPRS,RAD51,RNF43,TGFBR2	0.10681167	1.303034645
path_469	ARAF,CRLF2,DROSHA,EED,EGFR,ERCC2,ETV6,KMT2D,MYC,MYCL1,NCOR1,NF1,NSD1,PRDM1,RAD51B,SLX4,SPEN,SPOP,TAP2,TRAF7	0.433779245	1.157495054
path_470	BARD1,BCL2L11,BMPR1A,BRIP1,ERBB2,ERG,GRIN2A,IGF1R,IKZF1,INPP4B,JAK1,KDR,KLF4,MAX,MPL,NOTCH4,PIK3CG,SDHA,TBX3,TNFAIP3	0.28366633	1.178962391
path_471	ASXL1,BARD1,CDKN2A,CHEK1,CTCF,DOT1L,GATA1,IRF4,KMT2D,NCOA3,NTRK1,PAK7,PDGFRA,PGR,PMAIP1,RAD51C,RASA1,RBM10,RECQL4,RNF43	0.087031721	1.308482254
path_472	AMER1,CCND2,CDKN2A,CSF1R,EIF1AX,EPHA5,ERCC2,FAM46C,GPS2,HIST1H1C,HIST1H3E,HIST1H3I,ICOSLG,JAK1,KMT2D,MET,MLL2,PDGFRB,PIK3CB,SDHB	0.848044669	0.971102275
path_473	AR,BAP1,CARM1,CEBPA,CREBBP,DAXX,DOT1L,ERG,FOXA1,INSR,IRS2,KNSTRN,LATS2,MEN1,MPL,NKX2-1,PAK7,PLCG2,RFWD2,SDHC	0.103613116	1.31731713
path_474	ANKRD11,BMPR1A,CRLF2,DIS3,DROSHA,EPHA5,ERBB3,ERCC2,FAM46C,FGFR2,GATA3,HIST1H1C,HIST1H3A,INSRR,KMT2A,KMT2D,MRE11A,PLCG2,RAD51B,SHQ1	0.775463585	0.960388337
path_475	ARID5B,ASXL2,BCL6,CCND1,CDK8,CEBPA,CSF1R,CTNNB1,FGFR3,HIST1H3C,HIST1H3H,INPP4A,KDM5C,MSH2,NF2,NOTCH4,PIM1,PPM1D,PTCH1,RYBP	0.135000853	1.328867848
path_476	ANKRD11,BLM,DNAJB1,DNMT3B,ERRFI1,FGFR4,FLT3,GATA1,HNF1A,IRF4,MRE11A,NOTCH3,NTRK2,PLCG2,RAD51,SHQ1,SRSF2,TNFAIP3,TRAF2,U2AF1	0.158226591	1.224018573
path_477	BCL10,BLM,BRCA2,CD274,DAXX,FAT1,FOXP1,INPP4A,JAK1,LATS2,MAP2K4,MSH3,NKX2-1,NOTCH2,PLCG2,RAD51C,RASA1,RFWD2,SOS1,STAT5B	0.646551273	1.086651868
path_478	AKT1,EGFR,ESR1,FANCA,FLT3,HIST1H3D,IKBKE,INSR,JAK3,KDM5A,KDM6A,MAP2K1,NCOA3,PARP1,PTPRD,RAD51B,SDHB,SHQ1,SLX4,SMARCA4	0.311019728	1.17096135

path_479	AKT1,ATM,BCOR,CDKN1A,DNMT3A,ERBB3,ETV6,ICOSLG,IRS2,KDM5A,MCL1,NF2,NTRK3,PAK7,PIK3C3,RAD51D,REL,RNF43,RPS6KA4,SH2B3	0.071949789	1.323646277
path_480	EP300,EPHA3,ESR1,FGF19,GNAQ,IKBKE,INSRR,MAP2K1,MST1R,NFKBIA,NKX3-1,PNRC1,PTPRD,RFWD2,RHEB,RYBP,SOS1,SPEN,STK40,TGFBR1	0.201236253	1.22248779
path_481	CASP8,CTCF,CXCR4,ERCC4,FGF19,FUBP1,FYN,IDH1,JAK2,LATS1,MYCL1,MYOD1,NCOA3,NSD1,PALB2,PMS1,PTCH1,RAF1,RB1,RNF43	0.21880677	1.189094316
path_482	ALOX12B,CIC,DAXX,ERBB3,EZH2,GATA1,HIST1H3I,IDH1,IFNGR1,INPP4A,INSRR,KMT2A,MYCN,NUF2,PDGFRB,RAD51D,ROS1,SETD2,SH2B3,TGFBR2	0.632827282	1.070134117
path_483	ABL1,ARAF,B2M,CTLA4,DAXX,ELF3,ERBB3,FOXA1,IDH1,IL7R,INHA,IRS1,IRS2,KNSTRN,MAP2K1,MPL,MST1R,NTRK1,RASA1,RPTOR	0.862432827	0.975665715
path_484	ARID2,AURKA,BCL10,BCL2,CDK6,DNAJB1,DNMT3A,EED,EZH2,FOXL2,HIST1H3A,INPP4A,MEF2B,MUTYH,PDGFRB,PIK3C2G,PIK3R3,REL,SMAD4,SMO	0.605333404	1.096115459
path_485	ARID1B,ARID2,BRD4,CEBPA,CREBBP,ELF3,ERBB2,ERG,FAT1,FGFR1,GNA11,HIST1H3B,IGF2,JAK2,KDM6A,MLL,NUP93,RAD51,SPOP,TP63	0.39414359	1.154382999
path_486	ASXL1,AURKA,BCOR,CARD11,CDC73,CTCF,FGF19,FGFR2,FH,H3F3C,IGF1R,INHBA,INPP4A,MAP2K1,MPL,MYCL1,NPM1,NTRK3,PIK3CG,PRDM1	0.331669454	1.177622694
path_487	BARD1,BCL2L11,CCND3,EIF1AX,EP300,FLT1,IDH1,IGF1R,IKZF1,IRS1,MITF,NEGR1,PARP1,PRDM1,RAD54L,RASA1,SMO,STAG2,STAT3,WT1	0.515189232	1.096050237
path_488	ATM,CARM1,CDK8,CIC,DICER1,EIF4A2,HIST1H3A,IDH1,KMT2D,MAX,MED12,MET,MLL2,MST1R,PIK3CA,PTPN11,SOCS1,SPEN,STAG2,SYK	0.296839037	1.191865876
path_489	ASXL1,AXIN1,AXIN2,BCL2L1,BRCA2,DROSHA,EIF1AX,EIF4A2,ETV1,HIST1H3A,MLL,MSH3,NSD1,PARP1,PBRM1,PIK3C3,PTPRD,SOCS1,SOX2,TCF3	0.066671889	1.367602038
path_490	ARID2,BCL6,CDK12,DNAJB1,EPHA7,ERRFI1,IL7R,INSRR,LATS1,MAP3K1,MAP3K13,MYCN,NF2,NKX2-1,NTRK2,PIK3R1,PPM1D,RPTOR,SOCS1,TAP1	0.087633693	1.361020728
path_491	ARID5B,BLM,BMPR1A,BRCA1,CBL,CCNE1,DICER1,EIF1AX,EIF4E,ERCC5,FGFR3,FOXO1,H3F3C,KDM5C,MALT1,MPL,NF1,NTRK2,NUP93,PMAIP1	0.215554732	1.25703585
path_492	AMER1,ARID2,BCL2L11,BRCA1,CDC42,DOT1L,EGFR,FOXA1,FUBP1,MALT1,NCOR1,PDGFRA,PTEN,RAC2,RAD51,RAD51B,RB1,RHEB,SDHA,TAP1	0.344472159	1.156236968

path_493	ANKRD11,BCL2L1,BMPR1A,CCND3,CEBPA,EIF1AX,EIF4A2,FLT3,FOXO1,GATA2,IGF2,IKBKE,IL10,MET,MRE11A,NUF2,RAD51C,RBM10,RNF43,ROS1	0.243311628	1.257592515
path_494	AKT3,BCL2,BCL2L1,CARD11,CCND1,CRLF2,CSF3R,DAXX,DROSHA,EPHA7,FLT4,LATS1,MDM4,NFE2L2,NOTCH4,NUF2,PTPRD,RAD51C,SRSF2,TBX3	0.21986969	1.223538544
path_495	AMER1,ATR,CD276,CDK6,CDKN2C,CXCR4,EP300,FLT4,FOXO1,GNAS,IGF1R,KLF4,MDM4,PALB2,RAD21,RAD51C,RHEB,SH2B3,SOCS1,SRC	0.065799155	1.302314258
path_496	ATR,BCL2L11,CDK4,DNAJB1,DNMT1,EIF4E,FANCA,FANCC,GLI1,HGF,KDR,PALB2,PIK3C3,PIK3R3,RPTOR,RUNX1,SDHA,SMO,SOX17,TEK	0.482147998	1.104932444
path_497	ALOX12B,APC,BCL2,BCL6,ETV6,HIST1H3B,HIST1H3C,IDH1,KDM5C,KNSTRN,NFKBIA,NKX2-1,NOTCH1,PARK2,PIK3C2G,PMAIP1,PMS2,PPP2R1A,PTCH1,RAD51B	0.492561093	1.129276607
path_498	ACVR1,APC,AR,BRD4,EPHA3,EPHB1,ERCC2,ERCC4,ESR1,FGFR2,FOXO1,FOXP1,PDGFRA,PTPRS,RAD51D,RAD54L,SDHAF2,SDHC,SF3B1,STAT3	0.11682119	1.3199437
path_499	ASXL2,BRCA2,CDKN1A,CSF1R,DIS3,DNAJB1,DNMT1,EGFR,ERCC4,ESR1,EZH2,FAM46C,NBN,PALB2,PBRM1,PIK3CA,STAT3,STAT5B,TMPRSS2,TRAF7	0.443845459	1.140883566
path_500	AKT2,CCND3,EED,ERCC5,FGFR1,HIST1H3A,HIST1H3B,INSRR,KDM6A,LATS1,MLL2,NOTCH1,NUF2,PGR,PMS2,RARA,RICTOR,SDHC,SOCS1,SOX9	0.414677662	1.122456859
path_501	ARAF,BCL6,CBL,CCND2,CEBPA,CTNNB1,DNMT3B,HIST1H3A,HIST1H3D,HIST3H3,IL10,MRE11A,MTOR,NCOR1,NKX2-1,PLCG2,RAD51B,SETD2,SMAD4,SOX2	0.124371085	1.316252886
path_502	AXIN1,AXIN2,BBC3,BCL2,BRCA2,CDKN1B,CIC,EP300,ERBB3,HIST1H3I,ICOSLG,INHBA,KLF4,MALT1,MEN1,MSH2,NBN,NCOR1,PIK3CD,PIK3CG	0.107757486	1.349925561
path_503	CARM1,CDKN2A,EPHA7,EZH2,FLT4,HIST1H3I,HLA-A,IDH1,IGF1R,IRS1,KDR,MDC1,NFE2L2,PDCD1,PPP2R1A,REL,SDHC,SH2B3,STK40,TRAF2	0.101095121	1.311468108
path_504	ALOX12B,AURKA,AXIN1,BRCA2,FAM46C,FAT1,HIST1H3I,KDM6A,KMT2A,MAP2K2,MET,MSH6,NCOA3,PHOX2B,RBM10,RTEL1,SDHB,SHQ1,TAP1,TEK	0.106439416	1.307757916
path_505	ACVR1,ANKRD11,ATRX,CCND2,CCNE1,ERCC2,FOXL2,FYN,HIST1H1C,HIST3H3,IGF1R,IL7R,KDR,KIT,KRAS,MYC,PBRM1,PMS2,TRAF2,TSHR	0.335526862	1.154853291
path_506	CARD11,CARM1,CDK4,CENPA,CHEK2,FOXA1,FOXL2,HIST1H3C,HLA-A,IDH1,LATS1,MDC1,MED12,MSH2,NF2,NUP93,PIK3CG,PNRC1,RAD51B,RAD54L	0.162323714	1.287004814

path_507	CARD11,CHEK1,FAT1,FGFR3,IRF4,IRS1,KDM5A,MAP3K13,MET,MST1R,NF1,PAK7,PMAIP1,PRDM1,PTPRS,RARA,SDHAF2,SOX9,TAP1,TGFBR1	0.09186804	1.293401957
path_508	BRAF,BTK,CTNNB1,DDR2,DIS3,HIST1H3B,INSR,MSH2,MUTYH,NTRK3,PARP1,PBRM1,PDGFRB,PGR,PIK3CD,PPM1D,PTEN,RAD21,TET1,TET2	0.111949523	1.297595788
path_509	BBC3,BMPR1A,CD276,CDK12,CSF1R,ERBB2,ERG,FAM46C,JAK1,MUTYH,NRAS,NUP93,PARK2,PDGFRA,PDGFRB,PIK3R1,PPP6C,RHEB,SOX9,SPOP	0.675003088	1.0623419
path_510	AXIN1,BCL10,BRAF,EPHA7,ERRFI1,FGF4,FLT4,FOXP1,GNAS,HIST1H3E,HLA-A,MAP2K1,MAP3K1,MED12,NOTCH1,PAK1,PIK3CB,PIK3R2,RET,SOCS1	0.967179968	0.99264449
path_511	BRCA2,CTCF,EIF1AX,ERCC5,H3F3C,HGF,HIST3H3,INHBA,MALT1,NRAS,PIK3CD,PMS1,PPP2R1A,RAD54L,RBM10,RNF43,ROS1,RUNX1,TCF3,TEK	0.087777007	1.344541502
path_512	ASXL1,BCL2L1,CARM1,DIS3,DNMT1,EPHA5,INPP4B,IRS2,KLF4,LATS1,MAP3K13,MEF2B,NUF2,NUP93,PLCG2,RTEL1,SMAD2,SOX17,SRC,STAG2	0.598813077	1.077142305
path_513	B2M,CARD11,CTLA4,E2F3,ELF3,EPHA7,EPHB1,ERBB3,ERBB4,FANCC,GSK3B,HIST1H1C,INPP4B,KEAP1,MYC,PIK3CD,PMS1,SMAD3,SMO,SOX9	0.060234957	1.342684443
path_514	BARD1,BRCA1,CASP8,CDK12,ERBB2,ERCC2,EZH2,GRIN2A,HIST1H3I,JUN,MEN1,MLL,PARK2,PIK3CD,PMS2,RAD51D,RFWD2,SHQ1,SMO,TBX3	0.671647388	1.07980214
path_515	CSF1R,CTLA4,DNMT3A,DROSHA,GNA11,LATS2,MAP2K1,MAP2K2,MST1R,NBN,NF2,NFKBIA,NTRK3,PIK3R3,PLK2,PTCH1,RAC2,RAD51D,RPTOR,SF3B1	0.683454458	0.930914589
path_516	ACVR1,ARID1A,ATM,CDK4,CSF3R,GSK3B,INHA,JUN,KNSTRN,MALT1,MDM4,MITF,PHOX2B,PIK3CB,RAC2,RAD51B,REL,RFWD2,SETD2,SMAD4	0.167730013	1.273302148
path_517	AMER1,ARID2,BCL2,BTK,CHEK1,IGF2,INSRR,KDM5A,KDM6A,KIT,MEF2B,MGA,MLL2,MSH2,PALB2,PARP1,SDHA,STAT3,TGFBR1,TP53BP1	0.064644893	1.363161377
path_518	ALK,ERBB4,GATA2,GSK3B,HIST3H3,HNF1A,KLF4,MALT1,MDM4,MEF2B,MYCL1,NF2,PAK7,PARK2,PDGFRA,PHOX2B,PMAIP1,SMARCA4,SRC,TEK	0.07825818	1.324479324
path_519	AMER1,AURKA,BCL2L1,BCL2L11,BRCA1,CXCR4,E2F3,ESR1,FAM46C,FGF4,IKBKE,INPP4B,KDM6A,MDM2,PBRM1,PIM1,PREX2,RHEB,SRC,STAT3	0.193342301	1.21005145
path_520	CRLF2,CYSLTR2,DNMT3A,DROSHA,EIF4A2,ERBB4,ETV1,FAM175A,FGFR2,IGF2,INPP4A,INSR,MAP3K13,MCL1,MST1R,MYC,NCOA3,NEGR1,PLCG2,RASA1	0.2282059	1.185146967

path_521	BMPR1A,CCND3,CD274,CHEK2,DDR2,DIS3,GRIN2A,IGF1R,MEF2B,MST1,NRAS,NSD1,PIK3C3,PNRC1,PTPRD,RAD51C,RPTOR,TGFBR1,TOP1,TRAF7	0.325165877	1.173891459
path_522	AXL,BRCA1,CREBBP,CYSLTR2,DNMT3B,ELF3,EP300,FH,FLT1,HIST1H1C,HIST1H3E,IKBKE,KMT2A,MAX,MDC1,PIK3R2,RYBP,SDHB,SMAD2,SRSF2	0.102438224	0.771430538
path_523	BCL10,CCNE1,CD79B,CDKN1B,CIC,DROSHA,EPHB1,ERCC4,FOXA1,FOXO1,GATA2,GATA3,HIST1H3B,HIST3H3,INSRR,KMT2A,REL,RHEB,RPS6KB2,SOS1	0.294749921	1.182706057
path_524	ANKRD11,AXL,BRD4,CCND1,CD79B,CDC42,CDKN1A,CDKN2A,CTNNB1,EIF1AX,ERRFI1,FAT1,FUBP1,GATA3,JAK3,PAK7,PAX5,PIK3CD,PTPN11,SMARCD1	0.207920833	1.220710769
path_525	ANKRD11,B2M,BCOR,BRCA2,CCNE1,CDKN1B,CIC,DROSHA,EIF4A2,FGF3,FYN,INHA,JUN,MLL,NCOR1,NEGR1,NOTCH3,RAD54L,SLX4,SOX2	0.055720479	1.312573736
path_526	CASP8,CHEK2,CUL3,DICER1,EPHA3,FANCA,GNA11,HIST1H3J,HIST3H3,IRS2,KMT2A,MAP2K1,MDC1,MITF,MYC,PDGFRB,RB1,RBM10,RYBP,SMARCB1	0.167597796	1.241959069
path_527	ARAF,ARID5B,ASXL1,BARD1,CCND2,CDKN1B,DNMT1,FANCC,HIST1H3H,HIST3H3,IFNGR1,IL7R,KMT2D,MDM2,POLE,PPP2R1A,SMARCA4,SRC,STK11,TNFAIP3	0.520012858	1.101337812
path_528	BRCA1,CSF3R,EIF4E,ERBB3,FH,FOXL2,H3F3C,INSR,IRS2,MGA,MUTYH,NTRK3,PDCD1,PIK3R3,PMS1,RASA1,RFWD2,ROS1,SMAD4,SPEN	0.96779324	0.993183148
path_529	ACVR1,ARID1B,AXIN1,BRD4,CSF3R,CTCF,ERBB2,ERCC2,H3F3C,HIST3H3,IGF2,INPP4B,NF2,NFE2L2,PMS2,PTCH1,RAD21,RAD51B,SH2B3,TBX3	0.540598282	1.090536166
path_530	ATRX,BRIP1,CDK4,CRLF2,EED,ERBB3,FANCC,HIST1H3A,ICOSLG,IRF4,MCL1,NUP93,PARK2,PIK3C2G,PNRC1,PTPN11,RAD50,SRC,STK11,WT1	0.236654614	1.204196531
path_531	AXIN2,BRCA1,BTK,CRLF2,FGF3,FGFR2,FLT1,GATA3,GNAQ,HIST1H3I,HNF1A,IL10,KDR,NKX3-1,NTRK2,PAK1,PAK7,PTEN,RHOA,SMAD4	0.670315249	1.071863704
path_532	APC,ARID1A,ASXL2,CDH1,EGFR,GATA2,H3F3C,HIST1H3B,HIST1H3J,INSR,MAP2K1,MTOR,NCOR1,NEGR1,NOTCH3,PBRM1,RASA1,STAT3,TERT,TGFBR1	0.892767218	0.979475971
path_533	ALK,ASXL1,ASXL2,BLM,CBL,EPHA7,FLT1,FYN,GNA11,HIST1H3H,HIST1H3J,KNSTRN,MDM2,MST1,MYOD1,PAK1,PHOX2B,RAD51C,STK11,TET1	0.349310623	1.158292875
path_534	AKT1,AKT2,CHEK1,ERBB2,FOXL2,GATA1,GNA11,HIST1H3A,IRF4,KDM5C,MAP2K1,MRE11A,MTOR,MUTYH,NKX3-1,PMAIP1,PPP2R1A,SOS1,SOX17,STK11	0.697600267	1.065982872

path_535	AR,CCND2,CDK8,CHEK2,ERBB2,ERG,FH,FYN,GRIN2A,H3F3C,HIST1H3H,MCL1,MDC1,MEN1,NEGR1,NOTCH1,NTRK1,NUF2,PTPRS,RUNX1	0.641150289	1.06827542
path_536	ARAF,BRIP1,CARM1,CUL3,EGFR,EPHA5,ERBB4,FYN,GSK3B,IRF4,JUN,MITF,NFKBIA,NOTCH3,PIK3CA,RAD51D,RECQL4,SMAD3,TP53,TRAF2	0.074847776	1.328941501
path_537	APC,ATM,BMPR1A,CARM1,EPHA3,ERCC5,FGF4,FGFR2,GSK3B,IL10,MAP3K13,MUTYH,NCOA3,NTRK1,PIK3CD,PIK3R2,PRDM1,SDHC,SH2D1A,SMO	0.13233479	1.286646207
path_538	ARAF,BMPR1A,BRAF,EGFR,EPHB1,ETV1,FANCC,FGF4,HOXB13,KNSTRN,LATS2,MRE11A,NF1,PAX5,PLCG2,PTPRT,SETD2,STAT5B,SYK,TSC1	0.136673328	1.246181851
path_539	ABL1,AKT1,APC,ARID5B,B2M,EP300,EPHB1,ERCC4,FUBP1,HIST1H3C,KIT,LATS1,MED12,MYCN,NOTCH3,NRAS,PBRM1,PREX2,SMARCB1,SPOP	0.2265299	1.241698543
path_540	BRD4,CCND1,CDKN1A,CSF1R,DDR2,ETV6,FANCA,GPS2,IGF1R,IRF4,KLF4,MLL3,NFE2L2,NOTCH2,PHOX2B,PIM1,PMAIP1,RAC2,RB1,RYBP	0.768317419	0.950470039
path_541	APC,ASXL1,CCND2,EGFR,EIF4E,FOXP1,H3F3C,IL10,INHBA,LATS1,MEN1,MLL2,MSH6,PNRC1,PTCH1,RFWD2,SOX17,SOX2,STAG2,TET1	0.782004157	1.04510648
path_542	AXIN1,BCL10,BCL6,CD276,CDK4,CTCF,DNMT3B,E2F3,FGF4,FOXP1,H3F3C,IRS2,MUTYH,NKX2-1,NOTCH1,NUP93,PARP1,PPP2R1A,RAD51,RNF43	0.22567045	1.187559352
path_543	BCL6,BTK,CDK12,FGF3,FH,H3F3C,IDH1,IL7R,INHA,MAP2K4,MAP3K1,MDM4,MTOR,NCOR1,NTRK3,PIK3CA,PIK3CB,RB1,RHOA,TET1	0.31498067	1.184135054
path_544	ARID5B,BCL2,BCL6,DAXX,E2F3,EIF4A2,FGF3,FLT3,HIST1H3I,HIST1H3J,IGF2,JAK1,MRE11A,MSH6,PAK1,PIK3C3,PIM1,RARA,RET,ROS1	0.282018858	1.164899809
path_545	BCL2L1,CDKN1A,CENPA,CREBBP,CSF3R,ERBB3,ETV1,EZH2,FOXL2,HIST1H3B,HIST1H3H,IRS1,MSH3,NF1,NF2,ROS1,SMAD4,SOCS1,TCF3,TNFAIP3	0.130196354	1.330092796
path_546	APC,AR,CCND1,CENPA,EP300,ESR1,FANCA,FOXL2,GPS2,IRS2,JAK1,KDM5A,MCL1,MLL2,MYCL1,NKX3-1,PLK2,PMS2,SRC,TP63	0.948734858	0.989600166
path_547	BAP1,BCL2L1,CDC42,CDC73,CENPA,ERBB4,FAT1,GNA11,KIT,MSH2,NSD1,NUP93,PIK3C3,PIK3R3,PTPRT,RET,RFWD2,RHEB,SF3B1,TGFBR1	0.313066218	1.170679337
path_548	AKT3,ALOX12B,CARD11,CHEK2,CTNNB1,EED,EGFR,ERG,FLT1,IL7R,INHA,KIT,KMT2A,MDM4,NTRK2,PTEN,RYBP,SMO,SRC,TGFBR1	0.834907492	1.034247369

path_549	ASXL2,CCND1,CDK12,CTNNB1,ETV1,FOXA1,GRIN2A,HGF,IL7R,JAK2,MALT1,NRAS,PALB2,PDCD1,PIK3C2G,SOCS1,SOX9,STAT3,TCF7L2,TET2	0.579462097	1.09708924
path_550	ALOX12B,ATM,BMPR1A,CDC73,CREBBP,CRLF2,EP300,ERBB4,ERCC5,ETV1,FH,FLT3,GNAS,HIST1H3I,JUN,NCOA3,NEGR1,SDHAF2,SETD2,SOS1	0.496242014	1.124561389
path_551	ALK,AMER1,APC,AXL,CDH1,CDKN1B,CDKN2C,DDR2,EIF4A2,EPHA7,ERBB3,ERCC2,ETV6,ICOSLG,MITF,NSD1,PHOX2B,PTPRT,REL,ROS1	0.195451073	1.241083093
path_552	CARM1,CDK8,CRLF2,CUL3,DOT1L,EP300,ETV6,FGFR3,FUBP1,GPS2,IKBKE,MTOR,MUTYH,NBN,NCOR1,NF1,NFKBIA,PDGFRA,TAP2,TERT	0.340525718	1.18236229
path_553	B2M,CDK6,CDKN1B,DICER1,DROSHA,ELF3,EPHA3,HGF,HIST1H1C,KMT2A,MITF,NF1,PDCD1,PMAIP1,PMS1,RAD51B,REL,SOX17,SOX2,STAT3	0.678131861	1.07031096
path_554	AKT3,AXL,CBL,CCNE1,CDC73,CDK6,DROSHA,ELF3,ERCC4,FBXW7,FLT3,FOXL2,KDM5A,LATS2,MLL,NOTCH2,PIK3R2,PMS2,PPM1D,RAD51	0.691585125	1.058213584
path_555	ANKRD11,BTK,CCND1,CDKN1B,DOT1L,ERCC4,ERCC5,EZH2,FGF4,FLT4,FOXL2,HGF,HIST1H3A,IGF2,INHA,KDM6A,MAP2K1,MYOD1,PAK7,RPS6KB2	0.535276425	0.897657877
path_556	ALK,ARID1A,ARID5B,BCL2L11,CDK8,CDKN1A,EIF1AX,EPHA7,FOXL2,HIST1H3E,IL10,INSR,KEAP1,LATS2,MAX,NF1,NF2,NFKBIA,POLD1,RAD21	0.395347877	1.128890373
path_557	ACVR1,ASXL1,BCL2L1,CCND2,CD276,FAM175A,GNAQ,GSK3B,IGF1R,KRAS,MEF2B,MLL2,MPL,PIK3CA,PIK3CB,RAF1,RTEL1,SDHAF2,SOX9,STAG2	0.466494073	1.124688269
path_558	CARM1,CCND1,CDH1,CDK12,CDK4,CDKN2A,CHEK2,CREBBP,CYSLTR2,EGFR,ERG,EZH2,FGF3,INPP4B,MAX,MST1,NCOR1,PNRC1,SDHB,XPO1	0.56171155	1.104475929
path_559	BBC3,BRCA2,CDK12,CEBPA,CENPA,E2F3,ELF3,FUBP1,GATA1,IDH1,IRS2,MST1,NUP93,PMS1,RAD51C,SF3B1,SPEN,TBX3,TRAF2,WT1	0.16207073	1.225458472
path_560	AMER1,ARAF,AXIN2,BRCA2,CDKN2A,CIC,CSF3R,DICER1,DIS3,FGF4,INPP4A,IRS2,KDM6A,MALT1,NFKBIA,NOTCH3,PALB2,RAD54L,RB1,RNF43	0.717291997	0.939459074
path_561	B2M,BCL6,EPHA7,ERBB3,FLT4,GATA1,HIST1H1C,KMT2A,MALT1,NKX2-1,NTRK2,PIK3CG,PTPRT,RAD51B,RARA,SDHA,SMARCD1,SMO,SYK,TET2	0.131813977	1.272556834
path_562	AURKA,BCL10,DOT1L,EPHA5,FGFR1,GLI1,HGF,HIST1H1C,IRS1,JAK1,MALT1,PARK2,PIK3C3,PTCH1,SDHA,SDHB,SPEN,TAP1,U2AF1,WT1	0.145807441	1.258833103

path_563	ARID2,BLM,CHEK2,EED,EIF4A2,FGFR1,GATA3,HGF,ICOSLG,MLL,MLL3,NKX3-1,NOTCH1,NRAS,PALB2,PIK3CG,PLCG2,SETD2,SMAD4,SMARCA4	0.425841253	1.119894742
path_564	CARM1,CEBPA,CHEK1,EED,FGFR3,FLT3,FOXO1,FUBP1,HGF,IL7R,JAK1,MAP2K4,MITF,NBN,PHOX2B,PMAIP1,POLE,RAC2,SLX4,SOS1	0.490004561	1.105439849
path_565	BMPR1A,CDKN1B,CIC,DNAJB1,EIF4A2,FAT1,ICOSLG,INPP4B,MDM2,NOTCH2,PTCH1,RPS6KB2,SH2B3,SOCS1,SOX17,STK11,TOP1,TP53BP1,TP63,TSC2	0.423449519	1.134382617
path_566	AKT3,ALK,BRAF,CDK12,CXCR4,DDR2,DICER1,ESR1,GATA1,HIST1H3E,IFNGR1,IL7R,KDM5C,KRAS,PPM1D,PRDM1,PTPN11,RAD21,RAD51C,SHQ1	0.720631748	1.054541799
path_567	ATR,BAP1,BMPR1A,CALR,CARM1,CD79B,CTLA4,DROSHA,EPHB1,FAM175A,FAT1,FGFR4,H3F3C,HIST1H1C,HIST3H3,LATS1,MEF2B,MUTYH,PDCD1,STAT3	0.082900361	1.279006509
path_568	ARID1B,B2M,BCL10,CTCF,CUL3,FANCC,GNAQ,HGF,HOXB13,IL7R,INSRR,MDM2,MED12,PALB2,PMS1,RHEB,SRC,TET2,TP53,TRAF2	0.270218372	1.173024159
path_569	BCL2L11,CCND2,CDKN2A,DNMT1,DROSHA,EIF4E,ERCC2,ESR1,GATA2,HNF1A,IL10,INHA,KEAP1,KLF4,KMT2A,NBN,NRAS,PIK3R2,PTPRS,STAG2	0.361109922	1.149810692
path_570	AMER1,ATM,AXIN2,CCNE1,CD276,CYSLTR2,E2F3,EGFR,ERBB2,FANCA,HIST1H1C,HIST1H3J,HIST3H3,IRF4,MUTYH,PAK1,PDGFRA,RASA1,RHEB,TERT	0.863447042	1.028006806
path_571	AKT2,AR,BARD1,BCL2L1,CSF3R,CTNNB1,FGF3,GATA3,IRS1,KDR,MCL1,MPL,MYOD1,NFKBIA,NOTCH4,PIK3R3,PMS2,RASA1,SRSF2,STK40	0.20694155	1.272803311
path_572	ARID1B,ATM,AXIN2,BRIP1,CALR,CD276,ERCC2,ERCC4,FGF4,FLT4,HIST1H3B,HIST1H3D,MAX,NCOR1,RHOA,SH2B3,SMARCD1,STAT3,STAT5B,TOP1	0.983679884	1.003753563
path_573	ANKRD11,CHEK2,DDR2,DROSHA,EP300,FANCC,FGFR2,GATA3,IFNGR1,KDM6A,KNSTRN,MAP3K1,MAP3K13,MEF2B,MGA,MSH3,MYOD1,NSD1,PIK3CA,PTPRS	0.994739599	0.999067979
path_574	ACVR1,ATR,CSF1R,CTCF,DNAJB1,ERCC2,FGFR4,HIST1H3I,HIST3H3,IL7R,KEAP1,MAX,NKX2-1,PHOX2B,PLCG2,PNRC1,RPTOR,SDHB,SMARCB1,WT1	0.892749251	1.022720474
path_575	ASXL1,ATM,CTCF,EPHA7,ETV1,ETV6,IKBKE,IL7R,INSRR,JAK1,MAX,PARK2,PDGFRB,PLK2,RAD21,RUNX1,SDHAF2,SHQ1,SPEN,SPOP	0.519686099	1.095110922
path_576	BCL2L11,BMPR1A,BRAF,CCND1,CHEK1,EIF1AX,ERBB2,FOXA1,INSR,JAK2,MALT1,MGA,NCOR1,NFKBIA,NRAS,PAK7,PMAIP1,PNRC1,SDHB,SOX2	0.752268779	1.058125462

path_577	AURKA,BCL2,BTK,DDR2,ERCC4,FAM175A,FGFR2,FOXP1,GNAQ,HIST1H3J,HOXB13,IL7R,MED12,NF1,NRAS,NTRK2,PBRM1,PTPN11,RAD51B,RAD51C	0.133205211	1.31057427
path_578	ASXL1,CALR,CCND2,CRLF2,DNAJB1,ESR1,FGFR4,FLT3,MAP3K1,NCOA3,NSD1,PDCD1,PIK3CD,PPM1D,RAD21,RAD51B,RAF1,RUNX1,SOCS1,STAT5B	0.454153588	1.115352768
path_579	AXIN1,BAP1,BRCA1,DROSHA,ERBB2,ERG,FGF19,GNA11,H3F3C,IRS2,MSH3,NFE2L2,PIK3R3,PNRC1,POLD1,RASA1,REL,RHEB,RICTOR,SDHA	0.474264263	1.109389218
path_580	AKT2,CDC73,CDK4,EED,ERCC4,ERG,HIST1H3E,HOXB13,LATS2,MUTYH,NFE2L2,PAK7,PDCD1,PTPRS,RAD51C,SDHC,TBX3,TCF7L2,TP53BP1,TSC2	0.531242971	1.092991354
path_581	ACVR1,ASXL1,AURKA,AXL,CCND3,CDKN1B,CTCF,CTLA4,DOT1L,FGFR3,HIST1H3G,IGF2,IRF4,KDR,MSH6,MYOD1,PMS2,RAD51C,RFWD2,SETD2	0.621657046	1.072368478
path_582	AKT2,ARID1B,BCL2L11,CDKN2A,CHEK2,EIF4A2,FOXA1,HIST1H3A,IDH1,INPP4B,IRS2,JAK1,MEN1,MLL2,NCOA3,NKX3-1,PIK3R1,PTPRD,RPTOR,SETD2	0.158586767	1.246713891
path_583	ALK,ASXL2,AURKA,BCL2,CARM1,CCND2,CRLF2,CSF1R,EED,FGF4,INSRR,MAX,MST1R,NFKBIA,NOTCH3,PDGFRA,PGR,PIK3CD,PMS2,SOX2	0.15524315	1.222482919
path_584	ACVR1,AKT1,BCL10,CHEK2,DAXX,DROSHA,GNAQ,GNAS,HGF,ICOSLG,INPP4B,MAP2K1,MAP3K1,MITF,MTOR,PARP1,PHOX2B,RASA1,REL,SYK	0.186304645	1.2061627
path_585	APC,BCL2L11,CCND1,DROSHA,EIF4E,FYN,GNAQ,KNSTRN,MEN1,MLL2,MRE11A,NCOR1,NKX3-1,PIK3R1,RFWD2,RPS6KA4,SDHB,SMAD3,SPEN,SPOP	0.814247582	1.033892717
path_586	APC,AXIN2,BMPR1A,CD274,CDK8,CTNNB1,DNMT3A,ERBB3,ETV6,FBXW7,HIST1H3I,ICOSLG,LATS1,MAP2K2,MET,NFKBIA,PNRC1,REL,SRC,STK40	0.48914527	1.130508172
path_587	ARID5B,BRD4,CCND3,CDKN2A,CXCR4,DROSHA,EIF4E,ETV1,FH,FOXO1,IGF1R,KLF4,MAP3K13,NCOR1,NF1,PAK7,RAD50,SMAD2,SRC,SUFU	0.113014293	1.323081032
path_588	ATRX,BARD1,BCOR,CDK8,CENPA,EGFR,EIF1AX,ERCC4,GNA11,GRIN2A,IGF1R,INPP4B,MAP2K1,MAP2K4,MAP3K1,MET,NPM1,PARP1,RAD21,TCF7L2	0.139795848	1.279261416
path_589	ARID1A,AXIN2,B2M,CASP8,CCNE1,CYSLTR2,DDR2,E2F3,EIF4A2,FGFR3,FLT4,HIST1H3E,IKZF1,JAK2,KEAP1,MAP3K1,MUTYH,PIK3R2,SOCS1,STAT3	0.051693127	1.351241934
path_590	FGFR1,GNAQ,KLF4,MAP3K13,MET,MSH3,MTOR,PARK2,PGR,PIK3C2G,PIK3CG,PMAIP1,PTCH1,PTEN,RBM10,RFWD2,RHOA,SF3B1,SPEN,TAP1	0.157736858	1.247275564

path_591	ASXL1,BARD1,CARD11,CHEK1,EED,FGF3,FGFR3,FH,H3F3C,KLF4,MAP3K13,MST1,MUTYH,NFKBIA,RAD21,RICTOR,RPS6KA4,SOX17,SPOP,TOP1	0.315600294	1.154140536
path_592	BCL2L1,BRCA2,BRIP1,CDKN2A,CXCR4,DAXX,EIF1AX,EIF4E,FGFR4,FOXP1,GATA3,MDM2,MYC,NCOR1,NTRK1,NTRK3,RPS6KA4,RUNX1,SDHB,TAP2	0.06117515	1.364582742
path_593	ANKRD11,BAP1,CCND2,CDC42,CDKN2A,CUL3,FGFR3,HIST3H3,IDH1,MSH6,NRAS,PIK3CB,PNRC1,PPP6C,REL,RPTOR,SH2B3,SMARCB1,TGFBR2,TP63	0.689068052	1.058309067
path_594	DICER1,EIF1AX,EPHB1,ETV1,FGF4,FGFR2,FOXL2,KDM6A,MAP2K2,MAP3K1,MLL3,MST1,NF1,NKX2-1,PIK3C3,RAD54L,RHOA,RUNX1,SETD2,STAG2	0.376922491	1.144708273
path_595	ALOX12B,ARAF,ARID2,BARD1,BMPR1A,CCND3,CDK6,CSF1R,ERBB3,ERRFI1,INPP4A,IRS1,NKX2-1,PIK3R3,PLK2,PPM1D,RHOA,SH2D1A,TET2,TSC1	0.637924356	1.095622688
path_596	ATM,CDKN1A,EPHA7,ERBB3,ETV6,FGFR2,FOXL2,GRIN2A,HOXB13,IGF2,INSRR,KMT2A,MAP3K13,MST1R,NCOR1,PMS1,POLE,SDHAF2,SDHB,SOS1	0.738773909	1.059752563
path_597	AKT1,BCOR,CALR,CDC73,CENPA,CHEK1,DDR2,EPHA5,HIST1H3D,HIST3H3,IRS1,KEAP1,MED12,RAF1,RNF43,RUNX1,SPEN,TET1,TRAF7,YES1	0.150553062	1.243655727
path_598	AKT3,BCL2,BMPR1A,CCNE1,ERRFI1,GATA3,GLI1,GNA11,HOXB13,KLF4,KMT2D,MAP2K2,MCL1,MSH3,MYCN,NCOA3,RFWD2,RNF43,SRC,TOP1	0.580224542	1.083732974
path_599	BRCA1,CASP8,CDK8,CSF1R,DIS3,FOXP1,GATA3,KDM5C,KMT2A,MCL1,MTOR,MYC,NCOR1,NF2,NKX2-1,NTRK1,PAK1,PIK3R1,RBM10,SMARCA4	0.076815127	1.303263675
path_600	CDK8,DNMT1,EPHA5,FH,IFNGR1,JAK1,KDR,MTOR,NFKBIA,PARP1,PIK3CB,PPP2R1A,PRDM1,RAD51C,RARA,SPEN,SRSF2,STK11,TGFBR2,TOP1	0.25383028	1.217495927
path_601	ABL1,ACVR1,CTCF,CTLA4,DAXX,H3F3C,HIST1H3G,HOXB13,INHBA,KDR,KRAS,NOTCH4,PIK3C3,PIK3R2,POLD1,PREX2,RBM10,RHEB,SMARCD1,SMO	0.898351077	0.982079362
path_602	ALK,ARAF,BAP1,BCL2,BCL2L1,CDKN2C,DNAJB1,EED,ERBB2,FGF4,FGFR3,GATA2,HIST1H3H,MPL,PALB2,POLE,RHEB,SDHC,SOX9,TEK	0.818960793	1.033177201
path_603	AKT1,AR,AXIN2,BRAF,CCND1,EPHA3,ERCC4,FH,INHA,KLF4,MDC1,NTRK3,PLK2,RAD51D,REL,RNF43,SOCS1,STK40,TNFAIP3,TRAF2	0.134005771	1.314779856
path_604	AURKA,CALR,CTNNB1,ERRFI1,FGFR2,FLT1,HNF1A,KDR,MDC1,MDM2,MDM4,MEN1,NSD1,NTRK2,PARP1,PAX5,PHOX2B,PIK3R1,PIK3R2,RAD50	0.332208834	1.203703593

path_605	AXIN2,CARM1,CCND1,CDKN2A,CHEK2,ERBB3,ETV1,ETV6,FH,FOXL2,FYN,GNA11,HIST1H3C,INSR,KDM6A,MAX,PIM1,RAD21,RAD51B,RB1	0.986362949	0.996543967
path_606	ACVR1,AXL,BCL2L1,BCL2L11,CD274,CREBBP,DNMT3B,EP300,FOXP1,HIST1H3B,JUN,KDR,KMT2A,NF1,PIK3R2,PPP2R1A,PREX2,RAD51C,RHOA,SOX9	0.630130488	1.091924838
path_607	ANKRD11,ATR,CASP8,CHEK1,CIC,CYSLTR2,FGF19,FGF4,FOXA1,GATA2,GATA3,JAK2,KDM5A,MLL,NF2,PIK3CB,RAD50,RAF1,SETD2,SPOP	0.12480357	1.244721897
path_608	CDK4,CIC,CSF3R,CTCF,FOXL2,FOXO1,GLI1,IRF4,KDM5A,KDM5C,KIT,MEF2B,MSH2,NCOR1,PAX5,RAD51,RAD51D,RHOA,SETD2,TAP2	0.474531863	1.147050442
path_609	BCL2L1,CDK6,CSF1R,EIF4A2,EIF4E,EP300,HLA-A,KIT,MSH6,NOTCH2,PDGFRA,PIK3R3,SMAD4,SRC,STK40,SUFU,TCF7L2,TMPRSS2,TSC1,XPO1	0.616413617	1.073360246
path_610	AXIN2,BBC3,BCOR,CALR,EIF4A2,FGF4,FLT4,FOXP1,HIST1H1C,IDH1,IL10,MYCN,NKX3-1,PDGFRA,PMAIP1,RNF43,RPTOR,RYBP,SMARCA4,SMARCB1	0.357279781	1.163650407
path_611	AMER1,BTK,CD274,CSF3R,CTLA4,FAM175A,FGF19,FGFR1,IRS1,KLF4,KNSTRN,MDM2,MRE11A,PBRM1,PIK3CA,PLK2,POLE,RAD51D,RASA1,REL	0.406534206	1.125171882
path_612	AMER1,BRCA1,CCND3,DNMT1,EPHA5,HLA-A,KLF4,NBN,NCOR1,NTRK2,PAK7,PALB2,PRDM1,RARA,RNF43,SDHAF2,SH2B3,SMARCA4,SMARCB1,TP53BP1	0.449899946	1.123496866
path_613	ALK,ASXL2,CDKN2C,CSF3R,DOT1L,EIF4E,HOXB13,INPP4A,MDC1,MSH2,NCOA3,PHOX2B,PPM1D,RHOA,RICTOR,SETD2,SOX9,SRC,STAG2,TGFBR1	0.195723676	1.201071503
path_614	ARAF,ARID5B,CUL3,EGFR,ESR1,FGF4,GATA3,GRIN2A,HIST1H3E,HOXB13,IRS2,MAP3K1,MEN1,MST1,NEGR1,NOTCH2,PPP2R1A,ROS1,RPTOR,SOX17	0.791456587	1.046681775
path_615	ACVR1,AR,ARAF,AURKA,CARD11,CCNE1,CDKN2C,FGFR4,HGF,HIST1H3I,ICOSLG,MSH6,NUF2,RASA1,RPS6KB2,SDHB,SOX17,TAP1,TCF7L2,TEK	0.185767519	1.209495156
path_616	BRCA1,CD79B,CIC,FANCC,FUBP1,HIST1H3E,IKBKE,JAK2,LATS2,MAP2K1,MGA,NCOR1,NTRK2,PIK3R3,PMS1,RAD21,RB1,ROS1,SDHA,SRC	0.49139542	1.101920492
path_617	ABL1,AXIN1,CDK8,EPHA5,ERCC5,FANCC,FGFR1,GATA2,HIST1H3G,HNF1A,PARK2,PIK3C3,PLK2,PNRC1,PTPRD,RAC2,SDHB,SMAD3,STAT3,TEK	0.157735025	1.269799841
path_618	CBL,CIC,DICER1,DROSHA,EPHB1,ERCC4,FAM175A,GRIN2A,HLA-A,MUTYH,NF1,NKX2-1,PALB2,PDCD1,PNRC1,RAD51D,RHOA,RYBP,SMARCD1,TCF7L2	0.638281755	1.087069178

path_619	AKT2,APC,AURKA,CBL,CTNNB1,ERCC2,ERCC4,FBXW7,HIST1H3C,IFNGR1,IKZF1,INPP4A,PDGFRB,PLK2,PTPN11,RAD21,RAD51C,SOCS1,SOX2,TCF7L2	0.978219684	1.00396243
path_620	ABL1,AXIN1,BLM,BTK,CDK12,CDK8,FGF4,HNF1A,KDM5A,MET,MUTYH,MYCL1,NEGR1,PDGFRA,PDGFRB,RAD50,RAD51B,TERT,TGFBR2,TMPRSS2	0.144882425	1.230407706
path_621	ALK,AXL,CBL,CXCR4,E2F3,ERCC5,GRIN2A,HOXB13,IFNGR1,INPP4A,KNSTRN,MAP2K4,MUTYH,NPM1,NUF2,PIK3CA,SMARCD1,SOX9,TEK,TP53	0.548562997	1.092339741
path_622	ARID1A,ASXL1,B2M,BAP1,CDKN1B,CREBBP,FGFR4,GNAS,HIST1H3A,JAK3,KLF4,NFE2L2,PAK1,PREX2,RAD51C,RNF43,RUNX1,SDHB,SDHC,SH2D1A	0.311058698	1.192268377
path_623	ASXL2,CCND3,CXCR4,EGFR,GATA2,GSK3B,IGF2,INSR,JAK2,MAP3K1,MCL1,PARP1,PIK3R1,PPP2R1A,PTPRS,RAD51D,SDHB,SF3B1,TMPRSS2,TSC1	0.077814096	1.427098112
path_624	BMPR1A,CDH1,DAXX,EGFR,GRIN2A,H3F3C,IDH1,MLL,MST1,NOTCH1,PIK3R3,PLK2,PMS1,PPM1D,PPP6C,PTEN,PTPRD,RAD51D,RARA,REL	0.546709049	0.910679176
path_625	ACVR1,AKT1,ALOX12B,BARD1,CBL,CD274,CUL3,EGFR,EP300,ERBB2,ERCC4,GSK3B,HIST1H1C,HIST1H3D,MST1,PBRM1,RAD54L,RB1,SDHC,TGFBR2	0.969218846	0.99361728
path_626	ATM,ATR,BCL2,BCL2L11,CTLA4,ERCC4,ERRFI1,HIST1H3C,HNF1A,HOXB13,IDH1,MLL2,NF2,NOTCH1,NOTCH4,PDCD1,RAD50,RET,SMARCD1,TBX3	0.155897532	1.27186684
path_627	ATR,B2M,BCL2,BCL2L1,BCL6,DROSHA,EPHA3,FANCC,MGA,MYCN,NOTCH3,NRAS,NTRK2,PAK1,PARK2,PTEN,RAD51B,RPTOR,RUNX1,SUFU	0.056742296	1.369904314
path_628	ARID1B,ATM,BCL6,CYSLTR2,DIS3,EPHA3,EPHA5,EPHA7,FUBP1,IL10,KDM5C,KEAP1,MAP2K1,MLL,NTRK1,PARK2,PIK3CB,PIK3CD,RUNX1,TERT	0.124254939	1.2439034
path_629	AKT3,CHEK1,CHEK2,ERCC2,FBXW7,FGF4,FLT4,FOXA1,HIST3H3,MYCN,MYOD1,NCOA3,NOTCH2,PAK1,PGR,PIK3R3,PMAIP1,PREX2,PTCH1,RBM10	0.136419892	1.294840657
path_630	BCL10,CCND2,CRLF2,DNAJB1,EIF4E,FOXA1,HGF,HIST3H3,HNF1A,IDH1,JAK1,KDM5C,MSH3,NFE2L2,NKX2-1,PTCH1,RAC2,RFWD2,SPOP,TAP1	0.085121676	1.28061276
path_631	ARID1B,AURKA,BAP1,BCL2L1,ELF3,ERCC4,FH,GPS2,GRIN2A,HIST3H3,HOXB13,KDM5A,PMS2,RAD51B,RBM10,RICTOR,SHQ1,SMO,SOX17,TRAF2	0.99231964	1.001763961
path_632	CD274,DDR2,FANCC,FBXW7,FOXO1,GLI1,JAK1,MYCL1,PAK7,PGR,RARA,RICTOR,SDHB,SF3B1,SLX4,SOX9,SRSF2,STAT3,TCF7L2,TMPRSS2	0.281594294	1.16420327

path_633	AMER1,ATRX,CCND3,CCNE1,CD79B,CRLF2,ETV1,FANCC,GPS2,IL7R,KDM6A,KDR,MAP2K1,MDM4,MRE11A,NFE2L2,PIK3C2G,PLCG2,SDHB,TBX3	0.057119128	1.378244644
path_634	ACVR1,AXL,CD274,EGFR,FGFR2,IRF4,MAP3K13,MET,MUTYH,PARP1,PGR,PMS1,PREX2,PTCH1,ROS1,RPS6KB2,SLX4,SMAD2,SOX9,YES1	0.994005844	1.001332251
path_635	BCL2L11,BRCA1,BRD4,CDK6,CDKN2C,CSF1R,CSF3R,DDR2,DNMT3A,EIF4E,FGFR3,FOXO1,GNAS,INSRR,KDM5A,KDM5C,LATS2,MDC1,NRAS,SMAD3	0.963231642	1.006528126
path_636	AKT3,ARAF,AXIN2,BRCA2,CCNE1,CD79B,CSF1R,EZH2,IKBKE,KRAS,MSH2,MTOR,MYCL1,PTPRD,RHEB,RHOA,SDHB,SLX4,STK11,TNFAIP3	0.883091371	0.9795125
path_637	ABL1,ANKRD11,AR,ASXL2,BCOR,EP300,ETV6,EZH2,FGFR2,FYN,KIT,KMT2A,LATS2,MAP3K13,PARP1,PPP6C,SMARCD1,SMO,SYK,TAP1	0.405003195	1.157460329
path_638	ACVR1,AKT3,ALOX12B,ARAF,CDK4,CHEK2,DAXX,EIF1AX,GRIN2A,HGF,IDH1,INPP4B,KDM6A,KNSTRN,MEF2B,PBRM1,RASA1,SETD2,SMAD3,TP53BP1	0.788130322	0.956138157
path_639	ARID1B,ASXL2,CTCF,CTLA4,ERBB2,FLT1,HIST1H3H,KMT2A,KNSTRN,MDC1,MDM4,PARP1,PRDM1,SDHB,SHQ1,SLX4,SOCS1,SYK,TGFBR2,TOP1	0.576740954	1.082597501
path_640	AKT1,ALK,CARD11,ETV1,FGF3,HIST1H1C,INSRR,JAK2,JUN,KRAS,LATS2,MALT1,NKX2-1,PGR,PIK3CG,PIK3R1,RAD51,RICTOR,RPS6KA4,STAT3	0.306116602	1.158581249
path_641	BBC3,BMPR1A,BRCA2,CALR,CDK8,CEBPA,IKBKE,IL7R,MAP2K1,MEN1,MLL,MPL,PMAIP1,RAD51B,RASA1,SDHB,SDHC,SF3B1,TAP1,TEK	0.477086364	1.109306929
path_642	ANKRD11,ARID5B,BCL2,BRIP1,DAXX,ERBB3,FGFR2,HIST1H3H,INHBA,MITF,MRE11A,NPM1,PIK3CG,PIK3R1,RAD51C,RYBP,SDHAF2,STAT3,STAT5B,TCF3	0.151777469	1.229671498
path_643	ARID5B,BBC3,CDK6,CDK8,CDKN1A,DDR2,ERCC4,ERRFI1,GRIN2A,HIST1H3G,IKBKE,KDM5A,MAP3K1,NCOA3,NF2,PGR,PIK3CD,RAF1,RET,RNF43	0.053391839	1.314394304
path_644	ACVR1,APC,ARID5B,AXL,BMPR1A,BRCA2,CARD11,CHEK1,CTLA4,DICER1,DNMT1,FGFR2,HIST1H3H,MSH3,NEGR1,NF2,PIK3R2,POLD1,SMO,TP53BP1	0.24270645	1.179953505
path_645	ARAF,CDK8,DDR2,DNMT3B,ERG,FGF3,HIST1H3J,KDM6A,KNSTRN,MDC1,MDM2,MTOR,PALB2,PAX5,PLK2,RAD50,RPS6KB2,SDHA,SDHAF2,STAG2	0.15519556	1.254577663
path_646	ALOX12B,ASXL1,B2M,BCL2,CSF3R,EP300,FLT1,GNA11,HIST1H3J,INPP4B,LATS1,MITF,MYC,NOTCH1,NOTCH4,NUP93,PIK3C3,RPS6KA4,STAT3,SUFU	0.745672057	1.056615868

path_647	BRD4,CCND1,CCND2,CD274,CDK4,CHEK1,CRLF2,CSF1R,EPHA7,GLI1,HIST1H3C,KDR,NOTCH4,NSD1,PARK2,RBM10,RHEB,SRSF2,TAP1,TGFBR2	0.057442229	1.399344786
path_648	ALK,AMER1,CDH1,CEBPA,CHEK2,CXCR4,DNMT3A,EPHA3,ETV6,FOXA1,FOXL2,GLI1,HIST1H3E,HNF1A,IL7R,MST1,MYC,PIK3R1,ROS1,RUNX1	0.248525766	1.220060456
path_649	APC,BCL10,CALR,CDK6,CDKN1B,GSK3B,IDH1,JAK1,MAP3K1,MEN1,MPL,NSD1,PIK3CD,PPP2R1A,PTPRT,RAD50,RHEB,SMARCA4,SOCS1,SOX17	0.195776862	1.238232025
path_650	ABL1,AKT3,CD274,CSF3R,CTCF,FGF19,MALT1,MAP2K2,MLL3,MSH6,NCOA3,NFE2L2,PAK1,PBRM1,PGR,PIK3CD,RAD51C,RECQL4,RFWD2,SDHA	0.38087948	1.166555843
path_651	BRCA2,CDC42,ERCC2,FAM175A,FOXP1,GATA2,JAK2,MAP3K13,NEGR1,PDGFRB,PIK3R3,PMS1,POLD1,RBM10,RTEL1,SMO,SPEN,SUFU,TBX3,TET1	0.202292175	1.259501508
path_652	BARD1,CHEK2,CSF3R,CTNNB1,EGFR,EIF4A2,FLT1,HIST1H3H,IKBKE,LATS1,MSH2,MTOR,NCOR1,PALB2,PBRM1,PIK3R2,PTPRT,SMARCB1,TRAF7,YES1	0.654356512	1.069357248
path_653	APC,ATM,BRCA1,CDC42,CXCR4,DNMT3B,EPHA5,FBXW7,FGF3,FGFR2,GATA2,ICOSLG,IKBKE,IL7R,JAK2,NF1,PBRM1,PIM1,RAD21,STAT3	0.278186463	1.183870082
path_654	BBC3,BLM,CARM1,CBL,CD274,CD79B,DNMT3B,EIF4E,ERBB3,GRIN2A,IFNGR1,PMS1,PPP2R1A,PPP6C,RAC2,RARA,RB1,SRSF2,STAG2,STK11	0.625094659	1.085848414
path_655	ACVR1,ARID1A,ATRX,CARD11,DDR2,EZH2,FANCC,FYN,HIST3H3,HOXB13,IL10,MSH2,MST1R,NKX2-1,NTRK2,PPP2R1A,RPTOR,SDHA,SETD2,TOP1	0.074238903	1.341723125
path_656	AKT2,AKT3,BCL2L11,BMPR1A,CDK6,CDKN2C,DNMT1,EPHA3,EZH2,FLT1,GPS2,ICOSLG,KLF4,MAP2K2,NFKBIA,PIK3R2,RAD51B,RAD51C,RPS6KA4,SMAD3	0.122235921	1.243968149
path_657	BCL10,BTK,CD79B,CSF3R,CTLA4,EPHB1,ETV1,FAT1,FGFR1,FH,GRIN2A,MAP3K13,MDM4,MRE11A,NTRK1,PGR,RB1,RHOA,RPTOR,TCF7L2	0.443635323	1.134103676
path_658	AXIN1,BCL2,CDH1,CDK4,CHEK1,DNMT1,EGFR,FGF4,HIST1H3G,MUTYH,NCOA3,NSD1,NTRK1,PIK3CD,PNRC1,PREX2,RUNX1,SDHAF2,SH2D1A,STK40	0.724103604	0.951232478
path_659	ATM,BRIP1,CALR,CBL,CDC42,CDC73,CEBPA,CREBBP,CTCF,DNMT1,FGF4,HIST1H3A,HIST3H3,MGA,MUTYH,NKX2-1,PPP2R1A,RHEB,SETD2,TBX3	0.495681296	1.118809997
path_660	AKT1,ARID1B,BARD1,CDK8,CDKN1B,CDKN2A,CUL3,EIF1AX,ELF3,EZH2,FANCA,MDM2,MPL,MYCL1,NF1,RAD51,SDHA,SDHAF2,SMARCD1,SOCS1	0.08468966	1.273437071

path_661	AKT1,ALOX12B,B2M,BRCA1,CDKN1B,CENPA,CSF3R,DNMT1,FOXL2,IL10,KDM6A,MEF2B,MSH2,MTOR,NOTCH4,NTRK3,PIK3CD,PIK3CG,PTPRS,SPOP	0.276304071	1.166703233
path_662	ASXL2,ATRX,BRCA1,CD79B,CDC42,CSF1R,DICER1,DNMT3A,ETV1,FOXO1,HNF1A,MDC1,MSH3,MYOD1,NOTCH1,PTPRT,RAD54L,RNF43,TBX3,TEK	0.120858349	1.287674256
path_663	ARID1B,CDK4,EIF4A2,EIF4E,EPHB1,ERG,FH,HIST1H3E,HIST1H3H,IRS2,LATS1,MALT1,MAP3K1,MST1,MYCN,MYOD1,NUP93,RAD51D,SMAD3,STAG2	0.135577918	1.242805324
path_664	AKT2,ATM,ATRX,BRCA1,BRD4,CEBPA,IKBKE,JAK1,JAK2,KDM5A,MDC1,MGA,MITF,NFE2L2,NKX3-1,PAK7,PDCD1,PIK3R1,SMAD4,SMARCB1	0.880161953	1.023341956
path_665	BAP1,CARM1,CDC73,CDKN2C,CSF1R,DIS3,EIF4E,FGF4,HIST1H1C,ICOSLG,INHA,INSR,JAK2,MAP3K13,MEN1,NPM1,PMAIP1,PPP2R1A,RAD51D,YAP1	0.656944093	0.938273286
path_666	AURKA,BCL2L11,CDK4,CDK6,ETV1,FGF3,FGFR3,HIST1H3I,IL10,MED12,MST1R,MYOD1,NOTCH1,PIK3CA,PMAIP1,PPM1D,SOS1,SUFU,TET2,TP53	0.200479514	1.202331771
path_667	AURKA,CARD11,CDC42,DDR2,ETV1,FGFR1,FGFR2,FOXL2,IL7R,JAK2,MLL3,MPL,MSH2,NOTCH2,NUP93,SMARCD1,SOX17,TERT,TET2,TSC2	0.566901158	1.105728674
path_668	ARID1B,BCL2,E2F3,EIF4E,FGF3,FGFR3,FOXL2,FOXO1,GLI1,KNSTRN,MAP3K1,NEGR1,NSD1,PBRM1,PMAIP1,PMS2,PTPRS,RARA,RUNX1,SH2B3	0.978713067	1.003827728
path_669	CDKN2C,CEBPA,CYSLTR2,FANCA,FBXW7,INPP4B,IRS2,NFE2L2,PDGFRA,POLD1,PPP6C,PTPRS,RAD21,RAD51D,RAF1,RB1,SDHB,SLX4,SPOP,STAT5B	0.134656033	1.237899112
path_670	ATM,CDC73,DIS3,EIF4A2,EPHA3,FAM175A,HIST1H3J,IGF1R,JUN,MLL3,NCOA3,NFE2L2,PIK3R3,POLD1,PPP6C,PRDM1,RAD54L,RHOA,STK40,U2AF1	0.058240351	1.377610243
path_671	ATRX,B2M,BLM,FAT1,IDH1,INSR,KDM6A,LATS1,MAP3K13,MSH2,MSH6,NPM1,PDCD1,PMS2,PTPRD,RAD51,RAD51B,RHEB,SOS1,SPEN	0.102492621	1.291441074
path_672	AKT2,ARAF,BARD1,BCL2L1,BCOR,BRD4,CCND3,CD274,CENPA,EPHA3,ERBB2,ERBB3,HOXB13,INHBA,MPL,MST1R,NF1,NOTCH1,RAD50,RNF43	0.256412642	1.195827672
path_673	ARID1B,CASP8,CRLF2,CUL3,DOT1L,EPHA7,ERCC5,ESR1,FOXL2,IRF4,MLL,MRE11A,MST1R,NKX2-1,PALB2,PDCD1,RPTOR,SMARCB1,STAG2,TP53BP1	0.618925486	1.085956791
path_674	CCNE1,CDK8,DDR2,EGFR,ERRFI1,FANCC,FGFR2,FOXL2,HIST1H3B,IL7R,MET,MLL,MYC,MYCL1,PAK1,POLD1,PTEN,RHOA,STAT5B,TCF7L2	0.986493959	1.003036662

path_675	ARID2,BAP1,BCL2L1,CBL,CDK8,CHEK2,CSF1R,DIS3,DNMT3B,H3F3C,HGF,HIST1H3J,IRF4,NKX2-1,NKX3-1,NUF2,PHOX2B,PIK3C2G,SPEN,TAP1	0.130839583	1.285490291
path_676	ANKRD11,ASXL2,AXIN2,BRCA1,CCND1,CDKN1A,CHEK1,ERBB3,FOXP1,GNAQ,HOXB13,IKZF1,MAX,MGA,NF2,NUP93,PIK3R3,RB1,SH2B3,STK11	0.320290028	1.162422063
path_677	ATRX,AXL,BLM,CTCF,EIF1AX,EPHA3,FGF4,FLT4,HIST1H3A,IL10,INSR,KEAP1,MLL,PIK3R3,PTPRS,REL,SH2D1A,SMARCD1,SRSF2,TP53	0.126980484	1.270850173
path_678	ARID1B,ASXL1,CDH1,ERBB3,ERCC5,ETV1,H3F3C,INHA,IRF4,KEAP1,MEN1,NSD1,PDGFRA,PIK3R3,PTCH1,PTEN,PTPRD,RAC2,SDHAF2,SHQ1	0.38019276	1.141187648
path_679	ANKRD11,BARD1,CARM1,CXCR4,DICER1,FGF19,HGF,HIST1H3E,HIST3H3,KMT2D,MCL1,MDM4,NUP93,PDGFRA,RPS6KB2,TGFBR1,TOP1,TP53,TSC1,U2AF1	0.073606273	1.30765601
path_680	ACVR1,BAP1,BCL2L11,BMPR1A,BTK,CSF1R,CSF3R,EGFR,FGF3,FGFR3,GNAS,GSK3B,HIST1H1C,INPP4B,MDM4,NCOR1,NFE2L2,PMAIP1,PNRC1,SMAD4	0.197487648	1.232459891
path_681	ALOX12B,BRAF,CDKN1A,CENPA,ERCC5,EZH2,FANCA,FAT1,HIST1H3J,KNSTRN,MRE11A,MSH3,NTRK1,NUF2,PARK2,PBRM1,PIK3R3,PLCG2,RAF1,TSC2	0.061257772	1.429549377
path_682	CCNE1,CDC42,CTLA4,DAXX,EIF1AX,IFNGR1,INSR,KMT2A,MLL3,PAX5,PIK3R1,PIK3R3,PREX2,PTPRD,RASA1,REL,SDHAF2,SPEN,SRSF2,TP63	0.08210741	1.385363344
path_683	ATM,BCOR,CALR,CCND1,CDC42,CSF1R,CTLA4,FGFR1,FGFR2,FYN,GNA11,GNAS,GPS2,HOXB13,INPP4A,KDM5A,MITF,NKX2-1,PARP1,RAC2	0.637171733	0.935511466
path_684	AKT2,ALOX12B,CALR,CCND2,CDH1,EIF4E,ERRFI1,EZH2,FAM175A,GNAQ,HIST1H3A,IRF4,MYC,NOTCH2,NRAS,RAD50,RHEB,SH2B3,SUFU,TSC1	0.897897154	1.020121079
path_685	APC,B2M,BCL2L1,BCOR,CARM1,CCND3,FOXP1,JAK2,LATS1,MST1R,MYOD1,NCOR1,NTRK1,NTRK3,PIK3R2,SH2D1A,SMARCA4,TET1,TET2,TGFBR1	0.369642552	1.14483992
path_686	BCL10,BCOR,CBL,CCND2,CHEK2,ERCC2,IFNGR1,IL10,INSR,MALT1,MEF2B,PARK2,PARP1,RAD51,RUNX1,SDHC,SMARCD1,SOX9,STK40,YES1	0.398091989	1.130390699
path_687	APC,ASXL2,CDH1,CENPA,CREBBP,DICER1,EIF4E,ELF3,FOXO1,GRIN2A,GSK3B,JAK1,MPL,NOTCH2,NOTCH3,PDGFRB,PMAIP1,SHQ1,TEK,U2AF1	0.379288257	1.170007309
path_688	APC,ATRX,BAP1,BCL2L11,CCND1,CDC73,CHEK1,FGF19,IDH1,KDM5A,MLL2,MTOR,NKX3-1,NOTCH4,PARK2,RAD21,RET,SH2B3,SMARCB1,SPOP	0.262748053	1.205066915

path_689	ABL1,AKT3,CASP8,CDC42,CYSLTR2,EED,EIF1AX,FGFR3,IGF2,INHA,KDR,MUTYH,MYC,PAX5,PMS1,RAC2,RAD21,RAD51D,RET,SETD2	0.314777702	1.154289914
path_690	APC,ARID5B,BCL2L1,BCOR,BRD4,CEBPA,CENPA,EGFR,EP300,EZH2,FGFR3,HIST3H3,IGF1R,INPP4A,KIT,MCL1,PDCD1,STAT5B,STK40,TSC2	0.1216405	1.330627765
path_691	AKT3,CDC73,CDK6,ESR1,GNA11,GPS2,HGF,HNF1A,IKZF1,MAP3K13,PIK3CA,PLK2,PMS1,RAD50,ROS1,RPTOR,SDHAF2,SF3B1,STK11,SUFU	0.983414642	1.003214401
path_692	BCL2,BRD4,DNMT1,EED,EPHA5,ERBB2,FGF3,FGFR1,FOXP1,GATA1,GPS2,INHBA,KNSTRN,MDM2,MRE11A,RFWD2,SMAD3,SPOP,TBX3,TSC1	0.09116024	1.36230353
path_693	AURKA,AXIN2,EIF4E,FBXW7,FOXP1,HIST1H3G,IDH1,IKBKE,IL10,IL7R,MAP2K4,MDM2,MUTYH,MYCL1,MYOD1,PALB2,PIK3CG,RAD54L,SMAD4,SMO	0.944727732	1.010480353
path_694	ACVR1,BRIP1,CASP8,CDH1,CYSLTR2,EGFR,ERBB3,ERCC5,FGF3,HOXB13,MAP3K1,MAX,MDM2,MYCN,PDGFRB,PIM1,RAD54L,SH2B3,SRC,STAT5B	0.053306424	1.406551981
path_695	AMER1,AXIN2,BRIP1,CCND2,CDK12,CSF3R,CTNNB1,CXCR4,EGFR,ERCC4,FGFR1,FYN,HGF,HLA-A,INPP4B,LATS2,MDC1,MDM4,SDHA,SOS1	0.130195712	1.289556641
path_696	ANKRD11,ATR,BMPR1A,BTK,EPHA7,ERBB4,FAT1,FGF3,FH,FUBP1,FYN,JAK2,MRE11A,NFKBIA,PIK3C3,PIK3R2,PTPN11,RNF43,SH2B3,TRAF2	0.348254472	1.175264667
path_697	AURKA,CDKN1B,CIC,DNAJB1,DOT1L,EGFR,FANCC,HGF,KDM5C,MRE11A,MUTYH,PIK3CB,RAD51C,RAD54L,RUNX1,SMAD4,SMARCB1,SMO,SOX2,TSHR	0.518418856	1.112498615
path_698	AMER1,ARAF,CSF3R,DNAJB1,ETV6,FANCA,IL7R,JAK1,LATS2,MST1R,NOTCH2,PARK2,PIK3R3,POLD1,PTPRS,RARA,RTEL1,SH2B3,SHQ1,SMARCB1	0.680337127	0.943474383
path_699	ABL1,CDC42,CRLF2,FBXW7,FOXA1,GATA1,HIST1H3B,JAK3,KMT2A,NKX3-1,PDCD1,PDGFRA,PIK3R1,PTCH1,RBM10,RHOA,SMARCB1,SYK,TP53BP1,WT1	0.497747246	1.100917253
path_700	BCL2L1,BRAF,CCND3,CDC42,ERBB3,ETV6,IRS2,KDM5A,LATS2,MAX,MEN1,MPL,MYOD1,PRDM1,PTEN,PTPRD,RAD54L,RPTOR,SMARCD1,TAP1	0.118429382	1.293811586
path_701	ACVR1,ATRX,BARD1,ERBB4,ESR1,FAT1,FGFR1,FOXL2,FOXO1,FYN,GNA11,IGF1R,MGA,NFE2L2,NPM1,PAK7,PNRC1,RPTOR,SMAD4,TEK	0.086608663	1.283863854
path_702	ALK,BAP1,BRIP1,CTLA4,ERG,EZH2,HIST1H3E,HIST3H3,JAK1,MED12,NEGR1,NTRK3,PLK2,PTEN,PTPRD,RTEL1,SOCS1,SOX17,SPOP,SUFU	0.086123519	1.326523851

path_703	AKT3,ANKRD11,CDKN1A,CTLA4,E2F3,EZH2,GATA3,GNAQ,IKBKE,INHA,JUN,KDM5C,LATS2,NTRK1,PIM1,PTEN,RAD21,RPS6KA4,SDHA,TGFBR2	0.21085357	1.196864151
path_704	AR,BCL2,CALR,CARD11,CD274,CXCR4,CYSLTR2,FBXW7,FGFR1,FGFR3,GATA1,IGF2,IL10,MSH2,NOTCH4,PIK3CB,PTCH1,RAC2,TRAF7,U2AF1	0.445179027	1.114588824
path_705	BRD4,BTK,CDKN2C,ERCC2,FLT1,GRIN2A,IGF1R,LATS1,LATS2,MYCN,NBN,NCOA3,NOTCH4,PDCD1,PIK3R1,PTEN,RTEL1,STK40,TMPRSS2,TRAF7	0.270740415	1.20467856
path_706	BCL2L11,CCND3,CDH1,E2F3,EPHA5,ERBB4,ETV6,FGFR2,GNA11,HGF,HIST1H3I,INPP4B,MITF,NF1,PLK2,RAD51D,RBM10,RNF43,RUNX1,SOX9	0.221387028	1.203226217
path_707	ANKRD11,BCL2L11,CALR,ERCC2,FANCC,FOXL2,GNAQ,H3F3C,HIST1H1C,IGF2,KDM5A,KMT2D,MST1R,PAK1,PAK7,PRDM1,PTPRT,RHOA,RPS6KA4,SDHC	0.524649129	1.093942327
path_708	AKT1,CDC42,CDK12,CTNNB1,CXCR4,E2F3,EIF4A2,ETV1,FBXW7,FGFR1,FUBP1,HIST1H3I,JAK2,MALT1,MDM4,MLL3,MUTYH,PALB2,PREX2,RAD51B	0.173820064	1.293189156
path_709	ALOX12B,ASXL1,CDK8,CTNNB1,DNMT3B,FOXL2,HIST1H3H,JAK2,NEGR1,NF2,NOTCH4,PARK2,PIK3R3,PIM1,PTEN,RAD54L,RUNX1,SLX4,SOS1,TET2	0.100348533	1.353680648
path_710	AXIN2,BLM,CDC42,CDC73,CDKN2C,DNAJB1,FGF19,FOXO1,HIST1H3D,KNSTRN,MAP3K1,MDC1,MEN1,NOTCH3,PIK3C2G,PPM1D,RASA1,RUNX1,SDHAF2,SPOP	0.141557945	1.23723621
path_711	ARID2,BLM,BRD4,CCNE1,CD79B,CREBBP,H3F3C,INHA,INSR,JAK3,KDR,KMT2A,PAK1,PAK7,PBRM1,RAD54L,RPTOR,SDHAF2,SMARCA4,STK40	0.789511426	0.956699974
path_712	AKT3,CASP8,CDC42,CTCF,ELF3,ETV1,FYN,MAP3K13,MUTYH,NOTCH2,NOTCH4,NTRK3,PARP1,PMAIP1,RFWD2,RUNX1,SOX2,SPEN,STAG2,STK40	0.213226857	1.193986323
path_713	AURKA,BCOR,ETV6,FGFR1,FOXA1,FUBP1,HIST1H1C,HIST1H3C,IKZF1,MCL1,MGA,MSH3,MYC,NTRK2,PRDM1,RFWD2,RPTOR,SDHB,TMPRSS2,TNFAIP3	0.055137352	1.308283353
path_714	ATM,BARD1,BCL2L1,BMPR1A,FLT1,FOXA1,GATA2,GLI1,GRIN2A,IGF2,KDM5C,MAP2K2,MAP3K1,MET,MYCN,NOTCH4,PAX5,PIK3R1,RAD21,RUNX1	0.708358967	0.943693916
path_715	AURKA,AXIN2,BCL2L1,CHEK1,EPHB1,FANCC,FOXL2,IKBKE,KDM5C,KIT,KRAS,MDM2,MLL,PARK2,PIK3CA,PIK3R2,PTPRD,RPS6KA4,SHQ1,SOX9	0.179159775	1.212044946
path_716	ARID1B,AXIN2,CDC42,CDK6,EPHA7,ETV1,FGFR3,H3F3C,HIST1H3A,HOXB13,KIT,KRAS,MDM4,MSH6,NUP93,PDGFRA,PREX2,SDHA,SOX9,SRC	0.273164275	1.181423887

path_717	AXL,CARM1,CDC42,CIC,CTNNB1,EED,EPHA3,FOXP1,HIST3H3,IRF4,MDC1,MDM4,PIK3CB,PMS1,PREX2,RBM10,REL,TET2,TP63,TSHR	0.069290623	1.334367038
path_718	ATR,AXIN2,CARD11,CDKN1B,CEBPA,CREBBP,ERCC5,FGFR2,FUBP1,GATA3,HIST1H3C,IGF2,KLF4,LATS1,MAP2K4,MAP3K13,MAX,PALB2,PLCG2,RICTOR	0.161129828	1.299109512
path_719	ARID1A,CCND2,CTLA4,EIF1AX,EIF4E,EPHA3,ETV6,IFNGR1,MALT1,MSH3,MSH6,NTRK2,PDCD1,RAC2,RAD51,SDHB,SF3B1,SPEN,SRC,TP53BP1	0.087865567	1.347401515
path_720	ARAF,ARID2,EIF4E,EP300,FBXW7,FGF19,FGF4,FGFR1,IRS1,KLF4,MAP2K4,MEF2B,PIM1,PMS1,PPM1D,PTCH1,PTPRS,RAC2,RAD21,RICTOR	0.289082811	1.163303727
path_721	BLM,BRCA1,DNMT3A,FANCC,FAT1,FH,INPP4A,MAP2K1,MAP3K1,MGA,MSH6,MUTYH,MYOD1,NOTCH4,PIK3R2,RAD51C,RARA,SMAD3,SOX9,STK11	0.081479234	1.289515039
path_722	ACVR1,AR,CDKN1B,CTLA4,CYSLTR2,EP300,EPHA5,ERCC5,FLT4,JAK1,KNSTRN,MDM4,MLL,MRE11A,MSH3,PDCD1,PIK3CB,PIK3CD,RAC2,SDHC	0.095892968	1.266936106
path_723	AKT3,CARD11,CD274,CUL3,DNMT3A,FOXL2,HIST1H3A,HIST1H3D,INSRR,KLF4,MPL,MTOR,PBRM1,PMS2,RAD51B,SDHB,SOS1,SOX9,SPEN,TET1	0.396685663	1.128993937
path_724	AXIN1,AXL,BARD1,CDH1,CDK6,CHEK2,CTNNB1,EIF4A2,ERCC2,FANCA,FGFR2,FH,KDM5C,KDR,PMS1,RASA1,RICTOR,SDHA,SMAD2,TSC1	0.602821031	1.076952969
path_725	ARID5B,CDC42,CHEK1,CHEK2,FOXA1,GSK3B,HIST1H3A,IDH1,KMT2A,NCOR1,NFKBIA,NUF2,PIK3CA,PIK3R3,PRDM1,RARA,RFWD2,RTEL1,SOX9,TBX3	0.346302718	1.143532653
path_726	ARID5B,CCND2,CIC,ESR1,GRIN2A,HIST1H3D,HIST3H3,HNF1A,IKBKE,JUN,KDR,KMT2A,MST1,MUTYH,NFE2L2,PREX2,RASA1,SMAD2,SPEN,STAT5B	0.504136082	1.099890243
path_727	AR,ARID1B,BCL2L1,CREBBP,ERRFI1,FGFR1,FLT3,H3F3C,INPP4B,KRAS,MSH3,NF1,NOTCH1,PDGFRA,PIK3CG,POLE,PREX2,PTCH1,RPS6KB2,TOP1	0.450727529	1.113039158
path_728	APC,ASXL2,AXIN1,BCL2L11,BMPR1A,CDC73,CSF1R,DROSHA,ERBB2,FGF4,MED12,MLL3,MTOR,PAK7,PIK3R1,PNRC1,RNF43,SMARCA4,TAP2,TP53	0.223384123	1.215426138
path_729	CD274,CDK6,DROSHA,ELF3,EPHA5,FANCC,MALT1,MEF2B,MST1,MTOR,PBRM1,PDCD1,PIM1,RAD50,RAD51B,RAD51C,RHEB,RYBP,SH2D1A,SRC	0.522191557	0.913585431
path_730	ALOX12B,ANKRD11,CDKN1B,CHEK2,DNMT3B,EED,ERCC5,FANCA,GLI1,HNF1A,LATS2,MCL1,MEF2B,MLL,NKX2-1,NOTCH3,PARP1,PDGFRB,PTPN11,RAD50	0.063392714	1.300852154

path_731	ASXL1,CASP8,CHEK1,FGF19,FGF4,FOXO1,GLI1,GSK3B,H3F3C,JUN,KDM5A,MAP3K1,MAP3K13,MLL,PARK2,POLD1,RAD51B,SDHAF2,SH2D1A,SPEN	0.409735993	1.125249325
path_732	AKT2,CCND3,FAT1,FOXL2,FUBP1,HIST3H3,INPP4A,IRF4,KDR,MAP2K1,MSH2,NFKBIA,NKX3-1,NPM1,PMS1,PTEN,SF3B1,SH2D1A,TOP1,TRAF2	0.050364869	1.315123652
path_733	ALK,ARID5B,ATRX,B2M,BCL2,ERBB4,HIST1H3A,IDH1,IFNGR1,MAP2K1,NCOA3,NCOR1,NOTCH4,PBRM1,PDGFRA,PDGFRB,POLD1,PPP2R1A,PTEN,SLX4	0.259959497	1.18892705
path_734	ACVR1,ALOX12B,AXL,CCND2,CEBPA,CIC,DICER1,FLT3,FLT4,INSR,KEAP1,MEN1,PIK3CB,RAD51,RARA,RPS6KA4,RTEL1,RYBP,SYK,TMPRSS2	0.208129432	1.246853348
path_735	BCL2L1,BCOR,BLM,CARD11,CDC73,CEBPA,CHEK1,CIC,CSF3R,CXCR4,DROSHA,FUBP1,INPP4B,KIT,LATS2,MST1R,NFE2L2,NUF2,PAX5,PIK3CG	0.192432213	1.239267968
path_736	AKT2,AKT3,ALK,BCOR,CDK8,CXCR4,DIS3,FAM175A,FGFR4,FOXL2,KDM5C,MALT1,MAP2K2,MDC1,MET,PDGFRB,PIK3CG,PPP2R1A,RECQL4,SDHB	0.348661587	1.161996193
path_737	BARD1,CD274,CD276,CDC73,DICER1,EIF4E,ERG,ETV1,GNAQ,HIST1H3I,IKBKE,KDM6A,PNRC1,PTPRD,RAD51,ROS1,SPOP,STAG2,SYK,TOP1	0.082051614	1.28029306
path_738	ARID5B,BAP1,BRD4,CARM1,DNMT3A,FAM175A,FYN,GATA3,INPP4B,KEAP1,KLF4,MDC1,MST1R,NOTCH3,NPM1,PGR,PTCH1,PTPN11,SPEN,TBX3	0.054904186	1.325160729
path_739	ANKRD11,ARAF,BARD1,BCL2L1,CCND2,CD274,CUL3,FANCC,GATA1,HIST1H3A,INPP4B,KDR,MEF2B,MITF,MLL,NKX2-1,PDGFRA,PTPRD,RAD51B,SLX4	0.300458235	1.180737695
path_740	ACVR1,ALOX12B,BBC3,CCNE1,CDC42,FGFR1,HLA-A,HOXB13,IDH1,IFNGR1,IRS2,LATS2,MAP2K1,MCL1,MDC1,NTRK2,PIK3C3,SDHB,SMAD4,SUFU	0.436645532	1.17012858
path_741	BRCA1,CDK12,ERCC2,FH,FOXL2,HIST1H3B,HIST1H3J,IGF1R,INHBA,MAP2K2,MAP2K4,NTRK3,NUP93,PPM1D,RHEB,RNF43,SHQ1,SMARCD1,SRC,TNFAIP3	0.357122399	1.139213099
path_742	AKT2,CDC42,CTCF,CUL3,CXCR4,FAM175A,FANCC,FGFR2,FLT1,FUBP1,H3F3C,HIST1H3G,MRE11A,NTRK3,PMAIP1,POLD1,RASA1,SDHA,SMAD4,TP53BP1	0.394448141	1.128001305
path_743	ABL1,CARM1,CDKN1A,CUL3,DIS3,EGFR,FANCC,FOXO1,IRS1,MEF2B,PHOX2B,PIK3R2,PIK3R3,PMS2,PPM1D,SDHAF2,SMARCA4,STAG2,SUFU,TGFBR2	0.104003023	1.30111323
path_744	BCL10,CARD11,CBL,CD276,CDC73,CRLF2,DICER1,FBXW7,FLT3,HGF,HIST1H3C,MDM4,MUTYH,PBRM1,PMS2,PTCH1,SDHAF2,SOS1,TCF3,TET2	0.548757079	1.088445398

path_745	ARID5B,AXL,BAP1,CDK12,CDKN1B,CSF1R,DNMT3A,ERCC5,GNAS,GSK3B,HIST1H3G,INPP4B,MAX,NKX2-1,PIK3C3,POLD1,RNF43,SOS1,STAG2,TMPRSS2	0.856752284	0.969019557
path_746	AMER1,BCL2L11,BRD4,DNMT3B,FGFR2,HIST3H3,MAP3K13,MAX,MST1,MTOR,NFKBIA,NOTCH3,PARK2,PTPRS,RAC2,SOCS1,STAT5B,TBX3,TET1,TET2	0.14506479	1.341509212
path_747	ABL1,AKT2,CDK4,CDKN1B,CIC,CRLF2,IKZF1,INSRR,MCL1,NOTCH2,NTRK3,PIK3C3,PMAIP1,PMS2,RAD21,RBM10,RHEB,SDHC,SPOP,STAT3	0.109147935	1.259085299
path_748	BAP1,BLM,BRCA2,CSF3R,CYSLTR2,FGFR1,FOXO1,FOXP1,HIST1H3A,HIST3H3,INHA,INSRR,MSH2,MST1,NSD1,NTRK3,PTPRD,RASA1,SMARCD1,STAG2	0.661402916	1.077154541
path_749	ASXL1,BAP1,BMPR1A,CDK6,CSF1R,CTLA4,FGFR4,FOXA1,HOXB13,IDH1,INPP4A,MAP3K13,MYOD1,NKX2-1,PIK3C2G,PTPRT,SMAD2,STK40,TET1,TET2	0.073583685	1.344064172
path_750	ALOX12B,AMER1,ATRX,BRD4,CARM1,DNAJB1,ERG,ESR1,GRIN2A,H3F3C,HIST1H3A,HIST1H3D,MAP3K13,MEN1,PALB2,PTPN11,RAD50,RNF43,SRSF2,TET2	0.688210141	1.081035162
path_751	ABL1,ARID1B,AXL,CCND1,CD274,CENPA,DNMT1,EIF4A2,EPHA3,FGFR4,HIST1H3J,HOXB13,IGF2,JAK1,MYCN,NOTCH1,PALB2,SOX17,SRC,STAT5B	0.137681085	1.307277964
path_752	AKT2,APC,AXL,BRAF,CTCF,DDR2,ESR1,ETV1,FYN,GNAS,IRS2,KMT2A,MCL1,MTOR,PDCD1,PTPRT,RPTOR,SHQ1,TMPRSS2,TSC2	0.991939671	1.001630634
path_753	AKT3,ANKRD11,ASXL1,ATR,BAP1,BCOR,DNAJB1,FGF19,GPS2,IFNGR1,KDM6A,MYC,MYOD1,NPM1,PPP2R1A,RAD51C,RNF43,SH2D1A,SOX9,TNFAIP3	0.85916408	1.025657733
path_754	BRCA1,CDK4,CDKN2A,EIF1AX,FOXA1,IGF1R,IKZF1,INPP4A,KDR,NOTCH4,PIK3CB,PIK3CD,PIM1,PLCG2,PMS1,POLE,PPP6C,PTCH1,RET,SETD2	0.053573542	1.381877546
path_755	ALOX12B,CCND3,CDKN1A,CDKN1B,DDR2,ERCC2,GRIN2A,HIST1H3A,HOXB13,IKZF1,INHA,INHBA,NFE2L2,PARP1,PMS2,PREX2,RAD54L,REL,SMO,SOX17	0.86877943	0.97670117
path_756	AKT1,BLM,CDKN2C,CEBPA,CHEK2,JAK2,KMT2D,KNSTRN,LATS2,MST1R,PIK3C2G,PIK3CA,PIK3CG,PIK3R2,POLD1,PRDM1,RBM10,SDHAF2,SPEN,TBX3	0.596832609	1.085453504
path_757	B2M,CASP8,CTNNB1,DDR2,EGFR,FBXW7,FGF19,HIST1H3A,HOXB13,IGF2,INHBA,PAK1,PALB2,PHOX2B,PIK3CD,RAD21,RAD54L,RICTOR,SOS1,TAP1	0.483380452	1.136702626
path_758	ALOX12B,CDC42,ETV6,FYN,HLA-A,IGF2,INHA,IRF4,MCL1,MEF2B,NCOR1,NOTCH4,NPM1,PIK3R3,POLD1,PPP6C,PTEN,SYK,TMPRSS2,TOP1	0.550972792	1.088877459

path_759	CRLF2,EIF1AX,ERG,ESR1,EZH2,FBXW7,HIST1H3C,HIST1H3G,ICOSLG,JAK2,MAP2K2,MEN1,MGA,MPL,NFE2L2,PDGFRB,RASA1,RET,STAG2,TBX3	0.225515721	1.242423516
path_760	ARAF,ARID1B,ATR,BCL2L1,CSF1R,DOT1L,FANCA,FANCC,FGFR2,FLT3,GATA3,GNAQ,HGF,INHA,MEF2B,MLL2,MLL3,NCOR1,PARK2,POLE	0.514490681	1.104231524
path_761	ATM,BLM,CDKN2C,CEBPA,CYSLTR2,ERBB2,ERRFI1,INHA,INSRR,KNSTRN,MST1R,NKX2-1,NPM1,PDGFRA,PLCG2,PPM1D,PPP2R1A,RPS6KA4,SMARCB1,TCF7L2	0.812073365	1.034174783
path_762	ARID1A,ARID5B,CCNE1,CDK6,CIC,CSF1R,DNMT1,DNMT3A,DOT1L,DROSHA,FOXA1,GNAS,IL7R,MDC1,NOTCH1,PIK3CB,RAD51,RTEL1,SETD2,SMARCD1	0.199691675	1.226467789
path_763	CDKN2A,CUL3,DDR2,ERCC4,FBXW7,FGFR3,FLT3,HIST3H3,IGF2,KDR,KEAP1,KRAS,MAP2K1,MAP2K4,MUTYH,PIK3C3,PIK3R3,PMS2,TP53,TRAF2	0.206974495	1.197847928
path_764	ACVR1,AURKA,CDK6,ERBB4,HOXB13,IGF1R,MAP2K1,MAP3K1,MAP3K13,MITF,MSH2,MYCL1,NKX2-1,NTRK1,PLCG2,PPP6C,RAD54L,RARA,RHEB,RNF43	0.256938569	1.245518936
path_765	BCL2,CDK12,CIC,ERBB4,GATA1,GNAS,GSK3B,INHBA,KNSTRN,MSH3,MST1R,MYCN,NOTCH1,PARK2,PIK3C2G,PIK3C3,PMS2,SMARCA4,SUFU,TET1	0.090281837	1.314067934
path_766	ACVR1,ANKRD11,BCL2L11,CARD11,CD274,CEBPA,DNMT3A,ERBB4,ESR1,FANCA,FAT1,IRF4,MYOD1,NOTCH3,NTRK1,PARP1,PIM1,RHEB,SMAD4,SUFU	0.242349261	1.204557045
path_767	AR,AXIN1,CREBBP,FBXW7,HIST1H3I,IL7R,INPP4A,IRS1,JUN,MAP2K2,MLL3,MSH3,NOTCH4,RAD54L,RAF1,RET,ROS1,SDHC,SH2D1A,SPOP	0.520778044	1.11327285
path_768	ARID5B,BCL2L11,CDK4,CHEK1,CSF3R,CYSLTR2,DIS3,EIF4A2,FGFR3,MED12,MYCN,NF1,PALB2,PIK3C2G,PIK3CB,RBM10,ROS1,SDHB,SDHC,SOX17	0.078557957	1.349092317
path_769	BRIP1,CYSLTR2,DNAJB1,EIF4A2,EPHA3,ERCC4,FAM46C,FLT4,FOXO1,HNF1A,KDM6A,MLL2,MST1R,PMS2,POLE,PREX2,RBM10,RNF43,SMARCA4,SMARCB1	0.065755598	1.305157858
path_770	AR,BBC3,BRAF,CREBBP,FANCA,FGFR1,GNA11,GNAQ,GSK3B,IFNGR1,INHBA,JAK3,MSH3,PIK3R3,PNRC1,RAD51D,REL,RUNX1,TP53,TP53BP1	0.054478471	1.32368321
path_771	BRAF,CASP8,CCNE1,CDKN2A,E2F3,EPHA3,FGFR4,KEAP1,MGA,MLL3,MSH2,PAK7,PMAIP1,SF3B1,SMARCD1,SYK,TGFBR1,TGFBR2,TMPRSS2,TRAF2	0.238155109	1.242991327
path_772	AXIN1,BMPR1A,CALR,CCND1,CHEK1,EGFR,ERCC5,FAT1,FOXL2,IGF1R,IKBKE,MALT1,MLL2,MYCN,NBN,NOTCH1,PARK2,ROS1,SMAD4,SOX9	0.212060689	1.205991945

path_773	ASXL1,BRAF,CD276,CDKN1A,EIF1AX,FAT1,FGF19,FOXL2,IGF1R,KIT,MGA,MYCL1,MYOD1,NUF2,NUP93,PMS2,PPP6C,PREX2,RTEL1,TEK	0.237252826	1.25056407
path_774	ALOX12B,BRD4,CDK12,CRLF2,DOT1L,DROSHA,FGF4,FOXO1,GNA11,IGF1R,KNSTRN,MDM2,MPL,PGR,PPP6C,PTPRD,RAC2,RAD51D,REL,SDHC	0.278555123	1.165930945
path_775	AXL,BAP1,BRD4,CCND3,CTLA4,CUL3,DIS3,FGFR4,FLT4,FOXO1,HIST1H3H,IL10,LATS2,MYCL1,NCOR1,PIK3C2G,RAD51C,RHOA,TNFAIP3,YES1	0.391025357	1.129298221
path_776	BBC3,BCL2L11,CTNNB1,DNAJB1,EIF1AX,FAM175A,GATA3,LATS2,MLL2,NTRK1,PBRM1,POLD1,RAC2,RAD51C,RNF43,SDHB,SF3B1,SMAD4,TET2,TGFBR2	0.799750072	1.042933504
path_777	ACVR1,ARAF,BTK,CDH1,CREBBP,ERBB4,FGFR2,FLT1,JAK1,KIT,MAP3K13,NPM1,NTRK3,POLE,RAF1,RBM10,SHQ1,STK40,TCF3,TET1	0.330712543	1.160811064
path_778	AKT1,ATRX,BBC3,BCL10,BCL2L11,CCND1,CD79B,CRLF2,CTLA4,EPHB1,ERG,ETV6,HIST1H3C,HIST1H3I,KDM5A,KEAP1,PIK3R1,RAD21,RAF1,RHOA	0.050232535	1.481316227
path_779	AKT1,ARAF,AXIN1,ERBB3,FAT1,GATA2,HIST1H3C,HIST1H3J,HOXB13,IKZF1,JAK2,MCL1,MDM2,PIK3R3,PNRC1,POLE,RB1,RHEB,RICTOR,SPOP	0.134838613	1.346388958
path_780	CCND1,CCND2,CCND3,CD276,CEBPA,DNAJB1,EIF4E,FAT1,NUF2,PMAIP1,PRDM1,RAD50,RAD51,RAD54L,SDHB,SHQ1,SRSF2,STK40,SUFU,TAP2	0.441277015	1.123772977
path_781	AMER1,BRCA1,CSF3R,DAXX,FLT4,FOXP1,H3F3C,HGF,INSRR,IRS2,MRE11A,MST1R,NRAS,PHOX2B,PIK3R1,RAD51D,RNF43,SDHA,SDHB,TGFBR2	0.169673565	1.271605045
path_782	ATM,BCL2L11,BMPR1A,CDK12,CDKN1A,CHEK1,CTLA4,ERCC2,HIST1H3D,INPP4B,KRAS,MGA,MLL,MST1,NTRK2,PAK1,PARP1,SHQ1,SPOP,TAP2	0.354615934	1.143704902
path_783	ABL1,ARID1A,CREBBP,CSF1R,DIS3,EIF4A2,ERG,IDH1,KIT,KMT2A,MRE11A,MYC,NCOR1,NTRK3,PAK1,PLK2,PMS1,PTPN11,RHOA,SLX4	0.988379897	0.997617381
path_784	APC,ASXL2,ATR,BBC3,BCL6,GNA11,GPS2,HIST1H3B,IKZF1,KMT2A,KMT2D,MAP2K4,MSH3,MST1,NKX2-1,NOTCH3,PIK3R3,PPM1D,RHEB,SOCS1	0.405943367	1.129005501
path_785	ARID1A,ARID2,ARID5B,ATRX,BCL10,CCNE1,CTNNB1,CUL3,EPHA3,FOXL2,KIT,MET,MSH2,MST1,NFKBIA,PAX5,PTPRS,RASA1,RB1,SH2B3	0.07121536	1.302948338
path_786	ALOX12B,AR,BARD1,BRIP1,CD79B,CDK12,EGFR,EPHA7,ERCC2,H3F3C,IFNGR1,KMT2D,KNSTRN,NCOR1,NUF2,PIK3CA,PIK3R3,PTPRT,SH2B3,TERT	0.788555169	1.041172923

path_787	ANKRD11,BRCA1,CDKN1B,CEBPA,CYSLTR2,DIS3,FLT3,HIST3H3,HLA-A,IL7R,KMT2A,MAP2K4,MST1,PNRC1,POLD1,PTPRD,RAD21,RAD51D,RHOA,SDHC	0.868213881	1.023777557
path_788	ARID2,ASXL1,AXIN1,CSF3R,DNMT1,EIF4E,ERBB2,GNA11,HIST1H1C,HIST1H3E,KNSTRN,MALT1,MAP3K1,NTRK2,PALB2,PIK3CA,RAD51C,SH2B3,SH2D1A,STAT5B	0.341505238	1.202554237
path_789	AMER1,ATR,BRCA2,CASP8,CDC73,CYSLTR2,DNMT3A,ERRFI1,FGFR3,FH,FLT1,IGF2,IL7R,NUP93,PDGFRB,PPP2R1A,PTPRS,RAD50,SMARCB1,SYK	0.14696038	1.308184504
path_790	BCL10,CDKN2A,CHEK1,DOT1L,FGFR2,HGF,IFNGR1,IKBKE,INSRR,KDM5A,KDM5C,MDM4,NEGR1,NRAS,PAK1,PIK3C2G,PLCG2,RAD54L,RYBP,SMAD2	0.766548249	1.051243986
path_791	AKT1,ALOX12B,ARID5B,BCL2L1,CCNE1,EIF4A2,ERBB2,ERCC5,ERG,FANCC,FGF4,FYN,IFNGR1,IL7R,JUN,LATS1,MYCL1,NKX2-1,NUF2,PMS2	0.737384975	1.049809691
path_792	AXL,CDK12,CHEK2,DAXX,EED,EPHB1,ERCC2,ETV6,FOXA1,FUBP1,NF1,NRAS,PIK3R1,PTPN11,RAF1,RICTOR,SDHAF2,SF3B1,SPEN,TNFAIP3	0.272584154	1.16789225
path_793	BAP1,BCL6,CDKN2A,DICER1,DOT1L,EIF4A2,FOXA1,HIST1H3E,IKZF1,IRF4,KDM5C,MALT1,MRE11A,MST1R,PDGFRA,PMS1,PTCH1,SMAD3,STK11,TGFBR2	0.057585083	1.334445938
path_794	ABL1,AXIN2,AXL,CCND1,GATA1,HIST1H3D,KNSTRN,LATS2,MDM4,MST1,MTOR,NOTCH1,PDGFRA,PLCG2,PTEN,RAD51B,SOX2,SRSF2,TSC2,XPO1	0.820341703	1.032670786
path_795	AKT3,ALK,CCNE1,CD79B,CDKN2C,DNMT1,E2F3,HIST1H3C,IDH1,IL10,MYC,NF2,PDCD1,PGR,PLK2,RFWD2,RICTOR,RPS6KA4,RPTOR,SDHA	0.192126382	1.202562679
path_796	CBL,CDKN1B,DIS3,FAM175A,FLT3,FOXP1,GATA3,JAK2,KRAS,MAP3K1,MYOD1,NCOR1,NFE2L2,NRAS,PAK7,PAX5,RAD50,TEK,TET2,TGFBR2	0.613034515	0.930216985
path_797	BCL6,CALR,CCNE1,CIC,DICER1,DIS3,EIF4A2,HLA-A,IDH1,IRS2,NEGR1,PLK2,PREX2,RAD51C,RTEL1,SMAD2,SMARCA4,SOS1,SUFU,TERT	0.660308925	1.064232001
path_798	ABL1,BLM,CD79B,CDKN1B,CDKN2C,CHEK1,CIC,E2F3,ELF3,GSK3B,IGF2,INHBA,MAP2K1,MLL2,PGR,PIK3R2,PTCH1,RB1,SOCS1,TCF3	0.623456977	1.089178393
path_799	CD274,CDKN1B,CXCR4,FANCC,GSK3B,H3F3C,HIST1H3G,IFNGR1,IGF1R,MALT1,MEN1,PAK7,PAX5,RAD51,RASA1,RHEB,SMARCA4,SMO,STK11,SUFU	0.071312453	1.341277762
path_800	AKT3,BLM,CARD11,CDKN1A,CRLF2,HIST1H3C,LATS2,MPL,MYC,NCOR1,NOTCH1,NOTCH2,NUP93,PAK1,PMAIP1,REL,SDHA,STAT3,TAP1,TAP2	0.341092039	1.197525332

path_801	AXIN1,BCL2,CYSLTR2,EPHA5,FAM46C,H3F3C,HIST1H3C,MAP2K2,MRE11A,NBN,PDGFRB,PIK3R1,PLK2,RAD50,RAD51D,RAF1,REL,SMAD3,SMAD4,SPOP	0.371663274	1.181464806
path_802	AKT1,ATRX,BCL6,EGFR,HIST1H1C,HOXB13,ICOSLG,MDM2,MYCL1,NBN,NEGR1,NKX3-1,NRAS,PIK3R1,PIK3R2,PLCG2,RET,SOS1,STK40,SUFU	0.609290481	1.09153916
path_803	ATR,EED,EPHB1,ESR1,FANCA,FGF19,GSK3B,IKBKE,KDM6A,KRAS,MDM2,MSH2,MSH3,MSH6,NOTCH3,PDCD1,PRDM1,RFWD2,SDHAF2,TBX3	0.554013377	1.095580376
path_804	ACVR1,BRIP1,CALR,FAT1,FGFR2,FH,GLI1,HIST1H3D,IKZF1,INHBA,LATS2,MDM4,MET,MRE11A,MSH2,MYOD1,RAD54L,RNF43,RPS6KB2,SUFU	0.191314332	1.25490213
path_805	ACVR1,ARID1B,BARD1,BCL2L11,BRCA1,BRD4,CTCF,EP300,FOXL2,GNAQ,GPS2,MALT1,MCL1,MDM4,NOTCH1,PMAIP1,RAC2,RAD51,RB1,TBX3	0.525611704	1.123054749
path_806	AKT3,ARID2,BMPR1A,CDKN1B,CHEK1,CHEK2,FANCC,FLT4,GNA11,IKBKE,INHA,IRS1,KDR,NOTCH4,RB1,SDHAF2,SHQ1,SMARCD1,STAT3,TP53	0.682403897	1.059562963
path_807	AKT2,BMPR1A,CASP8,FGF19,FGFR2,GATA1,GNAS,H3F3C,HIST1H1C,IL10,KDR,LATS2,NEGR1,NF1,PIK3CB,PRDM1,SRC,SRSF2,TEK,TMPRSS2	0.996993956	0.999465403
path_808	BRAF,CD79B,CDKN2A,EIF4E,ERCC2,FUBP1,HIST1H3D,KDM5A,MAP3K1,MDM4,MITF,NTRK2,NTRK3,PARK2,PDCD1,RUNX1,SF3B1,SH2D1A,SRSF2,TCF3	0.058185642	1.407200356
path_809	BMPR1A,CASP8,CCND1,CRLF2,DNAJB1,EP300,FAM46C,FGFR4,FOXA1,HLA-A,NF1,NUP93,PAX5,PMS1,RAD51,RFWD2,SMAD4,SOCS1,SPOP,TMPRSS2	0.386470244	1.129707407
path_810	ALOX12B,ARAF,ARID1B,CCND3,CCNE1,CDC42,CHEK2,DNAJB1,DNMT3B,EPHA7,ERBB3,FGF3,KMT2A,MAP2K2,MDM2,MYOD1,PIK3R2,PIK3R3,PTPRS,TSC1	0.618788052	1.073759982
path_811	BCL2L1,CHEK1,CREBBP,CTNNB1,E2F3,ERCC2,FGF19,FGFR1,FOXL2,GATA1,GSK3B,HIST1H3H,NUP93,PIK3CA,PLK2,RARA,SDHC,SH2D1A,SMAD3,TCF3	0.853482601	0.974025317
path_812	BRCA1,CEBPA,ERBB3,ETV1,FGFR3,FLT3,FOXA1,HIST1H3G,INPP4B,MCL1,MET,MST1R,NRAS,PALB2,PAX5,POLE,PPP6C,RB1,SMARCA4,U2AF1	0.24585365	1.207118709
path_813	ARID1A,ASXL2,CUL3,FGF3,FOXL2,IRF4,KDM6A,MDM2,MPL,NEGR1,PAK1,PIK3C2G,PIK3CA,PLK2,PPP6C,RAC2,RAD50,SLX4,SMAD2,SMO	0.366277601	1.164072778
path_814	ALK,ARAF,CD79B,CIC,DNMT3A,EIF1AX,GATA2,HIST1H3H,HIST1H3I,HNF1A,JAK1,KMT2A,KMT2D,MRE11A,MSH3,PDGFRB,POLD1,PTPRT,SOX2,SOX9	0.719189411	1.064535266

path_815	ALOX12B,AXL,BTK,CDH1,CDKN2C,CHEK2,CSF3R,CUL3,FAM175A,FAT1,HIST1H3C,NF2,NTRK3,NUF2,PDCD1,PIK3R2,RAD21,SETD2,SPEN,TCF7L2	0.097105783	1.341283809
path_816	ALK,ALOX12B,ANKRD11,CRLF2,CXCR4,EGFR,EPHA7,INSRR,PALB2,PIK3CB,PLCG2,PTCH1,RASA1,RB1,SDHA,SDHB,SETD2,SOS1,TET2,TP53BP1	0.762142029	0.952715554
path_817	BRIP1,CD276,CENPA,CTCF,EPHA3,FANCC,FAT1,HIST1H1C,HIST1H3A,IDH1,KIT,MPL,MYCL1,NEGR1,PALB2,PMAIP1,RAC2,SDHA,SOS1,XPO1	0.221740153	1.229565329
path_818	ASXL1,CREBBP,DROSHA,EPHA3,ERG,FANCC,ICOSLG,IGF2,INSR,IRS1,KEAP1,LATS1,MGA,MSH6,NTRK3,PAX5,POLE,RPS6KA4,SDHC,SMARCD1	0.53561122	1.106550135
path_819	ATR,BLM,BMPR1A,BRCA1,CDKN1B,CTLA4,ERBB4,FAM175A,FANCC,GNA11,MST1R,NFE2L2,PIK3C3,PIK3R3,PLCG2,PPP2R1A,SH2D1A,SMARCB1,SOX2,TET1	0.495948492	1.120491988
path_820	AURKA,CBL,CEBPA,DROSHA,EIF1AX,ELF3,ERCC5,EZH2,FAM46C,GATA3,GPS2,H3F3C,HIST1H3H,IRS2,KDM5C,MLL2,PBRM1,SOX17,SOX9,STAT5B	0.526168922	1.132785981
path_821	ASXL2,BRIP1,CDK4,CYSLTR2,EED,ETV6,GATA1,HIST1H3J,ICOSLG,JAK1,KDR,KIT,KNSTRN,MTOR,NOTCH4,PIK3CA,PIK3CB,PMS2,RAC2,RAD21	0.84450191	0.963926723
path_822	AKT3,ARID1A,ARID5B,ASXL1,ASXL2,BCL2,BCOR,CD274,CD79B,CXCR4,EP300,ERCC5,GNAQ,IRF4,KDM6A,NFKBIA,NUP93,PARP1,RAC2,YAP1	0.140472547	1.286048881
path_823	ARID1A,AXIN2,BCL2,CASP8,CCND3,CSF1R,DNMT3A,EGFR,ETV6,EZH2,FYN,MAX,MDM4,MPL,NCOR1,PBRM1,PPM1D,RTEL1,SOX2,TP63	0.149339092	1.252755288
path_824	AR,CCNE1,CD276,CDK4,DNMT1,FAM46C,FUBP1,GNAS,IFNGR1,INSR,KDR,MSH2,PDGFRA,PIK3C3,PRDM1,RAD50,RAD51D,REL,SUFU,TP53BP1	0.156243202	1.222224674
path_825	AR,AXL,BAP1,BRCA1,CSF1R,EGFR,FGFR2,HIST1H3C,HIST1H3G,MCL1,MEN1,MSH2,MYC,NPM1,NRAS,PIK3C2G,PIK3CB,PLK2,SF3B1,SPOP	0.590325166	0.9112495
path_826	BCL2L11,CREBBP,DIS3,EPHA5,ERCC5,ERRFI1,FAM46C,FLT1,GATA2,HIST1H3E,IDH1,KLF4,KMT2A,PAK1,PAK7,PAX5,PTCH1,RBM10,SMAD4,TERT	0.734712227	1.051849742
path_827	ARID5B,BBC3,BCL2,BCL2L1,BRAF,BRD4,CALR,CD274,CD79B,DOT1L,ETV1,FLT4,FOXO1,FYN,INPP4B,MSH3,PARP1,PDGFRA,PIK3R1,RAC2	0.052669698	1.320026128
path_828	ATRX,AXIN1,CD274,CRLF2,DNMT1,EIF4A2,EPHA7,EZH2,IGF2,JAK3,KMT2D,MALT1,MAP3K13,MLL3,MTOR,MYOD1,NCOR1,NTRK3,RARA,RET	0.159397789	1.264100612

path_829	AKT2,ARID1B,CIC,CTLA4,CTNNB1,ERCC2,FOXO1,KDM5C,KEAP1,MSH6,NPM1,NRAS,PMAIP1,PTEN,RFWD2,SETD2,SMARCD1,STAT3,TP53BP1,TP63	0.629584637	0.931416114
path_830	AMER1,BCL6,FGFR1,FUBP1,IGF1R,INHA,KNSTRN,MITF,MRE11A,PBRM1,PLCG2,PMS2,PPM1D,RAD51B,RAD51C,RICTOR,SETD2,SF3B1,SMAD4,STK11	0.177510049	1.231941603
path_831	APC,AR,AXIN1,BCOR,CARM1,CDK4,CDKN1A,CTNNB1,EPHA7,ETV1,FH,IRS2,JUN,KEAP1,MLL2,NPM1,PIK3CB,PRDM1,PTPN11,RICTOR	0.351567195	1.148299108
path_832	AXL,CCND2,CDH1,CUL3,GSK3B,ICOSLG,IDH1,IL7R,INSRR,KMT2A,MLL2,MLL3,MYC,PLK2,PPP2R1A,RB1,RECQL4,RUNX1,SOCS1,TET1	0.132173212	1.257092304
path_833	BCL2L11,CCND2,CDK4,CDKN2C,CIC,CSF3R,ERCC5,GATA2,IKZF1,MALT1,MCL1,NTRK1,PAX5,PIK3R1,PTPN11,SDHAF2,SMARCB1,SOX2,SOX9,SPOP	0.503337709	1.108188484
path_834	ARAF,ATRX,BAP1,CDK12,CHEK2,DNAJB1,ERRFI1,FGFR1,GNA11,KLF4,MDC1,MITF,NF1,NRAS,PHOX2B,PREX2,RHEB,RICTOR,RPS6KB2,TNFAIP3	0.109351915	1.326308086
path_835	BCL6,EIF4E,FAM175A,GATA1,GLI1,HIST1H3C,HNF1A,INSRR,KRAS,MEF2B,MGA,MRE11A,NTRK1,PARK2,PLCG2,PTEN,RAC2,RARA,RET,SOS1	0.060347458	1.336738293
path_836	ACVR1,ASXL1,AXL,BBC3,CDC42,DDR2,EPHA3,FGF3,MAP3K13,MLL,MLL2,NFE2L2,NPM1,PAK1,PBRM1,RAD54L,RB1,RBM10,RUNX1,SOX17	0.087799176	1.286052373
path_837	ARID1B,ATM,BCL2L1,BCOR,BLM,CARD11,CD79B,CDC42,EGFR,EIF4E,IFNGR1,LATS2,MALT1,PDCD1,PHOX2B,RAD51B,SDHA,SH2B3,SOS1,SRSF2	0.913767924	0.982050873
path_838	ALOX12B,CARM1,CDH1,EP300,FANCA,FBXW7,FOXL2,H3F3C,IFNGR1,INHA,MCL1,PALB2,PIM1,PPP2R1A,PTPN11,PTPRD,SMARCA4,SMARCD1,STAG2,STAT3	0.071405927	1.350404879
path_839	BCL6,CCND2,CREBBP,DDR2,DNMT3A,FOXL2,FYN,HIST1H1C,MYC,NF2,NFE2L2,NTRK2,PAK1,PLK2,PMS2,PTPN11,RTEL1,SDHC,SMARCA4,TP63	0.82288273	1.035897594
path_840	BRD4,BRIP1,CARD11,CIC,EED,FLT1,GNA11,INHA,MALT1,MAP2K1,MAP3K13,MDC1,NBN,NPM1,PIK3C2G,PIK3R2,PTPRS,RB1,RECQL4,SDHA	0.226275469	1.22162029
path_841	AKT1,BCL2L1,CUL3,FAT1,FYN,HIST1H3I,IKZF1,IRF4,KLF4,MLL2,MYC,NOTCH3,PIK3CD,PIM1,RAF1,RASA1,RBM10,SDHC,SMAD2,SMAD3	0.126402271	1.280948792
path_842	BCL2L1,CASP8,CDC73,DDR2,EIF4A2,ELF3,ERCC4,HIST1H3B,HLA-A,JUN,KEAP1,MEN1,MUTYH,PAK1,PIK3R2,RB1,RHEB,STAG2,TEK,TGFBR1	0.187675855	1.229785096

path_843	ATRX,BCL2,CARD11,CHEK2,CREBBP,EP300,GSK3B,HIST1H3A,IRS1,JAK2,KDM6A,MALT1,MDC1,MGA,MRE11A,PAX5,PPP2R1A,PRDM1,PTPRS,SPOP	0.071738823	1.330038373
path_844	AR,BCL2L1,CDC42,CDC73,DDR2,ERCC2,FYN,GATA2,KDM5C,KLF4,MEF2B,MSH3,NBN,NFE2L2,NOTCH2,PALB2,PIK3CA,PIK3CD,RB1,RHOA	0.572434018	1.110195999
path_845	ALK,CCNE1,CD274,CDK8,DNAJB1,ETV6,FGF19,FLT4,FYN,GNAQ,HIST1H3J,IFNGR1,MEF2B,NBN,PPP2R1A,RARA,REL,SETD2,SMARCA4,TNFAIP3	0.113254891	1.339016693
path_846	ARID1B,ATM,BTK,CDKN1A,CENPA,CSF3R,CYSLTR2,DNMT3B,EED,FOXA1,JAK1,JAK3,MDM2,MEF2B,MPL,MSH3,NTRK1,NTRK3,PLK2,POLE	0.261136328	1.215544996
path_847	B2M,BARD1,BRAF,BRCA1,CCND2,CDKN2A,DAXX,DOT1L,EIF1AX,EPHA7,ESR1,FAM46C,FOXL2,NOTCH2,NTRK2,PIK3CD,RAD54L,RYBP,SOX17,SOX9	0.44858923	1.137124346
path_848	AMER1,ARID2,BCL6,CD79B,CTNNB1,EP300,FOXL2,GLI1,GNAQ,GNAS,GRIN2A,KMT2D,MSH2,PAX5,PGR,RASA1,RET,RUNX1,SDHB,SF3B1	0.866049987	1.026680813
path_849	AXIN1,AXIN2,BARD1,CCND3,CDH1,CDK4,CIC,GATA1,GATA3,INSR,INSRR,MYOD1,NUF2,PDCD1,PTEN,RAD51D,RHOA,SDHAF2,SHQ1,SMAD2	0.68419294	0.94350136
path_850	AKT2,AXL,EIF4E,EPHA7,FGF4,FOXO1,GATA1,H3F3C,HGF,IL7R,NF1,PAK7,PTPRS,PTPRT,RAD51D,RET,SUFU,TCF7L2,TEK,TET1	0.487946336	1.115567501
path_851	ACVR1,ALOX12B,BAP1,GATA3,HIST1H3J,IDH1,IGF2,KDR,MAP3K13,MRE11A,NOTCH3,PAX5,PGR,PIK3C3,PIK3CB,PIK3CD,RPS6KA4,RPTOR,SDHAF2,SOX9	0.050308466	1.325373547
path_852	ASXL1,ATR,BBC3,BRAF,CALR,CCND3,DNMT1,EGFR,HIST1H1C,HIST3H3,INHBA,INSRR,MAP3K13,NTRK2,PIK3CA,PIK3CG,POLE,RAD51,RARA,ROS1	0.846853842	0.969996768
path_853	ABL1,ARAF,AURKA,BAP1,BTK,CDKN1A,CDKN2A,DNAJB1,JAK1,MYC,MYCL1,NOTCH4,RYBP,SDHAF2,SHQ1,SRSF2,STK40,SUFU,TERT,XPO1	0.500792435	1.099834234
path_854	AKT3,AURKA,CUL3,EED,FGF19,FGF3,FUBP1,GNAS,HIST1H3B,HIST1H3G,JAK1,KDM5C,KLF4,MALT1,NCOA3,PDGFRB,RAD51C,SDHA,SMAD4,TNFAIP3	0.28750103	1.164040049
path_855	AURKA,BRCA1,CD274,DDR2,DNMT1,DROSHA,ERBB2,EZH2,FANCC,FGF19,GLI1,INPP4A,MLL,MRE11A,NCOR1,NFKBIA,PBRM1,PDGFRA,PIK3CG,REL	0.20753038	1.242371542
path_856	AKT2,ARID1A,CCNE1,CDC42,CHEK1,CTNNB1,DNMT3B,ESR1,FGFR3,FYN,MYCN,NCOR1,PTEN,RAD51,REL,RTEL1,SOX9,SRC,SYK,TET2	0.989244536	0.997750731

path_857	BTK,CCNE1,CDC42,CSF3R,EIF4A2,EPHA7,FOXP1,HOXB13,IFNGR1,IKZF1,MSH6,MST1R,NCOR1,NF1,PAK1,PIK3R1,PLK2,SMO,SPOP,STAT5B	0.61258474	0.919067354
path_858	ABL1,ACVR1,AXL,DDR2,DNMT3B,ERBB4,ERCC2,FOXP1,H3F3C,IFNGR1,INHBA,JUN,NOTCH4,NPM1,PALB2,PARK2,POLE,PRDM1,PREX2,PTPRS	0.347075411	0.875758311
path_859	ALOX12B,CBL,CUL3,DNMT3A,DNMT3B,EED,FANCA,FOXL2,GLI1,HIST1H3H,IDH1,KMT2A,MDC1,MRE11A,MSH6,NFE2L2,PARK2,PREX2,ROS1,SRSF2	0.550119264	1.113150536
path_860	AXIN1,BCL6,CARM1,CHEK2,CTNNB1,FGFR4,FOXP1,GLI1,GSK3B,HIST1H3B,JAK3,KMT2A,MDM2,MEF2B,NCOA3,PIK3CG,RAD51D,RARA,SDHC,SOX9	0.273428954	1.170555004
path_861	APC,ARID5B,BCL6,DDR2,EIF4A2,FBXW7,HIST1H3H,IGF2,IKZF1,JAK2,MAP3K1,MED12,MET,NPM1,PBRM1,PDCD1,PDGFRA,PTPRT,RTEL1,SLX4	0.461075668	1.130135658
path_862	AR,BLM,CXCR4,ERRFI1,ETV6,GNAS,IKBKE,KDR,KMT2D,MAP3K1,MLL3,NCOA3,NOTCH2,PDCD1,PTPRD,RAD21,RAD51C,RB1,RNF43,SUFU	0.111097047	1.282345649
path_863	AKT2,AXIN1,BAP1,BCOR,CD274,CREBBP,ERCC5,FAT1,GATA1,GNAQ,HIST1H3G,IGF1R,IRS2,JAK1,LATS1,PIK3CA,PPP6C,RNF43,RPS6KA4,TOP1	0.174732424	1.258295024
path_864	ASXL1,AXL,BCL2L11,CHEK1,CRLF2,ERCC2,ERG,FLT1,HIST1H3J,HIST3H3,INPP4B,LATS1,NBN,PIK3C3,PMS1,RAD21,REL,RNF43,SMARCA4,STAT3	0.087822585	1.344263367
path_865	BCL2,CTCF,CUL3,EPHB1,FGFR1,GRIN2A,HOXB13,IFNGR1,INSRR,KDM6A,KRAS,MAP3K1,PALB2,PIK3CB,RHOA,RPTOR,SDHAF2,SH2D1A,SOX2,TP53	0.909908271	0.980637294
path_866	ALOX12B,BCOR,CENPA,CSF3R,DIS3,DNMT3A,ERCC2,HIST1H3B,IGF1R,INSR,KRAS,MGA,PALB2,PARP1,RAC2,RHEB,SMAD2,SMAD3,SUFU,TSC1	0.762295126	1.045255897
path_867	AKT2,CCND2,CEBPA,CRLF2,CTNNB1,EIF1AX,EPHB1,ERBB3,FLT3,FOXO1,GATA3,H3F3C,IGF1R,MAX,NUF2,PAK7,PLK2,PRDM1,PTPRT,RAD51	0.220163504	1.23505139
path_868	AKT1,ARAF,ATM,CDKN1B,CRLF2,CUL3,E2F3,EIF1AX,FGFR2,HIST1H3B,IGF1R,INPP4A,IRF4,MDM4,PTPRT,RAD21,RTEL1,SDHC,SOX2,TRAF7	0.519213544	1.129768827
path_869	AR,ARAF,ARID5B,BAP1,BBC3,CDH1,CTNNB1,FGFR3,JUN,KEAP1,LATS1,MALT1,MPL,MYC,NCOA3,NCOR1,NOTCH1,NRAS,RUNX1,SMARCB1	0.367689074	1.146238819
path_870	ALOX12B,ARID2,BBC3,CCND3,CDK8,EIF4E,ERBB4,FANCA,HIST1H3I,HLA-A,KLF4,PAK7,PTPRS,RAC2,RAD51B,RET,STK11,TAP2,TET2,YAP1	0.247799737	1.194203606

path_871	ACVR1,CDH1,CDK6,CTCF,ERG,ERRFI1,FAM46C,FANCA,GNA11,GRIN2A,IRS1,KEAP1,NCOA3,NSD1,PDGFRB,PTCH1,PTPRD,RUNX1,STAT3,STAT5B	0.087953706	1.299780332
path_872	ALK,ASXL2,ATR,AXIN2,BARD1,BCL10,CSF1R,DNMT3A,EED,ERBB2,GATA2,HIST1H3B,MAP2K2,NFE2L2,NUF2,PBRM1,PHOX2B,PIK3R2,ROS1,SDHAF2	0.57255805	1.095842212
path_873	ASXL2,BRAF,CD79B,CDC42,EGFR,EZH2,FYN,GNA11,GSK3B,MAP2K1,MITF,MPL,MST1,NPM1,PNRC1,PTPRS,RASA1,RPTOR,SOS1,TEK	0.824740451	1.031800826
path_874	BRCA1,CARM1,CIC,CRLF2,CTNNB1,CXCR4,DIS3,GATA2,IL7R,MAX,PAK1,PGR,POLD1,RAD54L,RECQL4,RHEB,RPS6KB2,SDHA,SDHC,SMAD3	0.067697021	1.429559905
path_875	APC,ARID1A,ERBB2,FAM175A,FGFR1,GATA3,GNAS,HGF,IFNGR1,IRF4,IRS1,IRS2,MALT1,MET,MGA,MLL3,MSH2,MTOR,TCF7L2,TET1	0.22844719	1.195936111
path_876	AKT1,BCL10,CTNNB1,EED,EIF4A2,ERRFI1,FOXA1,HIST1H1C,HLA-A,IGF2,MDM2,MEN1,MTOR,PAK7,PIK3R3,PTPRS,RARA,RNF43,TSHR,U2AF1	0.188580201	1.203667869
path_877	BCOR,CARM1,CDK12,CDK6,CHEK2,ERCC2,FOXL2,FOXO1,HOXB13,IDH1,IL10,IRS2,MDM2,MSH2,RARA,REL,SDHB,SHQ1,SMAD4,SMO	0.492084408	0.907917126
path_878	ABL1,ALK,ASXL2,ATR,BCL10,BRD4,CBL,HIST1H3A,IDH1,IL7R,INPP4B,IRF4,MUTYH,PIK3CB,PLK2,RUNX1,SDHB,SDHC,STAT5B,TEK	0.435100635	1.117356087
path_879	CDK4,CDK6,CSF1R,DAXX,EPHB1,FOXL2,GNA11,GRIN2A,HOXB13,IRF4,NOTCH4,PIK3CA,RAD50,RAD51D,REL,SOCS1,SYK,TCF7L2,TEK,TP53BP1	0.740447887	0.954123358
path_880	BCOR,CCNE1,CDC73,CIC,EIF4A2,EPHA5,ERG,GLI1,HIST1H3J,KLF4,MGA,MRE11A,NEGR1,PDCD1,PTPN11,REL,SRC,SRSF2,TET2,TNFAIP3	0.073217384	1.366920688
path_881	ABL1,BRCA2,BRD4,CARM1,CEBPA,CSF3R,CTCF,EPHA5,EZH2,FANCA,HIST1H3E,INPP4B,MSH2,MSH6,PTPN11,RAD51,RAF1,SOCS1,SYK,TCF3	0.160083715	1.220059695
path_882	ARID1A,ARID5B,B2M,BBC3,BRIP1,CDC73,ERRFI1,FANCA,FANCC,GNA11,IGF1R,MAP3K1,MSH6,NKX3-1,PAK1,RB1,RBM10,SOS1,TGFBR1,TP63	0.125751459	1.268350124
path_883	AKT3,ARID2,BRCA1,CALR,CCND3,CXCR4,FANCA,FGFR2,FGFR3,GNAS,GPS2,HIST1H3C,KDM5A,MAX,MST1R,PIK3C2G,PIK3R1,SPOP,TERT,TP53	0.280624696	1.169568445
path_884	ARID1A,ASXL1,ATR,BTK,CCND1,CENPA,CIC,ERBB4,FANCA,FOXL2,FUBP1,IKZF1,INHA,INSRR,IRF4,MPL,PIK3C2G,ROS1,SDHA,YES1	0.112640207	1.307695706
path_885	ARID1B,ATM,BCL10,CARD11,CDK4,EP300,ERCC5,FBXW7,FLT1,IRS2,LATS2,MAP3K13,NBN,NOTCH2,PARK2,RAD51B,SHQ1,SMAD2,SRC,TAP2	0.823570107	1.037720803

path_886	ABL1,ALOX12B,BCL6,BCOR,BRAF,CARD11,ELF3,FGFR4,HGF,HIST1H3A,IRF4,MCL1,MTOR,NTRK1,PLCG2,PNRC1,PTPN11,SMAD2,TCF3,TEK	0.369197715	1.136705475
path_887	ARAF,BLM,BRIP1,CBL,CD276,CDK12,CIC,ERBB3,GSK3B,HIST3H3,INSR,KMT2D,NEGR1,RAD51D,RAD54L,RPS6KA4,RPTOR,STAT3,STAT5B,TGFBR1	0.952945942	1.008476502
path_888	ASXL2,FGF3,HIST1H3H,IGF1R,INHA,INPP4A,KDM5A,MCL1,NF2,NPM1,NUP93,PIK3R2,PPM1D,PREX2,RARA,RNF43,RPS6KB2,SDHC,STK11,TBX3	0.098996962	1.318006744
path_889	ARAF,CALR,CUL3,CXCR4,ERCC5,FGF19,FH,FOXO1,HIST1H3D,IGF1R,INPP4A,JAK2,KNSTRN,MLL2,NF2,NFE2L2,NOTCH4,PIK3CG,PMAIP1,PPP6C	0.495760134	1.11412316
path_890	ALOX12B,BCL2,BRAF,CCND3,DDR2,DROSHA,ERG,FAT1,HIST1H3J,IFNGR1,IL7R,IRF4,KIT,MAP2K2,PIK3CB,PLK2,PTPRT,RICTOR,ROS1,SOS1	0.189998618	1.236526473
path_891	ATR,AXIN1,BRD4,CDK4,CHEK2,CREBBP,ERBB4,INHBA,JAK1,LATS2,MITF,PDGFRB,PIK3CG,PIK3R1,PIM1,PMS1,SDHAF2,SDHB,SMAD4,TRAF2	0.292569572	1.192145978
path_892	ARID5B,BCL6,BTK,CARD11,CD276,CDK6,EPHA5,FOXP1,HIST1H3A,HIST1H3E,KMT2D,MYC,PARP1,PTPRD,SH2D1A,SMO,SOCS1,TCF3,TOP1,YAP1	0.241128579	1.202453538
path_893	AKT1,ALOX12B,ARID2,BAP1,BCL10,CCND1,CCND3,CDKN1B,CRLF2,CSF3R,DNMT3B,FBXW7,IL10,KRAS,MTOR,NF2,PREX2,RAD51B,SOX2,STAG2	0.197309698	1.226238755
path_894	AKT3,BCL6,BRCA1,DNMT3B,EP300,ETV6,FGFR1,FGFR3,HIST3H3,IDH1,INSRR,KMT2D,MAP2K4,MET,MGA,NBN,NOTCH4,RAD50,SDHAF2,TCF7L2	0.905064138	1.019989423
path_895	CDH1,DIS3,FANCA,FGF19,FOXA1,FOXL2,GATA1,GPS2,INHBA,INSRR,MEF2B,MST1R,NBN,PIK3C2G,PPP2R1A,PPP6C,RARA,SDHC,SMARCA4,TOP1	0.225101006	1.186794646
path_896	BBC3,BCL2L11,CD79B,CRLF2,CSF3R,EIF4A2,ETV1,FBXW7,IRF4,KMT2A,MLL2,PMS1,PPP6C,RARA,RB1,RICTOR,ROS1,SUFU,TCF3,TRAF7	0.508779469	1.13687124
path_897	BCL2L1,BRIP1,CCND2,CHEK2,E2F3,ERBB3,ERBB4,FGF19,GNAQ,HIST1H3C,INSRR,MLL2,NKX3-1,NOTCH1,RPS6KA4,RPTOR,RYBP,SF3B1,SLX4,STAT5B	0.334705665	1.190748123
path_898	ANKRD11,APC,ASXL1,CHEK1,DROSHA,EPHA5,EPHB1,HIST1H3H,IDH1,JUN,MST1R,NFE2L2,NFKBIA,PIK3CA,PIK3R3,SMAD2,SMAD4,SMARCD1,SOX9,TGFBR1	0.293332087	1.184832139
path_899	ABL1,AXIN1,BCL2,BCL2L1,CCND3,CDC42,CDH1,CDK6,DNMT1,ETV6,HIST1H3H,IGF1R,IL7R,KLF4,MPL,NOTCH2,RBM10,RPTOR,RUNX1,SDHA	0.398455289	1.126494266

path_900	BMPR1A,BRCA1,CDC42,CUL3,DNAJB1,E2F3,EIF4E,ESR1,ETV6,FOXA1,HIST3H3,IRS2,MCL1,NFKBIA,NUP93,PRDM1,RAD51D,RECQL4,RUNX1,STAG2	0.204042015	1.201844409
path_901	ARID5B,ATR,CALR,DROSHA,EPHA5,ETV6,FGF19,FOXO1,HGF,INPP4A,MET,MGA,MSH2,MYCN,PIK3R3,PMS2,PPP6C,RAD51,RAD51B,RHOA	0.068817859	1.363056415
path_902	ASXL1,BARD1,CARM1,CXCR4,DNAJB1,E2F3,FAM175A,FAM46C,FLT1,FOXA1,FOXO1,GNA11,H3F3C,HIST1H3C,HIST1H3E,IL10,INHA,PMAIP1,RAD51D,SH2D1A	0.919210011	1.014593508
path_903	BBC3,BCL2L1,BCL6,CD276,DICER1,ERCC5,FANCA,FGFR2,FOXA1,GATA1,JUN,KDM5C,MLL2,MST1R,NEGR1,PALB2,PTPRT,RAD51B,SMARCB1,STK40	0.071998441	1.383069334
path_904	AMER1,CDK12,CDKN2A,FAM175A,FGFR2,FOXP1,GLI1,GRIN2A,INSRR,KRAS,MYCL1,NBN,PDGFRB,PIK3C3,PIK3CD,PTEN,PTPRD,RBM10,RHOA,SRC	0.44994159	1.130587394
path_905	ARID5B,B2M,CTLA4,ERBB3,EZH2,FOXO1,GNAS,H3F3C,IDH1,MAX,MEF2B,MLL,NF2,NUF2,PIK3CB,PIK3R2,RAD51C,SETD2,SPOP,TERT	0.128188773	1.239855871
path_906	ARID1A,CSF3R,EIF4E,FANCA,FAT1,FLT3,GNAS,HOXB13,IFNGR1,IGF2,KDM6A,MGA,NEGR1,NFKBIA,NKX3-1,NUF2,PIK3CB,PNRC1,REL,SPEN	0.241608397	1.21242899
path_907	ACVR1,BBC3,BCL2L11,ERG,FAM175A,FGF3,FGFR1,HLA-A,JUN,MAP2K2,MLL3,MYC,MYCN,NFKBIA,PIK3CA,PPM1D,RARA,REL,SMAD3,SOX2	0.083033246	1.276687704
path_908	ARAF,B2M,BARD1,BCL6,BRAF,CDK4,CHEK2,CRLF2,ERBB4,ERCC2,FUBP1,HIST1H3I,HLA-A,KMT2A,NCOR1,NKX2-1,PTPRS,RAD51,RB1,SHQ1	0.17210566	1.214872131
path_909	ARID1A,AXIN1,BRAF,CASP8,GNAS,HIST1H3C,INHBA,INPP4B,JAK3,MAX,PDCD1,PHOX2B,PIK3CA,POLD1,RAD21,RAD54L,RPS6KB2,RUNX1,SETD2,SRC	0.176140107	1.247161427
path_910	ARID1B,B2M,BARD1,BRCA1,CD274,FGFR3,GATA3,HIST1H3D,HIST1H3I,ICOSLG,IDH1,KDM6A,MAP2K4,MDC1,MRE11A,NUF2,PIK3CD,PRDM1,RAD51,RARA	0.625491327	1.073232391
path_911	ABL1,CTNNB1,DNMT3A,EIF4E,ERBB3,FANCA,FGF19,HIST3H3,HOXB13,IRF4,LATS1,MYC,MYOD1,NCOA3,NEGR1,NFKBIA,PTCH1,RAD51,RUNX1,SOX9	0.835793622	1.030126881
path_912	AKT1,ANKRD11,CARM1,CASP8,CCND3,CTLA4,FGF19,GATA2,IL7R,IRF4,JAK2,MDM2,MET,MLL,NKX3-1,PREX2,PTCH1,RAF1,SMARCA4,SPOP	0.079312176	1.423130307
path_913	ATM,AXIN2,BCL10,CALR,DROSHA,EPHA7,FGFR3,FYN,GNA11,HIST3H3,ICOSLG,PAK7,PPM1D,REL,RHEB,SH2D1A,SHQ1,SOX2,SRSF2,TP53BP1	0.37544044	1.133956893

path_914	AKT1,BRCA1,CCND2,CCNE1,CDK12,CHEK2,DAXX,DIS3,EPHA5,ERCC2,FGF4,IGF1R,MAX,MCL1,MRE11A,NTRK1,RARA,RECQL4,SDHB,SPEN	0.453616021	1.154141156
path_915	CDC42,CHEK1,DIS3,DROSHA,EPHA3,FLT3,FOXO1,FYN,GNAS,IDH1,IGF1R,LATS1,MDM4,MST1,MTOR,MYCN,PIK3R2,RAD51C,SETD2,SH2B3	0.098184817	1.344002776
path_916	ARID1B,BCL2L11,BRAF,CHEK1,CSF1R,FANCA,GLI1,IKBKE,MPL,MST1R,NUP93,PGR,PIK3CG,PNRC1,SF3B1,SHQ1,SMAD4,SMO,SRC,TEK	0.647312571	1.080035342
path_917	AMER1,APC,ARID5B,AXL,BBC3,CRLF2,EPHB1,FAM46C,FUBP1,GLI1,HIST1H3J,IGF2,IKBKE,IRS1,MST1R,NEGR1,PLCG2,PNRC1,RAD51,SETD2	0.858303929	1.025565582
path_918	ANKRD11,CDC42,CTNNB1,DDR2,E2F3,EIF1AX,FAT1,GATA1,GNA11,HIST1H3E,IL7R,MLL,MLL3,MSH3,NCOR1,NOTCH3,PPP2R1A,RECQL4,RHOA,TSC1	0.206929445	1.23611248
path_919	ACVR1,AMER1,ATR,BMPR1A,BRIP1,CHEK1,CSF3R,EIF4A2,FYN,KDR,MSH6,MUTYH,MYCL1,NTRK2,PIK3R1,PRDM1,PTPN11,RHEB,SRSF2,TEK	0.483675335	1.106431232
path_920	ANKRD11,ARID1B,ASXL1,BRCA1,CHEK1,DDR2,FGFR3,FLT1,GATA1,IRF4,MYC,MYCL1,NUP93,PMAIP1,RECQL4,RET,RYBP,SH2B3,SOCS1,SRC	0.983241684	0.99703383
path_921	ALK,BRIP1,CCNE1,CHEK2,EIF4E,EPHA3,ERBB3,ERCC4,ERG,GATA1,GSK3B,HIST1H1C,HIST3H3,IRS2,JAK3,PIK3C3,PMAIP1,PRDM1,RHOA,RPS6KB2	0.086317962	1.357365721
path_922	ABL1,CBL,CDK6,FGFR2,FGFR3,FLT1,IGF2,IKBKE,MALT1,MLL2,MLL3,MSH2,NF1,NRAS,PIK3CD,PMAIP1,PREX2,RBM10,REL,SOS1	0.770294376	1.046013219
path_923	AKT2,ASXL2,ATR,BRD4,CASP8,CD274,CDK8,DAXX,EIF1AX,EPHB1,FGFR1,HIST1H3A,INHA,NSD1,NTRK3,PTPRS,RAD51C,RAD51D,RECQL4,RET	0.315910658	1.152689643
path_924	AR,ARAF,AXL,BCOR,CCNE1,FANCA,GNAS,HNF1A,JAK3,KLF4,KRAS,MGA,MPL,MRE11A,MUTYH,PMAIP1,PPP2R1A,RAD51B,RHOA,SRC	0.171443401	1.232295617
path_925	BRCA1,BRD4,CCND2,DNAJB1,EPHB1,FLT1,FOXA1,JAK2,JAK3,JUN,MAP2K1,NKX2-1,NSD1,PLCG2,PMAIP1,RYBP,SMAD2,SRC,STAT5B,STK40	0.705114596	1.055817905
path_926	ABL1,AR,DIS3,DOT1L,EPHA7,ERBB4,ESR1,GNAQ,GNAS,IFNGR1,IGF2,MLL2,MSH2,MST1,PMS2,PTPN11,SOX2,STAG2,STK40,TOP1	0.107605226	1.302397092
path_927	ACVR1,BMPR1A,CDK8,CIC,CREBBP,CTNNB1,CUL3,GNA11,GRIN2A,GSK3B,HIST1H3A,HIST1H3C,JAK3,MAP3K1,MYC,NCOA3,PIK3C3,PPP2R1A,RAD21,SMAD2	0.204650852	1.263908565
path_928	BCL2,BRAF,CDK4,CDKN2A,CREBBP,CUL3,DNAJB1,GATA3,IDH1,JAK3,JUN,KEAP1,MDC1,NFE2L2,NOTCH1,PARP1,PTCH1,ROS1,SETD2,SRC	0.462138224	1.111817318

path_929	BBC3,BCL10,DOT1L,ERBB2,FAM175A,FOXP1,LATS1,MALT1,MDM4,NRAS,PAK7,PMS2,POLD1,RTEL1,RYBP,SDHAF2,SETD2,SMO,STAT5B,TBX3	0.440678894	0.874710001
path_930	AKT3,ALOX12B,BCL6,BMPR1A,EIF4E,ETV6,HIST1H3I,IFNGR1,INPP4A,JAK1,KIT,MTOR,NFKBIA,NTRK3,PGR,PREX2,RET,RYBP,SDHAF2,SH2D1A	0.096955665	1.269287988
path_931	BCL6,CDK6,DIS3,ERCC4,ERCC5,FOXL2,FYN,HIST1H3B,HOXB13,JAK1,MAP3K13,MDC1,MDM2,MUTYH,PAK1,RAD51D,SH2B3,SRC,TAP2,TRAF2	0.409845733	1.126515917
path_932	AKT2,ALOX12B,AR,ARAF,ARID5B,BRIP1,CALR,DNAJB1,ERBB2,FBXW7,FGF4,INSRR,IRS1,JAK1,MDM4,MED12,NEGR1,NFKBIA,PIK3C2G,SDHC	0.502346117	1.099975432
path_933	ALOX12B,ANKRD11,ARAF,BCL10,CASP8,DNMT1,FOXL2,GSK3B,INHA,KRAS,MAP2K2,MITF,MPL,MST1R,PIK3R3,PMS1,RASA1,RPS6KA4,RTEL1,STK11	0.785413284	0.961952549
path_934	AKT1,ALK,ARID5B,BCL2L1,CDK12,CDK6,CDKN1B,CTNNB1,ERBB2,ERBB4,FLT1,ICOSLG,KDM5A,MAX,MDM2,NF1,PPP6C,PTCH1,PTPRT,RHEB	0.668907664	1.066092479
path_935	ASXL1,E2F3,ERCC5,GNAS,GSK3B,HIST1H3I,ICOSLG,MLL2,MTOR,NBN,NOTCH3,PARK2,RAD54L,RASA1,RBM10,ROS1,RPS6KB2,SF3B1,STAT5B,TP53BP1	0.057899749	1.385098107
path_936	AR,BAP1,BTK,CSF1R,DNMT1,ERG,FAT1,FGF19,GATA3,GNA11,HOXB13,KMT2D,MRE11A,NF2,NFE2L2,NKX3-1,NUF2,PHOX2B,PIM1,RNF43	0.177484185	1.209279613
path_937	AKT2,AURKA,BCOR,CDKN2A,CIC,CREBBP,EZH2,FAM46C,FGF4,HIST3H3,IRS1,KNSTRN,PIK3CA,PMS1,RET,SDHA,SH2B3,SH2D1A,SMO,TBX3	0.404087567	1.156288755
path_938	ABL1,AXL,BRIP1,CARD11,ERBB3,ERCC5,FAT1,GNAQ,HIST1H3G,MLL2,NOTCH3,PAK1,PIK3R3,POLD1,RAD51D,RAF1,ROS1,SH2D1A,TGFBR1,TP53	0.330524279	1.147124415
path_939	AKT3,BCOR,CALR,CDKN2A,CEBPA,CXCR4,EIF4A2,EP300,ERCC4,HIST1H3B,IRS1,MITF,MSH2,MST1R,NOTCH1,PTPRT,RAD51D,SMARCA4,SOX17,TRAF2	0.15720087	1.240738336
path_940	ARID5B,ATR,CCND1,CDK4,CDKN2A,CRLF2,DNMT3A,E2F3,GNAQ,IL7R,JUN,MDM4,MED12,NOTCH1,PAK1,PMAIP1,PREX2,SH2D1A,SOX9,TAP1	0.632619213	1.094759886
path_941	BRIP1,CD79B,CSF3R,DNMT1,DNMT3B,ERCC4,FANCC,FGFR4,FLT1,KMT2A,MDM4,MYC,NKX3-1,NOTCH3,PIK3R1,PMS2,RET,SMAD3,SRSF2,TOP1	0.273511483	1.166960114
path_942	ALOX12B,DOT1L,DROSHA,ERCC2,EZH2,FANCA,FBXW7,HGF,HIST3H3,IDH1,MTOR,POLD1,RAD21,RARA,RHEB,RICTOR,SDHA,SLX4,TBX3,TP53	0.680384757	1.061832897

path_943	ASXL2,BBC3,BRD4,CARD11,CASP8,FGFR1,GSK3B,HLA-A,IDH1,IRS2,KDM5C,LATS2,MAP2K1,MTOR,MYCL1,NEGR1,PIK3R1,PIK3R2,RAD21,TET2	0.122765372	1.33235874
path_944	AKT2,ASXL1,CARD11,CARM1,CDH1,EIF1AX,FOXA1,IGF2,INSR,IRF4,KRAS,MEN1,MGA,MUTYH,NUF2,PIK3R1,PTPRD,RAD21,RAD51B,RARA	0.295307268	1.161253025
path_945	APC,ATM,BLM,BRCA1,BTK,CDKN2A,CTLA4,EGFR,FAT1,FUBP1,GNAS,HIST1H3A,HOXB13,KMT2D,KRAS,PMS2,RAD51B,RICTOR,RPTOR,RUNX1	0.969760222	0.994654592
path_946	ATM,BCL10,BRCA2,CDK12,CDK4,CDK6,DOT1L,FANCA,FGFR1,GNAS,IGF1R,JAK1,JUN,LATS2,PDGFRA,PIK3CA,PIK3CD,PNRC1,RAD54L,RPS6KB2	0.646869178	1.079429002
path_947	ARID5B,ASXL1,B2M,BCL2L1,CDC42,CDKN2C,EP300,FBXW7,FGF4,FGFR3,INPP4A,MAP3K13,MYOD1,NPM1,PARK2,PPM1D,PTCH1,PTEN,RFWD2,RPTOR	0.3263658	1.150104824
path_948	CCND3,CDK6,E2F3,EED,FANCC,FGF4,FUBP1,HLA-A,IRS2,JAK2,KDM5C,MCL1,PHOX2B,PIM1,RBM10,RYBP,SDHC,SOX9,SYK,TBX3	0.482952494	1.104309186
path_949	AKT3,ATM,AXL,CENPA,CRLF2,FGFR4,FOXA1,FOXP1,FUBP1,IDH1,LATS2,MST1,PLCG2,PTCH1,RAD51D,RPS6KB2,RUNX1,SDHA,SH2D1A,SRC	0.627451524	1.070814038
path_950	ABL1,ALOX12B,BBC3,BCL2L11,CCND3,CENPA,CIC,CUL3,EED,ERBB4,ETV1,ETV6,FOXL2,GNAQ,KDR,MDM2,MYC,PRDM1,RASA1,SOCS1	0.125692956	1.246971851
path_951	ARID1B,ASXL1,CD274,CEBPA,DNAJB1,GSK3B,IKZF1,IRF4,KIT,MYC,NF2,NOTCH4,NTRK2,PIK3C2G,PIK3CB,PMAIP1,RAC2,RAD51C,RAD51D,RTEL1	0.297685893	1.212086919
path_952	ABL1,BCOR,CDK12,CDK6,CUL3,ERBB3,FAM175A,FGFR1,FUBP1,KEAP1,KMT2A,KRAS,MSH2,MYOD1,NBN,NCOR1,PALB2,PDGFRA,SDHAF2,SF3B1	0.191913793	1.252013001
path_953	BAP1,BRCA1,CASP8,CTNNB1,ERCC4,FGF4,FH,FUBP1,KMT2D,LATS1,NKX3-1,PARK2,PIK3C3,POLD1,RECQL4,RHOA,SF3B1,SH2D1A,SOX2,SRSF2	0.088483705	1.271344932
path_954	AXIN2,CASP8,CD276,CEBPA,ERRFI1,HOXB13,KDM6A,KRAS,MLL,MST1R,NUF2,PGR,RHOA,RTEL1,RYBP,SRC,STAT5B,TAP2,TNFAIP3,TOP1	0.074199506	1.362947249
path_955	AMER1,AR,CDK8,ERCC4,FGF4,GATA3,GNA11,GNAS,GRIN2A,HNF1A,INPP4A,MAP2K1,PGR,RAD51C,REL,RHEB,SUFU,TAP1,TBX3,WT1	0.427877539	1.149578855
path_956	ABL1,ACVR1,BAP1,BRCA1,CCND2,CREBBP,ERG,IRF4,JAK1,KNSTRN,MAX,MYCN,NUF2,PDGFRB,PNRC1,PPP6C,RAD51D,SF3B1,SMAD3,STK40	0.070851684	1.292673025

path_957	ARAF,BAP1,CDH1,CEBPA,FAT1,FUBP1,FYN,HIST1H3G,HLA-A,HOXB13,INHA,IRS1,MAP3K13,MUTYH,PLCG2,POLD1,PPP2R1A,SDHC,SETD2,SMARCD1	0.484291194	1.104528612
path_958	AMER1,ARID5B,ATM,B2M,CCND3,CDKN1A,CXCR4,DICER1,EP300,FGF3,GNAS,HIST1H1C,HLA-A,KDR,NF1,PDGFRA,PIK3R1,RB1,RHOA,SRC	0.602495525	1.084183437
path_959	ABL1,AKT2,AKT3,BRAF,BRCA1,CTLA4,EPHB1,FANCC,GATA1,GNAQ,GPS2,IGF2,MCL1,MGA,MLL,MYCL1,NFE2L2,NFKBIA,PALB2,PDGFRB	0.402859058	1.168703432
path_960	BCL10,CDC73,DIS3,ELF3,EPHA7,ERBB2,FLT4,FOXA1,FUBP1,GNAS,MAP3K13,MSH3,NBN,PIK3C3,RAD51D,RPTOR,SDHB,SMAD2,SMAD3,TGFBR1	0.414410918	1.123650799
path_961	ASXL1,CARM1,CD274,CSF3R,DOT1L,EED,FOXA1,GNA11,IDH1,IFNGR1,KDR,KEAP1,LATS1,MAP2K1,MET,MLL,MUTYH,NUF2,PIM1,PTPRT	0.127271503	1.243716995
path_962	ATR,AXIN1,CDK6,DNAJB1,FH,FYN,GATA3,KRAS,LATS2,MAP3K1,MAX,NTRK1,NUF2,PALB2,PGR,RASA1,RFWD2,SLX4,STK40,SYK	0.31480557	1.15861866
path_963	ALK,BRAF,CCND1,CUL3,DAXX,ELF3,EPHA3,EZH2,FYN,HLA-A,IGF1R,IRS2,MAP3K13,NEGR1,PAK7,POLD1,POLE,PTPN11,SDHAF2,TCF3	0.067896647	1.365417444
path_964	ABL1,AXIN2,CDK4,CREBBP,EPHA3,EPHA5,HIST1H1C,HIST1H3D,HIST1H3J,IRS1,KLF4,KMT2D,MDM2,MLL,NOTCH1,RTEL1,SOX2,SUFU,TCF7L2,TP53BP1	0.103808756	1.305074533
path_965	BCL6,CRLF2,CTCF,DICER1,ERBB2,ERCC2,FAT1,FYN,KLF4,MPL,NBN,PLCG2,PMS2,RAC2,RB1,RHOA,RPTOR,STK11,TET2,TGFBR1	0.256629111	1.193275912
path_966	ANKRD11,ARID1A,CDK4,CDKN2C,CHEK1,FANCC,FGF4,FH,H3F3C,NF2,PAK1,PALB2,PIK3CD,PLK2,RAD51C,RET,SF3B1,SLX4,SPOP,STAT3	0.646478564	1.067057504
path_967	ANKRD11,AXL,CD276,CDC73,CIC,CRLF2,CSF1R,ERBB2,GATA2,HIST1H3G,HLA-A,INHA,RECQL4,RHOA,RTEL1,SDHA,SDHC,SOX2,TET1,TRAF2	0.064358624	1.309243148
path_968	BCL2L1,BRCA1,CBL,CCND2,CD276,ERBB2,FOXO1,FYN,HIST1H3D,HIST1H3G,HNF1A,IDH1,IKBKE,IRS1,MST1,PLK2,PTCH1,RAD54L,RHOA,SH2B3	0.462822397	0.901059051
path_969	ATM,ATR,BRD4,CCNE1,CDK12,EIF4A2,FOXP1,HIST1H3J,MAP3K13,MYOD1,NF2,NRAS,PHOX2B,PMAIP1,PTPRT,RAD21,SMO,TBX3,TEK,YES1	0.066754875	1.344561305
path_970	ACVR1,CALR,CTNNB1,ERBB4,ESR1,ETV1,GPS2,GSK3B,INSR,KRAS,MDC1,MGA,NCOR1,NUF2,PLK2,RB1,RHEB,SDHA,SDHAF2,SMARCD1	0.264281205	1.172285633

path_971	ASXL1,AURKA,BCL2L11,BCL6,ERBB2,H3F3C,HIST1H3A,HLA-A,KDM5C,MRE11A,PIK3R2,PIK3R3,PMS1,PRDM1,PTPRD,REL,RPS6KB2,STAG2,TEK,TOP1	0.085874365	1.362204258
path_972	APC,ARID1B,ARID2,AXL,BCL2,CSF3R,ELF3,ERBB4,IRF4,JAK2,JUN,MYC,NSD1,NUF2,PDCD1,PIK3R1,PNRC1,RAD51,RB1,TMPRSS2	0.55488441	1.108358662
path_973	ABL1,ASXL1,AURKA,BCL2,CENPA,CSF1R,CSF3R,CUL3,DAXX,ETV6,FAT1,FGF3,JAK1,MDC1,MRE11A,PDGFRA,POLE,RNF43,RYBP,STAG2	0.119661177	1.284872056
path_974	AKT3,ANKRD11,APC,BCL2L11,CARM1,CDH1,CEBPA,ERG,FGFR2,HGF,HNF1A,IL10,LATS2,PIK3C2G,PMS1,PPP2R1A,RASA1,RFWD2,RPS6KA4,RTEL1	0.365311765	1.13670673
path_975	AXIN1,B2M,CD79B,CDK12,CSF1R,FH,FYN,H3F3C,HOXB13,ICOSLG,MAX,MSH6,NOTCH1,NUF2,PIK3CB,PREX2,RAD50,RAD51,RAD51C,SUFU	0.770665315	0.958220069
path_976	ABL1,ARID1A,AXIN1,CARM1,DNAJB1,EIF4E,FAM46C,FGF3,FOXA1,FUBP1,GRIN2A,HNF1A,INSRR,IRS1,JAK1,KDM6A,MEN1,MLL,MLL3,NCOR1	0.220456236	1.211241055
path_977	ARID1B,BARD1,BCL2L11,CALR,CARM1,CHEK1,EIF4A2,EPHA7,ERCC2,HIST1H3D,IFNGR1,JAK1,MST1,NTRK2,SH2B3,SOCS1,SOX9,STAT5B,TET1,TNFAIP3	0.076173004	1.294357617
path_978	ALK,APC,ASXL1,AURKA,BMPR1A,CARM1,CENPA,DDR2,EPHA7,FANCA,GRIN2A,HIST1H3C,IRS1,KDM5C,KEAP1,MSH3,NCOR1,NFKBIA,SMO,SOX2	0.345761069	1.146111273
path_979	ALK,BARD1,CDKN2A,DNMT1,EIF1AX,EPHB1,ERCC5,ERG,ICOSLG,IKZF1,KDM5A,KDM5C,MAP2K4,MGA,MPL,MSH3,MYC,PTPN11,RAD54L,RPS6KA4	0.211016698	1.243050088
path_980	ABL1,BTK,CD274,ERG,ERRFI1,FLT1,HGF,HLA-A,IKBKE,KLF4,MLL,MUTYH,NTRK3,PMS2,PRDM1,PTPRD,RAC2,RAD51D,STAT3,SYK	0.122207171	1.295505086
path_981	BCL10,ERG,FANCC,FH,GATA1,GNAS,INHBA,NFE2L2,NUP93,PARK2,PDCD1,PTPRD,RAD51C,RARA,RYBP,SDHA,SH2B3,SOCS1,SUFU,YAP1	0.050986198	1.31455664
path_982	ANKRD11,BCL2,EGFR,EPHA7,ERCC4,ETV6,FOXA1,HIST1H3G,HNF1A,IGF2,KNSTRN,KRAS,MALT1,NOTCH3,PARP1,PLK2,PMS2,RNF43,RYBP,TET2	0.696111338	0.945931223
path_983	ARID2,BRD4,CARD11,CBL,CD276,CDC42,DNMT3A,EP300,HNF1A,IRS1,JUN,MEF2B,MET,MSH6,NPM1,PAK1,PMS1,PTPRS,SOX17,STAT3	0.318546063	1.151077748
path_984	ARAF,CYSLTR2,DAXX,DNMT3B,EIF1AX,EPHA5,HIST1H3C,INHA,JAK3,KDM5A,KDM5C,MDC1,MITF,NTRK3,PDGFRA,PIK3C3,PIK3CG,PIK3R1,RPS6KB2,SMARCA4	0.297736039	1.169257095

path_985	AURKA,CD274,CDK8,CTLA4,ERCC2,FGFR2,ICOSLG,KDM5C,KDR,MET,MLL3,NUF2,PAK1,PAX5,PIK3R3,RASA1,RECQL4,SDHA,SH2D1A,SLX4	0.078373541	1.388303188
path_986	CDK4,CXCR4,HIST1H3H,IFNGR1,IGF1R,IRF4,KLF4,LATS2,MAP3K13,NEGR1,NUF2,RAD51B,RAD54L,RPTOR,SETD2,SLX4,SRSF2,TGFBR2,XPO1,YES1	0.432263608	1.118711917
path_987	ACVR1,ALOX12B,BRAF,CSF3R,DNAJB1,DNMT1,FOXO1,GATA1,GLI1,GNA11,GPS2,IKBKE,INHA,INHBA,MDC1,MITF,MTOR,MYOD1,NBN,PGR	0.562229543	1.085209584
path_988	AKT2,ARID5B,ATRX,BCL2L1,BCL6,CSF3R,EPHA5,FGFR3,GLI1,GNAS,HIST1H3I,IGF1R,MAP2K4,MCL1,MDM2,NKX2-1,PTPN11,RAC2,RAD54L,SDHB	0.30283318	1.156252404
path_989	AXIN1,BCL2L1,CALR,CCNE1,CDKN2C,CTLA4,EGFR,EPHA5,ERBB2,GRIN2A,HNF1A,JAK1,LATS2,MDM4,PIK3C2G,RHOA,SRC,TAP1,TCF7L2,TERT	0.997388394	0.99946101
path_990	FLT1,GATA2,H3F3C,HIST1H3B,HIST1H3H,INHBA,INSR,IRS1,IRS2,MDC1,MSH3,NOTCH2,PARK2,PIK3C2G,PIK3R3,RAD54L,ROS1,SMARCB1,SPOP,TEK	0.25179948	1.220660299
path_991	APC,ARID1A,ARID2,AURKA,BAP1,BCL10,CDKN2A,EED,FANCA,FLT3,GLI1,GNA11,KDM5C,KMT2D,NOTCH1,PTPN11,RAD21,RHOA,SMAD2,TCF3	0.417712907	1.136145715
path_992	CDKN1B,CREBBP,CUL3,DDR2,DOT1L,EED,ERBB4,FUBP1,HIST1H3B,JAK2,KLF4,MALT1,NEGR1,PBRM1,PLCG2,PPP2R1A,RARA,SOX9,SRSF2,STAT3	0.422626145	1.149797394
path_993	APC,ASXL1,BCL2L11,BCOR,BRCA1,CDK4,CDKN2C,E2F3,HGF,HIST1H3D,JUN,KMT2A,MDC1,MYCN,NEGR1,RAD51,RYBP,SDHA,SUFU,TAP2	0.112339825	1.343899418
path_994	AKT2,ARID5B,BRIP1,CHEK2,DIS3,ETV1,EZH2,FH,GPS2,HIST1H3I,KLF4,MAX,MGA,NRAS,PDCD1,PIM1,PLK2,RAD51C,SH2B3,SYK	0.295601911	1.158780729
path_995	ACVR1,ALOX12B,AMER1,ASXL1,BCL2L1,CDC73,EIF4E,EPHA5,ERBB3,FLT3,GNAS,HIST1H1C,MALT1,NOTCH4,PTPRS,RBM10,RECQL4,SRSF2,SUFU,TBX3	0.390251379	1.148786799
path_996	ACVR1,CDH1,CHEK2,CUL3,EIF4A2,ERBB2,FAM175A,FAT1,FGFR3,GLI1,HIST1H3B,KDR,MAP2K2,NCOA3,PMAIP1,RHOA,RNF43,STAT3,TAP2,TBX3	0.116995802	1.323371474
path_997	BTK,CD276,CDK8,CXCR4,ELF3,FLT4,GSK3B,IDH1,MGA,MLL,NCOR1,NPM1,PAX5,PIK3C2G,PTPRD,RAC2,RBM10,RNF43,SDHA,STK11	0.096859857	1.265724299
path_998	B2M,CDK6,CTNNB1,EIF4A2,EZH2,FBXW7,FOXO1,GATA3,HIST1H3C,HIST1H3H,IL7R,JAK3,KDM6A,PAX5,PIK3C2G,PMAIP1,RET,RPS6KB2,SF3B1,SOX17	0.778458495	1.040702232
path_999	CBL,CDK4,CDKN1A,DAXX,EP300,FLT1,H3F3C,IRS1,MEN1,MITF,MST1,MYC,NFE2L2,PARK2,RAD50,RB1,SETD2,SMARCA4,SOX17,STAT3	0.361015427	0.870739388

path_1000	AR,BLM,CCND2,EGFR,EIF1AX,GNAQ,IL10,MAP2K4,MDC1,MDM2,MPL,MSH3,MYOD1,NCOR1,PAK7,RHOA,RPTOR,SMO,STAT5B,TERT	0.707700085	0.939351272
path_1001	B2M,BARD1,BRD4,CD79B,CREBBP,CRLF2,ERBB2,H3F3C,IRF4,IRS2,MEN1,MUTYH,NF1,NSD1,RTEL1,SETD2,STAT3,SUFU,TAP1,TET1	0.167676117	1.263238667
path_1002	ABL1,AKT2,EIF1AX,GRIN2A,INSRR,IRS1,IRS2,LATS2,MAP3K13,MED12,MTOR,NRAS,NTRK1,PARP1,PRDM1,RPS6KA4,RYBP,SMARCB1,STK11,TEK	0.421041234	0.882682654
path_1003	ATM,CCND1,CD276,CENPA,DAXX,FGF19,GATA1,HIST1H1C,IFNGR1,INHBA,INPP4A,PDGFRB,PIK3C3,PIK3CG,RAD51B,RBM10,RET,SOS1,STAG2,STAT5B	0.684471154	0.9382726
path_1004	BRAF,BTK,DAXX,ERG,FANCC,FUBP1,GRIN2A,INHA,INHBA,JAK2,MYOD1,PAX5,PIK3CG,PNRC1,PPM1D,RPTOR,SOCS1,TET2,TP53,XPO1	0.513425651	1.096886715
path_1005	AKT2,ARID1B,ARID2,ATM,BARD1,BRCA1,CCND2,CENPA,DNMT3B,EPHA3,FLT1,KDM5A,KIT,KNSTRN,MDM4,MGA,MTOR,PGR,PIK3R2,PTPN11	0.0852329	1.294595761
path_1006	AKT2,AMER1,AURKA,BLM,CARM1,CDKN1A,DNMT3B,FGF4,IFNGR1,INPP4A,JAK1,KMT2A,MAP2K4,MLL,NF1,NTRK3,PIK3C2G,RPS6KB2,SH2B3,SOX17	0.071522532	1.36626231
path_1007	ARID2,BCL10,BRCA1,CD274,DICER1,EIF4E,FGFR1,HIST1H3C,IGF1R,IRF4,MAP3K13,MDC1,MDM4,MLL,NKX3-1,PARK2,RICTOR,SDHAF2,TBX3,TEK	0.393097508	1.129169876
path_1008	CYSLTR2,DDR2,FGF3,FLT3,FLT4,FYN,GLI1,HIST1H3B,JAK1,MALT1,MST1,NUP93,PBRM1,PGR,RAD51B,RTEL1,SDHC,SMARCB1,TBX3,TET2	0.368129645	1.164496349
path_1009	AKT2,CASP8,DNMT1,E2F3,FANCA,IRS1,NCOR1,PDGFRA,PIK3R1,PMAIP1,PNRC1,RAD21,SMARCD1,SOX2,SRC,TAP1,TGFBR2,TMPRSS2,TNFAIP3,TP53BP1	0.142544795	1.233084783
path_1010	ANKRD11,BMPR1A,CUL3,FLT4,KEAP1,MITF,MSH6,MUTYH,MYCN,NPM1,PGR,PTEN,PTPN11,PTPRS,RAD50,RHOA,SH2D1A,SMAD2,SMAD3,SOX9	0.386298593	1.144429774
path_1011	BTK,CDKN1B,CHEK2,CRLF2,EIF4A2,FGFR1,FLT3,GRIN2A,INSR,MED12,MET,NOTCH4,PAK7,PBRM1,PDGFRB,PRDM1,PTCH1,SDHC,SH2B3,SMARCD1	0.135858421	0.785032301
path_1012	ABL1,DAXX,DDR2,ERCC2,ERCC5,ETV6,EZH2,GATA2,H3F3C,HIST1H3I,IGF1R,MEF2B,MTOR,NEGR1,NKX2-1,PAK1,PIK3CB,RBM10,STAG2,TCF3	0.712681207	1.066823924
path_1013	ASXL1,BAP1,BCL2L11,CD274,CDKN2A,DNMT3B,ELF3,EPHA7,ERCC2,HGF,HIST1H3H,HOXB13,INSRR,IRF4,MUTYH,RFWD2,SMARCB1,TAP1,TCF3,TSC2	0.353316343	1.167636115

path_1014	CCND3,CDKN1B,FANCC,FGF19,FLT1,FOXP1,FYN,GRIN2A,HGF,HNF1A,MAP3K13,MUTYH,NBN,NKX2-1,PAX5,PTEN,RET,RPS6KA4,SDHB,SH2B3	0.753428366	0.950551392
path_1015	CHEK2,CSF3R,CTNNB1,CXCR4,ELF3,ERBB4,ETV6,FLT3,HNF1A,IKZF1,IL10,INSR,KMT2D,MAP2K4,MGA,MYCN,NOTCH4,PLK2,RUNX1,SETD2	0.545674053	1.099999067
path_1016	ACVR1,BCOR,CTCF,CTLA4,FGFR4,HGF,IFNGR1,IRS1,KDM5A,KDM5C,MAP2K4,MLL3,NTRK1,PDCD1,RAD54L,RAF1,RYBP,SMAD3,SOX17,SUFU	0.404687127	1.15617947
path_1017	APC,ATM,AXL,CHEK1,EGFR,EPHA7,FGF4,H3F3C,HGF,IL7R,KIT,LATS1,MST1R,MYCL1,NEGR1,PAK1,PIK3CB,PIK3R2,ROS1,STAT5B	0.405565294	0.880975639
path_1018	AKT2,ASXL2,B2M,BRAF,CD79B,DOT1L,ERCC4,FGF19,GATA2,KDM5C,KLF4,KMT2A,MEN1,MLL2,MYCL1,PALB2,PPP2R1A,RHOA,SPEN,TET1	0.123347705	1.333404035
path_1019	APC,BCL2,ESR1,FGFR2,FOXL2,HIST1H3J,HNF1A,INHA,INPP4A,MSH6,NOTCH3,PBRM1,PIK3C2G,PLK2,PPP6C,RBM10,SDHC,SH2B3,SOX17,STAT3	0.257923277	1.217465842
path_1020	BLM,BRCA2,CALR,CCND1,CCNE1,CDK4,CTCF,E2F3,EIF4E,EP300,EZH2,FAM46C,HGF,HIST1H3I,INSR,KDM5A,MST1,PARK2,PDGFRA,STK40	0.271019543	1.172419244
path_1021	ABL1,AXL,CASP8,CD79B,CDC42,CDKN1B,CHEK2,CYSLTR2,FGF3,HIST1H3B,IGF2,JAK3,KDR,KMT2A,MED12,MST1,NF1,RAD54L,RARA,RASA1	0.167595254	1.215510016
path_1022	APC,BARD1,BCL2L11,BMPR1A,BRD4,CCNE1,CHEK1,CUL3,DICER1,DNMT3A,EPHB1,ERCC4,FH,FUBP1,HIST1H3G,IL10,NTRK1,RASA1,RNF43,TBX3	0.819561073	1.032871063
path_1023	B2M,BRIP1,CDC42,CDK12,CHEK2,CYSLTR2,DNAJB1,EIF1AX,HNF1A,INPP4A,MLL3,MSH2,NBN,PARK2,PIK3CG,PMAIP1,PMS1,POLD1,PTEN,TAP1	0.260608222	1.216146719
path_1024	ACVR1,CHEK2,CYSLTR2,ETV1,FLT3,HNF1A,ICOSLG,IGF2,KMT2D,MEF2B,MLL2,MSH3,NCOA3,NEGR1,PDCD1,PMS1,RUNX1,SOS1,SOX2,TMPRSS2	0.402282795	1.127409792
path_1025	AMER1,ARID5B,ASXL1,BLM,FANCC,FH,HIST1H3D,KNSTRN,MEN1,MET,MSH6,NEGR1,PARP1,PBRM1,PGR,PIK3C3,PIK3R1,PIK3R2,RAD50,TRAF7	0.142450386	1.302513164
path_1026	ASXL2,BCOR,CARM1,CHEK2,EIF4E,ERCC2,FGF3,FOXP1,ICOSLG,IL7R,JUN,MAP2K4,NKX2-1,PIK3CA,PIK3R2,PIK3R3,PMS2,PTPRS,RPTOR,TBX3	0.099999976	1.359190383
path_1027	ARID1B,AXIN1,CDKN2C,CENPA,ERBB2,FBXW7,FGF19,FGF4,FGFR2,GNAS,HNF1A,MDC1,MET,NCOA3,PBRM1,PPP6C,SMAD3,SMARCB1,TRAF2,YAP1	0.371031614	1.192146409

path_1028	DAXX,DNMT3A,EIF4E,ELF3,EPHB1,FAT1,FLT4,GPS2,HIST1H3E,IGF1R,INSRR,MSH6,MYC,NOTCH1,PARK2,PDCD1,PIK3CB,PLCG2,SMAD4,TNFAIP3	0.833633993	1.036576597
path_1029	AMER1,ARID5B,AXL,BTK,CARD11,DROSHA,EP300,EZH2,FGF19,HIST1H3J,JAK1,KDM6A,NPM1,PGR,PIK3R1,PIM1,PPM1D,RET,SMARCD1,STAT3	0.056015963	1.313946548
path_1030	APC,AXL,CARD11,CDH1,DNMT1,FGF4,FGFR3,FLT1,FOXL2,HIST1H3C,INHA,JUN,KDR,KRAS,MALT1,NTRK2,PMAIP1,PTPN11,PTPRS,RPS6KB2	0.87417179	0.976644136
path_1031	ATR,BCL2L11,BRD4,CALR,CARD11,EPHA3,EPHA5,GNAQ,IDH1,INHBA,INSRR,MALT1,MAP2K1,MLL2,MRE11A,NFE2L2,NFKBIA,PPP6C,RHEB,TP53BP1	0.34550179	1.156716032
path_1032	ATR,BRAF,CALR,CASP8,CDK4,EGFR,ERCC2,HOXB13,IGF2,IKBKE,KLF4,NFE2L2,NFKBIA,NKX2-1,NOTCH2,POLE,PPP2R1A,SMARCA4,STAT3,TNFAIP3	0.052178808	1.375094085
path_1033	APC,B2M,CDKN1B,CTNNB1,FOXL2,HGF,HIST1H1C,IGF2,MAX,MED12,MSH6,MYC,MYOD1,PDCD1,PDGFRB,PIK3CA,PIK3R1,PTPRD,PTPRS,RICTOR	0.100634127	1.2836078
path_1034	AKT2,ATM,BARD1,BCL2,CCND3,EPHA5,ERG,FLT3,GRIN2A,IDH1,IGF1R,INSR,IRS2,JUN,KNSTRN,PAK7,PDGFRA,RHEB,RTEL1,SOS1	0.496678164	0.899804668
path_1035	ARAF,BLM,CDK6,CSF1R,CTNNB1,CUL3,DICER1,EIF4E,HIST1H3A,HLA-A,HNF1A,IKZF1,KDR,KIT,MAP2K1,NCOA3,RAD50,RB1,RFWD2,TCF3	0.764285616	0.951199343
path_1036	ARID2,AXL,BRIP1,CDK12,EIF1AX,GNA11,IRF4,LATS1,MDM2,MDM4,NEGR1,NF1,NFE2L2,NFKBIA,NOTCH3,NPM1,RAD54L,RTEL1,SOX2,SYK	0.116304769	1.331869454
path_1037	ARID1A,ARID1B,CTCF,DIS3,ERRFI1,FOXO1,IKBKE,IL10,INSR,JAK1,LATS2,PALB2,PMS1,RAD21,RICTOR,SDHC,SLX4,SMARCD1,SOCS1,SOX17	0.322392822	1.150522588
path_1038	BCL2,DDR2,FGF3,GNAS,GSK3B,IL10,INSR,INSRR,IRF4,IRS2,MAP2K2,NF1,NF2,PIK3C2G,RAD50,RPS6KA4,SDHAF2,SETD2,SMARCA4,SOX2	0.145666146	1.272093676
path_1039	ALK,ATRX,CALR,CCND3,FBXW7,FGF19,H3F3C,MALT1,MAP3K1,MST1,PDGFRA,PHOX2B,PLK2,PRDM1,PTCH1,RAD51,SDHAF2,SRC,STAG2,STAT5B	0.629217935	1.070791393
path_1040	ABL1,AKT2,AURKA,CBL,CDK6,CENPA,CTCF,DAXX,EPHA5,ERBB4,ERCC5,HIST1H3C,JUN,LATS2,NOTCH4,NUP93,PIK3CA,RAD51B,TET2,TGFBR2	0.327633381	1.167636833
path_1041	AMER1,APC,ATM,CALR,CRLF2,CUL3,E2F3,EIF4E,ESR1,FLT3,GLI1,GPS2,HIST1H3B,IDH1,LATS1,NBN,NFE2L2,NKX2-1,PTPRD,TMPRSS2	0.898444721	0.98114141

path_1042	ACVR1,BAP1,BRCA1,CDH1,CDK12,CEBPA,CSF3R,CTLA4,DNMT3B,E2F3,FANCC,KIT,MCL1,PAX5,PIK3C3,PIK3CG,RAD51,SDHA,SLX4,SMAD3	0.113715288	1.338597044
path_1043	ABL1,AKT2,BLM,CD274,ERBB3,ETV6,H3F3C,IL7R,IRF4,MDM2,MET,NEGR1,POLE,PTPN11,RAD21,RPS6KB2,SHQ1,SOS1,SOX9,TNFAIP3	0.806083806	1.035783706
path_1044	BAP1,CCND3,CDK4,CUL3,FBXW7,IDH1,KEAP1,LATS2,MCL1,MSH3,NFE2L2,NOTCH3,PGR,PIK3C2G,RAD51D,RECQL4,SH2D1A,SMAD3,SOX2,SYK	0.155447802	1.239820755
path_1045	ANKRD11,CSF3R,DOT1L,E2F3,EGFR,FANCC,HNF1A,KLF4,NKX3-1,PAK7,PALB2,PBRM1,PHOX2B,PTEN,RAC2,RAD51C,REL,RHEB,RHOA,SF3B1	0.889839868	1.024678318
path_1046	ATRX,AXIN1,BCL10,CDH1,CTNNB1,ERBB2,KRAS,LATS1,MAP2K1,MAP2K2,MLL,MSH3,MST1R,NTRK3,PMS2,RHOA,RPS6KA4,RYBP,STK11,TET2	0.301406022	1.182553567
path_1047	ATRX,CDC73,CDK12,DOT1L,EPHA7,ERBB4,EZH2,FBXW7,HLA-A,MALT1,MAP2K2,MSH6,MST1R,NKX3-1,PIK3C2G,PMAIP1,POLD1,PPM1D,RPS6KB2,TAP2	0.114140589	1.306082858
path_1048	BBC3,CDK12,CDKN1A,EPHA7,EZH2,FAM175A,HIST1H3C,IKBKE,MED12,MST1,NEGR1,PHOX2B,PLCG2,PNRC1,PPP2R1A,RAD51C,SOX2,TAP1,TBX3,TERT	0.056941707	1.326787581
path_1049	ALK,ASXL2,BRD4,CARM1,CDC42,CDKN1A,CEBPA,ELF3,EPHA7,FGF4,FGFR4,HIST1H3B,MAP2K4,MDC1,MYOD1,NBN,PIK3R2,PRDM1,TCF7L2,TP63	0.732532018	0.952558951
path_1050	AKT1,AURKA,BLM,BRCA2,CCND3,CD79B,CDKN1A,CHEK1,HIST1H3G,IDH1,INPP4A,JAK3,LATS2,MST1R,NCOA3,PIK3C3,RAD51B,SH2D1A,TEK,TP53	0.055072343	1.321621734
path_1051	AMER1,AXL,DICER1,EIF1AX,ERBB3,FGFR3,FOXL2,FOXO1,IKBKE,JAK3,NRAS,NTRK3,PBRM1,PGR,PREX2,PTEN,RHEB,RPS6KA4,SHQ1,TET2	0.308879886	1.194461067
path_1052	AXIN2,BCL2L11,BCL6,CEBPA,ETV6,FUBP1,HIST1H3E,INPP4B,JAK2,LATS2,MAP2K4,MDC1,MLL,NPM1,NRAS,PAK7,PIK3CA,PREX2,RAD51D,RB1	0.541588788	1.1066034
path_1053	AXL,BCL2,CCND1,CDH1,DNAJB1,DNMT1,EIF1AX,EIF4E,EPHA7,FGFR4,HIST1H3C,MYCL1,PAK7,PARP1,RAD51,RFWD2,ROS1,SOCS1,SOS1,STK11	0.488380185	1.126926616
path_1054	ANKRD11,BRAF,BTK,CCND3,FAM46C,HIST1H3E,JAK1,KDM6A,MEN1,MGA,MITF,NKX2-1,NOTCH4,NRAS,PDGFRB,PIK3R1,PIM1,PTPN11,RPS6KA4,TCF7L2	0.944174134	0.990173517
path_1055	CCND3,CDKN2A,CENPA,FANCC,FOXA1,HIST1H3J,KDR,MALT1,MYC,NF1,PNRC1,RPS6KB2,RTEL1,SOX2,SRSF2,SYK,TEK,TET1,TNFAIP3,TRAF7	0.08310802	1.42572531

path_1056	ATM,BARD1,BRCA2,BRD4,CENPA,EPHB1,ERCC5,FGFR1,FGFR2,MET,MLL3,NOTCH4,PDCD1,PDGFRB,PIK3C3,POLD1,SETD2,SOX9,TERT,TET1	0.535647653	1.097655652
path_1057	ATM,ATR,CDKN2C,ERCC2,ESR1,FOXA1,FOXL2,ICOSLG,MEN1,NTRK1,PDCD1,PGR,PLCG2,PMS1,PPP2R1A,RAD21,RFWD2,SOX17,TCF3,TOP1	0.447316874	0.880162491
path_1058	BRD4,CDH1,CRLF2,E2F3,ELF3,ERG,HIST1H3A,IL7R,JAK3,MAP3K13,MPL,NOTCH3,PARK2,PBRM1,PTPRS,RARA,RPTOR,SMARCA4,TCF3,TP53	0.167154266	1.219630961
path_1059	CASP8,CCND1,CRLF2,CTCF,ERBB3,FOXP1,HLA-A,KDM6A,MAP2K4,MYC,NF1,NF2,NKX2-1,PDCD1,PIM1,PPM1D,PPP2R1A,RTEL1,SDHA,SDHC	0.221861752	1.188052692
path_1060	CIC,FGFR4,HIST1H3B,HIST1H3D,HIST3H3,IKBKE,KLF4,MAP3K1,NFE2L2,NUF2,PARK2,PBRM1,PIK3R2,PMAIP1,RET,RHEB,RHOA,RPTOR,SH2D1A,TET1	0.060241605	1.304780397
path_1061	BCL6,CDK4,CDKN2C,CTCF,CYSLTR2,EIF1AX,FANCA,FLT4,HIST1H3H,KMT2D,LATS1,MET,MYC,NKX2-1,NOTCH1,PAX5,PGR,PLK2,SMAD2,STAT3	0.247275204	1.223153269
path_1062	ALK,ALOX12B,BBC3,CBL,CSF3R,DNAJB1,E2F3,EP300,ERBB4,GLI1,GSK3B,IL10,IRS1,NFKBIA,NOTCH4,PDGFRA,PHOX2B,PTPN11,TCF3,TGFBR2	0.204478846	1.233844098
path_1063	BCL2L11,CDC42,CDH1,CDK8,CDKN2A,CIC,ERRFI1,GRIN2A,HNF1A,INHA,KDM5C,KIT,MAP2K4,MYCN,PIK3CG,PIK3R1,SDHC,SETD2,TCF3,TP63	0.172498986	1.242745893
path_1064	ATRX,BBC3,BRAF,CD274,CTNNB1,DNAJB1,EIF4E,FGFR1,FGFR3,HIST1H3D,IKBKE,IRS2,MAP3K13,MET,NOTCH2,PREX2,RARA,SETD2,SH2B3,TP63	0.302892917	1.192779153
path_1065	ALOX12B,CDH1,CDK4,CDK8,FGF3,FH,FOXA1,GATA2,IDH1,JAK2,JUN,MDC1,MLL,MTOR,NBN,NTRK1,RAD54L,RPTOR,SDHA,SMAD4	0.767307955	0.958964562
path_1066	ATM,AXIN2,BRAF,CRLF2,CYSLTR2,DDR2,DICER1,H3F3C,HIST1H3D,HNF1A,ICOSLG,KDM5A,MDM2,MYCL1,NTRK1,PDCD1,PIK3CG,PLK2,PTPN11,RUNX1	0.502313105	1.117731868
path_1067	ABL1,AKT1,CCND3,CD274,CIC,EPHA3,ERRFI1,FGFR3,FLT1,LATS1,MDM4,MEF2B,MYOD1,NFE2L2,NOTCH4,PIK3C3,RAD54L,RASA1,RET,RFWD2	0.966900976	1.00686513
path_1068	ARID2,BARD1,CREBBP,CUL3,DIS3,ERBB3,FGFR3,INSR,IRF4,LATS2,MET,MITF,NFE2L2,NKX3-1,NRAS,POLE,ROS1,RPS6KB2,SMARCA4,SOX9	0.086605799	1.289381715
path_1069	ARAF,ATR,BRIP1,DDR2,DNMT1,FANCC,GATA3,HIST1H3H,MALT1,MAP2K4,MAP3K1,MSH2,NOTCH2,NUP93,PTEN,RAD51D,SDHAF2,SDHC,STK40,SUFU	0.171868773	1.214901411

path_1070	AKT1,AKT3,BCL2L1,BLM,CD79B,CRLF2,EPHA7,FBXW7,FGFR4,FLT1,FOXO1,HOXB13,MAP2K2,NCOR1,NF1,NOTCH1,PDGFRA,PPM1D,ROS1,SDHAF2	0.341837649	1.166813602
path_1071	ANKRD11,AURKA,CHEK1,CSF3R,CYSLTR2,EGFR,FGFR2,FH,GATA3,HIST1H1C,HIST1H3J,MUTYH,NBN,NUF2,NUP93,RTEL1,SDHC,SOX17,SRSF2,TOP1	0.511581577	0.911335453
path_1072	AKT1,AR,BCL10,CCND1,CCND2,CDK6,CIC,DDR2,FOXL2,H3F3C,INPP4B,KDR,KIT,MAP3K13,MEN1,MST1R,NEGR1,PARK2,PIK3R3,RBM10	0.263759543	1.210649276
path_1073	ARID1A,BCL2L11,CASP8,FH,HIST1H3H,IKBKE,JAK2,KEAP1,KLF4,MGA,MYCN,NCOA3,NF2,PIK3CA,PTPN11,RAD51C,RAF1,RPS6KB2,SMO,TAP1	0.267393079	1.183545436
path_1074	ACVR1,AKT3,ALK,CARD11,CCND3,CD276,CDC73,CYSLTR2,FGFR1,GLI1,GRIN2A,KLF4,MAP3K13,MDM2,MST1,NCOR1,NKX2-1,PIK3CA,PLCG2,RAD54L	0.167276717	0.82453523
path_1075	ARID5B,AURKA,BCL6,DNMT1,GATA1,GATA2,GATA3,HIST1H3D,KLF4,KMT2A,MET,PIK3R3,PMS2,RAF1,RICTOR,SMARCB1,SMO,SRSF2,SYK,TGFBR1	0.561169453	1.142260578
path_1076	ANKRD11,AXIN2,BRIP1,CDK12,CEBPA,CENPA,ERCC2,FAM46C,FGFR3,FYN,GPS2,HIST1H3J,HOXB13,INHA,IRS1,MAP2K4,MUTYH,PAK1,REL,STAT3	0.682368395	1.059503585
path_1077	ARID2,AXL,CDK8,CTCF,ERBB4,ERG,ERRFI1,ETV1,GNAS,HIST1H3I,KRAS,MALT1,MRE11A,MYOD1,NTRK2,RAD21,SMARCD1,STAT5B,TAP1,TP63	0.769020236	1.046870221
path_1078	BRCA2,CBL,CDK12,DIS3,E2F3,FBXW7,FGFR1,FGFR2,H3F3C,IDH1,IL7R,JAK2,MAX,PTPRS,RICTOR,RNF43,RTEL1,SDHAF2,SMAD4,SRSF2	0.751214633	1.045814348
path_1079	ATR,AURKA,AXL,BCL2,BCL2L1,CDC42,CEBPA,ETV6,FH,HGF,HIST1H3D,ICOSLG,MTOR,NF1,PIK3CG,RAD51C,SDHA,SDHB,SOS1,TAP1	0.15255367	1.274106158
path_1080	AXIN2,BRCA2,CCNE1,CREBBP,EPHB1,FGFR4,GATA1,HIST1H3J,HOXB13,IL10,KRAS,MDC1,MDM4,MSH2,NTRK1,PDGFRB,PIK3C3,RAD51C,TCF7L2,TERT	0.842070654	1.029883567
path_1081	AR,ARID5B,BRCA2,CALR,CDK8,EIF4A2,ERCC2,FLT1,HIST1H3C,HIST1H3E,KNSTRN,KRAS,MPL,MYOD1,PTPRS,RUNX1,SLX4,SRC,TCF7L2,TEK	0.555593044	1.096603058
path_1082	CD274,CD276,CD79B,DROSHA,EIF1AX,EIF4A2,FGFR4,HIST1H3H,MAP2K1,MAP2K2,MTOR,NCOR1,NTRK3,PMS1,PTPN11,PTPRD,PTPRT,RET,SMARCD1,STAT5B	0.183161055	1.242794109
path_1083	ARID2,AXIN2,CCND3,CDKN1A,CTNNB1,ERCC4,ETV1,FGF19,FOXO1,HIST3H3,IGF1R,IRF4,KDM5A,LATS1,MPL,PAK1,PMAIP1,RAD51C,RTEL1,SDHC	0.715589308	1.053148879

path_1084	AXIN2,CALR,CHEK2,GRIN2A,HLA-A,HNF1A,KRAS,MEF2B,MET,MSH6,NF1,NOTCH2,NTRK2,NUP93,PDGFRB,PIK3R2,PPP6C,PTCH1,PTPN11,RECQL4	0.842587919	1.028744084
path_1085	ASXL1,BRCA1,CASP8,DICER1,DROSHA,EIF4E,EP300,FYN,JAK3,MAP2K2,NKX2-1,NPM1,PGR,PIK3CA,PRDM1,RAD51,RECQL4,SDHB,SF3B1,STAG2	0.810304884	1.034685018
path_1086	ASXL2,CDH1,CDKN2C,CENPA,DNAJB1,EIF4E,EPHA5,EZH2,FANCA,FGFR1,INSR,JAK2,MSH2,MST1,RAD51,SH2B3,SOS1,TBX3,TET2,WT1	0.343879669	1.143476749
path_1087	AXIN1,CDKN1B,CTNNB1,EIF4A2,ETV6,FUBP1,GSK3B,HIST1H3A,HIST1H3C,IL10,IL7R,INHA,MAP3K1,MSH3,MUTYH,NTRK2,PHOX2B,RAD51D,RAF1,TET2	0.484519963	1.103844629
path_1088	ARID1A,CTLA4,E2F3,EIF4A2,ERCC5,FANCA,FGFR4,MDC1,MSH2,POLE,PTPRT,RAD51D,RAD54L,RB1,RBM10,RHOA,SH2B3,SOS1,TBX3,TP53BP1	0.179816723	1.222295828
path_1089	ARAF,CALR,CREBBP,CRLF2,DOT1L,EPHA7,ERCC4,GSK3B,HIST3H3,KLF4,MLL2,MYCL1,MYCN,PAK7,RAC2,RPS6KB2,RTEL1,SDHB,SOX2,SRSF2	0.118683263	1.32910385
path_1090	ATR,BAP1,CCND2,CD79B,CDK12,EPHB1,FAM46C,FAT1,IFNGR1,IGF2,JAK2,JUN,KEAP1,MAP3K13,PARP1,PIK3CD,PPM1D,RAD54L,SOCS1,SRSF2	0.716859233	1.06114958
path_1091	BBC3,DIS3,DROSHA,EGFR,ETV1,FAM175A,FGF3,GRIN2A,HIST1H3A,IGF2,IKZF1,KEAP1,KRAS,MDM2,MUTYH,MYCN,PAX5,PIM1,PNRC1,SDHC	0.349382926	1.166611844
path_1092	ARID1A,B2M,BCL2L11,CD79B,CIC,EIF1AX,FGFR1,FGFR4,HNF1A,IGF1R,MAP3K13,MET,NCOA3,NFKBIA,NTRK3,PDCD1,PMS2,RAD51C,RHEB,RTEL1	0.298183462	1.209176195
path_1093	AKT3,ARID5B,AURKA,BLM,EP300,FLT3,FYN,GATA2,MRE11A,NOTCH4,PARK2,PIK3CA,PIK3R2,PIK3R3,RASA1,RNF43,SDHC,SH2D1A,SMAD4,TCF3	0.252374202	1.208961798
path_1094	ASXL2,ATRX,AXIN1,BCL6,CDH1,CSF1R,CYSLTR2,HIST1H3E,JUN,KLF4,MEN1,MPL,MTOR,NTRK1,PMAIP1,RET,RNF43,RPTOR,SMAD3,STK40	0.073834209	1.291598425
path_1095	BMPR1A,CSF3R,EGFR,EIF4E,EPHB1,ERCC2,FGFR4,FOXL2,HIST1H3J,JAK3,MED12,MEN1,MSH3,MYC,NOTCH3,NUP93,PIK3R2,PPP2R1A,RHEB,SMARCD1	0.338456722	1.182443589
path_1096	ALOX12B,ATM,BLM,EED,EPHA3,FH,GATA3,HIST1H3H,JAK1,MAP3K1,MDM2,NRAS,PAK7,PPM1D,RAD51C,RASA1,SDHAF2,SDHC,STK40,TAP1	0.116418468	1.319513414
path_1097	BTK,CCND2,CHEK1,DROSHA,FGFR1,GLI1,MAP2K1,MDC1,MST1R,MYCN,NFKBIA,NOTCH3,NSD1,PAK1,PIK3C3,PRDM1,RNF43,RTEL1,SMAD4,STAT3	0.820746867	0.962579413

path_1098	ATM,AXIN2,CARM1,CDKN2C,CSF3R,DICER1,DNMT3A,FBXW7,FGF3,FUBP1,HIST1H1C,IKBKE,KLF4,KRAS,MAP3K1,ROS1,RTEL1,SDHA,SDHC,SH2D1A	0.424451657	1.124003075
path_1099	AKT3,ARID1B,ARID2,BCL2L1,CDK12,EP300,ERCC4,FAM46C,FANCA,ICOSLG,IDH1,IRF4,IRS2,KDM5A,MDC1,NF2,NOTCH1,NTRK2,RAD51C,RFWD2	0.935097917	1.013244124
path_1100	ALOX12B,AR,ARID2,CCND2,HIST3H3,IRF4,IRS1,KLF4,LATS1,MTOR,MYOD1,NCOA3,PARK2,PREX2,PTEN,RAF1,RB1,RTEL1,SH2B3,SMARCB1	0.194281029	1.255875294
path_1101	ASXL1,CCND2,CDKN1A,DIS3,EPHA5,FANCA,FOXO1,FOXP1,GSK3B,HIST1H3C,IKBKE,INHA,KNSTRN,MEN1,MITF,MYCL1,PLK2,PTCH1,SMAD2,SMAD3	0.89790211	0.982016231
path_1102	ARAF,ARID5B,CDK8,CSF1R,CSF3R,EGFR,ERCC4,FLT1,FOXA1,FOXL2,FOXO1,GRIN2A,IKBKE,IL7R,MST1R,NBN,NF1,PDGFRA,PIK3CA,RBM10	0.83577627	0.970632255
path_1103	ANKRD11,APC,ARID2,AXIN1,BBC3,BCOR,BRAF,CD79B,CHEK2,DNMT1,EPHA3,EZH2,FOXO1,GPS2,HIST1H3J,MLL3,NOTCH3,RUNX1,SDHB,SLX4	0.140093744	1.259512305
path_1104	ACVR1,ARID1B,BBC3,CCND1,CDH1,CSF1R,DDR2,ELF3,FLT4,FYN,IRS1,KEAP1,MLL3,PTPRD,RAD51C,RASA1,SETD2,SMAD4,TAP2,TMPRSS2	0.206075502	1.201131499
path_1105	AXIN2,BCOR,BRCA1,CARD11,CBL,EIF1AX,FANCC,FGF4,FGFR4,HIST1H3J,KRAS,MEF2B,MET,MSH2,NOTCH1,PHOX2B,PNRC1,POLE,SOX2,STAT5B	0.484353443	1.106385994
path_1106	AXIN2,BRAF,CDC42,CDKN2C,CXCR4,EIF4A2,EP300,GRIN2A,HOXB13,KDM5A,KEAP1,KNSTRN,NOTCH2,NSD1,PDCD1,PIK3C2G,RUNX1,SDHB,SOX9,TCF7L2	0.509215458	1.101727882
path_1107	AXIN2,B2M,CCND1,CDC73,ELF3,EP300,FANCC,GNAS,MAP2K1,MTOR,NKX3-1,PALB2,PIK3CD,PIK3R1,PMAIP1,PRDM1,PTPRT,SDHA,SOX9,SRSF2	0.782332192	0.961683317
path_1108	AKT3,CCND2,CD79B,CYSLTR2,DOT1L,FOXO1,FOXP1,KRAS,MRE11A,NOTCH4,NTRK1,PIK3CA,PRDM1,RBM10,SF3B1,SMARCB1,SPOP,TAP1,TAP2,TGFBR1	0.274059366	1.167790906
path_1109	ARID1A,CDKN2A,EIF1AX,FOXP1,GRIN2A,H3F3C,HGF,INSRR,KDM5A,MAP2K2,MDM2,MTOR,NF2,NTRK1,PDGFRB,PLCG2,PRDM1,PTPRD,RBM10,TNFAIP3	0.102359088	1.263779008
path_1110	AMER1,ASXL1,ATM,BRAF,BRD4,EGFR,FLT4,GATA2,H3F3C,MAP2K1,MAX,MGA,MSH3,PARK2,PAX5,PIK3CA,PIK3R3,RET,SOX9,STAG2	0.832629629	0.970562007
path_1111	ARAF,AXIN1,BCL6,CDH1,CTCF,DNAJB1,EGFR,ERRFI1,FGFR2,FLT3,GLI1,H3F3C,HGF,KRAS,NKX3-1,NOTCH4,PMAIP1,PTCH1,RARA,RYBP	0.779489854	1.041168017

path_1112	BCL2L11,CARD11,CENPA,CIC,E2F3,EPHB1,ESR1,FANCC,FGFR4,HIST1H3E,HOXB13,IKZF1,IRS1,MET,MST1,PIK3R3,PLK2,ROS1,SMAD4,SOX17	0.66293423	1.06354712
path_1113	ALK,CCNE1,CDK6,CENPA,CSF1R,DNMT1,DROSHA,FOXO1,FUBP1,HIST1H3J,KDM5A,KNSTRN,MALT1,MAX,MLL2,PLK2,RASA1,RHEB,RPS6KB2,SMAD3	0.560885572	1.085581273
path_1114	ASXL2,BCL2L1,CBL,CD274,CD79B,CDK4,CRLF2,DAXX,EPHA3,EPHA7,ETV1,GPS2,KDM6A,NBN,NEGR1,NTRK2,PIK3CA,SDHB,SOX9,SYK	0.959442895	1.007209863
path_1115	AURKA,CDKN1A,CSF1R,CXCR4,EGFR,JAK2,KDM5A,MAP2K1,MCL1,NF2,NOTCH3,NOTCH4,PAK1,PAK7,RAF1,REL,RYBP,SMAD2,SMARCD1,SMO	0.245993797	1.217323713
path_1116	ALOX12B,APC,ATM,CDKN1A,CHEK2,FOXL2,JAK2,MSH3,MYOD1,NCOR1,PALB2,PIK3R2,PLK2,PRDM1,RPS6KA4,SH2D1A,SLX4,SOS1,SOX2,STK40	0.271859614	1.252902002
path_1117	AKT1,ARAF,ATM,BRAF,CARM1,HIST1H3D,IKZF1,IRF4,JAK3,KDR,LATS1,MGA,MST1R,NOTCH2,NOTCH4,PIM1,PPP2R1A,PRDM1,RAD51B,RECQL4	0.065915245	1.339114077
path_1118	AXIN2,BCL2L1,BCL6,BRCA2,CCND2,CENPA,CXCR4,ELF3,FOXP1,GNAQ,KEAP1,NKX2-1,PAK7,PIM1,PLK2,RBM10,REL,SMARCD1,STAT3,TOP1	0.365150939	0.874473509
path_1119	ATR,CHEK2,EIF1AX,ERG,ESR1,FAM175A,FGFR2,HIST1H3A,HNF1A,KDM6A,KEAP1,KIT,MDC1,MRE11A,MYOD1,PBRM1,RAD51C,RAD51D,SMARCD1,SOX9	0.570514788	0.916931567
path_1120	ATR,BRCA2,CDH1,CDKN2C,CIC,DNMT3A,E2F3,ERCC5,ESR1,FGF3,FLT1,H3F3C,HOXB13,MET,MLL2,MSH3,NKX2-1,PDGFRB,PTPRD,SRC	0.051494426	1.342822071
path_1121	AURKA,BMPR1A,BRCA1,CDC73,CDKN2A,CDKN2C,CREBBP,ETV6,EZH2,FAM175A,FGFR1,HIST1H3J,MALT1,MED12,MEF2B,PTEN,RICTOR,RNF43,SDHB,TSC2	0.249416627	1.222585728
path_1122	ASXL2,BCL10,BRIP1,CCND1,EED,ESR1,FGFR4,FLT1,GATA1,GNA11,HIST1H3D,KDR,KIT,PAK7,PIK3CA,PTPRD,RUNX1,SDHB,TBX3,TRAF2	0.054123243	1.332126666
path_1123	ABL1,AMER1,APC,BRCA2,CCNE1,ERBB4,EZH2,FGF19,KMT2D,MAP3K13,MUTYH,NKX3-1,NOTCH2,PHOX2B,PIK3R1,RB1,RHEB,SMARCB1,SOS1,TNFAIP3	0.359066852	1.169762653
path_1124	BCOR,BTK,CSF3R,CXCR4,DIS3,EPHA3,FGF4,FGFR2,FLT3,MEN1,MYC,PIK3CB,POLD1,PTCH1,RAD54L,RB1,SDHAF2,SMAD4,TMPRSS2,U2AF1	0.707603969	1.061697647
path_1125	ARID1A,AURKA,AXIN1,BCL2L11,CASP8,CDK8,ERBB4,FLT1,GATA1,HIST1H3E,LATS2,MAP3K13,MED12,NKX2-1,PHOX2B,PIK3R1,RAD54L,RB1,TERT,TP63	0.096701591	1.301834738

path_1126	AKT1,ATM,BBC3,BRCA1,E2F3,ETV1,FGF3,FYN,GATA2,GNAS,HOXB13,INHBA,LATS1,MST1R,PAK1,PIK3CA,RAC2,REL,STK40,TCF3	0.066497269	1.30066155
path_1127	ATM,BCL6,BCOR,CDK12,CDK6,CXCR4,DNMT1,EPHA5,EPHA7,FGF3,GRIN2A,MAP2K1,MITF,PAK1,PIK3C2G,PIK3CG,RICTOR,SRSF2,TET2,TSC1	0.405301166	1.12921762
path_1128	AKT1,CYSLTR2,DNMT3B,E2F3,EIF4E,EPHA7,ERG,FLT1,HIST1H3I,HLA-A,JAK2,KDR,MCL1,MDM2,MEN1,NCOR1,PDGFRA,PMS1,SRSF2,TP53	0.126142381	1.243556029
path_1129	BCL2,CBL,CTNNB1,FAT1,HLA-A,ICOSLG,IKZF1,INSRR,KDM5C,KRAS,MALT1,MAX,MDM4,NKX3-1,NRAS,PIK3C2G,RBM10,RPS6KB2,SDHB,SOCS1	0.696261501	1.071490092
path_1130	ALOX12B,CBL,CDH1,CDKN2C,CTCF,DNAJB1,ERBB3,GNA11,HGF,HIST1H3H,HIST3H3,IL7R,IRF4,PPM1D,PTCH1,SOX17,TCF3,TCF7L2,TET1,TOP1	0.185545873	1.210618783
path_1131	BRCA1,CDC73,CDKN2A,DAXX,GPS2,MAP2K1,MAP3K1,MAX,MCL1,PIK3R1,PIK3R3,PLCG2,PREX2,RFWD2,RHOA,SH2B3,TRAF2,TSC1,WT1,XPO1	0.210407639	1.193509565
path_1132	BAP1,CCND1,CTCF,ERBB2,FGFR4,GLI1,HIST1H3A,HIST1H3C,IGF2,KLF4,MDM4,NOTCH2,PIK3CD,PIK3R3,RECQL4,SHQ1,SMAD4,SMARCB1,TCF7L2,TNFAIP3	0.379838743	1.133225922
path_1133	ABL1,BAP1,CDC73,CDK6,CTLA4,ERBB3,FANCC,KMT2A,MET,MYCN,NCOA3,NEGR1,NF1,NF2,PBRM1,PMS2,RAD54L,RPS6KB2,SMARCA4,SMO	0.067582575	1.337008514
path_1134	ABL1,AR,ARID1A,CDK4,CXCR4,DICER1,EPHA7,ERBB4,FUBP1,HIST3H3,IKZF1,LATS2,MALT1,MED12,MSH3,MYC,NKX3-1,RHOA,STAG2,YAP1	0.154509042	1.257703365
path_1135	AKT3,AURKA,CHEK2,CTLA4,DDR2,DOT1L,HIST1H3I,HIST1H3J,INSR,IRS2,KDR,KRAS,MAP2K1,MYC,MYCN,NF1,NSD1,NTRK1,NUP93,PHOX2B	0.368723039	1.135537365
path_1136	ACVR1,ARID1B,ATM,BCL2,BRD4,CDK4,DOT1L,FAT1,GNAQ,H3F3C,IL10,MRE11A,NTRK3,PBRM1,PTPRS,SHQ1,SOX2,STAG2,TBX3,TERT	0.222096558	1.222453026
path_1137	AR,ARID1B,CASP8,CHEK1,DOT1L,FLT4,GATA2,GLI1,H3F3C,IGF1R,IKZF1,INHBA,KMT2A,MSH3,NFE2L2,NTRK2,PARK2,RNF43,SMAD4,STK11	0.218797843	1.195205811
path_1138	AKT3,ARID1A,B2M,CIC,CRLF2,CTLA4,DICER1,EGFR,FAT1,GRIN2A,HIST1H3C,ICOSLG,IFNGR1,MDC1,MST1,PPP2R1A,RAD50,RAD54L,STAG2,TMPRSS2	0.877846816	1.024263828
path_1139	BCL10,CDH1,CDK4,CTLA4,DAXX,GNAS,HGF,HIST1H3E,IKBKE,INHBA,KDR,MAP3K13,MITF,MYOD1,PALB2,REL,RET,RFWD2,TAP2,TMPRSS2	0.807267726	1.035156548

path_1140	ACVR1,BRAF,CCND3,CDK4,CDKN2C,DIS3,DROSHA,ERG,FGF4,H3F3C,HGF,MDM2,MST1,PDCD1,PMAIP1,PNRC1,PTCH1,RAF1,RPS6KA4,TAP2	0.339507695	1.149707049
path_1141	ALOX12B,BARD1,BCL10,BLM,DDR2,DNMT3B,EIF4A2,IGF2,INHA,MET,MPL,MSH3,PPP2R1A,RAD21,SH2D1A,SHQ1,SMO,TERT,TET2,TP53	0.151102887	1.226081968
path_1142	ALK,ARID1B,ASXL2,ATRX,B2M,BLM,CENPA,CHEK2,DDR2,DROSHA,ERCC5,FAT1,FOXP1,GPS2,HIST1H3G,LATS2,MDM4,RASA1,RFWD2,RYBP	0.078608358	1.328547897
path_1143	AXIN1,B2M,BMPR1A,CDK6,CYSLTR2,EPHA3,EZH2,FGFR3,IDH1,MAP2K2,MYCN,NOTCH1,NTRK3,PAK7,PARK2,PBRM1,PIK3CG,PIK3R3,STK40,TCF3	0.337323365	1.160238745
path_1144	ARAF,BCL2,BRAF,CARD11,CDKN2A,ERRFI1,FGF19,FGF4,GRIN2A,INHBA,INSRR,IRF4,JUN,KIT,NFE2L2,NOTCH4,NUP93,PTPRS,RICTOR,STAG2	0.236235688	1.202980092
path_1145	ATRX,BRCA2,CEBPA,CENPA,CSF3R,CTCF,E2F3,EPHB1,HIST1H3J,MAP2K4,NCOA3,NF2,RB1,SETD2,SMARCA4,SOX2,SRC,SRSF2,STAG2,TRAF2	0.098320333	1.315330012
path_1146	ABL1,B2M,BRCA2,CCND1,CDC42,CDKN1B,CYSLTR2,ESR1,FGFR1,HIST1H3C,IFNGR1,INPP4A,MAP3K1,MSH6,NCOA3,NEGR1,PREX2,PTPRD,RB1,RFWD2	0.58882173	1.097299206
path_1147	ARAF,ATR,B2M,BCL2L1,CCND1,CDKN2C,CENPA,ELF3,FAM175A,FOXA1,INHA,KMT2D,NEGR1,NKX2-1,RAC2,RNF43,ROS1,SMARCA4,TCF3,TCF7L2	0.109907927	1.254485132
path_1148	AKT2,BCL10,CCND2,CD274,CDC73,CENPA,DOT1L,ERCC2,GLI1,HGF,MAP2K4,MSH2,NSD1,PTEN,PTPRS,RET,RNF43,RYBP,SDHAF2,SLX4	0.372354125	1.134453382
path_1149	AURKA,CDK4,EPHA3,ESR1,EZH2,FYN,IFNGR1,IRS2,JAK2,JAK3,MAP2K1,MGA,NOTCH2,PAX5,PIK3C3,PIK3CB,PRDM1,PREX2,RET,SUFU	0.060270679	1.384797722
path_1150	ABL1,BAP1,CDK12,CDK6,CDK8,CEBPA,CREBBP,EIF4E,GNA11,GPS2,IGF1R,MALT1,MDC1,NBN,NKX3-1,PHOX2B,PMS1,SMAD2,SMARCD1,TSHR	0.125919676	1.240664411
path_1151	ACVR1,ARID1A,DAXX,EIF4A2,ERG,FANCC,GPS2,HIST1H3J,HNF1A,INHBA,INPP4B,MDM2,MST1R,MTOR,PAK1,PTPRD,SMAD2,SOS1,SOX17,STAT5B	0.115153082	1.292666696
path_1152	APC,ARID5B,ASXL1,CDK12,CIC,DOT1L,EGFR,EIF1AX,FAT1,FBXW7,FGFR3,IGF1R,IGF2,MAP2K1,MAP2K4,MLL3,RAD51,RECQL4,SMAD4,SMARCA4	0.679747121	1.061058908
path_1153	ASXL1,CYSLTR2,DROSHA,EPHA7,GATA3,IRS2,JAK2,JUN,KIT,MED12,MEN1,MST1R,NSD1,PIK3CA,PPP6C,PTCH1,RASA1,SH2D1A,SMARCA4,SMARCD1	0.183973739	1.232363295

path_1154	APC,B2M,BARD1,BRD4,CD79B,CDK12,DNMT3B,EPHB1,FGF4,FGFR1,KEAP1,KMT2A,MAX,NOTCH2,PMAIP1,RAD21,RAD51,SMAD2,SOX2,SRC	0.506006265	0.897102076
path_1155	AKT2,ALOX12B,ANKRD11,ATR,BBC3,BTK,CDC73,CDK8,DROSHA,GLI1,HIST3H3,HLA-A,INPP4B,MAX,NOTCH4,PAK1,RAD21,RAD50,RAF1,SYK	0.276912541	1.16567812
path_1156	AR,BAP1,CDK8,CTNNB1,DAXX,DIS3,GNAQ,IRS2,KNSTRN,LATS2,MAP3K13,MDM2,MLL,NCOA3,PARK2,PTCH1,RARA,RNF43,SDHC,SH2D1A	0.820090428	1.032642831
path_1157	ALOX12B,BARD1,CDK4,DNMT1,ERCC5,FGF4,IGF2,IKBKE,KMT2A,MSH6,NOTCH2,NRAS,PDGFRB,PMS1,PRDM1,PTEN,RAD51B,ROS1,SDHAF2,TEK	0.171973374	1.214946022
path_1158	BCL2L11,BRCA2,BRIP1,CDC73,CSF3R,DAXX,DNMT1,GNA11,INSR,PDCD1,PIK3R2,PLK2,PMS1,PTPRS,RHEB,RNF43,RUNX1,RYBP,TAP2,TNFAIP3	0.423393675	1.121170987
path_1159	ARID5B,BCOR,BTK,CDKN2C,CHEK2,DDR2,EPHA7,FGFR2,HIST1H3J,INSR,JUN,NPM1,NTRK2,PIK3R2,RAD54L,RASA1,SOX17,TEK,TNFAIP3,TP53BP1	0.402518984	0.888537095
path_1160	AKT2,ALOX12B,CDK8,CDKN1A,EZH2,FGFR3,IDH1,INPP4B,INSR,IRS2,KRAS,NCOA3,NFKBIA,PBRM1,PDGFRA,PIK3R1,PLK2,PTCH1,SDHAF2,SOX17	0.988191083	1.00231198
path_1161	CRLF2,EZH2,FANCC,FGF19,GRIN2A,IL7R,INPP4B,KMT2D,MCL1,NKX2-1,NPM1,NRAS,RARA,RB1,SDHC,SH2D1A,SRC,TERT,TGFBR1,TP53	0.457464882	0.900525868
path_1162	BAP1,DDR2,EIF4E,ETV1,GATA1,HIST1H3I,IRF4,KDM5C,KDM6A,MDM4,MUTYH,NOTCH2,PIK3CG,PPM1D,PPP2R1A,RAD50,SMAD4,SMARCD1,SYK,TNFAIP3	0.445643023	1.113659832
path_1163	AR,CDK8,CDKN2A,CDKN2C,CEBPA,ESR1,FOXO1,FUBP1,GSK3B,JAK3,NCOA3,NKX2-1,PTCH1,RPTOR,SF3B1,SOCS1,SOX17,SRC,TAP2,WT1	0.242861107	1.179211573
path_1164	BCL10,CDC42,CDKN1B,CTNNB1,CYSLTR2,DNMT3B,EGFR,HIST1H3A,HIST1H3C,HOXB13,INSRR,NCOA3,NFE2L2,NKX3-1,PIK3CB,RHEB,RNF43,ROS1,RTEL1,SDHAF2	0.814645921	1.045904932
path_1165	ARID2,BRAF,DAXX,DNMT3B,DOT1L,EPHA3,FOXO1,HIST1H3B,IRS2,LATS2,MDC1,MLL3,MYC,NKX2-1,PTCH1,SETD2,SH2D1A,SMAD3,TAP2,TBX3	0.355857895	1.143863462
path_1166	ALK,ARID1A,BAP1,DROSHA,ERG,GATA2,GRIN2A,GSK3B,HIST1H3J,MAP2K1,PDCD1,PIK3R1,PMS1,POLE,PTPRD,RAD51D,SDHAF2,TEK,TP63,TRAF7	0.315899063	1.16456648
path_1167	BRAF,CD79B,DAXX,ERBB2,ERRFI1,FBXW7,FLT4,FOXL2,INSRR,LATS1,MTOR,NKX2-1,NPM1,PIK3R2,RAD50,RAD51C,RB1,SHQ1,SPEN,STAT3	0.984298498	0.996738482

path_1168	AKT2,AKT3,AMER1,ARID2,CALR,CASP8,ELF3,FGFR3,GNAS,HIST1H3C,HIST1H3E,HIST3H3,IDH1,INSRR,JAK2,KEAP1,MCL1,NOTCH2,RHOA,SLX4	0.88484815	1.026297452
path_1169	ARID2,AURKA,BBC3,CD274,CDK12,DNMT1,FANCC,FGF4,HIST3H3,IGF1R,KRAS,MAP3K13,MSH6,NSD1,PREX2,RTEL1,SDHAF2,SHQ1,STAT5B,TEK	0.525620718	1.129245669
path_1170	BRCA2,CDC73,CDKN2C,CTCF,EIF4E,ERBB4,FANCA,FGF3,FLT4,FOXL2,HIST1H1C,HIST1H3G,IRF4,MLL2,MST1,NF1,NTRK3,SH2D1A,SOX17,SPEN	0.532101858	1.101763683
path_1171	BCOR,BRCA1,CDC42,CDC73,CTCF,CTNNB1,CUL3,FGFR3,FGFR4,FLT1,HLA-A,INSR,KDM5A,MALT1,NF1,NSD1,PTPRD,RAD54L,REL,SOS1	0.202328403	1.223141876
path_1172	BRCA2,CDC42,CDH1,CIC,EIF4E,EZH2,FGF19,GATA1,GATA2,GRIN2A,JUN,KIT,MAX,MED12,PIK3CA,PNRC1,PRDM1,SDHC,SF3B1,SMAD2	0.075748926	1.386629575
path_1173	AKT1,B2M,BCL10,CD274,ERBB2,FANCA,FYN,H3F3C,HIST1H3C,KDM5C,MPL,MST1,NTRK1,PDGFRA,PDGFRB,PIK3C2G,PRDM1,SOCS1,SUFU,TCF3	0.118401887	1.248161907
path_1174	ACVR1,ALOX12B,BCL6,CDKN2A,EIF1AX,FANCC,FUBP1,HIST1H3I,HLA-A,JAK3,MSH2,MYCN,NOTCH4,NTRK2,PAK7,POLE,RAF1,RNF43,SRSF2,STAT3	0.483439558	1.12747852
path_1175	ARID5B,CSF3R,CTLA4,DOT1L,EIF4E,FBXW7,HOXB13,IGF2,KDM6A,MAP2K1,MCL1,MET,MYCL1,NCOR1,PAK1,PDGFRB,PPP6C,RHOA,SH2B3,SRSF2	0.688548866	1.058155731
path_1176	ANKRD11,AR,ARID1B,ATR,DAXX,DOT1L,FANCA,FGF3,FGFR1,FGFR2,GSK3B,H3F3C,PBRM1,PIK3CB,PIK3R3,PMS1,PNRC1,PTCH1,RASA1,RPS6KB2	0.104321802	1.25823013
path_1177	APC,CEBPA,CTLA4,CUL3,EPHB1,EZH2,FANCC,FOXP1,INPP4A,KDM5C,LATS2,MEF2B,MRE11A,NF1,NKX2-1,PDCD1,PLK2,RAD51D,RAD54L,ROS1	0.71178306	1.064689582
path_1178	APC,AXIN2,CBL,CD274,CDK4,CTLA4,HIST1H3A,IDH1,INSRR,KMT2D,MAX,NSD1,PAK7,PIK3CA,PPP2R1A,PTPN11,RECQL4,SMAD2,SPEN,STAT5B	0.461571856	1.14709126
path_1179	AKT1,ALK,BBC3,BMPR1A,BRAF,CARD11,CD274,EPHA3,ERBB4,FOXA1,ICOSLG,MDC1,MITF,MYCN,PIM1,PNRC1,RNF43,SMAD4,STK11,SUFU	0.15763726	1.221101691
path_1180	AKT2,ARID2,CENPA,ERCC4,HIST1H3H,IKBKE,JAK1,KDM6A,MAP2K4,MYCL1,NOTCH4,PIK3CA,RTEL1,RYBP,SH2B3,SH2D1A,SMAD4,SRC,TGFBR2,TMPRSS2	0.43058558	1.145684152
path_1181	ATR,BAP1,BBC3,BRCA1,CDKN1A,DNMT3B,EED,ERBB2,INHA,INSR,KLF4,MET,MUTYH,NRAS,PTPRS,RBM10,RFWD2,STAG2,TET1,TMPRSS2	0.204142785	1.225683222

path_1182	AKT3,ALK,ARID1A,ARID2,ASXL1,EPHA5,ERCC2,ERRFI1,FGFR3,FGFR4,HIST1H3H,HIST3H3,IKZF1,KLF4,MSH2,NKX3-1,PIM1,RBM10,SMAD3,SMARCB1	0.16268256	1.245277806
path_1183	ALOX12B,BCOR,BRIP1,CCND1,DICER1,EED,ERBB2,ERBB3,EZH2,FAM46C,FGFR3,HIST1H3E,IKBKE,IL10,KLF4,MDM4,NF1,NKX2-1,RPTOR,SPOP	0.116054467	1.344698381
path_1184	ABL1,CD276,CDK8,CUL3,FGF19,FGFR1,INHA,INPP4B,INSR,MPL,MYCL1,NFE2L2,NOTCH1,NTRK3,PIK3CA,RET,RHOA,RPS6KA4,SDHA,TAP2	0.162122101	1.262078393
path_1185	ARID1B,BBC3,CD274,CTLA4,DNMT1,ELF3,ESR1,FGF3,FGFR4,INSR,NCOR1,NSD1,NUP93,PMS1,PREX2,RAD21,RBM10,RNF43,SMAD4,SOX17	0.933830004	1.013589782
path_1186	AKT2,ATM,AXL,B2M,CD79B,ELF3,EPHA5,ETV6,FGFR4,KDM5A,KEAP1,KNSTRN,PDCD1,PIK3CG,PIK3R3,RAD54L,SOX2,SRSF2,TNFAIP3,TRAF2	0.724605205	1.05323503
path_1187	BRD4,CALR,CARM1,CBL,CDKN1A,FANCC,GATA1,IKZF1,INSRR,KLF4,MSH2,NKX2-1,NOTCH4,NRAS,PAX5,PREX2,RAC2,RAD54L,SOCS1,STK40	0.506893438	1.099649629
path_1188	AKT2,ALOX12B,BRCA1,BRIP1,CASP8,CTNNB1,ETV1,HIST1H3C,HIST1H3G,HIST1H3H,KEAP1,MDC1,MDM2,NSD1,NUP93,PMAIP1,PREX2,RICTOR,SPEN,TAP2	0.357724959	1.160960671
path_1189	ATM,ATR,AXIN2,B2M,BRD4,CALR,CD79B,CDC42,CXCR4,DNMT1,FAT1,FGF4,FGFR1,JAK1,JAK2,NTRK3,RASA1,SMAD4,STAT5B,STK11	0.350659892	1.146699958
path_1190	AKT3,ARID2,ASXL2,BCL6,CDK12,CENPA,CTLA4,FGF4,HIST1H3E,KDM5C,MAP2K4,NPM1,PIK3CB,PPP2R1A,PRDM1,RAC2,RTEL1,SDHA,SOS1,STK11	0.245184187	1.231095667
path_1191	AKT2,BCL2,CCND1,CCND2,EIF1AX,ETV6,FANCA,FOXP1,GNA11,INHBA,JAK2,MAP3K1,MPL,MST1R,NCOR1,PPM1D,PREX2,RPTOR,SMARCD1,TAP1	0.469901083	1.108296927
path_1192	ABL1,BCL2L1,CARD11,FANCC,FAT1,FGF19,FLT4,FYN,GLI1,GPS2,HIST1H3G,HNF1A,IL7R,MAX,NOTCH2,PTEN,RNF43,RPS6KB2,SMARCD1,TBX3	0.234373967	1.206780884
path_1193	AKT1,ATR,BCL6,BLM,CCND3,DNMT3B,FLT3,FOXA1,GPS2,HIST1H1C,HIST1H3I,HOXB13,IFNGR1,IL7R,MSH2,NKX2-1,PAK1,PIK3C3,RAC2,SRSF2	0.86902663	1.023640149
path_1194	ARID1B,ATM,BMPR1A,BRAF,CALR,CDKN1A,CDKN1B,EPHA5,FAT1,GLI1,HIST3H3,IKZF1,JAK2,KEAP1,KNSTRN,MYC,PIK3CD,PTPRT,SDHB,SH2D1A	0.554184593	0.919071433
path_1195	ACVR1,AKT3,ALK,ATR,AXIN2,CCND2,CDK4,CIC,DROSHA,ETV1,LATS1,MUTYH,NBN,NUP93,RHEB,SMARCD1,SPOP,STK11,TSHR,YES1	0.335451813	1.162737171

path_1196	BCL2,CASP8,CDK12,CDK8,DDR2,DNAJB1,EIF4A2,EPHA5,FGFR2,FOXP1,HIST1H3C,HIST1H3E,MALT1,MYOD1,NKX2-1,PIK3CB,PIK3CG,RAC2,RAF1,RPS6KB2	0.138965821	1.231358647
path_1197	ABL1,AKT2,ALK,ANKRD11,ATR,CCND3,CREBBP,CTNNB1,FOXO1,HNF1A,KMT2D,MAP2K1,MRE11A,MYC,NKX3-1,PBRM1,PREX2,RAD54L,RFWD2,STAT5B	0.498139447	0.890542241
path_1198	BAP1,CBL,CEBPA,EIF4E,ERCC4,ETV1,FOXO1,GATA2,IRS2,KDM6A,LATS1,MAP3K1,MDM4,NF2,RAD54L,REL,SDHB,STAT5B,TEK,TGFBR1	0.141751385	1.230443114
path_1199	ARID2,ASXL2,ATM,CCND2,CXCR4,CYSLTR2,FANCA,GLI1,HIST1H3G,IDH1,JUN,KRAS,MLL2,PGR,PIK3CD,RBM10,RICTOR,RPS6KB2,RUNX1,TGFBR1	0.459315197	1.110349471
path_1200	BARD1,BRCA1,CARD11,DNMT1,DNMT3B,E2F3,EP300,ERCC2,ERCC5,HIST1H3E,ICOSLG,KMT2D,MSH2,MYCN,NFE2L2,NTRK2,RARA,RPTOR,TMPRSS2,TSHR	0.717104449	1.070241791
path_1201	APC,ATM,CCND1,CREBBP,DNMT3A,ESR1,FBXW7,HIST1H3G,NCOA3,PALB2,PDGFRB,PHOX2B,PTCH1,RAD51D,RAD54L,RICTOR,ROS1,SMO,SRC,STK11	0.5595828	0.91851504
path_1202	ABL1,ARAF,AXL,CCNE1,CD276,CTCF,GNAQ,HIST1H3J,IGF2,INHBA,INSR,IRS2,JAK3,KMT2A,KMT2D,NCOA3,NCOR1,PARP1,PLCG2,RAD21	0.320380096	1.153405469
path_1203	ARID2,ATRX,B2M,CCND1,CCND2,CDH1,EP300,EPHB1,FOXL2,HGF,HIST1H1C,KDM5A,KDM6A,MALT1,PIK3CB,RAD54L,RECQL4,RFWD2,SETD2,SHQ1	0.12517166	1.295145287
path_1204	ABL1,ASXL1,BCL2L1,CARM1,DICER1,DNMT1,EPHB1,ERBB4,INHA,MDC1,MLL2,MPL,NF1,PARP1,PIK3CG,PIK3R3,SF3B1,SHQ1,TBX3,TOP1	0.218125057	1.206879149
path_1205	AXIN2,CHEK2,CYSLTR2,DDR2,ERCC5,ETV6,FAM175A,FH,FLT3,GATA3,HIST1H3C,HNF1A,KDR,MDM2,MSH3,MYC,PARP1,PPP6C,RYBP,SMARCB1	0.087506433	1.283945799
path_1206	ARID1B,CDC73,CREBBP,FLT1,GRIN2A,GSK3B,HIST1H3G,IDH1,IRS1,JAK3,MAP3K1,MITF,MUTYH,PAK7,RAD51D,SETD2,SMAD3,SMARCA4,SOS1,TSHR	0.081085492	1.295697257
path_1207	BARD1,ERCC4,FLT1,FLT4,FOXL2,IDH1,IGF2,INHA,LATS1,MTOR,MYCL1,PAK1,PHOX2B,PIK3C3,PIK3CD,PIK3R3,RAD51,SRSF2,TBX3,TCF3	0.558092278	1.110687573
path_1208	AKT2,BARD1,CBL,CCNE1,CDKN1A,FGF4,HLA-A,INSRR,MAP3K13,MED12,MRE11A,NF1,PDCD1,RAF1,RARA,RHEB,RUNX1,SOCS1,TET2,TMPRSS2	0.116645238	1.248064545
path_1209	BCL2,BCL2L11,CDC73,CUL3,DNMT1,E2F3,FAM46C,GSK3B,HIST1H3J,MLL2,PDGFRB,PTPN11,ROS1,SDHB,SDHC,SHQ1,SOCS1,SYK,TCF3,TEK	0.12285395	1.245543149

path_1210	AR,BAP1,BMPR1A,CALR,CCND2,CRLF2,EIF1AX,ERRFI1,HIST1H3B,HIST1H3G,HIST3H3,INSR,KRAS,MDC1,MTOR,NEGR1,PDCD1,RAC2,SMARCB1,TCF3	0.881893194	1.023450545
path_1211	AURKA,CBL,CDK4,CTLA4,CUL3,CXCR4,EGFR,EPHA3,EPHB1,ERBB3,ERRFI1,FOXP1,FUBP1,GNAS,ICOSLG,INPP4A,INPP4B,SH2B3,SOCS1,STK11	0.912352269	1.016614713
path_1212	CREBBP,DDR2,EPHA7,ERBB2,ERBB4,HIST1H1C,HIST1H3G,HNF1A,ICOSLG,IGF2,IKBKE,MAP3K13,MYOD1,NFE2L2,NKX3-1,PLCG2,PTEN,SDHAF2,TEK,TSC2	0.529314403	1.107632036
path_1213	ALK,ARID1B,BARD1,CCND3,DNMT3A,EIF4E,ELF3,ERCC5,FANCC,GSK3B,HIST1H3A,NF1,NFE2L2,NSD1,NUF2,PLK2,RASA1,SOCS1,SPEN,SPOP	0.445689734	1.139396558
path_1214	ABL1,AR,BAP1,BCL6,CDK4,CDKN1B,DROSHA,EPHB1,FGF4,HIST1H3J,KDM5A,KRAS,LATS1,MAP2K2,MYCN,NFE2L2,NRAS,RPS6KA4,RUNX1,TAP1	0.816757698	1.035683353
path_1215	ATRX,CALR,CBL,CDK4,CDK8,CHEK2,CTNNB1,DNAJB1,EP300,FOXA1,GNAQ,HIST1H3A,IKBKE,JAK3,JUN,KRAS,NBN,PREX2,RASA1,SOX9	0.07642038	1.33432475
path_1216	ARID1A,CDK12,DNMT3B,EIF4E,FANCA,FGF3,HIST1H3G,IGF1R,IKBKE,INSRR,KNSTRN,MST1R,MYC,MYOD1,NEGR1,NOTCH1,NRAS,PIK3R2,RAD21,SMAD3	0.078010814	1.35404969
path_1217	AMER1,ARAF,ASXL1,BCL10,BCOR,CD274,EIF1AX,ERG,FAM46C,HOXB13,KIT,MAP2K1,MLL,MYOD1,NF1,PMAIP1,PMS1,RASA1,SMAD4,STAT5B	0.111679656	1.303482645
path_1218	APC,ATM,B2M,BAP1,CDK4,ERCC2,FGFR4,HIST1H3D,HIST1H3G,HLA-A,INPP4A,MLL3,NCOR1,PAK1,PIK3CA,PLK2,RAC2,RHEB,SMAD3,SMAD4	0.666411715	1.068463116
path_1219	CDK6,DNMT1,FANCA,GRIN2A,HNF1A,IKZF1,MAP3K1,MDM2,NF1,NUF2,PARK2,PIK3C3,PNRC1,RET,RFWD2,RNF43,RYBP,SDHA,SMAD3,SUFU	0.313624626	1.186320703
path_1220	ATR,BAP1,BCL10,BCL2L11,BLM,BRIP1,CCND2,CRLF2,FUBP1,GLI1,INSR,MGA,MITF,NEGR1,NOTCH3,PALB2,PIK3R2,PPP2R1A,RET,SUFU	0.073146715	1.375801165
path_1221	AKT2,ALOX12B,FGF3,HIST1H3G,HIST3H3,INSRR,LATS1,NOTCH2,NTRK1,PAK1,PALB2,PARK2,PNRC1,POLE,PRDM1,RPTOR,SLX4,SMARCD1,STK40,TGFBR1	0.286020195	1.165040886
path_1222	AXIN1,CCNE1,CTCF,DNMT3B,EPHA7,EPHB1,ERG,FBXW7,FGFR1,GNA11,HIST1H3A,ICOSLG,IRS1,MSH3,NUF2,PMS2,PTPRD,RHEB,SDHA,SDHAF2	0.373435272	1.167062034
path_1223	AKT1,ASXL1,ASXL2,AURKA,BAP1,CDK4,CDKN1A,CIC,EP300,ERG,FGFR4,FLT3,HIST1H3G,IL10,MDC1,PIK3CG,RNF43,STAT3,TSC2,WT1	0.188647487	1.265146207

path_1224	ANKRD11,ATR,BLM,DAXX,ERRFI1,ETV1,FANCA,FANCC,H3F3C,HLA-A,KDM5A,MYOD1,NOTCH4,PIK3C3,PIK3CB,PNRC1,PTPRT,RBM10,ROS1,SPOP	0.38224105	1.150062909
path_1225	ARID1B,BCL10,CDK4,CDK8,CDKN2A,CENPA,FGFR4,INHA,INPP4A,MDM2,MTOR,MYCL1,NEGR1,PAK7,PIK3CA,PIK3R3,PTPN11,RAD51B,SMAD4,TAP1	0.678389027	1.076341981
path_1226	AMER1,AR,ARID2,BARD1,DNMT3A,ERBB2,EZH2,HIST1H3A,KDM5A,MAP2K4,MEF2B,NOTCH3,PIK3C2G,PMS1,PTCH1,RAD21,RPS6KA4,SDHB,STK11,TGFBR2	0.464987311	1.113179399
path_1227	BTK,CCND2,CD276,CD79B,CDKN1B,EP300,ERG,FLT1,GRIN2A,HGF,JAK2,KMT2A,MSH3,NCOA3,NFE2L2,PALB2,RUNX1,SOCS1,SOX2,STK40	0.585754328	1.103743656
path_1228	ASXL1,ATRX,DIS3,HGF,HIST1H3A,IKBKE,INPP4B,KDM6A,KIT,MAP2K4,MAX,MLL3,NFKBIA,PIK3CG,POLD1,PTCH1,RAD50,SMAD3,SUFU,TBX3	0.050834614	1.336420125
path_1229	ARID5B,ASXL1,CCND2,CDK6,CEBPA,EP300,INHA,INHBA,IRF4,MAP2K1,MITF,NSD1,PIK3R3,PRDM1,RAF1,REL,SMARCB1,SOCS1,SRC,STK40	0.348089589	1.142622141
path_1230	AKT1,AKT3,ALOX12B,ARAF,BMPR1A,CTLA4,DROSHA,EPHB1,ESR1,FGF3,FOXA1,GPS2,HIST1H3E,JAK2,KIT,MSH2,NRAS,REL,RPS6KA4,SYK	0.737195814	1.051130431
path_1231	CBL,CDK12,CENPA,CTNNB1,ERCC5,GATA3,HIST1H3H,MDC1,MLL,MYCL1,NKX3-1,NOTCH2,PDGFRB,RAD51D,RFWD2,RHOA,SETD2,TET1,TP63,YAP1	0.125440967	1.318791621
path_1232	BLM,BRCA1,CARM1,CBL,CD274,CDC42,EGFR,ERG,FANCA,FAT1,FLT3,GRIN2A,HIST1H3A,HIST1H3C,IL10,INHA,KDR,NOTCH4,RBM10,TGFBR2	0.698760439	0.944800837
path_1233	BAP1,BTK,CTLA4,HGF,HIST1H3C,IKBKE,INSRR,KDM5A,KIT,KLF4,MAP3K13,MRE11A,NFE2L2,NOTCH4,NUF2,NUP93,PIK3CD,PTPRS,RASA1,RB1	0.74358236	1.055387628
path_1234	ATM,BAP1,ERBB2,ERG,FGF3,GRIN2A,IRF4,IRS2,JAK3,KDM5A,MLL2,MSH2,NKX3-1,PBRM1,PHOX2B,PIK3CA,PIK3R3,RHEB,SHQ1,SMO	0.898702039	1.020170908
path_1235	B2M,BRCA1,BRIP1,CDK8,ELF3,GATA1,GSK3B,H3F3C,HIST1H3A,HIST1H3I,JAK1,KDM5A,MED12,NFE2L2,PTPRT,RET,SDHAF2,SHQ1,SMAD2,SMARCA4	0.136228507	1.277014175
path_1236	ARID5B,BARD1,BBC3,BRAF,CBL,CD79B,CRLF2,DICER1,DROSHA,EED,ELF3,IGF1R,IKBKE,MAP3K13,PTPRD,RET,TCF7L2,TEK,TET2,TRAF7	0.074610919	1.362397515
path_1237	ETV1,HLA-A,IRS1,KMT2D,MDM4,MLL,NF1,NKX3-1,NOTCH1,NTRK2,PARK2,PIK3R2,RAD51B,RAD54L,RAF1,RASA1,RHEB,SDHA,SETD2,SMAD4	0.410787431	1.148612064

path_1238	AKT3,CD276,EPHA3,ERCC2,FLT4,HIST1H3J,IDH1,IKBKE,IKZF1,JAK1,MYC,NSD1,PDGFRA,PIK3C3,PMS1,RBM10,RECQL4,RET,RHEB,ROS1	0.114508871	1.273338469
path_1239	AMER1,CDK6,CEBPA,CENPA,CTLA4,FH,H3F3C,HIST1H3C,HIST1H3H,IFNGR1,INHBA,JAK1,NKX3-1,PIK3CG,PREX2,PTPRS,RAD21,RAD51B,ROS1,TGFBR2	0.2121356	1.19406493
path_1240	BCL10,CARM1,CDK6,CDKN2A,DNMT3B,EP300,FGFR2,IKZF1,MAP3K13,MAX,MST1,PHOX2B,PIK3R3,PMAIP1,PTPRD,RAD50,RAD51C,SH2B3,SMARCD1,SYK	0.166628737	1.293546602
path_1241	ATR,CDC42,CDKN1A,CTNNB1,EPHA5,ESR1,INHBA,MALT1,MGA,MLL,PAK1,PAK7,PIK3CB,PPP6C,RAD51B,RYBP,SMO,TERT,TNFAIP3,WT1	0.349339935	1.152385463
path_1242	AKT3,AMER1,AXL,BRCA1,BTK,CXCR4,ERBB2,FANCA,FAT1,FLT4,FOXP1,GATA3,KDM5C,KMT2A,MYCL1,NKX2-1,NUF2,PIK3R1,RNF43,TET1	0.063256989	1.340167255
path_1243	AURKA,BCL2,BRCA2,BRD4,CARM1,CDC42,CHEK2,DIS3,DNAJB1,EED,ERCC2,FGF4,IKZF1,LATS2,MDC1,MYOD1,NUF2,RPS6KA4,SH2B3,TSHR	0.528444715	1.096548261
path_1244	AXL,BCL2L1,BCOR,BMPR1A,CDC42,CREBBP,CUL3,EIF4A2,EPHB1,EZH2,HIST1H3H,IRS1,MEF2B,MST1R,NFKBIA,NOTCH2,PALB2,PTPN11,RPTOR,SH2D1A	0.277624009	1.165555394
path_1245	ASXL1,AXIN1,B2M,CD79B,ERCC2,EZH2,FAM46C,FAT1,FGF3,FH,GRIN2A,HIST1H1C,KLF4,MITF,MLL3,PALB2,PARK2,PIK3R1,RAD51,RAD54L	0.324040369	1.158878593
path_1246	AKT3,ALOX12B,AMER1,ARID1B,BCL6,CCND2,ERCC2,FGF19,HGF,HIST1H1C,ICOSLG,MITF,MYCN,NRAS,PARP1,PIK3R2,TBX3,TGFBR2,TP53BP1,XPO1	0.747488781	1.06059027
path_1247	ASXL1,ATR,AXIN1,BBC3,CBL,CSF1R,EED,FOXA1,FYN,GATA1,GPS2,IL10,KDM5C,MDM2,MUTYH,NOTCH2,PIK3C2G,RBM10,SDHC,SMO	0.135927146	1.280353424
path_1248	ALK,AR,ASXL1,BBC3,BTK,CXCR4,EGFR,ERBB3,ERCC5,GATA3,HIST1H3A,HIST1H3C,HIST1H3D,MAX,MRE11A,PBRM1,RASA1,SOCS1,TP53BP1,TRAF7	0.130775594	1.306039072
path_1249	BCOR,CCND1,CDC73,ERCC2,FBXW7,FLT1,FOXA1,GNA11,GRIN2A,ICOSLG,MAX,MCL1,MEF2B,NBN,PIM1,PLCG2,PTPN11,RET,STK11,STK40	0.299620577	1.177010889
path_1250	AMER1,AXIN1,CCND3,DOT1L,ERCC2,FANCC,GNA11,INHBA,KMT2A,MAP2K4,MRE11A,NFKBIA,PAK7,PDGFRA,PIK3C2G,PIK3CG,SMARCA4,SMARCB1,TBX3,TCF3	0.135893014	1.254286194
path_1251	ARID1A,BBC3,CDH1,E2F3,EED,EIF1AX,EZH2,FGF19,HIST1H3C,IKBKE,INSRR,MEF2B,MLL2,NFKBIA,NOTCH2,PNRC1,PREX2,PTCH1,SHQ1,SLX4	0.185749364	1.207765294

path_1252	ALOX12B,BCL2L11,CARM1,CCND2,DICER1,EP300,GRIN2A,HIST1H3H,HIST1H3J,HOXB13,IKZF1,MEN1,MRE11A,NCOR1,NOTCH4,NUP93,RB1,RHOA,SLX4,TP53	0.684727147	1.059098578
path_1253	BTK,CD79B,CDK12,CREBBP,EED,EIF1AX,EPHA5,ERBB3,FYN,ICOSLG,INSRR,JAK3,KNSTRN,NOTCH4,NUP93,RBM10,RFWD2,RHEB,TET2,TP53BP1	0.32825278	1.167624316
path_1254	ARID5B,BRCA2,BTK,CCND2,CSF1R,EGFR,FANCC,FAT1,FGF19,FGF4,FGFR1,FOXO1,GLI1,HIST1H3I,HIST3H3,INSR,MALT1,NCOR1,NF1,PTPRD	0.501827422	1.102414356
path_1255	ATM,BCL2,BRAF,DNMT3B,EPHA3,FAM175A,FGFR3,GPS2,HIST1H3I,IDH1,INHBA,KDM5C,MET,MYCN,MYOD1,NOTCH1,PIK3CG,PRDM1,RASA1,RPTOR	0.662766085	1.065419168
path_1256	AKT1,AXIN2,CREBBP,CXCR4,EPHA7,HIST1H3G,HLA-A,JAK1,MAP2K1,NOTCH1,NOTCH3,PDGFRA,PLCG2,SMARCA4,SOS1,SRSF2,STAG2,SYK,TET1,TMPRSS2	0.0799915	1.312980316
path_1257	ACVR1,AMER1,BBC3,BRCA1,CYSLTR2,EIF4E,ERBB2,FGFR4,HIST1H3B,HOXB13,JUN,KLF4,MAP3K13,MST1R,NKX2-1,NKX3-1,PAK7,RICTOR,SDHA,TGFBR1	0.263307469	1.171923359
path_1258	B2M,CD79B,CUL3,DNMT3A,EGFR,ERBB4,ERG,ESR1,FANCA,GRIN2A,HIST1H3J,HNF1A,KNSTRN,NTRK1,PIK3R1,RARA,RNF43,SETD2,SH2B3,SH2D1A	0.150836664	1.252062125
path_1259	ANKRD11,APC,ATR,ATRX,BCL10,BMPR1A,CCND2,CXCR4,EGFR,EPHA5,IRF4,KEAP1,PDGFRB,PLCG2,PTPRD,RAD21,RAD50,RHOA,RNF43,RPTOR	0.504639209	1.098543705
path_1260	AKT1,FAM46C,FGFR3,GLI1,HIST1H3B,HIST1H3H,MAP2K2,MITF,NBN,NUP93,PIK3C2G,PMS1,PPP2R1A,RAD51,RAD51D,SMARCD1,SOX9,TAP1,TAP2,TP63	0.098413724	1.273430105
path_1261	CARD11,CCNE1,CD274,CDC73,CDK4,DNAJB1,EGFR,ELF3,ERBB2,FGF19,FGFR4,FYN,GNAQ,HOXB13,IRS2,MAP3K1,MYCN,NFE2L2,PIK3R3,PLCG2	0.094917409	1.340191328
path_1262	AXIN2,BCL10,CDK12,DROSHA,EIF4E,FGFR1,FGFR3,FH,HIST1H3E,IRF4,IRS1,MAP2K1,MAP3K13,MAX,MDM2,MEF2B,MPL,NKX2-1,NSD1,PPP6C	0.977021216	1.004104344
path_1263	ARAF,BRAF,CD276,CD79B,DICER1,EIF1AX,FANCC,HGF,HIST1H1C,HIST1H3I,HNF1A,NUF2,PALB2,PIK3CB,PIM1,PPP2R1A,RYBP,SDHAF2,SMAD2,SMAD4	0.677545058	1.081867772
path_1264	ABL1,AKT1,ASXL2,AXIN1,BCL2L11,ERCC5,ETV1,FLT1,FOXO1,INPP4A,MAX,MDC1,MITF,MSH3,NCOR1,PIK3C2G,PTPRS,RHEB,SOX17,TSC2	0.098453525	1.331944811
path_1265	AMER1,ATM,CD79B,CDK4,CENPA,EZH2,HNF1A,INSR,JAK1,MCL1,MPL,MUTYH,NPM1,PMS2,PRDM1,PREX2,SLX4,STAT3,TMPRSS2,TSC1	0.83235717	1.030949505

path_1266	ABL1,ARID5B,CDK4,CDKN1A,FAM175A,FGFR1,FGFR3,FLT3,FOXA1,GLI1,HIST1H3I,KEAP1,KRAS,MDM2,MST1,MYCL1,PIK3R2,PRDM1,RFWD2,SETD2	0.95663566	1.007716736
path_1267	ABL1,APC,ARID5B,CDC42,CDC73,FYN,GATA2,HIST1H3B,HIST3H3,HLA-A,HNF1A,INSRR,MET,MST1,NOTCH1,PDCD1,PTPN11,RHEB,SHQ1,SMAD2	0.852631506	0.96720978
path_1268	CDKN2A,CTCF,DIS3,ERBB4,ERCC2,FANCA,FGFR3,MAP2K4,MET,MGA,MYCN,NFKBIA,PAK1,PLCG2,PLK2,PNRC1,PTCH1,PTPRD,RNF43,TAP2	0.468699356	1.113507889
path_1269	BCL2L1,CD276,DDR2,EIF4A2,ETV6,FGF3,IFNGR1,IKBKE,MST1R,NKX2-1,PMS2,PREX2,RAD54L,ROS1,RYBP,SDHA,SH2B3,SOCS1,SOX9,SYK	0.053493906	1.321962711
path_1270	ARID2,AXIN2,BRAF,FAT1,HLA-A,ICOSLG,IDH1,INSRR,JAK2,NOTCH3,NRAS,PIK3C3,PIK3CB,RAD54L,RECQL4,RFWD2,RPS6KB2,SPOP,TOP1,WT1	0.238430857	1.214057345
path_1271	ACVR1,BLM,CASP8,EP300,ERCC4,ERCC5,GLI1,JAK2,KLF4,KMT2D,MAP2K1,MCL1,MDM2,MSH2,NF2,PLCG2,RAD51C,SOX9,TCF3,TGFBR2	0.054126097	1.310345982
path_1272	ANKRD11,BCL2L11,DNMT3B,FAM175A,FGFR2,GLI1,HOXB13,MDM4,MITF,NCOR1,NKX2-1,NTRK3,PGR,PPM1D,RTEL1,SH2D1A,SOX17,SRC,TAP1,TET1	0.16622915	1.217305464
path_1273	AXL,CBL,CRLF2,EIF1AX,EIF4A2,ERBB4,FANCC,FLT3,HIST1H3G,IGF2,INPP4A,KDM5C,PGR,PLK2,POLE,RARA,RPS6KA4,SDHAF2,STAG2,TSC1	0.089242449	1.36214905
path_1274	ATM,CD79B,CHEK1,ERBB4,FANCA,GPS2,HIST1H3J,IGF2,IL10,IL7R,JAK1,KDM5A,LATS2,MDC1,MEF2B,PHOX2B,POLD1,RAD51D,RAD54L,RNF43	0.23075172	0.815104787
path_1275	BCOR,BMPR1A,CXCR4,DNMT3A,E2F3,FH,FYN,GATA2,GNAQ,INSR,JAK2,JAK3,MEF2B,MYCN,NBN,PALB2,PAX5,PTPRS,RFWD2,SRC	0.197230109	1.314046437
path_1276	AKT1,ATRX,CDC42,CTNNB1,FAM46C,FUBP1,GATA3,GSK3B,LATS2,MAP3K1,MSH2,MYC,NUP93,PPP2R1A,PPP6C,PTPRT,RAD50,RAD51B,RET,RFWD2	0.256209214	1.206222738
path_1277	APC,ASXL1,AURKA,CSF1R,CTCF,EIF4E,INSRR,IRF4,JAK2,MDM4,MSH3,NPM1,PDGFRA,PMS1,PNRC1,RECQL4,RPS6KB2,SMAD3,SOS1,TCF3	0.688720582	0.944339342
path_1278	ARID2,AXIN1,BCL6,CDC42,DNMT3B,ERCC4,FANCC,GNA11,GRIN2A,HLA-A,HNF1A,INPP4A,IRF4,JAK2,MED12,MRE11A,NUF2,PDGFRB,RAD50,SMARCB1	0.076463216	1.28359504
path_1279	AXIN2,BMPR1A,CDC42,CDKN2C,CENPA,CXCR4,ERRFI1,FANCA,KMT2A,KNSTRN,MDM2,MDM4,NUP93,PMS1,PMS2,PNRC1,SDHB,SHQ1,SMO,SPEN	0.110775328	1.264881879

path_1280	ARID2,BARD1,BTK,CCND1,CDKN2C,DNAJB1,ETV1,IKZF1,INPP4B,JAK1,NRAS,NTRK1,PIK3R3,POLD1,RAD54L,RASA1,RPTOR,SH2D1A,SLX4,SOX2	0.266000129	1.227926052
path_1281	AKT1,AKT2,BCL10,BMPR1A,DOT1L,E2F3,FOXP1,GSK3B,HIST1H1C,INSR,KNSTRN,MDM4,PARP1,PIK3CB,RAD50,REL,RNF43,SOS1,TAP2,TP63	0.269360711	1.167792961
path_1282	ANKRD11,BCL2L1,CARM1,FLT1,FUBP1,GATA2,GNA11,IL10,INSRR,MED12,NCOR1,NF1,NTRK1,PIK3R3,PPM1D,PRDM1,SH2D1A,TP53BP1,TSC1,TSHR	0.499851547	1.128567035
path_1283	ALOX12B,ASXL1,ATR,CREBBP,CTCF,EIF4A2,ERBB2,FGF4,HIST1H3A,HIST1H3E,IDH1,KDM5A,MSH2,MTOR,NKX3-1,PIM1,PNRC1,PTPRD,SDHAF2,SDHB	0.216762136	1.221776694
path_1284	AKT1,ATR,ATRX,BCL2,CALR,CHEK1,CREBBP,CXCR4,EGFR,HGF,HIST1H3A,MDC1,NCOA3,PIK3R1,PPP2R1A,RAD51C,RBM10,SDHC,SMAD3,SMAD4	0.670122084	1.065892883
path_1285	APC,ARID5B,ASXL1,ATR,AURKA,BCL2,CENPA,DIS3,FLT3,HIST1H3B,IDH1,KIT,MAP3K13,MSH3,MUTYH,PMAIP1,PMS2,PTEN,SF3B1,SRC	0.470957131	1.122411608
path_1286	ALK,ARID5B,BCL2L1,CDK4,EGFR,ERG,ERRFI1,HIST1H3J,INPP4B,KNSTRN,MAP3K13,MUTYH,MYC,NF2,NOTCH3,PHOX2B,POLE,RAD51C,RAD54L,SDHC	0.700104401	1.071416994
path_1287	AR,ARID5B,BRCA2,CARD11,FH,HIST1H3B,IL7R,MLL2,NF1,PALB2,PMS2,PPP6C,RAD51,RAD51C,RB1,RHEB,SF3B1,SHQ1,SOX17,TP63	0.117032865	1.274503714
path_1288	ARAF,ATM,AXL,CHEK2,CTCF,CTLA4,EIF4E,EP300,EZH2,FANCA,IRF4,KIT,MEN1,NKX3-1,PIK3CA,PLK2,REL,RYBP,SUFU,TCF3	0.831390557	1.038142305
path_1289	AMER1,BBC3,CCND2,CRLF2,CSF1R,ERCC4,FBXW7,FLT3,HGF,MAP2K2,MSH2,NCOA3,NFKBIA,NOTCH1,PIK3C2G,PLK2,RFWD2,SDHC,SMAD2,TGFBR2	0.536402745	1.108867312
path_1290	ARID1B,CDH1,CHEK1,HNF1A,IL10,INSRR,LATS1,MYC,MYCL1,MYOD1,NF2,NFKBIA,RASA1,REL,RET,SHQ1,STAT5B,TGFBR1,TP53,TSC2	0.091409346	1.281878131
path_1291	BRIP1,CCND1,DAXX,EIF1AX,FGF19,GNAQ,IL10,KMT2A,KRAS,NEGR1,NUF2,PIK3C2G,PIK3C3,PIK3R1,PNRC1,PTPN11,RARA,SDHB,SMO,TBX3	0.067135332	1.320634871
path_1292	BRCA2,CTCF,FOXO1,GATA2,GRIN2A,HIST1H3A,HIST1H3J,KDM5C,KMT2A,KMT2D,LATS2,MDC1,NF1,NOTCH4,NPM1,PGR,PIK3CD,RASA1,RB1,RECQL4	0.128033522	1.253356459
path_1293	AKT2,BARD1,CALR,CDH1,CENPA,ERG,GNAS,GPS2,HIST1H3D,KMT2A,NOTCH2,NUP93,PIK3R1,PMAIP1,PPM1D,RAD51D,ROS1,SHQ1,SMAD2,SMARCB1	0.205820202	1.206155296
path_1294	AKT1,ARID1A,BCL10,BLM,CDH1,DIS3,EPHA5,GNAQ,HLA-A,IRF4,KDM5C,KEAP1,MITF,MSH2,PAX5,PIK3CA,PMAIP1,POLE,ROS1,SDHC	0.205950574	1.197671377

path_1295	ALOX12B,AXIN2,BCL2L11,BRCA1,BTK,CCNE1,CYSLTR2,ESR1,FGFR1,GATA2,HIST1H3B,IRS2,MAP2K4,MEN1,NUP93,PAX5,PTCH1,RAD51B,STAT5B,TRAF7	0.903956092	1.01732145
path_1296	ASXL1,BTK,CHEK1,EIF1AX,EPHA5,FANCC,HIST1H3C,HIST1H3G,HIST1H3I,HNF1A,LATS1,MYCL1,NOTCH4,PARK2,POLE,RAD51D,SDHC,SETD2,SMAD2,STAG2	0.456616283	1.125289033
path_1297	CARD11,DAXX,DOT1L,FGF4,FLT1,GATA3,INSR,IRF4,MLL,NFE2L2,NKX2-1,NOTCH4,PIK3R1,PMS1,PRDM1,PTPN11,SETD2,SMAD2,SOX17,STK11	0.945501525	1.010050205
path_1298	ACVR1,AKT3,ARAF,AXIN1,CDK4,EPHA5,FGFR3,FLT1,GATA3,HIST3H3,INHBA,INPP4B,IRS2,MSH6,NEGR1,PMS2,RAD54L,SETD2,SF3B1,SH2D1A	0.057112055	1.394929831
path_1299	AMER1,ASXL1,CCNE1,DNMT3B,EIF1AX,ERCC5,FANCA,FGF4,HIST1H1C,INPP4B,KLF4,MAP3K13,PAK1,PMS1,PRDM1,RAD51,RBM10,SMAD2,SMAD4,SOCS1	0.126646018	1.303434983
path_1300	BARD1,BMPR1A,BRAF,CXCR4,DROSHA,EGFR,FBXW7,GATA1,GSK3B,IRF4,MDC1,MDM4,MGA,MSH6,NPM1,PAX5,PNRC1,PPP6C,RAD51D,TCF3	0.674742644	1.071983408
path_1301	CD276,CDKN2A,FLT1,IL7R,MEF2B,MEN1,MYC,MYCL1,PHOX2B,PIK3C2G,PMAIP1,PMS2,POLD1,RAD51B,SMARCB1,SOX17,STAG2,SUFU,TCF3,TGFBR1	0.07135384	1.288750702
path_1302	AKT1,AXIN2,AXL,CCND2,CD79B,CTLA4,DNAJB1,EPHB1,GNAS,ICOSLG,IGF2,MDM2,NEGR1,PTCH1,RASA1,RICTOR,SDHA,SRSF2,STK11,TMPRSS2	0.388882843	1.163401899
path_1303	ARAF,BCL2,BRAF,BRIP1,CDK6,CDKN2C,DIS3,DNMT3B,ERCC4,EZH2,FGFR2,FLT4,KNSTRN,MAP3K1,NRAS,RAF1,RPS6KA4,SPOP,STK40,SUFU	0.722634992	0.950805235
path_1304	BBC3,BRAF,CARM1,CBL,CDK8,CYSLTR2,EIF4E,FGF19,FOXL2,GNAQ,HIST1H3D,HIST3H3,IL7R,INHA,JAK1,MYCN,NRAS,PARP1,RECQL4,SLX4	0.541191987	1.094698538
path_1305	CDH1,CDK12,CDKN2C,CTNNB1,EED,GATA2,IL10,INPP4A,NKX3-1,PMS1,PTPN11,RAF1,RHOA,ROS1,SMAD2,SMARCB1,SOX2,TCF3,TET1,TMPRSS2	0.189848373	1.212278901
path_1306	AKT2,ANKRD11,BCL2L11,CDKN1A,CREBBP,EIF1AX,FYN,GATA1,GNA11,GNAQ,IFNGR1,IL10,INHA,LATS1,LATS2,MAP2K4,MEN1,MYC,SDHB,SH2B3	0.785535674	0.961612832
path_1307	APC,ARID5B,AURKA,BCL2L1,BCL2L11,CD276,CD79B,CENPA,EZH2,GATA3,GLI1,GSK3B,HIST1H3J,HIST3H3,MAP2K4,MCL1,MYOD1,PIK3C2G,PIK3R2,SDHB	0.749951372	1.04601597
path_1308	ACVR1,ALK,ARID2,AXIN2,DNMT3B,EED,EIF4E,ERG,FANCA,FBXW7,IFNGR1,IKBKE,LATS2,MAP2K4,MDC1,PIK3CB,PMS1,RHOA,TERT,TP53	0.435263422	1.116884044

path_1309	BRCA2,CUL3,CXCR4,DNMT3B,EGFR,ESR1,FGF3,FYN,HGF,HIST1H1C,IRF4,NSD1,PIK3CG,PPM1D,RAC2,REL,RPS6KB2,RTEL1,SMAD2,STK40	0.49034888	1.116721127
path_1310	BCL6,CDKN1B,CENPA,EPHA3,FOXP1,GLI1,INSR,IRS2,JAK1,KMT2D,NKX2-1,NKX3-1,PAX5,PIK3CD,PRDM1,RPTOR,SRC,SUFU,TP53BP1,TP63	0.390440328	0.848913788
path_1311	DDR2,EP300,EPHA5,FANCC,FGFR2,FOXA1,HIST1H3A,HIST1H3C,IFNGR1,IL7R,MLL3,MST1,PAK7,PIK3CG,PMS1,PNRC1,RBM10,RHEB,STAT5B,TBX3	0.211981088	1.200615531
path_1312	APC,BRCA1,CALR,CDK4,CUL3,EPHB1,ERCC5,ERG,EZH2,FH,MITF,MLL2,MRE11A,NOTCH4,PIK3CD,PIK3R1,RAD21,REL,RUNX1,SDHAF2	0.723576675	0.946156786
path_1313	AR,CBL,CDKN1B,CTCF,DROSHA,FOXO1,FYN,GATA1,INSRR,JAK2,MSH6,MST1,MYCN,NPM1,PIK3CA,PMS1,RAD51B,REL,SUFU,SYK	0.99730447	1.000504459
path_1314	ABL1,BCL10,CCND1,CREBBP,FGF19,FGF4,FOXL2,GNA11,GRIN2A,HIST1H3B,IKZF1,INPP4A,MLL2,NOTCH4,PDGFRB,PIK3R2,PTPRS,RAD51D,RAD54L,SMO	0.878403111	1.025326885
path_1315	AKT3,ARID5B,CCND1,CTNNB1,DIS3,FGFR4,GLI1,HIST1H3D,HIST3H3,KLF4,MPL,MRE11A,PARK2,PGR,PIK3R1,PMS1,POLD1,RICTOR,SOX9,XPO1	0.952527702	0.991444963
path_1316	ATR,BARD1,BMPR1A,CD79B,CDC42,HIST1H3C,HIST1H3J,INPP4B,IRS2,MAP2K4,MLL2,NSD1,PAK7,PPM1D,PTEN,PTPN11,PTPRT,RB1,SOX17,TAP2	0.283007495	1.175802152
path_1317	ACVR1,ARID2,BAP1,BRD4,CCND1,CEBPA,CENPA,CHEK1,CTCF,E2F3,EPHA7,FGF3,GLI1,GNAQ,MAP2K2,MAX,PDCD1,SH2D1A,SMO,TET2	0.207960772	1.249858363
path_1318	AKT3,BCL2,CDK6,CYSLTR2,EPHB1,FAM46C,FGF3,FGFR1,FGFR3,GRIN2A,HIST1H3A,HIST1H3H,KLF4,MAP3K1,PRDM1,RAC2,RAD51,RARA,RHEB,SPEN	0.069010753	1.298905801
path_1319	BCL2L11,BRIP1,ERRFI1,FLT4,HIST1H3D,INHBA,MAP2K1,MDM2,NF2,NFKBIA,NOTCH4,NTRK1,RECQL4,RICTOR,SDHC,SH2D1A,SRC,SRSF2,STAT5B,TNFAIP3	0.056082474	1.400838736
path_1320	B2M,BARD1,CCND1,CD276,CRLF2,EPHA5,GATA1,HIST1H3G,HIST3H3,PAK7,PARP1,PDCD1,PIK3R1,POLD1,PREX2,PTPRD,STK40,SUFU,TBX3,TOP1	0.33268154	1.17426553
path_1321	ATRX,BCOR,BRD4,CALR,CDK8,CHEK2,EED,FUBP1,GATA2,INSR,MEN1,MLL3,NOTCH2,NUP93,PBRM1,RAD51,RPS6KB2,SF3B1,SH2D1A,STK11	0.067592263	1.316102766
path_1322	BRD4,CDH1,FAT1,FOXA1,ICOSLG,IKBKE,KDM6A,MED12,PDGFRA,PDGFRB,RB1,RHEB,RHOA,RNF43,SH2B3,SHQ1,SMAD4,SPOP,TGFBR1,TSC1	0.218492372	1.243614538

path_1323	ATR,AXIN1,CTNNB1,EIF4A2,EP300,ERBB2,FANCA,FGFR4,HIST1H3C,HNF1A,KMT2D,MAP2K1,MAP3K13,NCOA3,NKX3-1,NOTCH2,PIK3CB,RICTOR,SMAD2,SOX2	0.605674344	1.075618625
path_1324	DNMT1,ERCC2,FAT1,HIST1H1C,HIST3H3,MEN1,NCOA3,NOTCH2,NPM1,PBRM1,PMAIP1,PPP2R1A,PTPN11,RET,RICTOR,RNF43,SDHAF2,SDHB,STAG2,TGFBR2	0.346100069	1.179721794
path_1325	BARD1,BRCA2,CDK12,CEBPA,EGFR,ELF3,INHA,IRF4,KDM5A,KNSTRN,MDM2,MEN1,MITF,NFE2L2,NOTCH3,PMS2,PTCH1,RAD54L,SUFU,TGFBR1	0.774959003	0.952695065
path_1326	BTK,CD79B,CDH1,CDK6,CDKN1A,CSF3R,FGF19,GATA1,IGF2,IKBKE,IKZF1,INHA,KDM5A,MRE11A,PNRC1,PPP2R1A,PTPRD,RAF1,SDHA,STAT3	0.158810267	1.223559915
path_1327	AKT2,ALOX12B,APC,ARID1B,BRCA2,CREBBP,FANCA,HIST1H3I,IGF2,KDM5A,PHOX2B,PTPRS,RAD51,RAD54L,RTEL1,RYBP,SETD2,SH2B3,SYK,TGFBR2	0.265308269	1.230919712
path_1328	BRCA1,BRCA2,BRD4,CDKN1A,CEBPA,CHEK1,EED,ERBB2,ERBB3,ERCC4,FAM46C,IRS1,KLF4,MDM2,MLL3,MSH2,MST1,NSD1,PMAIP1,RECQL4	0.118363298	1.310733446
path_1329	CBL,CIC,CSF3R,FH,GATA2,GSK3B,IDH1,IGF1R,NF2,NSD1,NUP93,PAK7,PGR,PIK3CB,PIK3R2,RAD21,SH2D1A,SMO,SRC,U2AF1	0.128518791	1.311979648
path_1330	AKT1,AKT2,ALK,ATR,DNMT1,DNMT3B,EIF4A2,FAT1,FGFR4,H3F3C,HNF1A,KRAS,MST1,POLE,RAF1,RARA,SOCS1,SYK,TGFBR2,TOP1	0.278711677	1.169878944
path_1331	ALOX12B,APC,CDK12,CSF3R,CUL3,DOT1L,EED,EIF4A2,FGFR1,FGFR2,FLT3,FOXL2,GPS2,GRIN2A,HIST1H3B,IDH1,MCL1,MLL3,NOTCH2,RAD21	0.679474216	1.074675186
path_1332	BCL2,BMPR1A,CCND3,CDK4,CDKN2C,ERBB3,FLT1,FUBP1,GATA2,GNAS,IKZF1,IRF4,IRS1,MRE11A,PDGFRB,RB1,SH2B3,SOX17,U2AF1,WT1	0.879067828	0.978739241
path_1333	CALR,CARM1,CD79B,CDC73,CDK12,CDKN2A,CTCF,ETV6,FANCC,FYN,GNA11,GSK3B,HOXB13,IKZF1,INHBA,IRS1,LATS1,MSH2,PIK3CG,RICTOR	0.100800362	1.326184196
path_1334	BRCA2,CBL,CCNE1,CDK12,DNMT1,EIF4E,EPHB1,GRIN2A,HIST1H3G,HLA-A,IDH1,INPP4B,KDM5A,MET,MITF,NCOA3,PDGFRB,PIK3CB,PLCG2,RASA1	0.101410017	1.30824982
path_1335	ACVR1,BRAF,CTLA4,FANCC,FAT1,HGF,HIST1H3G,IFNGR1,INSRR,MAP3K13,MITF,MLL2,MYCN,NEGR1,PIK3R3,RAC2,RARA,RPS6KB2,SH2B3,TP53BP1	0.302834757	1.186259896
path_1336	AR,ARID5B,ASXL1,CBL,CDC73,CIC,CTLA4,CTNNB1,FOXA1,INPP4B,KEAP1,MAP3K1,MED12,NOTCH2,NTRK1,PIK3R1,PPM1D,PTPRD,RAD50,SOX9	0.132054905	1.243301144

path_1337	ALOX12B,ATR,AURKA,BCL2L1,CCND1,ERG,FBXW7,HIST1H3G,HIST1H3H,HIST3H3,HNF1A,MAX,MDC1,PMAIP1,POLE,PREX2,RAD21,REL,SHQ1,TRAF7	0.168465053	1.216758376
path_1338	ARID5B,BCL2L11,CHEK1,DNMT3B,EP300,ERBB2,ERCC5,ESR1,FAT1,INHA,INPP4A,NPM1,PIK3R1,PIM1,RAD50,SHQ1,SMARCD1,SMO,STAT5B,STK11	0.068408918	1.318692732
path_1339	BRIP1,BTK,CDKN1A,CEBPA,CRLF2,FAT1,IKZF1,MITF,MSH3,MYCL1,MYCN,NCOR1,PMS1,PREX2,PTCH1,RAD51D,RYBP,SMAD3,STAT5B,TP53	0.070124539	1.29868496
path_1340	ALOX12B,CDC73,EPHA5,FGF3,IGF1R,KMT2A,MAP2K2,MAP3K1,MLL3,PBRM1,PIK3CA,PMAIP1,PTPRD,RAF1,RASA1,SETD2,SMAD2,SMARCA4,SYK,TP53	0.224203674	1.19523674
path_1341	BCL2L1,CCND2,DAXX,EZH2,FGFR2,IKZF1,JAK1,MAX,MET,MLL2,PAK7,PIK3C2G,PIK3C3,RAD51B,RAD51C,RBM10,RHOA,SH2B3,SOS1,TET2	0.745461441	0.951913473
path_1342	B2M,DROSHA,EZH2,FAM46C,FLT1,FOXA1,GATA1,HIST1H1C,HIST3H3,IKBKE,MEF2B,NKX2-1,PARP1,PDGFRB,PMS1,PTCH1,RASA1,RUNX1,SRSF2,TAP2	0.533049662	1.093459613
path_1343	ATM,AXIN1,CENPA,CXCR4,ERBB2,ERCC4,FGF19,HGF,JAK3,KEAP1,MDM4,MLL2,NSD1,PARP1,PIK3R3,PMS2,RARA,SMAD2,SMAD4,SOX2	0.468885322	1.113731848
path_1344	AR,BCL2L11,CALR,CARD11,CDK8,DICER1,FOXA1,FUBP1,IGF1R,INSR,KRAS,MDM2,MYC,MYCN,PMS1,RFWD2,RTEL1,SETD2,SH2B3,SRC	0.051395884	1.353406396
path_1345	BCL2,CD79B,DICER1,DROSHA,ERCC5,ERRFI1,FGF19,FGF4,IKZF1,JAK2,KRAS,MAX,PGR,PIK3R2,SMARCB1,SMO,SOX17,SOX9,SUFU,TERT	0.170525304	1.242211135
path_1346	ARID1A,ASXL2,AXIN1,BBC3,BMPR1A,CARD11,CSF1R,CXCR4,FLT1,GNA11,HIST1H3G,JAK3,KDR,MAP2K4,MAX,MUTYH,PIK3CB,RAD51C,RBM10,TET2	0.155412995	1.245792147
path_1347	AKT2,AMER1,ATM,CCNE1,CD79B,CENPA,FOXL2,FYN,HIST1H3D,IL7R,INPP4B,KIT,NCOA3,NKX3-1,NUP93,PIK3R1,PREX2,RBM10,SF3B1,SOCS1	0.580283666	1.089526287
path_1348	AURKA,BCL2L1,CARD11,CBL,CDK8,DICER1,DNMT3B,FGF4,FLT1,GSK3B,HGF,INSRR,MYCN,NF2,NTRK1,PNRC1,POLD1,RICTOR,SHQ1,TRAF2	0.200770128	1.256603841
path_1349	ACVR1,AKT3,ALK,BARD1,CUL3,DNMT1,E2F3,FAM175A,FANCA,FUBP1,IRS2,JAK1,KDM5C,MAP2K4,PIM1,PTPRD,REL,SDHC,STAG2,TBX3	0.449365804	1.137119266
path_1350	ABL1,AKT1,AKT2,ASXL1,BBC3,BCL6,CCND2,CHEK2,CUL3,DICER1,DIS3,DOT1L,ERBB2,FBXW7,HIST1H3I,ICOSLG,MAP2K4,MTOR,SMARCB1,SRC	0.893351373	0.98126721

path_1351	ARID1A,CREBBP,CRLF2,CTLA4,DNMT1,EGFR,ETV6,FAT1,HIST1H1C,HIST1H3D,KNSTRN,NKX2-1,NTRK2,PARP1,PIK3C2G,RFWD2,SDHB,SMAD2,STAT3,TAP2	0.490024096	1.111381413
path_1352	AMER1,BARD1,CDK4,DOT1L,EIF1AX,EPHA5,ERBB4,FAM175A,FGFR3,INHBA,JAK1,MDM2,NF1,NFE2L2,NOTCH1,NOTCH4,PHOX2B,PIK3CG,PPM1D,SMAD2	0.500703043	1.101336115
path_1353	ARID2,CD276,CDC42,CHEK2,DAXX,EGFR,EIF1AX,EIF4A2,FAM46C,GNAQ,HIST1H3J,IFNGR1,INPP4B,MSH3,MYOD1,NF1,NTRK2,PBRM1,PIK3R3,ROS1	0.478335809	1.122079381
path_1354	BCL10,CDK8,CDKN1A,CIC,ERRFI1,FBXW7,FOXA1,KDR,MAP2K1,MAP2K4,MEN1,MET,MUTYH,NKX2-1,RET,SLX4,SMARCB1,SOX9,TNFAIP3,TOP1	0.201913701	1.197012458
path_1355	AKT2,ARID5B,BCL2,CCNE1,CDC73,CDH1,CRLF2,CTCF,CTLA4,DDR2,FOXP1,JAK3,LATS1,MGA,NF1,NKX2-1,POLE,RICTOR,RPS6KA4,SYK	0.067572129	1.362405647
path_1356	BAP1,E2F3,ERCC4,GATA3,HNF1A,ICOSLG,KMT2A,MEN1,MPL,MRE11A,MYCN,NTRK1,PIK3C3,PIK3CD,RAD51B,RECQL4,RYBP,SDHA,SF3B1,SLX4	0.050183237	1.32467582
path_1357	ACVR1,BRD4,CDC42,CDC73,ERCC2,FUBP1,GSK3B,KDM5A,MAX,MST1,NOTCH2,PGR,PIK3CD,PTEN,RAD51C,RUNX1,SMARCB1,STK40,TGFBR1,TSHR	0.069056251	1.29250975
path_1358	ALK,ARID1B,CARM1,CDH1,CREBBP,DOT1L,ERBB4,FGFR2,HIST1H3I,HLA-A,MED12,POLD1,RBM10,RPS6KB2,SMAD4,SYK,TAP1,TCF3,TGFBR2,TMPRSS2	0.36976566	1.145662565
path_1359	AKT2,ANKRD11,ATRX,BCOR,DNMT3A,EP300,FOXP1,HIST1H3C,HIST1H3E,HIST1H3I,INSR,LATS2,MCL1,PALB2,PBRM1,PIK3CB,PTPRD,RAD54L,SDHC,TET2	0.345541824	1.158903675
path_1360	ACVR1,ATR,AXL,BBC3,CDKN1B,CHEK2,EPHA5,ETV6,HIST1H3A,HIST1H3J,MAP2K4,MPL,NFKBIA,NTRK2,PIK3R3,PMS2,PTPN11,RHEB,SF3B1,SMAD4	0.807153564	0.966094283
path_1361	BCL2L1,CSF1R,CXCR4,DIS3,DNMT1,EED,EPHA5,EPHB1,ERCC2,ERG,FAT1,INPP4A,KDM5A,MYC,NBN,PIK3C3,PIK3CD,PTPRD,REL,RHEB	0.063522587	1.327055243
path_1362	AKT2,CDK4,CRLF2,ELF3,GATA3,MED12,MGA,MSH3,PIK3R2,RAD51D,RFWD2,RHEB,SETD2,SRSF2,SUFU,TMPRSS2,TOP1,TRAF7,TSC1,U2AF1	0.053047982	1.313638734
path_1363	ATR,AXIN1,CTCF,FGFR4,FH,IKBKE,IRS2,KDM6A,KMT2D,MALT1,MEN1,MSH2,PARK2,PMS2,POLD1,RNF43,RPTOR,SHQ1,SMARCA4,SMO	0.331908685	1.196655288
path_1364	ACVR1,ALOX12B,APC,BBC3,BCL2L1,BCL2L11,CDKN1A,CDKN2A,DNMT1,EPHB1,FGF19,FLT4,ICOSLG,MDM2,MLL2,MLL3,RET,SDHA,SMO,SOS1	0.341133328	1.168400823

path_1365	CARD11,CDC73,CTCF,CUL3,DOT1L,ERCC2,FOXL2,GATA2,IDH1,IGF2,IL10,JAK2,PHOX2B,PMS2,RHOA,ROS1,RPS6KA4,SH2D1A,SLX4,SMARCA4	0.1040814	1.328769543
path_1366	BLM,CSF1R,CYSLTR2,HIST1H3H,MCL1,MDC1,MDM2,MEF2B,MYCN,NFE2L2,NOTCH1,NTRK2,PIK3CB,PMAIP1,PPP2R1A,PTCH1,RAD50,RHOA,SOS1,STAG2	0.132043095	1.236876093
path_1367	ATM,B2M,BRCA1,CCND3,CDK4,CUL3,DNMT1,EP300,ESR1,FANCA,JAK1,MYCN,NKX3-1,NTRK2,PARP1,PAX5,PRDM1,RAD51C,SOX2,STK40	0.840049153	0.971895323
path_1368	ARID1A,BCL2L11,CCND3,CCNE1,CDKN2C,DNMT3A,EGFR,ERBB3,ERRFI1,ESR1,HIST1H3D,HLA-A,LATS1,NFE2L2,NUP93,PBRM1,PGR,RHEB,SMARCD1,STK11	0.480768459	1.106026284
path_1369	ARID2,ASXL2,B2M,BRCA1,CEBPA,DNMT1,GSK3B,HIST1H3A,HLA-A,KIT,MAP3K13,MEF2B,MLL,MSH2,NTRK2,PPP6C,PTPRD,RAD51D,ROS1,TCF3	0.778107633	1.047887
path_1370	B2M,BCL2L11,CDK6,CREBBP,CSF1R,CYSLTR2,DOT1L,EP300,ERRFI1,FANCC,FAT1,IKBKE,IKZF1,NPM1,NUP93,PMS1,PTPN11,RAC2,RYBP,SETD2	0.60885441	1.075000743
path_1371	ANKRD11,CDKN1A,DOT1L,FANCA,FAT1,FBXW7,FGFR4,IL7R,INSRR,IRF4,JAK3,KDM5A,KRAS,MST1,MYC,PAK7,PIK3CG,RARA,RICTOR,SPOP	0.235630899	1.183156175
path_1372	ABL1,ALK,ATM,AXIN1,CD79B,CEBPA,CENPA,CSF1R,DIS3,FGFR1,ICOSLG,INHA,MYC,NKX2-1,NUP93,PIK3R1,PPP6C,RAF1,TAP2,TET1	0.411185379	0.890570916
path_1373	BCL2L1,CD79B,CSF1R,CUL3,EIF1AX,FOXL2,HIST1H3H,IKBKE,INPP4B,IRS1,MDM4,MYC,NSD1,PBRM1,PTPN11,RAD50,SDHA,SMO,SPOP,TRAF2	0.197588801	1.198426409
path_1374	ALOX12B,ARID1A,CALR,CIC,DDR2,EIF1AX,FGFR4,FH,HIST1H3B,KEAP1,MGA,NTRK1,PIK3C3,PIK3CD,RECQL4,SDHB,SMARCA4,STAT5B,TERT,TRAF2	0.423957534	1.123272408
path_1375	AKT2,AMER1,BRIP1,CD79B,CUL3,GATA2,IGF1R,IKZF1,INSRR,JAK1,NOTCH4,NUF2,PARK2,PDGFRA,PIK3R2,RASA1,RYBP,SMAD4,STAT5B,TOP1	0.123448735	1.293081225
path_1376	ASXL1,B2M,BLM,CARM1,CENPA,ERBB2,FOXP1,FYN,GLI1,HOXB13,KDM5C,KDM6A,KMT2A,MEN1,MUTYH,PIK3C2G,PIK3CA,PIK3R2,RAD50,SF3B1	0.288979208	1.18911734
path_1377	ARID1B,ATRX,BRCA1,EPHA7,FANCA,HIST1H3G,INHBA,IRF4,KNSTRN,MAP3K13,MEN1,NFE2L2,NOTCH2,PAX5,PMS1,PPP6C,RAD54L,ROS1,RTEL1,SMAD4	0.069061508	1.350220177
path_1378	ABL1,APC,ASXL1,ATR,CSF3R,CYSLTR2,FLT4,FYN,INPP4B,KNSTRN,MGA,NTRK2,NTRK3,PDCD1,PIM1,PLK2,RAF1,TGFBR2,TOP1,TP63	0.067966233	1.338592622

path_1379	ALOX12B,ARAF,ASXL2,AXIN1,CHEK2,ETV1,FGFR4,HIST1H3D,IL7R,JAK2,KDM5A,MAP2K1,MGA,PAX5,PHOX2B,POLD1,RAD51D,SOS1,STAG2,STK11	0.129412765	1.280831719
path_1380	CBL,CTCF,EIF1AX,EZH2,FGFR3,FGFR4,GATA2,HGF,HIST1H3I,IKBKE,INSRR,MLL3,NF2,NUP93,PARP1,PBRM1,POLD1,PTPRT,RASA1,RPS6KA4	0.21884596	1.227694746
path_1381	ALOX12B,BAP1,CDC73,CDKN2C,DIS3,EPHA5,FGF3,GSK3B,JAK1,MLL2,MPL,NOTCH3,NRAS,PBRM1,PPP6C,RAC2,RUNX1,SMARCB1,SOX2,U2AF1	0.224770055	1.226420879
path_1382	ALOX12B,ARID5B,ATM,BCL2L11,CDK4,CSF3R,EP300,EPHB1,ERRFI1,FAT1,FOXO1,HIST1H3H,MST1,NFKBIA,PDGFRB,PIK3C2G,RAD21,RNF43,SOX9,TP53BP1	0.990876688	0.998059316
path_1383	AKT1,ARAF,BRCA1,EIF4A2,ELF3,ERCC4,ESR1,FGFR3,HIST1H1C,HIST1H3D,IL10,KEAP1,MAP2K2,MGA,PNRC1,RAD51,RASA1,RBM10,RPS6KB2,SMARCD1	0.290391154	1.179236645
path_1384	ALOX12B,BCL2L1,BMPR1A,CDC73,CSF3R,CUL3,EED,EPHA3,ERCC2,GNA11,HIST1H3A,INSR,KDM5C,MYCL1,NKX2-1,NTRK2,PDGFRB,PIM1,RAD54L,RBM10	0.537754666	1.112283766
path_1385	AXIN1,CBL,CREBBP,ERCC2,GRIN2A,INHBA,KLF4,KRAS,MRE11A,NCOR1,NUP93,PAX5,PDCD1,PIK3CG,PPP6C,RAD54L,RPTOR,SDHA,SHQ1,SMAD2	0.702497861	1.05596967
path_1386	ACVR1,ASXL2,AXIN2,BRD4,CRLF2,CSF1R,CUL3,EED,FGFR3,GATA1,HIST1H3G,IL10,IRS1,LATS2,NF2,NOTCH3,NUP93,RARA,RPS6KA4,SHQ1	0.063335095	1.3003602
path_1387	BCL2,BCL2L11,BCOR,CD274,CDK4,CDKN2C,DICER1,EPHA5,ERCC4,GATA2,GATA3,JUN,KDM6A,KEAP1,NOTCH4,NTRK3,PBRM1,PIK3C2G,SMAD4,SMARCA4	0.183039283	1.206462883
path_1388	AKT1,BCL2L1,BRCA1,CALR,CREBBP,EIF1AX,FANCA,H3F3C,HIST1H3C,IFNGR1,MLL2,MSH6,NKX3-1,RAD51B,RHOA,SDHC,SMAD3,STAG2,STK11,TP63	0.114918789	1.278977204
path_1389	ALOX12B,ARID1B,AXIN1,CXCR4,E2F3,ERBB3,ERBB4,FGFR4,FLT4,HIST1H3I,IKBKE,KNSTRN,MAP2K2,MCL1,NPM1,PIM1,RHEB,RPS6KB2,SMAD3,SYK	0.056950351	1.323032911
path_1390	AXIN1,BCL10,CTLA4,EZH2,FUBP1,GATA1,GLI1,INHBA,MGA,MYC,NF2,NFE2L2,NOTCH4,NPM1,NUF2,PAK1,REL,SDHC,SOCS1,SOS1	0.1437671	1.28378098
path_1391	AXIN2,BCL10,BRAF,CDKN1B,CXCR4,DROSHA,FGF19,IDH1,IRS1,JUN,MAP2K1,MST1,NOTCH3,PAK7,PIM1,PPP6C,RAD54L,RECQL4,SLX4,SOS1	0.070134929	1.293253162
path_1392	ABL1,ARID1A,AXL,EPHA3,ERBB2,ERBB4,FAM46C,FGF4,FLT4,FOXL2,HGF,KDR,NOTCH4,PIK3C3,PIK3CB,POLD1,RASA1,SDHA,SDHC,STK40	0.076320623	1.29964516

path_1393	ARID5B,DICER1,DNMT1,EED,EZH2,FGFR1,FGFR2,GPS2,KMT2D,MITF,NOTCH4,PRDM1,RAD51,SMARCA4,SMARCD1,SMO,STAT3,TAP2,TOP1,TSC2	0.201685339	1.289362464
path_1394	ANKRD11,BCOR,CSF1R,E2F3,ERBB2,FANCA,FGFR1,GATA2,H3F3C,HNF1A,INHA,MDM4,MSH2,MSH3,NKX3-1,PIK3R2,RPTOR,SMAD2,STK11,STK40	0.99876353	1.000251953
path_1395	BRIP1,CCND2,CENPA,DOT1L,FOXP1,GATA2,GPS2,INHBA,JAK3,KRAS,MAP3K13,MED12,PDGFRB,PIK3R3,PPM1D,RARA,SH2B3,SOX2,TAP1,TEK	0.132182548	1.248942202
path_1396	ACVR1,AKT2,ALOX12B,CD79B,DROSHA,EGFR,EPHB1,KDR,KLF4,MAP3K1,MDC1,MED12,MSH6,NF1,PGR,PIK3R1,SOX17,TCF7L2,TNFAIP3,TOP1	0.183339032	1.244574415
path_1397	CBL,DNMT3B,ERBB2,H3F3C,HIST1H3B,HIST1H3H,JAK2,KLF4,LATS2,MALT1,MDM4,MLL2,MUTYH,PTEN,SF3B1,SOCS1,TAP1,TCF7L2,TERT,TET1	0.062294996	1.360427488
path_1398	ATM,B2M,CDK8,CHEK2,ETV6,FUBP1,GATA2,HOXB13,MET,MSH2,MSH3,PIK3CB,PMAIP1,PPP6C,PTPN11,RAD21,SH2B3,SPOP,TSC2,WT1	0.400170832	0.887907224
path_1399	BAP1,BMPR1A,CARD11,CCND2,CDH1,DAXX,DNMT1,EIF4E,JAK1,KDM5A,MGA,NRAS,NTRK1,PIK3CB,PLCG2,PLK2,PPP2R1A,SOX17,TGFBR2,TSHR	0.084833513	1.373992667
path_1400	AXIN1,BCL6,CDH1,CDK4,DROSHA,GRIN2A,HIST1H3C,JAK1,KIT,MEN1,MST1R,NFKBIA,NKX2-1,PBRM1,PDGFRB,PIK3CB,PIK3R1,RAD51C,SDHAF2,TAP2	0.852685744	0.974102186
path_1401	ALK,AXL,BCL6,CARD11,CBL,CDC73,CENPA,CSF1R,FBXW7,KDM5C,MAP2K2,MLL2,MST1,NF2,PARP1,PGR,PMS2,RAD54L,RFWD2,SMAD3	0.434002699	1.153909756
path_1402	AMER1,CRLF2,CTLA4,EPHA3,ERBB2,FOXA1,GATA2,HLA-A,ICOSLG,KMT2D,KRAS,PIK3R2,RAC2,RAD51D,RARA,RB1,SDHA,SDHAF2,SOX17,TBX3	0.351099485	1.149182149
path_1403	AMER1,ANKRD11,ARAF,CD79B,CTNNB1,ESR1,FGFR2,FGFR3,HIST1H3C,INPP4B,KRAS,MYOD1,NF2,NKX2-1,NOTCH1,PAX5,PGR,PLK2,RAD51,RB1	0.872545023	1.022995236
path_1404	ARID1B,BLM,BRIP1,CDK12,CDKN2A,CTLA4,FGF19,GATA1,GNAS,H3F3C,IFNGR1,INPP4B,MAP2K2,NKX2-1,PAK7,PDGFRA,PPP6C,RNF43,SMAD2,SMARCB1	0.433092138	1.134915935
path_1405	BCL2L1,BRD4,CARM1,CCNE1,CDK12,CTLA4,DNMT1,EPHA3,FGFR2,GNA11,HIST1H3H,MED12,MET,MST1,NCOA3,NEGR1,PAK1,RAD51C,SMARCD1,STK11	0.412013747	1.142340496
path_1406	ATM,CASP8,CDH1,CDK6,CDK8,DNAJB1,DNMT3B,EGFR,IGF1R,IL7R,IRS2,KRAS,MET,NBN,NUF2,RAD54L,RASA1,SDHAF2,SMARCB1,SMARCD1	0.539187724	0.916353851

path_1407	ARID1A,CDKN2A,DAXX,EIF4E,ERG,FANCC,GATA2,GPS2,INPP4A,JAK1,KDM5A,KEAP1,MTOR,MYCL1,PARK2,PIK3CD,RAD51D,ROS1,SMAD2,TET2	0.266138039	1.18808254
path_1408	BCL2L1,BMPR1A,DOT1L,EED,EIF4A2,ERCC5,FLT4,GNAQ,IFNGR1,KDM5A,LATS2,MDM2,MEN1,MITF,MYC,NTRK1,PPP6C,RAD21,RAD50,RAD51C	0.238733174	1.18174898
path_1409	AXL,CDC42,CUL3,DNMT3A,DROSHA,FGFR2,FGFR3,GNA11,GNAQ,HIST1H3G,IDH1,KMT2A,KRAS,NTRK3,PBRM1,PIK3CB,RAD51B,RFWD2,RPS6KA4,RPS6KB2	0.348173577	1.156531555
path_1410	ATM,EP300,ERCC4,FGF4,FGFR1,FLT1,FLT4,FOXO1,GNA11,GRIN2A,HNF1A,LATS1,MDM4,MRE11A,NCOR1,NF2,PMAIP1,PPP2R1A,PPP6C,STAG2	0.92671383	1.014879725
path_1411	BCL6,BRAF,BRCA1,CCND3,CD79B,CTCF,DNMT3A,FGF3,HLA-A,JAK3,KNSTRN,NBN,NEGR1,POLD1,RFWD2,RPS6KA4,SDHA,SRC,TERT,TRAF2	0.414470075	1.122668317
path_1412	AKT1,BLM,CENPA,CHEK2,CRLF2,FAM46C,HIST1H3G,IDH1,KNSTRN,MLL3,MSH3,MST1R,NF2,NFE2L2,PDGFRA,POLD1,POLE,SMAD3,TCF3,XPO1	0.074720526	1.383849835
path_1413	BARD1,CASP8,CCND3,CSF1R,DAXX,DICER1,EIF4E,ERBB3,FBXW7,FGFR3,FOXL2,HIST1H1C,KDM5C,KNSTRN,MEF2B,PARP1,PIK3CA,REL,RTEL1,TP63	0.649221223	1.06656622
path_1414	APC,ARID1A,CUL3,EIF4A2,FAM46C,HIST1H3A,HIST3H3,IDH1,INHBA,IRS2,JUN,MAP2K1,MAP2K4,MAX,MDM2,MSH6,NCOR1,NEGR1,NFKBIA,POLD1	0.533569436	0.899770732
path_1415	AR,ARID2,BRCA2,CD276,CDK4,DNAJB1,H3F3C,HIST1H3B,IKZF1,MAP2K4,PARP1,PDGFRA,PIM1,PLK2,PMS1,RAC2,RAD51,RECQL4,RFWD2,SOX17	0.099026005	1.3880533
path_1416	AMER1,BCL10,DNMT3A,FOXL2,GSK3B,IL10,KDM5A,MCL1,NKX3-1,NOTCH4,PMS1,PNRC1,PPP6C,PTPRD,SMARCD1,SRC,STK11,TSC2,U2AF1,WT1	0.076253147	1.294811356
path_1417	ASXL1,ATM,CDC73,CIC,CRLF2,CXCR4,DAXX,ERBB2,FAT1,HOXB13,IRS1,KEAP1,LATS2,MDC1,MYOD1,PMAIP1,SDHC,SOX2,STK40,TBX3	0.774295432	1.044214981
path_1418	ACVR1,ALOX12B,BRCA1,CARM1,CSF1R,EPHA5,FYN,HIST3H3,INPP4B,KDM5A,MAP3K1,MET,MRE11A,NEGR1,NOTCH2,NSD1,NTRK1,RAF1,RHOA,SHQ1	0.052383929	1.380679256
path_1419	ARID1B,BCL2L1,BCL6,BRD4,CCND3,CHEK1,CRLF2,DDR2,ERRFI1,KDM5C,KIT,KNSTRN,MAX,MRE11A,MYCN,NCOR1,PGR,POLE,RAC2,RHOA	0.135592187	1.364208215
path_1420	EP300,EPHA3,ERCC4,ETV1,FGFR3,FOXP1,GNA11,HIST1H3C,HIST1H3J,KDM6A,KDR,LATS1,MTOR,PNRC1,PREX2,RAD51C,SMAD3,STK11,TOP1,TSHR	0.317867531	1.172355083

path_1421	ARID5B,ATM,BCL10,BCOR,BTK,CASP8,CDK6,EIF4E,GLI1,GNAS,HIST1H3H,INPP4B,JAK1,KDR,MUTYH,PALB2,PMS1,PTCH1,RECQL4,RPS6KA4	0.361160518	1.164776695
path_1422	ACVR1,BRAF,BRCA2,CARM1,EIF4E,KMT2A,LATS2,MAP2K2,NTRK2,PARK2,PIK3C2G,PLCG2,POLE,PRDM1,RAD51,RET,RHOA,RICTOR,SH2D1A,SOS1	0.057116734	1.30831405
path_1423	ASXL1,CDK8,CTCF,GLI1,IRS1,LATS2,MAP3K1,MEN1,MITF,NEGR1,PDCD1,PMS1,PTCH1,ROS1,SDHA,SHQ1,SMARCD1,SMO,SOX2,SOX9	0.106696471	1.262818962
path_1424	BARD1,CSF1R,CUL3,DNAJB1,DNMT3B,EED,EGFR,EPHB1,FH,FOXA1,FOXL2,IKZF1,JAK1,PDGFRA,PGR,RAD51,RBM10,SDHB,SH2D1A,SRSF2	0.781112074	0.958617418
path_1425	ARID1B,CD274,CD276,CDKN1A,CDKN1B,CTNNB1,FAM175A,FLT1,GATA1,MDC1,MITF,MSH3,MTOR,NCOA3,NOTCH1,PIK3R1,PPP2R1A,SDHB,SETD2,SOCS1	0.915965576	0.982087635
path_1426	AXIN1,AXL,CDKN2C,EIF4A2,EIF4E,GATA2,INPP4A,MSH6,MUTYH,MYC,NSD1,PAX5,PDGFRB,PPP6C,RAD54L,RB1,RBM10,RHEB,SMAD2,TEK	0.264462043	1.223835136
path_1427	ALK,AMER1,BBC3,BCL2,BRIP1,CDKN2C,FGFR3,INHBA,LATS2,MAP2K2,MSH3,MYCN,NFE2L2,NRAS,PIK3CD,PLCG2,PREX2,RET,RTEL1,SH2D1A	0.311712795	1.192456093
path_1428	AKT1,AKT2,ANKRD11,AXIN2,ESR1,FUBP1,GLI1,GNA11,HNF1A,IRS2,KEAP1,MDC1,MDM4,NBN,NUF2,PAX5,PMAIP1,PPM1D,RAD51B,RYBP	0.099880303	1.277182081
path_1429	ANKRD11,BBC3,BMPR1A,BRAF,CDKN2C,EED,ERRFI1,FGF19,FGFR3,IKBKE,IL7R,IRS2,JAK3,KRAS,MAP3K1,MED12,PMS1,PPP6C,SMAD2,SPEN	0.112702226	1.297540373
path_1430	BRCA1,CDC73,CTLA4,DNMT1,EPHA7,ERG,FGFR2,GLI1,HIST1H3G,IKZF1,INSR,KIT,MET,NOTCH1,PARK2,PBRM1,PIK3CG,PNRC1,SMARCB1,STK11	0.668983574	0.940420578
path_1431	AMER1,ANKRD11,APC,AXIN2,BCOR,CASP8,DICER1,DNMT1,ESR1,EZH2,FBXW7,MDM4,MLL,MSH3,NSD1,PBRM1,PRDM1,RAD51,SF3B1,TGFBR1	0.079721161	1.344765953
path_1432	AURKA,BLM,BRCA1,CDK6,CUL3,CXCR4,E2F3,EIF1AX,FH,FOXP1,GATA1,GRIN2A,HIST1H3G,HIST1H3H,NOTCH3,PALB2,PDGFRB,RTEL1,SDHAF2,SPEN	0.382631963	1.131291147
path_1433	BARD1,BRD4,CBL,DOT1L,ELF3,EPHA5,ETV1,FBXW7,INPP4B,KIT,MAP2K1,MAP3K13,MST1R,NOTCH4,PGR,PMS2,RAD54L,RAF1,RHEB,TAP2	0.241388747	1.20009335
path_1434	BRIP1,CCND1,EPHA7,ERG,FANCA,FGFR2,FH,HIST1H3C,JAK1,KDM5A,KMT2A,MAP3K13,MRE11A,NF1,PGR,PPM1D,PRDM1,SDHB,U2AF1,YES1	0.127099846	1.320891317

path_1435	AKT3,AR,ATM,ATR,CDK8,CTCF,ETV1,FOXL2,HIST1H3B,HIST3H3,HOXB13,IGF2,KDR,NFKBIA,NOTCH2,NTRK2,PLCG2,TEK,TP53,XPO1	0.444046531	1.117221163
path_1436	APC,AURKA,B2M,BCL2,BCL2L1,CALR,CARD11,CDC42,CDKN2A,CENPA,CSF3R,IDH1,MAP3K13,MLL2,PAK1,PHOX2B,PIK3R3,RAD54L,TCF7L2,TP53BP1	0.484714672	1.138126612
path_1437	CDH1,CDK8,CHEK2,EIF4A2,ESR1,FGF3,GRIN2A,H3F3C,HIST1H3A,INPP4B,INSR,KIT,MYOD1,NOTCH2,RB1,RICTOR,RPTOR,SF3B1,SOX17,TMPRSS2	0.262641375	1.200912437
path_1438	ACVR1,AKT1,ALOX12B,CCND2,CDH1,GATA2,GPS2,HIST1H3C,HIST1H3J,ICOSLG,INHA,LATS1,PAK7,PARP1,PIK3CB,PIK3CG,PIK3R1,POLD1,PTPN11,RAD54L	0.962298544	1.006689582
path_1439	ATR,CALR,CDH1,CDK6,DICER1,ERBB2,ERG,HIST1H3H,INPP4A,INPP4B,JAK3,MLL,MRE11A,NEGR1,NTRK1,PARK2,PDGFRB,PIK3R2,PIK3R3,PTPN11	0.254380404	1.176314475
path_1440	AMER1,ATRX,CDK4,FGF19,GPS2,IL10,IRF4,IRS2,MLL,MLL2,MYCL1,NOTCH2,NSD1,NTRK2,PPP6C,RAD51D,RYBP,TCF3,TERT,TNFAIP3	0.130189592	1.313346052
path_1441	AKT3,BRCA1,CDK12,CIC,DNMT1,DNMT3A,HIST1H3A,JUN,KMT2D,MAX,NFE2L2,NOTCH1,NRAS,PMS2,PTPN11,RAD51C,SOX9,SYK,TNFAIP3,TP53	0.439089762	1.119692903
path_1442	B2M,BARD1,BCOR,EPHA7,FLT1,HIST1H3I,INHBA,KNSTRN,MAP2K1,MAX,MED12,MUTYH,NKX3-1,NUF2,PAK1,PTCH1,RAD51,RASA1,RYBP,SPEN	0.144209099	1.281895777
path_1443	BLM,BRIP1,BTK,CDH1,CDK8,CDKN2C,CTNNB1,EIF4A2,EP300,FUBP1,HIST1H3H,KDM5C,KNSTRN,PDCD1,PDGFRB,PHOX2B,RAF1,SH2D1A,SMAD4,TCF7L2	0.6483533	1.081197232
path_1444	APC,CDK12,EED,EIF1AX,ERBB4,FH,FYN,HIST3H3,HLA-A,IGF1R,JAK1,KMT2D,MAP2K1,MAP2K4,MLL,NCOR1,PGR,PTEN,SH2D1A,SMAD4	0.078384396	1.324743379
path_1445	BARD1,CDH1,CEBPA,EPHB1,ERRFI1,FGFR1,FLT4,MALT1,MEN1,PDGFRA,PLK2,PMAIP1,RAD51D,RAF1,RBM10,RECQL4,RFWD2,RPS6KA4,SDHC,SOX17	0.482162267	1.130308594
path_1446	AKT1,CARM1,CTCF,EPHA3,ERBB4,ETV6,FGF4,GATA3,KEAP1,MLL,NEGR1,NPM1,NUP93,PLK2,PMS1,PPM1D,PTPN11,RAD51C,TNFAIP3,U2AF1	0.153091762	1.258541224
path_1447	ABL1,AR,ARID5B,B2M,BTK,CDH1,FAM175A,FGF19,KDM5A,MSH3,MST1R,MTOR,NOTCH3,PPP6C,PTPRD,RAD51,RUNX1,SPOP,TAP2,TP53BP1	0.129026984	1.238632328
path_1448	ARID1A,DAXX,DNMT3B,EPHA3,FGF4,FGFR1,FOXL2,FUBP1,FYN,GATA2,ICOSLG,KMT2D,MAP2K2,MAP2K4,PIK3R1,PTPRD,SDHAF2,SF3B1,SOCS1,TAP2	0.213932727	1.218691161

path_1449	ASXL2,CCND3,CDC42,CYSLTR2,DICER1,ERBB3,ESR1,EZH2,FGFR3,FGFR4,HIST3H3,HOXB13,MEN1,MPL,MYC,MYCL1,NTRK1,RAC2,RAD54L,RHOA	0.434024069	1.124094483
path_1450	ARID1A,CD79B,CDC73,EIF4E,FGF19,FGFR2,FH,HIST1H3B,MPL,MRE11A,NPM1,PMAIP1,PPM1D,PRDM1,PTCH1,RAD54L,RFWD2,RPS6KB2,SDHC,TGFBR1	0.65787325	1.066391314
path_1451	CDKN1B,EGFR,EIF4A2,FBXW7,FOXA1,FOXP1,GSK3B,JAK3,MEF2B,MPL,NCOA3,NPM1,PDGFRB,PIK3CG,PIK3R3,PMS1,PTPRS,RAD51B,SMARCB1,SOS1	0.152076648	1.267216293
path_1452	AXIN2,BLM,DNMT3B,EGFR,EIF4E,FGF4,FUBP1,HIST1H3E,INHBA,KDM5C,KDR,MAP2K1,MAP2K2,MAP2K4,PARP1,PNRC1,RAD54L,SMARCB1,SPOP,TP53	0.830937059	1.030640708
path_1453	ARID5B,ATM,BCL2L1,BRCA2,CBL,CSF3R,EIF4A2,FOXP1,GNAQ,H3F3C,KDM5A,LATS1,MALT1,MLL,NKX2-1,PHOX2B,PIK3R3,SPOP,SRC,TEK	0.648622351	1.081102206
path_1454	ATRX,B2M,BCL2,CBL,CCND1,DDR2,DROSHA,GATA1,GLI1,HNF1A,ICOSLG,KEAP1,LATS2,MYCL1,NCOR1,PGR,PPP2R1A,RPTOR,SMAD2,TAP1	0.279819691	1.184381945
path_1455	ARID1A,DOT1L,ETV1,HIST1H3D,HIST1H3J,INSR,KEAP1,KIT,KMT2A,MAX,PARK2,PMS1,RAD21,RTEL1,SDHAF2,SDHB,SDHC,SHQ1,TEK,TET2	0.639986047	1.072479297
path_1456	AMER1,CARM1,CCNE1,CDK12,CEBPA,EED,EIF4A2,EP300,EZH2,FGFR2,MALT1,MAP2K4,MYCL1,NTRK3,PMS1,REL,RET,RPS6KB2,RUNX1,SH2D1A	0.167534302	1.219550628
path_1457	CCND2,DAXX,EPHA3,EZH2,FLT1,GNAS,HGF,INHBA,MAP3K1,MSH2,NF1,NOTCH3,PHOX2B,PPP6C,RFWD2,RPS6KA4,SETD2,SMAD4,TAP1,TEK	0.181579527	1.222863082
path_1458	CCND2,CXCR4,EED,FANCC,GPS2,H3F3C,IFNGR1,IKZF1,IL10,INSRR,MAX,NCOR1,PDGFRB,PHOX2B,PNRC1,PTEN,RAD54L,RARA,SH2B3,SRSF2	0.406720889	1.126200929
path_1459	AMER1,B2M,BRCA1,CCND3,CDK12,CTCF,CTNNB1,EPHA7,EPHB1,FANCA,FGFR2,GNAQ,KIT,MEN1,MYCL1,PTPRT,RAD50,RAD51C,SF3B1,SRC	0.146847748	1.296375773
path_1460	AKT3,ATM,ATRX,AURKA,CDH1,ERCC2,FGF4,FUBP1,GATA1,KDM5C,KLF4,MEN1,NUP93,PMS2,RAC2,RET,RICTOR,ROS1,SDHB,SLX4	0.579655484	1.101187234
path_1461	BCOR,CARD11,CD274,CDC42,EIF4A2,ERCC2,FGFR3,FOXL2,INPP4B,IRS1,KDM5A,KDR,MALT1,MLL,MLL2,MSH2,MYCL1,NTRK2,PALB2,STAT5B	0.452302423	1.139935958
path_1462	ARAF,AURKA,BBC3,DNMT3A,EIF4A2,EPHB1,FANCC,FGF4,KMT2A,MSH3,PIK3CB,PPP2R1A,PRDM1,PTPRT,RAD21,SDHC,TBX3,TCF3,TP53BP1,TRAF7	0.140028041	1.232546285

path_1463	ANKRD11,ARID1A,CALR,CHEK1,CYSLTR2,DNMT3A,FANCA,FAT1,GATA1,H3F3C,KRAS,MLL,MSH6,NUF2,PDGFRA,PIK3R1,PIK3R3,POLE,PREX2,RHEB	0.354174753	1.145194666
path_1464	APC,AR,CDK12,CIC,DNMT3B,DOT1L,EIF4E,FLT1,FUBP1,FYN,KMT2D,MCL1,NEGR1,PDGFRB,PIK3CA,RAC2,SH2B3,SLX4,TAP1,TCF3	0.189568347	1.293200737
path_1465	AXIN2,CARD11,CRLF2,CSF3R,CXCR4,DIS3,DOT1L,EIF1AX,ESR1,EZH2,FANCC,FOXP1,MAP3K13,MCL1,NKX2-1,PARK2,PREX2,TAP2,TEK,TP53BP1	0.386208551	1.131109148
path_1466	ANKRD11,CSF1R,FAT1,FH,FLT1,HIST1H1C,HIST1H3D,IDH1,MED12,MSH6,NCOA3,PNRC1,PREX2,PTCH1,RAD21,RPS6KA4,TAP1,TERT,TMPRSS2,TNFAIP3	0.625851368	1.071203302
path_1467	ASXL1,BCL10,BLM,BTK,CBL,CYSLTR2,FGFR3,FOXL2,HIST1H1C,INHBA,KNSTRN,KRAS,MEF2B,NSD1,REL,RET,SDHAF2,SMO,SPOP,TAP1	0.415899732	1.13420842
path_1468	ATR,BCL6,CALR,CCNE1,CDKN1A,DICER1,ERBB2,EZH2,FGFR1,KDM5A,KEAP1,MPL,NTRK3,RAF1,RARA,SF3B1,SHQ1,SOCS1,SOX17,SOX9	0.133481042	1.279708904
path_1469	AKT1,APC,ARID1B,BTK,CASP8,DNMT3A,EIF4A2,EIF4E,ETV6,IFNGR1,KDM5C,MST1,NOTCH1,NUP93,POLD1,PRDM1,PREX2,RUNX1,SETD2,SMAD3	0.970804329	1.006241965
path_1470	ASXL2,B2M,BCL2L1,BRCA1,CD79B,CEBPA,EPHA5,GPS2,JUN,KIT,MITF,MLL2,NF1,PIK3C3,RAD51B,SOX2,SYK,TCF7L2,TRAF2,TSHR	0.307932369	1.177545043
path_1471	AURKA,BAP1,CSF3R,EIF1AX,EPHB1,FAT1,FLT3,GNAS,GPS2,HIST1H3B,KIT,LATS2,NOTCH4,PAK1,PDCD1,PTPRT,RBM10,ROS1,SUFU,TRAF2	0.313773982	1.164144474
path_1472	ACVR1,BBC3,BCL10,BLM,BRD4,CCNE1,CDH1,CYSLTR2,EED,EIF4A2,ERCC4,HIST1H3C,MAX,MLL2,MUTYH,NTRK2,PARK2,PDGFRB,SH2D1A,TAP2	0.246491767	1.179195151
path_1473	AKT2,AMER1,BCL2L1,BRCA2,CDC42,GATA3,HIST1H3G,MDM2,MPL,MRE11A,MSH2,NOTCH2,NPM1,PLCG2,RAD21,RAD51,RAF1,RARA,SHQ1,SMARCB1	0.150502493	1.23798506
path_1474	APC,BAP1,BMPR1A,CBL,CCND2,CDKN2A,CEBPA,CYSLTR2,GPS2,ICOSLG,PAK7,PIK3C3,PPP6C,PTCH1,RAD21,RASA1,RET,SDHAF2,TET1,TP53BP1	0.539258742	0.9001514
path_1475	CBL,CDH1,DICER1,ERBB4,FGF19,FGF3,FH,GSK3B,HIST1H3G,JAK1,JAK3,NEGR1,NSD1,PARK2,PMAIP1,PMS1,PNRC1,PREX2,SF3B1,STAT3	0.146811028	1.232148168
path_1476	ARAF,BRCA1,BTK,CENPA,CYSLTR2,DDR2,FLT4,GLI1,IFNGR1,IRS2,JAK3,MYC,NF2,PAK1,PLCG2,PMS1,PPP6C,SPOP,TCF7L2,TNFAIP3	0.121111448	1.315185751
path_1477	ATR,BCOR,CDK6,CENPA,CTNNB1,ERBB4,FGF19,H3F3C,HIST1H3C,HLA-A,IL7R,KDM5A,KRAS,LATS1,MAP2K2,MDM4,MSH3,POLE,RAF1,STK40	0.126478747	1.251058771

path_1478	ARAF,ASXL1,AXL,CXCR4,DNMT1,EED,EIF4A2,ERBB3,FGFR4,HIST1H1C,HIST1H3D,IKZF1,KMT2A,MCL1,MYOD1,NBN,NFKBIA,PIK3R2,RTEL1,TCF7L2	0.53393518	1.095930789
path_1479	ALOX12B,APC,AURKA,BMPR1A,CDKN1B,EIF4E,ERCC2,ERG,HIST1H3H,HIST1H3I,HIST3H3,INPP4A,JAK1,NCOR1,PIK3CD,PMS1,RAC2,RAD51C,RB1,RHOA	0.410939858	1.130617607
path_1480	BMPR1A,CBL,CCND3,CXCR4,EPHA7,FAT1,FGFR2,GNA11,HIST1H1C,IKZF1,KDM5C,MEF2B,MLL,MUTYH,PARP1,PIK3CD,TGFBR1,TNFAIP3,TP53BP1,TRAF7	0.538849777	1.090297157
path_1481	AKT2,CARM1,CD79B,ERCC5,FANCA,FAT1,FLT4,INHBA,JAK2,MAX,MET,MITF,MLL,MST1,NTRK1,NUF2,RAD21,RYBP,SMAD3,ZFHX3	0.068730078	1.344860807
path_1482	ARID5B,ASXL1,ATRX,CDKN1B,DNAJB1,FLT1,GATA1,INHBA,INSR,KDM6A,LATS2,MAP2K1,MITF,PARK2,PPP6C,PREX2,RPS6KB2,TAP1,TEK,YES1	0.307258336	1.154365023
path_1483	CBL,CDKN1A,CYSLTR2,DNMT1,DNMT3A,FAM175A,FBXW7,GRIN2A,HIST1H3D,HOXB13,ICOSLG,IL7R,NBN,NTRK2,NTRK3,PDCD1,PNRC1,RB1,RPS6KA4,SUFU	0.840863578	0.965638252
path_1484	B2M,CD276,CTNNB1,E2F3,EED,EGFR,ETV6,FGFR4,IKZF1,INHA,INSR,JAK3,MLL3,NFKBIA,NKX2-1,RAD54L,RB1,SDHB,SOS1,SYK	0.96467537	1.007062049
path_1485	ATR,BRD4,CEBPA,DICER1,DOT1L,GNAS,HIST1H3D,MALT1,MAP2K1,MAP3K13,MED12,MET,NFKBIA,NUP93,PARK2,PDCD1,PPM1D,RPS6KA4,SF3B1,SUFU	0.17594148	1.210513355
path_1486	BARD1,BCOR,CHEK1,EIF1AX,FANCA,FGFR2,GNAS,GPS2,INHBA,KDM5A,MDC1,MDM2,MST1R,PBRM1,PDCD1,RPTOR,SMARCD1,SPEN,SYK,TGFBR1	0.37774168	1.171002322
path_1487	CCND2,CCNE1,CDK8,CSF1R,DDR2,DNMT3B,ERG,ETV1,FAT1,GATA2,GPS2,IKBKE,MCL1,MYC,NCOR1,NUF2,PIK3CA,RHOA,ROS1,SETD2	0.057585635	1.3619744
path_1488	ABL1,AKT3,BRIP1,CALR,CD276,CDC73,CDKN1B,CENPA,CTCF,EIF4E,GATA3,KDR,MSH3,NFE2L2,PIK3CD,RB1,RFWD2,SPEN,SUFU,TET2	0.284487829	1.198769315
path_1489	ABL1,ARID5B,CBL,CD276,CDC42,DDR2,DNAJB1,EGFR,ERBB3,FOXP1,INHA,KNSTRN,MDM4,MUTYH,NPM1,REL,RNF43,RPS6KA4,SOCS1,TERT	0.757562506	1.057323002
path_1490	ARID5B,CDK4,CDK8,CHEK2,EPHA7,ERBB3,ETV1,FGFR2,FOXA1,HIST1H3A,HIST1H3B,HIST1H3D,INHA,PBRM1,PIM1,PREX2,RAD50,RHOA,RICTOR,TMPRSS2	0.104870498	1.259597198
path_1491	BCL2L11,BRAF,CEBPA,CTCF,DNMT3A,EPHB1,FGFR3,FLT4,FOXO1,GSK3B,IKBKE,MALT1,NBN,PARP1,PPP2R1A,PRDM1,PTPN11,RAD51B,RB1,TGFBR1	0.270802704	1.168112352
path_1492	AKT3,BBC3,BRD4,CCND1,CD276,DAXX,EIF1AX,EP300,EPHB1,ERBB3,IRS2,KDM5A,MSH6,MUTYH,RAF1,RARA,RPS6KB2,SOCS1,SRC,STAG2	0.807028226	0.965019407

path_1493	AURKA,BCL2,CD274,EIF1AX,EPHB1,FAM175A,FOXA1,HGF,HIST1H3A,INHA,KMT2D,MCL1,MPL,MRE11A,NSD1,PTCH1,RB1,SRSF2,STAT5B,TGFBR1	0.532451826	1.092429114
path_1494	CDKN2A,CENPA,EPHA3,ERBB2,ETV1,HIST1H3E,IGF2,KDM6A,MDM2,MITF,MYC,NCOA3,NOTCH3,PAK1,PIK3R2,RAD51D,SDHB,SH2B3,SRC,TET2	0.297301699	1.189590065
path_1495	BRCA1,CD79B,CDKN1B,DNMT3B,EPHA3,ERBB2,FGFR1,HIST1H3C,KMT2A,MCL1,MDM4,MGA,MTOR,MYOD1,NOTCH1,NRAS,PNRC1,SMARCB1,SMARCD1,SRC	0.33877294	1.182622796
path_1496	ARID5B,BCL6,CBL,CDK8,CYSLTR2,DAXX,ETV6,FAT1,FOXA1,HOXB13,INSRR,MAP3K1,MDM2,MLL2,MLL3,PAK7,PRDM1,SF3B1,SOX17,SOX2	0.170900122	1.250034005
path_1497	ARID2,B2M,BCL6,BTK,CTCF,FYN,GPS2,ICOSLG,KLF4,LATS1,MLL2,MYCL1,PAK1,PAK7,PGR,PMS1,RAD51,RAD51B,RPS6KB2,SPEN	0.126142387	1.330111929
path_1498	CD79B,CDK6,CHEK1,EPHA7,ERCC4,ESR1,ETV1,FAT1,FLT1,FUBP1,HIST1H3A,HIST1H3J,IFNGR1,INHA,JAK2,MALT1,MLL3,RASA1,SOCS1,TSC2	0.256854675	1.213563763
path_1499	BCL2,CARM1,CBL,CSF3R,EPHA5,GPS2,IL10,INSR,MPL,MRE11A,MSH2,NKX3-1,NTRK1,PIK3R2,PNRC1,SH2B3,SMAD2,SOX9,SRC,TCF3	0.930217613	1.012491689
path_1500	AKT3,ARID5B,ATR,B2M,BCL2,CDK6,CXCR4,CYSLTR2,DIS3,EPHA5,FGF19,FGFR3,GNAS,HGF,INHBA,INSR,MUTYH,PTPRD,RNF43,SF3B1	0.132091935	1.257057344
path_1501	AKT1,ARAF,AXIN2,CDK6,CREBBP,ERBB4,ESR1,GATA2,H3F3C,INHBA,IRS1,JUN,MYOD1,NOTCH4,PIK3C3,PTEN,ROS1,SDHAF2,SOX2,TMPRSS2	0.22310675	1.209023143
path_1502	ABL1,CHEK2,EGFR,FGFR3,FLT3,FOXO1,GATA2,GSK3B,HGF,HIST1H3I,KMT2A,LATS1,MALT1,MYCN,PTEN,REL,RFWD2,STAT3,TCF3,TOP1	0.501734807	0.893815194
path_1503	AR,BRAF,CCND2,CDC42,CDH1,CTLA4,CUL3,DAXX,DNAJB1,EIF1AX,FLT1,MEN1,NCOA3,NKX2-1,NOTCH2,PDGFRA,RFWD2,STAT3,TAP1,TAP2	0.278814418	1.165538095
path_1504	CSF3R,EIF4E,ESR1,FAM46C,FGF19,IKZF1,INHA,IRS1,KDM5A,KMT2D,MDC1,MDM4,MSH6,NOTCH1,PAK1,PIK3R3,SDHB,SOX17,STAG2,STAT5B	0.404088178	1.125179403
path_1505	ACVR1,ALK,ASXL1,BRCA1,CHEK2,CUL3,DROSHA,EPHA5,FGF3,GATA2,GRIN2A,HIST1H3G,IGF2,IRS2,KRAS,MED12,MRE11A,MYOD1,PAX5,RPTOR	0.615297892	1.074293557
path_1506	AKT2,ARID1A,ATR,ATRX,BCL10,CDKN2A,EGFR,EIF1AX,EZH2,FAT1,FLT3,GSK3B,HIST1H3J,IL7R,KLF4,MDM4,MLL3,MYC,PALB2,TAP1	0.20247486	1.202597835

path_1507	AKT3,B2M,CARD11,DROSHA,EED,GPS2,INSR,MYC,NOTCH1,NOTCH3,PAK1,PARK2,PGR,PHOX2B,PMAIP1,RHOA,SDHB,SMARCB1,SOX17,TP53BP1	0.089349779	1.272038581
path_1508	ARID2,ATM,BRAF,CIC,CTNNB1,DAXX,DROSHA,ERBB4,FGF3,GATA3,KIT,MALT1,MDM4,MLL2,PREX2,RAD21,RAD54L,SH2B3,SOS1,TCF3	0.460561115	1.127411214
path_1509	BCL2L11,CIC,CXCR4,DIS3,ELF3,ERCC2,ERG,FLT3,FUBP1,GNA11,HIST1H3H,HIST3H3,KDR,NKX3-1,PARP1,PBRM1,PDGFRA,PPM1D,RPTOR,SMARCA4	0.713675579	1.053265486
path_1510	ARID2,ATR,FGFR4,INSR,IRF4,KLF4,MAP3K13,MGA,MSH3,MSH6,MYCL1,MYOD1,NCOR1,NKX3-1,NTRK2,PAX5,PLCG2,SH2B3,SPEN,SRSF2	0.286704922	1.19973413
path_1511	ABL1,APC,ARID2,B2M,BCL2L11,BMPR1A,CARD11,CCND3,CHEK2,EIF4E,EPHA5,FOXP1,IFNGR1,MEF2B,NSD1,NUP93,PIM1,PTPN11,RAD54L,STAT5B	0.154000481	1.290384324
path_1512	ABL1,CD276,CD79B,CEBPA,CREBBP,CSF1R,DNMT1,EPHB1,FOXP1,HOXB13,IFNGR1,IL10,MRE11A,MYCL1,MYOD1,NCOA3,NOTCH2,PIK3CD,RHOA,TAP1	0.792803101	1.038686808
path_1513	ABL1,AURKA,AXL,BRAF,CTLA4,DNMT3B,EGFR,HIST1H1C,IRS1,JAK1,JAK2,MDM2,MEN1,MET,PDCD1,RFWD2,SF3B1,SMARCB1,STK40,TP53BP1	0.987566037	0.997561185
path_1514	B2M,BARD1,CASP8,CDH1,CDKN2C,CREBBP,DNMT3B,FAT1,FGFR4,HIST1H3A,KDM6A,MAP2K4,NBN,NFKBIA,PDGFRB,RARA,RNF43,SDHB,SMAD3,SMARCD1	0.297032477	1.214943937
path_1515	ARID1A,BARD1,CHEK2,CSF1R,DDR2,EPHA5,FAT1,INSRR,KDM6A,MAP2K2,MSH6,MST1R,NEGR1,PIK3CG,PTPN11,RECQL4,ROS1,SDHB,SF3B1,TAP1	0.05531657	1.326938145
path_1516	AKT3,ARAF,BARD1,CARD11,CCND2,CTLA4,CYSLTR2,ERCC4,FLT1,GATA2,IRF4,KDM5C,MAP2K2,MST1,NSD1,PIK3CD,RAD21,RAD54L,RPTOR,RTEL1	0.211659269	0.838142503
path_1517	BLM,CDK4,CREBBP,DNMT3A,FGFR2,FOXL2,IDH1,INSRR,MALT1,MET,PHOX2B,PIK3C3,PIK3CB,PTCH1,RAD51B,RPTOR,SH2D1A,SOX2,TEK,TGFBR2	0.482532746	1.138673897
path_1518	AKT1,BRCA1,BRIP1,CDC42,CIC,CTCF,GATA1,H3F3C,HIST1H3B,JAK3,MLL3,NUP93,PAK7,PBRM1,RAD51,RASA1,RHOA,RTEL1,SMARCB1,STAG2	0.378743145	1.133279977
path_1519	ALOX12B,BCL2L1,CDH1,DNMT3B,EIF4A2,ERBB3,ERCC2,FGFR1,HIST1H3B,IL7R,MAX,MTOR,NFE2L2,RFWD2,RPS6KB2,SMAD3,STAT5B,STK40,TBX3,TSHR	0.677770366	0.921538092
path_1520	ABL1,CENPA,CTLA4,DROSHA,FGFR1,IDH1,IFNGR1,IRF4,JAK3,KRAS,MED12,MSH6,NF2,NOTCH2,PAK1,PLCG2,PTPRD,SDHC,SLX4,SOX17	0.060992109	1.3061789

path_1521	ATM,ATR,BCL6,CASP8,CDC73,DIS3,DNMT1,FLT4,GNA11,ICOSLG,INHBA,JAK1,JUN,KDR,NCOR1,RBM10,RHEB,SLX4,TCF3,TERT	0.114886725	1.29102421
path_1522	B2M,BBC3,BRIP1,CDH1,CENPA,DIS3,DOT1L,EIF4E,FANCA,FOXL2,INPP4A,JAK1,MITF,MYCL1,NTRK1,PTPRD,RBM10,RYBP,SDHA,SH2B3	0.068412983	1.348032602
path_1523	ARID1B,ATRX,BARD1,CHEK1,CSF1R,CSF3R,CYSLTR2,IFNGR1,MDC1,MYCL1,PIM1,PNRC1,RBM10,RPS6KA4,SDHB,SMARCB1,SOCS1,SRSF2,TAP2,TCF3	0.419494281	1.15091759
path_1524	ASXL1,CREBBP,EGFR,EPHA5,ERBB2,FGFR1,FGFR3,FYN,GATA1,GATA3,HGF,KDM5A,MYC,NTRK3,NUF2,PMS2,RARA,ROS1,SOCS1,STK40	0.37493932	1.150889653
path_1525	BRCA1,ERBB2,ERG,FH,FLT4,FOXA1,FUBP1,IL10,IRS2,KEAP1,LATS1,MDM2,NBN,PIK3CD,PPM1D,RAD51C,RAD54L,SMAD4,SRSF2,TP53	0.673581987	1.069296117
path_1526	CDKN1A,CHEK1,FAM175A,FGFR2,GATA1,H3F3C,INSRR,NKX3-1,NOTCH3,PIK3CG,PIK3R1,PIK3R3,PMS2,RAD51,RHEB,RHOA,RPS6KB2,SH2D1A,SUFU,TGFBR1	0.299958079	1.160719259
path_1527	ARID2,CDKN1B,CSF1R,DAXX,EED,EGFR,ETV1,FH,H3F3C,HIST1H3C,MAP3K13,MST1R,NOTCH1,NTRK2,NTRK3,PGR,PTPN11,RAD51,SOCS1,SRC	0.942810172	1.011942625
path_1528	BRD4,CTCF,EIF4E,ERBB2,ERBB3,FGF19,FGFR4,INSRR,IRS1,KMT2A,LATS1,NF2,NFKBIA,PAX5,PDGFRB,RUNX1,SH2D1A,TERT,TOP1,U2AF1	0.081888705	1.443353595
path_1529	ARID2,AURKA,BCL2L1,EIF1AX,ERCC4,ESR1,FOXO1,IRF4,KDR,KIT,MAP2K2,MSH2,MYCL1,NPM1,PIK3CD,PIK3CG,PMS2,PPP2R1A,PTPRD,SF3B1	0.166104477	1.246214962
path_1530	BBC3,BCL2L1,BRD4,BRIP1,CENPA,DAXX,DNAJB1,DOT1L,ELF3,ETV6,FAM175A,FGFR4,GSK3B,HGF,ICOSLG,MLL,PMS1,RAD21,RTEL1,SOX9	0.138867198	1.233528869
path_1531	BCL2L1,BTK,CARM1,CDKN1B,CHEK2,CTCF,DNMT3B,HIST1H1C,HIST3H3,JAK2,LATS1,MSH2,PHOX2B,PIK3C3,PIK3CG,PMS1,RAD50,SH2B3,STK11,TSC2	0.782410071	0.957860236
path_1532	AKT1,AR,ARID5B,BCL6,BRCA1,CARD11,DAXX,E2F3,EPHB1,FGF19,GNAS,HIST3H3,IKBKE,MSH3,NBN,PBRM1,PMAIP1,PREX2,SHQ1,SOX9	0.693473083	0.944262906
path_1533	BCL2L1,CCND2,CDH1,CDK4,CTLA4,DNMT1,ETV6,FOXP1,GNA11,LATS1,MAP2K1,MDM2,MYCN,PLCG2,PNRC1,RAD54L,RNF43,SMO,SOS1,SYK	0.203830744	1.1990839
path_1534	ANKRD11,BLM,BMPR1A,CENPA,DIS3,DNMT3A,ETV6,FGF19,INSRR,IRS2,JAK2,JUN,KEAP1,MCL1,MLL2,PALB2,PIK3CA,PIK3CG,SH2D1A,TMPRSS2	0.764969369	0.957095004

path_1535	ASXL1,CCNE1,CREBBP,CTCF,EPHA7,FGF19,FGFR2,HNF1A,ICOSLG,IRS2,MLL2,MYOD1,NCOR1,NOTCH1,PIK3CD,PIK3R3,POLE,PRDM1,RPTOR,SUFU	0.185875343	1.241263852
path_1536	BLM,BTK,CARD11,DAXX,DICER1,DNAJB1,FGFR1,FUBP1,HNF1A,INSR,JAK2,JUN,MAP3K13,NOTCH4,PARK2,PDGFRB,PIK3CB,PMS1,RNF43,RTEL1	0.850849784	0.973744247
path_1537	ARID1A,ARID1B,ATM,AXIN1,BCL2L1,CHEK2,DICER1,DOT1L,ETV6,KIT,MED12,MITF,NBN,NFKBIA,PGR,PIK3R1,PMS2,RB1,SHQ1,SRSF2	0.126388875	1.271538973
path_1538	AXIN2,BMPR1A,BRCA2,CD79B,CDK6,EIF4A2,EZH2,FYN,GNA11,GNAQ,H3F3C,NRAS,NTRK3,PAK7,PIK3R3,PPM1D,RYBP,SMARCD1,SOCS1,TERT	0.479999932	1.105419073
path_1539	ANKRD11,ARID5B,CARM1,CD79B,CDC73,CDKN1B,DOT1L,ERBB3,HIST1H3A,KDM6A,KDR,MAP2K1,MEN1,NBN,PIK3CB,POLD1,SDHA,SPOP,TEK,TET1	0.148813661	1.2309695
path_1540	AKT1,AXL,CTNNB1,FGF4,HIST1H3J,IKBKE,KDM5C,MITF,MYC,NEGR1,NF2,NRAS,PHOX2B,PIK3CA,RASA1,RNF43,RPS6KA4,SMO,TBX3,TP53BP1	0.871282159	1.030471901
path_1541	ABL1,ATM,CARM1,CDKN2C,CEBPA,CRLF2,CTCF,EED,ERBB2,ERG,HIST3H3,IL7R,MAP3K1,MDC1,NKX3-1,PIK3C3,POLE,RAD21,RAD51D,RECQL4	0.393329657	1.155828266
path_1542	AKT2,CDC42,DIS3,EPHA5,EPHA7,HIST1H3G,IKZF1,KLF4,MAP3K13,NCOA3,NEGR1,NKX2-1,PAX5,PIK3CD,PREX2,PTPRS,STK11,TAP1,TGFBR1,WT1	0.127104258	1.256373369
path_1543	BBC3,CASP8,CDC42,CIC,CSF3R,EPHA3,FAT1,FGFR1,FH,FLT1,HIST1H3I,KDM5A,MAP3K1,MUTYH,NOTCH2,PDGFRA,PIK3CG,PREX2,PTEN,SMARCA4	0.06726211	1.305849592
path_1544	B2M,CARD11,CD276,CHEK2,CSF1R,EPHA5,ERCC2,FBXW7,HIST1H1C,IL10,INSR,MCL1,MYCN,NOTCH2,PPM1D,RPS6KA4,RPTOR,SMARCD1,STAT5B,WT1	0.308185717	1.20030197
path_1545	ABL1,BCL2,CEBPA,CHEK2,DNMT3A,ELF3,FGFR1,FYN,GPS2,ICOSLG,INHA,KEAP1,LATS1,MYCN,NOTCH3,PARP1,PIK3R3,PMAIP1,SDHAF2,SYK	0.920946722	0.983799827
path_1546	ANKRD11,CDK6,CDKN2A,CENPA,EP300,FBXW7,GNAS,GRIN2A,IGF2,MED12,MLL3,MRE11A,MST1,NKX3-1,NOTCH3,PIK3C2G,POLE,RAD51C,RHEB,TEK	0.057277182	1.362498242
path_1547	ARID1A,AXIN1,BRCA1,CHEK1,CREBBP,EPHA3,FOXL2,HIST1H3D,IGF2,MALT1,MDC1,MDM4,MET,NCOR1,PREX2,RAD51,SETD2,SMAD3,TCF7L2,TGFBR2	0.596891995	1.086432856
path_1548	ALK,ASXL1,BRCA1,CTCF,ERCC5,FBXW7,FOXP1,GRIN2A,INSRR,KDR,MPL,NCOA3,PAK1,PLK2,PTEN,PTPRD,REL,SMAD4,SRSF2,STAT5B	0.891793758	1.020827506

path_1549	AKT1,AKT2,DNMT3A,DNMT3B,FGF19,IDH1,KNSTRN,MDM4,MST1R,PAK1,PARP1,PIK3CA,PIK3CG,PIM1,PRDM1,RPS6KA4,SLX4,SMO,SOX9,XPO1	0.213884385	1.192171148
path_1550	BARD1,BLM,BRD4,BRIP1,CASP8,CIC,DROSHA,ERBB2,FLT4,JAK2,KEAP1,NEGR1,NF2,NOTCH3,NRAS,RAD50,RPS6KB2,SH2B3,SMAD4,TBX3	0.30384282	1.162497148
path_1551	ALK,AXIN1,BCL2L1,CEBPA,CRLF2,FGFR2,FLT4,HIST1H3H,HLA-A,IDH1,INHA,INPP4A,IRS1,KMT2D,MAP2K1,MST1R,PTEN,RHOA,SOS1,TET1	0.066795209	1.30053816
path_1552	ATR,CDC73,CDH1,CDKN2C,CTNNB1,EGFR,ERBB2,ERG,FGF3,KDM5A,KDR,MDM2,NCOA3,PDCD1,POLD1,PREX2,RHEB,SMAD4,TBX3,TGFBR1	0.929994933	1.014115956
path_1553	ATM,BRCA1,BRIP1,CCND3,CD274,CENPA,CHEK1,EIF4E,FGFR3,FLT3,GRIN2A,INHBA,MEN1,NCOA3,PHOX2B,POLD1,SDHAF2,SPEN,SYK,TOP1	0.065849819	1.439705432
path_1554	ARAF,AXIN1,BRD4,CEBPA,CSF1R,ETV1,FUBP1,GATA2,HGF,MST1,NKX3-1,PPP6C,PTEN,PTPRD,RB1,SDHAF2,SMARCB1,SOCS1,SPEN,SRSF2	0.172648673	1.245471178
path_1555	BCL2,BCL6,BTK,CDK4,CENPA,DIS3,ELF3,ERCC4,ERRFI1,EZH2,GATA1,IGF2,INHBA,MGA,NFE2L2,PIK3CB,PLK2,PNRC1,SMARCB1,TGFBR1	0.870656212	0.977349643
path_1556	BARD1,CDC42,CDK6,CEBPA,CSF1R,CTLA4,EPHA5,FH,HIST1H3C,HLA-A,ICOSLG,NBN,NEGR1,PIK3R1,PMS2,RAD51B,SDHB,SHQ1,SMAD4,STK40	0.654317575	0.938638712
path_1557	ALOX12B,BBC3,BCOR,DICER1,DNAJB1,DOT1L,GATA3,HGF,INSRR,IRF4,MALT1,MLL3,MRE11A,PDGFRA,PIK3CD,PMAIP1,POLD1,RPS6KA4,SDHC,SF3B1	0.227278899	1.21655854
path_1558	ANKRD11,APC,BARD1,BRAF,BRIP1,CDK12,CRLF2,CYSLTR2,DICER1,EIF4A2,FAT1,IRS2,MDC1,PBRM1,PIK3C2G,PIK3C3,PTPRS,SDHA,SLX4,STAG2	0.24091657	1.193278302
path_1559	CHEK2,CUL3,DNMT3B,EPHA7,FH,HIST1H3C,INPP4A,INSR,IRS1,KRAS,MLL,PAK1,PTPRD,RHEB,RPS6KA4,SOX17,SYK,TCF7L2,TERT,YES1	0.295426596	1.162615787
path_1560	ACVR1,B2M,BAP1,BCL10,CUL3,DIS3,EPHA7,ERCC4,FGF3,FOXP1,HIST1H3G,HIST3H3,INPP4A,INSRR,KDM5A,MAP2K2,PTPN11,RBM10,SMARCB1,SOCS1	0.186646753	1.272780456
path_1561	AXIN2,BCL2L1,BMPR1A,CCNE1,DNAJB1,E2F3,ERBB4,HIST1H3E,HIST1H3I,HOXB13,KDM6A,MLL2,NKX2-1,PDGFRA,PIK3R1,PLK2,PPP2R1A,REL,RPS6KB2,SDHA	0.592874641	1.078733081
path_1562	ARID5B,ATR,AXIN2,CRLF2,DAXX,EP300,EPHB1,FBXW7,HIST1H3H,IDH1,INPP4B,NSD1,PPM1D,PTPRS,RAD51B,RB1,RECQL4,RET,TSC1,U2AF1	0.144608366	1.282273908

path_1563	AKT3,AURKA,CENPA,CSF3R,DNMT3B,EED,ERBB3,HLA-A,KRAS,NEGR1,PDCD1,PTCH1,RAF1,RHEB,SMAD4,SMARCD1,SYK,TP53,TRAF2,U2AF1	0.057324747	1.389096202
path_1564	ASXL1,CIC,EPHA5,FLT1,FLT3,FUBP1,GLI1,HIST1H3J,HOXB13,INSRR,KDR,LATS2,MYCL1,PLCG2,PNRC1,RB1,RPTOR,SMAD4,STAG2,TGFBR1	0.073226391	1.326758647
path_1565	ATR,B2M,BRAF,CCND3,CTNNB1,ERCC4,ERCC5,HIST1H1C,INHA,MAP2K1,MAP3K1,MST1,NFE2L2,PDCD1,PIK3R1,PMAIP1,RAD21,RAD54L,SDHC,SOX9	0.636314137	1.069354506
path_1566	BRCA1,CD276,CDC73,CXCR4,DNMT3B,ERBB3,FGFR1,FLT4,IL7R,KDM6A,KEAP1,KIT,KMT2A,MYOD1,PDGFRA,PIK3CG,PIK3R1,RTEL1,SH2B3,SRSF2	0.928966337	0.986834791
path_1567	ABL1,ANKRD11,CASP8,CCND3,CCNE1,CD276,CDK12,CSF1R,CTCF,ERCC5,FAM175A,FGF19,HIST1H3A,IGF2,LATS2,MEN1,SDHAF2,SMAD4,SMARCD1,SOS1	0.803139212	1.03622674
path_1568	ABL1,ASXL1,BRAF,CD276,CEBPA,DIS3,EPHA7,EPHB1,FAM46C,FOXA1,GNA11,INHA,INPP4A,MAP3K13,MEN1,MPL,PDGFRB,PTEN,PTPRS,TBX3	0.05417557	1.403997264
path_1569	ASXL2,AXL,BRAF,CENPA,DIS3,ERG,ESR1,GATA1,IKBKE,IKZF1,KDM5A,KNSTRN,MSH2,PTPRS,RAD51,SMAD2,SMAD4,SOX17,STK11,TAP2	0.370568481	1.153214369
path_1570	BCL2,DNMT3A,ERBB2,ERCC4,FH,FLT4,GLI1,INHBA,KDR,MAP2K1,MST1,PIK3C2G,RAD51C,RASA1,RBM10,RPS6KB2,RTEL1,SDHAF2,TAP1,TP53	0.462394441	1.109473637
path_1571	ARID2,AXL,BCL2,BCL2L1,ERRFI1,H3F3C,IKBKE,IRS1,KDM6A,MED12,MGA,MITF,MPL,MSH2,NSD1,PIK3CA,RAF1,SYK,TAP1,YAP1	0.090124065	1.37611489
path_1572	DICER1,ERBB4,EZH2,FAT1,FH,FOXL2,HIST3H3,IDH1,IFNGR1,IKZF1,KMT2A,LATS2,MAX,MDC1,MYCL1,NFE2L2,PLK2,PPP2R1A,RARA,SMAD4	0.542255549	1.103104218
path_1573	AKT2,ATR,AXIN2,BBC3,CDC42,CTCF,DAXX,DNMT3B,EIF1AX,ERBB2,GATA2,HLA-A,KDM5C,KLF4,LATS1,NF1,PDCD1,PPP2R1A,RICTOR,RPTOR	0.763873784	1.045351792
path_1574	AKT1,AKT3,ATR,CASP8,CD274,CEBPA,CTCF,ERRFI1,FGFR2,HIST1H3B,IKBKE,INPP4B,MST1,MTOR,NCOA3,PALB2,RAD54L,RUNX1,SH2B3,SMO	0.617701874	1.07508893
path_1575	ASXL1,BAP1,CCND1,CDK8,EPHA7,EZH2,FGF4,HIST1H3D,IGF2,INSR,KMT2D,MDM4,MED12,NCOR1,NOTCH1,RARA,RHEB,RPS6KA4,SLX4,TAP1	0.479613558	1.149135844
path_1576	AMER1,AR,BCL2,BCL2L1,BTK,CCND2,CD276,CDC42,EP300,ETV1,GATA2,HIST3H3,IGF1R,IKBKE,MET,PAX5,RAD54L,SDHB,SPEN,YAP1	0.094493789	1.271062142
path_1577	ALK,ARID2,CREBBP,EIF1AX,EPHA5,FGFR3,GNAS,JAK2,KNSTRN,MLL2,MLL3,MUTYH,NEGR1,NOTCH3,NPM1,RICTOR,RTEL1,SHQ1,SMARCB1,TEK	0.305949319	1.165028269

path_1578	ALOX12B,ATM,ATR,CDK6,CTLA4,E2F3,HOXB13,IKZF1,JAK1,KNSTRN,LATS1,LATS2,MAP2K1,MCL1,NF1,PDGFRA,PLCG2,RFWD2,RICTOR,STK40	0.600787284	1.092999115
path_1579	ALK,ASXL1,CDKN2A,EP300,EPHA3,FGF4,FH,FYN,GNAS,IL7R,MAP2K4,MGA,NFE2L2,NKX3-1,PPP2R1A,RAD54L,RHEB,STK11,TERT,TET1	0.126322875	1.245872551
path_1580	BRCA1,CBL,DNMT1,ETV6,FANCA,FGF3,IKBKE,INHA,KDM5C,MAX,MRE11A,MST1,PDCD1,PIK3R1,PMS2,PNRC1,PTPRD,RFWD2,SOX9,TSHR	0.143261585	1.267548864
path_1581	ARID2,AURKA,BLM,BRIP1,CD274,CDKN2A,CXCR4,FLT4,FOXP1,GATA3,MYC,NEGR1,NOTCH4,NRAS,NTRK2,PAK1,PDGFRB,PIK3CG,RAD51,SHQ1	0.343107961	1.155061016
path_1582	ALK,AMER1,ARID5B,AXIN2,CARM1,ELF3,EPHA5,FGFR1,FUBP1,IFNGR1,MET,MPL,MYCN,NTRK1,REL,SDHB,SMAD2,SMAD4,SRC,TP53BP1	0.339501224	1.169867869
path_1583	AKT3,ALOX12B,AMER1,ANKRD11,ASXL1,ASXL2,AXL,BCOR,DICER1,DIS3,DNMT1,MYCN,NCOA3,NFKBIA,NPM1,PIK3R1,POLD1,PTPN11,PTPRS,RET	0.408345565	1.124719309
path_1584	BCL2,BRAF,BRCA1,CALR,CD79B,CHEK1,CHEK2,EIF4A2,ETV6,HIST1H3E,JUN,MYOD1,NOTCH3,NRAS,NSD1,PLCG2,PTPRT,RAC2,RAD51C,TAP2	0.059770709	1.303703937
path_1585	ABL1,ALK,ATR,DNMT1,EPHA7,ESR1,FANCC,GLI1,GPS2,HIST1H3B,INPP4B,KLF4,MDM4,MED12,MET,MPL,NTRK3,RAD21,RBM10,SDHAF2	0.134834112	1.264256292
path_1586	AKT2,ASXL2,BCL2,CCND2,CCND3,DNAJB1,EP300,FLT4,FOXA1,GSK3B,MSH6,NCOA3,NOTCH2,PARK2,PLK2,PMAIP1,RET,SDHA,SMAD4,SOCS1	0.164641585	1.215766531
path_1587	AKT3,APC,BLM,CDC73,CDK12,EED,FANCA,FGFR1,FLT3,HIST1H3E,LATS2,MAP2K2,MCL1,MYCN,NOTCH1,RET,SRC,TEK,TERT,TGFBR2	0.420593267	1.160587181
path_1588	BAP1,CDK12,EIF4A2,ERCC5,ETV1,FGFR2,GATA2,HGF,IL7R,INSR,KMT2D,MPL,MYCL1,PARP1,PBRM1,RAD51,RAD51D,SLX4,SOCS1,TEK	0.124730391	1.240942594
path_1589	ARAF,CCND3,CRLF2,DAXX,ERBB3,ETV1,FAM46C,FANCC,GATA3,IL7R,JUN,KDM5C,MAP3K13,NCOA3,PBRM1,PPP2R1A,RB1,RET,RNF43,STK40	0.879735628	1.021940168
path_1590	AR,B2M,BRCA1,CDK12,CDKN1A,GNA11,HIST1H3C,IDH1,IL10,JUN,MAP2K2,MYOD1,POLE,PTCH1,RAD21,SETD2,SHQ1,TAP2,TNFAIP3,U2AF1	0.610022568	1.075112218
path_1591	ATR,BBC3,CCND1,CENPA,DNAJB1,EP300,FAM175A,HIST1H3C,IFNGR1,KDM6A,MTOR,NPM1,NUF2,PMAIP1,RB1,RET,RPTOR,SHQ1,SMAD2,TSC1	0.567842099	1.086306056

path_1592	ANKRD11,CDC42,CHEK2,FOXA1,HIST1H3A,IFNGR1,JUN,KDM6A,KDR,MDC1,MSH2,NBN,PARP1,PIK3R2,PIM1,RPS6KA4,SDHB,SHQ1,TEK,TOP1	0.932685918	0.987873879
path_1593	BTK,CDK8,DROSHA,EGFR,EIF1AX,ELF3,FGFR2,GNAQ,HIST1H3J,IRS2,MCL1,MDM2,PBRM1,PIM1,PPP2R1A,RARA,RECQL4,RPS6KA4,TSC1,U2AF1	0.08669892	1.435852959
path_1594	ARID1A,BBC3,BRCA1,BRD4,CDKN1A,ERBB4,FANCC,FGFR1,GATA1,KMT2D,KNSTRN,MAP3K13,MDM4,NRAS,PIK3R2,RNF43,SMAD3,SMARCB1,SOX17,TCF3	0.087195642	1.374877552
path_1595	BMPR1A,CCND3,CD276,CREBBP,DDR2,ERBB3,GATA3,INHBA,KLF4,LATS2,MAP2K4,MDM2,NTRK1,SH2D1A,SOCS1,SOX17,STK11,TCF3,TCF7L2,TRAF2	0.957174188	1.008624066
path_1596	ABL1,ASXL1,ASXL2,BMPR1A,CUL3,ERRFI1,ESR1,GATA3,IRS2,KIT,KMT2D,MAP2K1,MSH6,NUF2,PAK1,PLCG2,RAD54L,STAG2,TET1,TGFBR1	0.611245883	1.089395102
path_1597	APC,ARID5B,BRAF,CD79B,CDKN2A,DROSHA,ERBB4,ICOSLG,MET,MUTYH,MYOD1,NKX3-1,NTRK2,PARK2,SETD2,SMARCB1,SOX9,STAT3,TCF3,TNFAIP3	0.408353611	1.142517904
path_1598	ASXL1,AXIN1,CDC73,CDK4,CXCR4,EGFR,EIF4A2,FOXO1,MAP2K1,MAP3K13,NKX3-1,PMS1,RAD51D,SOCS1,SRC,STAG2,STAT3,STK40,SUFU,TRAF7	0.409769991	0.836857219
path_1599	ACVR1,AKT2,AMER1,ARID1A,CDK8,CDKN1B,CHEK2,DDR2,ETV1,GNAQ,MLL2,MSH6,PDGFRA,PIK3R3,PTPRS,RAD51,RB1,RFWD2,RHEB,SOX17	0.060624184	1.318148567
path_1600	AXIN1,DAXX,EPHA7,ETV6,FANCA,GNA11,GNAS,HIST1H3C,HIST3H3,IL7R,KRAS,LATS1,MED12,MITF,RFWD2,RPS6KA4,RTEL1,TCF7L2,TRAF2,TSHR	0.151287823	1.249497843
path_1601	AKT3,ALK,ANKRD11,AURKA,CARM1,CHEK2,CREBBP,EP300,HIST1H3A,KEAP1,MAP2K1,NEGR1,PAK1,PTPN11,PTPRS,RASA1,RET,RNF43,SMARCD1,SRSF2	0.750121642	0.952907407
path_1602	AKT1,CTNNB1,DROSHA,ELF3,FAM175A,FOXL2,IGF2,IKBKE,KIT,MST1R,MYC,NBN,NOTCH2,NOTCH4,PIK3CA,RAF1,RNF43,RTEL1,SMAD3,TOP1	0.859890738	1.032182434
path_1603	ARID2,BCL2,DAXX,EP300,EPHA3,FAT1,GATA1,HIST1H3D,HIST3H3,INPP4A,KDM5A,MDC1,NOTCH2,NTRK2,PLCG2,PMAIP1,PREX2,RECQL4,RUNX1,SRSF2	0.136392007	1.267625409
path_1604	ARID5B,CD274,CENPA,EPHA3,IGF1R,IL7R,INSRR,KDM5A,MDC1,MET,MTOR,NF1,PAK1,PNRC1,RAD51C,RHOA,RYBP,SDHC,TRAF2,U2AF1	0.07384983	1.351379935
path_1605	APC,AXIN2,CD274,CDKN1B,CHEK2,CSF3R,DROSHA,FGFR3,GATA1,HLA-A,MAP3K1,NOTCH3,PIK3CD,PPP2R1A,PPP6C,RHOA,RPS6KB2,SOCS1,SOX9,TMPRSS2	0.278393235	1.179152646

path_1606	BAP1,CBL,CYSLTR2,ERBB4,ETV6,GATA1,HGF,HIST1H3E,IKBKE,MAP3K13,MCL1,MUTYH,NOTCH4,PARP1,PIK3R2,PPM1D,RHEB,SMAD2,SMAD3,SYK	0.265054725	1.211613621
path_1607	ARAF,BCL2L1,CBL,CD274,FAT1,FLT1,GLI1,HLA-A,INSRR,IRS2,LATS1,LATS2,MET,MSH6,PIK3C3,PIK3R2,RECQL4,RPS6KB2,SH2D1A,SMO	0.096637705	1.311115942
path_1608	ATR,ATRX,BRD4,CCND2,CDK8,CRLF2,FLT4,GATA1,GPS2,KLF4,PALB2,PAX5,POLE,PTPN11,PTPRT,RICTOR,RTEL1,SPOP,STK11,TAP2	0.068394373	1.32938202
path_1609	AMER1,ASXL1,BCL2,BRD4,CARD11,CHEK2,CRLF2,DAXX,DIS3,E2F3,FGFR3,HIST1H3H,NCOA3,NUP93,PTCH1,RET,RHEB,SDHB,TBX3,TET1	0.2973497	1.159581199
path_1610	ALOX12B,ARID1B,ARID5B,BCOR,BRCA1,CD276,CDH1,EP300,FAM46C,FGFR4,FH,FLT1,FUBP1,HIST1H3D,KLF4,MDM4,MLL2,NSD1,NTRK3,NUF2	0.314841067	1.181135063
path_1611	ARID2,AXL,BCL2L1,CDKN2A,CHEK1,ICOSLG,JAK2,KRAS,MDM4,PLCG2,PNRC1,RAF1,RET,RNF43,SHQ1,SPOP,STAT5B,SYK,TOP1,TSHR	0.32022157	1.162532569
path_1612	ARAF,CCND1,CTCF,DDR2,FANCC,FGFR4,FOXL2,GRIN2A,IGF1R,INPP4B,IRS1,IRS2,KEAP1,KIT,MCL1,NPM1,NTRK3,PMAIP1,SDHC,SETD2	0.567245172	1.086819514
path_1613	ATM,AXIN2,CCNE1,CDC42,CDK8,CTLA4,DDR2,GNAQ,HIST3H3,IRS2,MAP2K1,MDM2,MST1,NOTCH2,PMS1,PNRC1,RET,RPTOR,SRC,TEK	0.784020186	0.961732273
path_1614	APC,CBL,CCND1,CDK6,DNAJB1,EZH2,FAM175A,HIST3H3,KDM5C,MAP2K1,MAP3K13,MLL,MST1,NUP93,PTEN,PTPRD,RAD51,RAD51B,SMARCB1,SOS1	0.172656208	1.268204024
path_1615	AKT1,ANKRD11,BMPR1A,CCND3,CDKN1B,ERBB2,FAM46C,KMT2A,MAP3K1,MDM2,MRE11A,MST1R,PTCH1,RAD51,RECQL4,RPS6KB2,SH2B3,SMAD4,STAT5B,SYK	0.752630783	1.045946878
path_1616	ABL1,AMER1,BRCA1,CDC73,CDKN2C,EGFR,ETV1,GATA3,INPP4A,KDM6A,KMT2D,MGA,MST1,NTRK2,PIK3CD,PTPN11,RAD51C,RNF43,SMARCA4,SRC	0.316482592	1.172768836
path_1617	ACVR1,AXIN2,CARM1,CDK4,CIC,CSF1R,EIF4E,FGFR3,HIST1H3C,INHA,IRS1,LATS1,MDM2,NCOR1,NPM1,ROS1,RPS6KA4,RPS6KB2,RYBP,SUFU	0.050873649	1.326799889
path_1618	ARAF,ARID1B,BMPR1A,CASP8,CSF1R,DICER1,EPHA7,ERCC4,FGF3,IDH1,KIT,MAP2K1,MSH2,MSH6,NTRK3,PIK3CG,PIM1,PTCH1,RASA1,RYBP	0.561499589	1.101191248
path_1619	APC,EIF1AX,ERBB4,ERCC4,GATA2,HIST3H3,INSRR,JUN,KMT2A,MCL1,MTOR,MYCL1,NBN,PIK3CD,RAC2,RAD50,RHOA,SDHAF2,SPOP,TP53BP1	0.644237662	1.069110635

path_1620	CBL,CCND2,CDC42,CDKN1A,CDKN2A,CRLF2,CXCR4,ERCC4,FOXA1,GRIN2A,HIST1H3D,NOTCH4,NUP93,PDGFRA,PHOX2B,PIK3CG,PMAIP1,RAC2,SMAD2,TGFBR1	0.884801536	0.975862495
path_1621	ARAF,ARID5B,BRCA2,CBL,H3F3C,ICOSLG,IL10,IL7R,MAP2K4,MAP3K1,MDM4,NOTCH4,NTRK3,PIK3CA,RASA1,RHOA,SMAD3,TCF3,TP53,TSHR	0.150447523	1.227140406
path_1622	ALK,ANKRD11,ARAF,BCL10,BCL6,DICER1,EPHB1,ERBB4,MET,MRE11A,MYC,NPM1,NUF2,PMS1,PTEN,PTPN11,RAF1,RB1,RET,RICTOR	0.322278318	1.178302329
path_1623	ANKRD11,ARAF,ATRX,BTK,EIF1AX,EIF4E,ERCC2,ERCC4,FYN,GATA3,INHA,MYOD1,PAX5,PBRM1,PIK3C3,REL,RPS6KA4,RPS6KB2,SOX9,SYK	0.106818166	1.254840231
path_1624	BAP1,CALR,ERCC5,FANCA,FAT1,FGFR3,FLT3,FUBP1,GATA1,GPS2,JAK3,MALT1,MAX,MED12,MLL3,POLE,PPP6C,SOS1,SOX2,TEK	0.314905509	1.187622113
path_1625	ABL1,AKT2,AR,BRIP1,BTK,CCNE1,CDC42,FLT3,GATA2,GRIN2A,HIST1H3G,HIST1H3I,KDM6A,MSH3,NEGR1,PIK3R2,POLE,STK11,TAP1,TSC1	0.230439063	1.2064689
path_1626	CCNE1,CDC73,CDH1,CDK8,DROSHA,EED,ERG,FAM46C,HIST1H3G,IFNGR1,MSH2,MYC,PHOX2B,PLK2,PTPRS,PTPRT,RAF1,RET,SLX4,SMARCD1	0.260651653	1.177736916
path_1627	ALOX12B,ARID5B,CARD11,CDC42,CDKN1A,EP300,EPHB1,FANCA,IL7R,INPP4A,LATS1,MDC1,MITF,PIK3CD,PIM1,PLK2,RAD51,REL,SH2D1A,STK40	0.558217274	1.088820725
path_1628	BCL2L1,CCND2,EIF4E,ERG,IKBKE,IL7R,KEAP1,NKX3-1,PARK2,PDGFRA,PHOX2B,PIK3CG,PIK3R3,RAD51B,RECQL4,RPTOR,SDHB,SMARCD1,TET1,YES1	0.238438174	1.206090957
path_1629	ATM,BCL2L1,BRCA2,CCND1,CD276,CDH1,CDK4,EPHB1,ERBB4,GATA1,H3F3C,IFNGR1,IL10,JAK3,MET,PIK3R3,PTPN11,SMARCB1,TAP1,TGFBR1	0.852633822	1.03254055
path_1630	ALOX12B,CDH1,CTCF,DAXX,DNAJB1,FANCC,FGFR3,GATA3,HIST1H3D,IL10,KMT2D,MET,NTRK1,PGR,RAD51B,RAD51D,RARA,RB1,SMO,TCF3	0.066295018	1.439979886
path_1631	ATRX,B2M,BRD4,BRIP1,DOT1L,DROSHA,ERBB4,ESR1,FGFR2,GPS2,IL10,KMT2D,NFE2L2,NOTCH2,PAK7,PIK3CB,RAD51D,RASA1,RET,SETD2	0.227217429	1.2014333
path_1632	BCOR,BLM,BRCA1,CD274,CDK12,DAXX,DROSHA,ERCC4,ETV1,FGFR2,FUBP1,IRF4,KDM5A,KRAS,NKX3-1,NTRK1,NUF2,PMAIP1,PRDM1,RAD51B	0.717048535	1.060001346
path_1633	ARID1B,FANCC,FGFR4,FLT4,HGF,HIST1H3G,KLF4,MEF2B,MET,NCOR1,NOTCH2,NRAS,NTRK3,PIK3CA,PLCG2,ROS1,SDHB,SOX17,TAP1,TCF7L2	0.086760864	1.322047752

path_1634	AKT1,ALK,ATRX,BBC3,BRCA1,DDR2,ERCC2,ERRFI1,FGFR3,GLI1,H3F3C,HIST1H1C,IKZF1,MAP2K4,MAX,MED12,PIK3CG,RECQL4,RICTOR,RUNX1	0.174614074	1.244336804
path_1635	ACVR1,BRD4,CDK8,CTNNB1,FGF3,HNF1A,ICOSLG,IL7R,KRAS,MRE11A,NBN,NF1,NOTCH1,NOTCH2,PTEN,RAD51B,RBM10,SPEN,STAT5B,SYK	0.199738024	1.254619603
path_1636	AKT1,ARID2,BRCA1,CARM1,CHEK2,CIC,CTNNB1,DAXX,GSK3B,HIST1H3E,MDM2,NKX2-1,PMAIP1,PPP6C,RASA1,RBM10,RHOA,RNF43,SMO,SOX17	0.42011078	1.155503139
path_1637	ARAF,AURKA,AXIN2,BRCA2,CCNE1,ERCC5,FAM175A,FANCC,FGF19,FLT3,GATA3,INHA,NFE2L2,NTRK1,PAK1,PIK3R2,PIK3R3,PPM1D,RHEB,SMAD4	0.221033518	1.189211491
path_1638	AKT2,AKT3,BBC3,DDR2,EP300,FANCA,FYN,GNA11,ICOSLG,INSRR,MRE11A,NTRK1,NTRK2,PDCD1,PIK3R2,PMS2,PPP2R1A,SDHC,SMARCA4,STAG2	0.561973264	1.101852672
path_1639	AKT3,CHEK1,DDR2,DNMT3A,EGFR,FGF4,FOXO1,GNA11,GNAQ,HIST1H3B,HIST1H3C,IGF2,KLF4,NRAS,PIK3C2G,PMAIP1,RAD50,SDHB,SMAD2,STAT3	0.154593489	0.817979706
path_1640	ARID1B,CASP8,CDK12,ERCC5,FBXW7,GPS2,H3F3C,HIST1H3G,MPL,NKX2-1,NRAS,NTRK2,PIK3R1,PIM1,PTEN,SHQ1,SMARCD1,SPOP,SRC,STK40	0.372582622	1.136949939
path_1641	AKT3,AR,AURKA,BCL10,CDKN2A,CHEK2,CIC,EPHA5,FUBP1,IDH1,KDM5C,KLF4,MRE11A,MTOR,PMS1,RAC2,RICTOR,SLX4,SOX9,SYK	0.20195221	1.256317092
path_1642	AXIN2,BAP1,CCND3,CD274,CD79B,CDC73,CIC,ERRFI1,ETV1,GNAQ,H3F3C,HIST1H3E,KEAP1,MAP2K4,MPL,MST1R,NUP93,PMS1,PRDM1,RAD54L	0.729176186	0.937946093
path_1643	ARID1A,CASP8,CD79B,CDC42,CDK6,CDKN2C,EPHB1,FUBP1,GRIN2A,HIST1H3E,HIST1H3G,IGF1R,IRS1,MRE11A,NEGR1,NOTCH3,PIK3CD,PTPRT,RUNX1,SMAD2	0.125904018	1.277795116
path_1644	AKT1,ARAF,ARID5B,BRCA1,BRCA2,BTK,EIF1AX,EIF4A2,ESR1,ETV1,GSK3B,MALT1,NUP93,PMS2,RHEB,SH2B3,SYK,TRAF2,TSHR,YAP1	0.749038554	1.046576734
path_1645	AXIN2,B2M,BCL6,BRCA2,CDKN2A,FOXP1,FYN,GNAQ,GRIN2A,HIST3H3,HLA-A,MITF,MLL2,NUF2,PIK3C2G,RAD50,SDHB,SOX2,TET1,TRAF2	0.257568024	1.208887342
path_1646	BARD1,BCL2L1,BRCA1,CDC42,CDKN1B,ERCC4,FAM175A,FOXO1,H3F3C,HIST1H3C,IKZF1,LATS1,MDM2,MEN1,MET,MGA,MSH3,MYOD1,PIK3C3,PLK2	0.376101094	1.181919282
path_1647	ALK,BMPR1A,BRCA1,BRIP1,CD276,CDK4,ERRFI1,FGFR4,FUBP1,GATA3,HGF,IGF2,MET,MLL2,NF2,NFKBIA,PIK3CA,PREX2,PTPN11,TP53BP1	0.052158617	1.365487869

path_1648	AKT2,BCL10,CARM1,DDR2,EPHB1,FANCC,FUBP1,GLI1,GPS2,HIST1H3E,IRS2,JAK1,KMT2A,LATS2,MCL1,PGR,PHOX2B,POLD1,PPP2R1A,RAD50	0.055756165	1.315585138
path_1649	BCL10,BCL2L11,CD274,CDK4,CDK6,ELF3,FANCC,JAK2,MLL,MYCN,NOTCH3,NUP93,PDGFRA,PIK3R3,RARA,RPTOR,RYBP,SMAD3,SMAD4,STAG2	0.255347449	1.175062146
path_1650	ARID5B,AXIN1,BAP1,CBL,CCNE1,CD276,CENPA,CHEK1,CTCF,ERCC2,ERCC5,ERRFI1,ESR1,FAT1,IDH1,JAK1,JUN,KMT2D,PDGFRA,PLK2	0.45094853	1.112415425
path_1651	AKT1,CCNE1,CDK12,CDKN1B,CSF1R,CXCR4,DNMT1,DNMT3B,E2F3,ELF3,EPHA5,ETV6,FLT1,FOXP1,HIST1H3B,IL10,MTOR,RAF1,RPS6KA4,RTEL1	0.565089619	1.113076496
path_1652	ALK,AMER1,CHEK2,DNMT3A,EGFR,EIF4E,FANCA,FGFR2,HIST1H1C,KEAP1,KMT2A,MEN1,MLL2,NOTCH2,NPM1,PGR,PMS1,PTEN,RAC2,SLX4	0.837339587	1.029743781
path_1653	AKT1,ALOX12B,ATRX,CBL,CDKN1A,DDR2,EPHB1,ERCC4,FLT1,GNAS,HNF1A,KEAP1,MGA,NKX2-1,NPM1,NTRK3,RAD50,SMAD2,SOX9,SRSF2	0.522237873	1.098089841
path_1654	AKT1,ATRX,CCNE1,CDK12,CDKN2C,CSF1R,DNAJB1,ERBB2,ETV1,ETV6,HIST1H1C,IRF4,KIT,MSH2,NTRK1,PAK1,PIK3R1,PPP6C,RICTOR,RPS6KA4	0.964222901	0.993674871
path_1655	ARID5B,CCND2,E2F3,FOXP1,HIST1H3B,KIT,KRAS,MRE11A,MUTYH,NEGR1,NKX2-1,PAX5,PDGFRA,PTEN,RAD51,RBM10,RUNX1,SMAD2,SMARCA4,SOS1	0.079029018	1.27937836
path_1656	APC,BBC3,BRAF,BRIP1,FAT1,GATA1,GNAQ,HIST1H3J,KIT,KRAS,MAP3K13,MEF2B,MRE11A,PARK2,PNRC1,RYBP,SDHA,SH2B3,SOCS1,SOX17	0.05618931	1.321383128
path_1657	ARID1B,DICER1,DIS3,EED,EPHB1,ETV1,FAM175A,FGFR1,HGF,MALT1,MGA,NKX3-1,NTRK2,PDGFRB,PIK3CG,PPP2R1A,RAC2,RAD51D,RHEB,TEK	0.220532065	1.226911284
path_1658	AKT3,ANKRD11,ARID2,BTK,CARD11,DAXX,EGFR,EPHA5,EPHB1,ERRFI1,ESR1,FANCC,GLI1,HIST1H1C,LATS2,MSH3,NRAS,NUF2,NUP93,RECQL4	0.731689474	0.948838734
path_1659	BMPR1A,CD276,CRLF2,EGFR,FOXA1,HIST1H1C,HIST1H3D,KDR,MSH2,NKX2-1,NTRK2,NUP93,PARP1,PIM1,PRDM1,SDHAF2,SF3B1,SH2B3,SMO,TAP2	0.321445461	1.21516045
path_1660	ARID1B,ATRX,AXIN1,BRCA1,CSF1R,DAXX,ERBB3,IL7R,INHA,JAK3,KEAP1,KIT,KMT2A,MLL2,MRE11A,MSH2,NKX3-1,PPP2R1A,RAD21,RNF43	0.205298862	1.200992082
path_1661	AXL,CDKN2C,ERBB3,FGF4,FGFR3,FH,FLT3,IFNGR1,IRS1,MDC1,MEF2B,MLL,MPL,MSH3,NBN,NFE2L2,NOTCH1,PTPRS,RAC2,RUNX1	0.289322039	1.163094266

path_1662	CCND2,CXCR4,DNMT3B,ERBB4,H3F3C,HOXB13,KMT2D,MDM2,MET,MLL3,PAK1,PIK3CG,PLK2,POLD1,PPP6C,PTPRD,SMARCD1,SOX2,TNFAIP3,TSHR	0.296947366	1.171137782
path_1663	B2M,CDK4,CHEK1,CTCF,EPHA5,FLT4,FOXA1,IFNGR1,LATS2,NPM1,PIK3C2G,PMS1,PPP2R1A,RAD50,RAD51B,RAD51D,RBM10,RICTOR,STAG2,TGFBR1	0.406079755	1.138849362
path_1664	ABL1,ARID2,BBC3,BCL2L1,BCOR,CDH1,CDK4,CDKN1A,E2F3,EIF1AX,ELF3,ERCC5,IL10,MEF2B,MRE11A,NRAS,PPP2R1A,RAD51B,SOX17,YAP1	0.362799286	1.138967141
path_1665	BCL2L1,CALR,CASP8,EPHA3,FLT1,HIST1H3I,HIST3H3,IRS2,KIT,MDC1,MDM2,NOTCH4,NTRK2,PAK1,PALB2,PLK2,POLD1,RAD51D,SF3B1,SRSF2	0.995482585	0.999112003
path_1666	ACVR1,ALOX12B,BCL2L11,EGFR,EIF4A2,FAM46C,FANCC,FGF19,GRIN2A,HIST1H3J,IL7R,KLF4,LATS2,MDC1,MTOR,MYOD1,NUP93,PLCG2,SMAD2,STAT5B	0.86887218	0.972385057
path_1667	AKT2,ATM,CDKN1B,DAXX,ERCC5,FBXW7,FOXL2,GNA11,HIST1H3A,HNF1A,MAP3K1,PBRM1,PIK3R1,PMAIP1,PTCH1,SETD2,SOX9,SPEN,SRC,STAG2	0.697351056	1.069322824
path_1668	BCL10,CARD11,CDKN2A,CRLF2,CTLA4,DAXX,ELF3,FH,HIST1H3C,HIST3H3,IL7R,MCL1,MET,MYC,MYCN,NKX2-1,RAD51,RARA,SMAD4,TET1	0.229093127	1.26234572
path_1669	BBC3,BCL2,BCL6,CASP8,CDC73,DNMT3A,EP300,EPHA7,GATA2,GNAS,H3F3C,INHBA,MET,NKX2-1,PARK2,PDGFRB,PIK3CG,PMS1,RNF43,SOX17	0.072929654	1.338874775
path_1670	AKT1,AKT3,ARID5B,AXIN1,BAP1,BCL2L1,BCL6,BTK,CCND1,CEBPA,CSF1R,EGFR,ERBB2,ERCC4,HOXB13,MDM4,PGR,PIK3R3,PNRC1,PTPN11	0.930924445	1.016984708
path_1671	CXCR4,ERBB2,FANCA,FUBP1,GNAQ,IL7R,KNSTRN,MALT1,MAP3K13,MCL1,MED12,MYC,NBN,NOTCH2,NOTCH3,PLK2,RAF1,RHEB,RPS6KB2,SOX17	0.127942225	1.239866423
path_1672	BCL6,BCOR,CALR,CCND3,FAM46C,H3F3C,HIST1H3B,HNF1A,ICOSLG,IGF2,MAP2K4,MDC1,MLL3,MTOR,NKX3-1,NTRK2,PIM1,SDHB,SETD2,SLX4	0.36278553	1.168214813
path_1673	ARAF,BCL10,BLM,ERG,FLT4,GLI1,IGF1R,JAK2,KMT2D,MITF,MLL,MSH2,MTOR,NOTCH1,PAK1,PMS2,RFWD2,SDHB,STK40,TP53	0.72774285	0.951038014
path_1674	APC,ASXL1,BARD1,CREBBP,DAXX,EPHA5,FGF3,FGFR2,FUBP1,HOXB13,IDH1,IL10,KMT2D,NCOA3,NF2,NFE2L2,NSD1,POLD1,RAD51D,RET	0.148689662	1.270256648
path_1675	ALOX12B,AMER1,ARID2,BCL2L1,CASP8,CSF1R,CYSLTR2,FUBP1,GATA3,GPS2,JUN,LATS2,MAP2K1,MLL,MLL3,NKX3-1,PAX5,PNRC1,RUNX1,SOX9	0.966482053	1.007481024

path_1676	AKT1,AURKA,BLM,CD274,CTCF,FAT1,FOXO1,FOXP1,HIST1H3A,HIST1H3B,HIST1H3H,MRE11A,MSH2,MST1,NKX2-1,PIK3C3,PLK2,TAP1,TBX3,TERT	0.971903272	1.006616627
path_1677	ABL1,APC,CARM1,CDK4,CDK8,FGF4,GSK3B,IL10,INPP4A,INSRR,KNSTRN,MAX,MDM4,MLL2,PPM1D,RAD51C,RAD54L,RASA1,RYBP,SOX9	0.052015736	1.31797064
path_1678	BCL2,BRAF,CBL,CD79B,CREBBP,EIF4E,EPHA7,FGFR4,GNAQ,GNAS,ICOSLG,INPP4A,KMT2A,MSH2,NF1,NTRK1,PIK3CB,PIK3CD,RAD50,RAF1	0.102576233	1.33778331
path_1679	BRD4,CDK4,CDKN2A,CSF3R,FOXL2,GPS2,HIST1H1C,JUN,MGA,MLL2,NOTCH2,NUF2,PDGFRB,PGR,PIK3CA,RBM10,RYBP,SDHB,STAT5B,STK11	0.752559414	1.046639598
path_1680	AXL,CBL,CIC,DNMT3A,E2F3,EPHA3,FGFR3,FYN,IKZF1,MAP3K1,MST1R,MUTYH,PAX5,PRDM1,RAC2,RTEL1,SH2B3,SPOP,STK11,TAP2	0.525764171	1.109385173
path_1681	APC,BCL2L1,BRIP1,CDC73,DNMT1,FH,HIST1H3H,HOXB13,KIT,MCL1,NOTCH3,PIM1,RHOA,RNF43,RPTOR,SRC,STAG2,TEK,TERT,XPO1	0.117825234	1.246447262
path_1682	ATR,BCL2L1,BRD4,CDKN1A,CIC,CREBBP,CSF1R,ERCC2,ERRFI1,GLI1,HIST1H3E,HIST1H3H,IRS1,PDCD1,PIK3C3,PTEN,RAF1,RB1,RICTOR,RYBP	0.373960496	1.171864787
path_1683	AKT1,AXIN2,BCL6,BRAF,CCND3,CRLF2,ERCC2,FGF3,FGF4,HIST1H3J,IGF2,IKZF1,KEAP1,NPM1,PGR,PIK3R1,PPP6C,RAD51,SDHAF2,SHQ1	0.615315808	1.089847398
path_1684	BCL2L1,BRIP1,CARM1,CDC42,CDKN1B,CXCR4,DOT1L,ERBB3,GATA1,IKZF1,MAP3K1,MLL2,NCOR1,NEGR1,NOTCH3,PAK1,PAK7,PARP1,PIK3R3,RNF43	0.197479013	1.230657811
path_1685	CARM1,CDH1,CENPA,EPHA3,EZH2,FGF4,HIST1H3E,IKZF1,IL7R,MEF2B,MGA,MPL,MSH2,NF1,RHOA,RUNX1,SDHA,SDHB,SMARCD1,TCF7L2	0.109319797	1.326816184
path_1686	CARM1,CCND1,CREBBP,CTCF,ESR1,FOXO1,HNF1A,IL10,INPP4A,MAX,MEN1,PIK3CD,PPP2R1A,PRDM1,PTPRT,RAD51D,RFWD2,RPS6KB2,SMAD3,TRAF7	0.261657569	1.239418921
path_1687	ARID1A,BMPR1A,BTK,CDK8,CHEK2,CRLF2,ETV6,FGF4,FGFR2,FH,HGF,HIST1H3D,MGA,NEGR1,PAK7,PARP1,PLCG2,SMAD2,SMO,SPOP	0.371546113	1.163775387
path_1688	DOT1L,EGFR,EP300,EPHA5,FAM46C,FANCA,GATA2,GNAQ,HIST1H3E,IRS2,KMT2A,MUTYH,NEGR1,PALB2,PIK3CA,PIK3CG,PTCH1,RUNX1,SDHB,SRC	0.954482199	1.008653893
path_1689	ALOX12B,BARD1,CIC,DIS3,E2F3,FAM46C,GLI1,HIST1H3A,HIST1H3J,ICOSLG,JAK1,LATS1,MDM2,MYCN,NBN,PTPN11,PTPRD,RAD51,RAF1,SMAD2	0.170046416	1.281193741

path_1690	ALK,ANKRD11,BRIP1,ERCC2,FGFR1,HIST3H3,HLA-A,INPP4A,MDM2,MEF2B,MTOR,MYOD1,NCOR1,PAX5,PDGFRB,PIK3CG,PRDM1,RAD51B,RET,SMAD3	0.248632009	1.229452652
path_1691	ARID5B,ASXL1,AXIN2,BCL2L11,BCL6,CENPA,DOT1L,GLI1,GNAS,HIST1H3J,INPP4A,JUN,KDM5A,MDC1,MSH2,MTOR,PIK3C2G,PTPN11,RAD51C,SH2D1A	0.990521197	1.001680245
path_1692	ARAF,BCL2L1,CALR,CDH1,CIC,DICER1,EPHA3,FYN,MYCN,NOTCH1,PIK3CD,PIK3CG,RPS6KA4,SMAD2,SMARCB1,SMO,SRC,SRSF2,TEK,U2AF1	0.081625519	1.336577369
path_1693	ATM,CSF3R,DNMT1,DOT1L,EIF1AX,ERBB4,FANCC,KIT,MAP3K13,MSH2,MYCL1,NOTCH2,PAK1,PIK3C2G,PIK3CD,PIK3R2,PRDM1,SLX4,SPEN,TP63	0.452515726	1.130420588
path_1694	AKT3,BARD1,BCL10,BLM,ELF3,ERRFI1,FUBP1,HGF,HLA-A,IKBKE,KRAS,MAP2K1,MDM4,PARP1,POLD1,RICTOR,SOCS1,SPOP,STAT5B,TNFAIP3	0.237590547	1.211009805
path_1695	AXIN1,BRCA2,BRD4,CDC73,CDK12,CENPA,FAM46C,GATA2,H3F3C,HIST1H3H,HIST1H3I,MAP2K1,MYOD1,NSD1,NUF2,PDCD1,PIK3CB,PNRC1,PTEN,SDHAF2	0.075736307	1.30463923
path_1696	APC,ARID2,ATRX,BRCA2,CIC,ERCC5,FUBP1,KNSTRN,MGA,PAX5,PIK3CB,PLK2,PTCH1,RFWD2,RICTOR,SLX4,SYK,TBX3,TRAF7,TSC1	0.075968977	1.327526972
path_1697	ACVR1,CD79B,CDK4,DNMT3A,EGFR,GATA3,GSK3B,INHBA,IRS1,MLL3,MSH3,MSH6,NUP93,PBRM1,PTEN,PTPRT,RUNX1,SDHC,SLX4,TAP2	0.966714885	1.006218354
path_1698	ARID1A,ATM,CD274,CDH1,CDKN1A,CEBPA,DNMT1,DNMT3A,E2F3,EPHA3,EPHA5,H3F3C,IKZF1,JUN,MDC1,MDM4,MST1,PHOX2B,RBM10,SLX4	0.246616185	1.185668818
path_1699	BCOR,CARD11,CDH1,CDK12,CRLF2,DNAJB1,EPHA7,FAM175A,FANCC,GRIN2A,IL7R,INHA,IRS2,MYCL1,NCOA3,RFWD2,RYBP,SOX2,TGFBR1,WT1	0.662003551	1.063827009
path_1700	ASXL2,BBC3,BCOR,CCND2,CD79B,CDKN2C,FGF3,FOXA1,HIST1H3H,HNF1A,JUN,KEAP1,MUTYH,NCOA3,NEGR1,PGR,POLE,PTPRS,RARA,RPS6KB2	0.07673111	1.322552343
path_1701	ARID5B,BBC3,BTK,CIC,CSF3R,CTCF,EED,FLT4,INHA,MST1,MST1R,NKX3-1,PIK3C2G,PPM1D,RFWD2,RYBP,SH2D1A,SLX4,TAP1,TET2	0.494860253	1.101644054
path_1702	APC,BMPR1A,BRAF,CCNE1,CENPA,DNMT3A,FAM46C,FGF4,HGF,IL10,KIT,MPL,NOTCH1,PIK3CD,POLD1,RAD51,SETD2,SMO,SOX2,STK11	0.053677747	1.329538467
path_1703	ARID5B,ASXL1,AURKA,AXIN1,CARD11,CTNNB1,E2F3,EIF4E,FGF3,FGFR2,FYN,KLF4,MLL,MLL3,NUF2,PIK3CG,RAD51,RHOA,SMAD4,TP63	0.265610548	1.201038047

path_1704	ALOX12B,AXIN1,AXL,CEBPA,CRLF2,ERCC4,ERRFI1,FUBP1,INHBA,KMT2A,NOTCH4,NSD1,REL,RHEB,RNF43,RPS6KA4,RTEL1,SOX9,SPEN,SPOP	0.328992415	1.150699006
path_1705	APC,ARID5B,BBC3,CDK12,FOXP1,GNAQ,KEAP1,MDC1,MET,MYC,NCOA3,PBRM1,PDCD1,PIM1,PLK2,RAD51,RPTOR,SDHAF2,TRAF7,U2AF1	0.781598669	1.042559725
path_1706	ALK,AXL,B2M,BCL2L1,CALR,CREBBP,DNAJB1,EED,EPHA3,GNAQ,GNAS,HIST1H3H,INHBA,INSR,KMT2A,MET,MRE11A,MST1R,PALB2,RECQL4	0.140498771	1.294874934
path_1707	BRCA1,BTK,CDC73,CSF3R,DROSHA,GATA1,GNAS,IL10,INSRR,MGA,NCOA3,PAK7,PIK3CG,PREX2,PTPRT,RAD51D,RICTOR,SOX2,STK11,TRAF7	0.760538758	1.045402026
path_1708	BAP1,CTCF,FAT1,FGFR1,FLT1,FLT3,GATA1,H3F3C,IGF2,IKBKE,INPP4A,KDR,LATS1,MRE11A,PIK3CD,PTPRD,RAD54L,RARA,RB1,RTEL1	0.1912682	1.226450918
path_1709	BRCA2,CSF1R,CSF3R,ERRFI1,FAM46C,FH,HIST1H3H,IDH1,KIT,NBN,NSD1,PDCD1,PHOX2B,PIK3CD,RB1,ROS1,SOX17,SYK,TET2,TOP1	0.237541788	1.182809815
path_1710	ARID1A,ARID1B,CASP8,CBL,CCNE1,CHEK2,CIC,FGF19,HIST1H1C,IRS1,MED12,MET,PIK3R3,PTPRT,RAD51C,RAF1,TGFBR1,TGFBR2,TP53BP1,TP63	0.16235508	1.245595073
path_1711	ASXL2,BRD4,CDH1,EED,EGFR,EIF4E,FBXW7,GSK3B,MRE11A,MSH2,MYOD1,NBN,NCOR1,NPM1,PIK3CA,PIK3CD,PTPRS,RECQL4,RHEB,STAT3	0.133934102	1.310694425
path_1712	ASXL2,BAP1,CDK8,DICER1,FYN,GLI1,GNAQ,HIST1H3B,IDH1,MALT1,MAP3K1,MDM2,NEGR1,NFKBIA,NTRK2,RAD51,RBM10,TGFBR2,TSHR,YAP1	0.459265661	1.110199793
path_1713	AKT3,CDK4,E2F3,EED,FAM46C,GNAQ,HIST1H3G,INPP4B,KRAS,LATS2,NF1,NKX3-1,PPM1D,RAD51C,RAF1,SH2D1A,SOCS1,STAG2,STAT5B,TRAF7	0.050734342	1.34054415
path_1714	ARID2,ATRX,CALR,CREBBP,EED,EPHA7,FLT3,FOXA1,HIST1H1C,HIST1H3A,INSR,IRS2,MYOD1,PIK3R2,RAF1,SMARCA4,SOCS1,TEK,TMPRSS2,TSC1	0.181465983	1.241641616
path_1715	BCL10,CDKN1B,DNMT3A,FGFR1,FH,GATA2,HGF,IGF2,INPP4B,KDM6A,MPL,NFKBIA,PAK7,PTPRD,RAD51,RBM10,RHOA,SPEN,TAP2,TCF3	0.070475058	1.31821859
path_1716	AXL,BCL2L11,CDK6,CTLA4,DDR2,DICER1,DIS3,DNMT3A,FGF4,GSK3B,IRS1,KDM5A,KDM6A,PBRM1,PDGFRA,RBM10,RPS6KA4,SDHB,SMAD4,SOS1	0.676642694	1.067869208
path_1717	AR,ATR,BARD1,BCL6,CALR,DAXX,ERBB2,ESR1,GSK3B,HIST1H3B,IL10,IRS1,MDM2,MLL3,NSD1,PMS1,PTCH1,STAG2,TBX3,WT1	0.121228797	1.285630038
path_1718	B2M,BARD1,BBC3,BTK,CCND2,CDC73,CUL3,FBXW7,KDR,MST1,MYCN,NOTCH3,PARK2,PPP2R1A,RAC2,RBM10,RICTOR,SDHA,SDHB,TP53BP1	0.120550728	1.306510227

path_1719	ASXL1,ATM,ATR,BARD1,CDK12,DNAJB1,DNMT3B,EPHA7,FAM175A,FBXW7,HIST1H1C,IFNGR1,NOTCH3,PALB2,PAX5,PMAIP1,RAD51C,SDHAF2,SUFU,YAP1	0.33301649	1.193807026
path_1720	CALR,CD274,DICER1,ETV1,FGFR3,FOXO1,FYN,GNAQ,GPS2,GSK3B,HIST1H3G,NKX2-1,NTRK1,PALB2,PAX5,PIK3C3,RAD54L,RPS6KA4,RYBP,SMARCD1	0.535113477	1.094637611
path_1721	AMER1,ARID2,CEBPA,ELF3,ERBB3,GLI1,GNA11,H3F3C,INHA,MAP2K1,NOTCH2,NSD1,NUF2,PALB2,PLK2,RECQL4,SRC,TBX3,TEK,TGFBR1	0.657983174	1.084447616
path_1722	ALK,ANKRD11,AXIN1,CARD11,CDK6,CEBPA,FOXA1,GSK3B,IKZF1,INSR,MSH6,NBN,NFE2L2,NKX3-1,PMS1,RAD51B,RAD54L,RAF1,RHEB,TBX3	0.266704622	1.243495491
path_1723	AKT3,AURKA,CALR,CDC73,CIC,DNMT3A,EPHB1,ERBB2,ERCC2,FGFR2,HIST1H3I,HIST3H3,ICOSLG,KMT2A,MAP3K13,MEN1,MSH3,PNRC1,SRSF2,STK40	0.775318847	1.042151677
path_1724	ACVR1,ARID5B,BCL6,CASP8,CD274,CDH1,CHEK2,EED,ERCC4,FGF19,HIST1H3B,IGF2,KDM5A,KLF4,MST1R,NCOA3,PAK1,RAF1,RASA1,TERT	0.238420679	1.192272095
path_1725	BARD1,BCOR,BRIP1,CDK6,CTLA4,ERBB3,ETV6,GATA2,IL10,NF1,NOTCH4,NTRK3,PGR,PIK3CG,PPP2R1A,PTPN11,RAD54L,RAF1,RARA,RBM10	0.276049684	1.176989858
path_1726	ALOX12B,AXIN1,BCOR,DNMT3B,EPHB1,ERBB2,ERBB3,ERCC4,ERG,FBXW7,FLT1,FOXO1,HIST1H3A,ICOSLG,IL7R,INPP4A,KDM5A,MSH3,NTRK3,RHEB	0.764760019	1.043201004
path_1727	ANKRD11,CD274,CD276,CDKN2A,DOT1L,ERG,FGFR2,GATA2,GPS2,ICOSLG,INSRR,KNSTRN,LATS2,NCOR1,NF1,RAD51,SF3B1,SLX4,SRSF2,STAT3	0.096588639	1.357875527
path_1728	CREBBP,CXCR4,ETV1,FANCA,GATA2,HIST1H3D,HLA-A,ICOSLG,INHA,KNSTRN,MCL1,MED12,MST1R,NKX3-1,NOTCH3,RARA,RASA1,STK11,TCF7L2,TGFBR1	0.165068977	1.244075189
path_1729	ACVR1,AMER1,ARID5B,BRIP1,CDK6,CDKN1A,CENPA,ETV1,IKZF1,KLF4,KMT2D,MAP2K1,MET,MGA,MPL,MYC,RARA,SDHB,SMARCB1,TOP1	0.150974964	1.283090212
path_1730	APC,CD276,DDR2,DNMT1,E2F3,EGFR,EPHA5,FLT3,FUBP1,GATA2,HIST1H3C,HNF1A,HOXB13,MAP2K1,MED12,MYOD1,NSD1,RYBP,SDHB,TAP2	0.409201403	1.14873803
path_1731	ATR,AXIN1,CALR,CD274,CDC73,CSF1R,EPHA5,ERRFI1,ESR1,HIST1H3C,KLF4,MCL1,MYC,NPM1,NTRK3,PIK3CB,PLCG2,RBM10,RFWD2,SMAD4	0.666784011	1.071451907
path_1732	BRIP1,CDK12,DAXX,FAM175A,FAT1,FLT4,FOXL2,FOXP1,GNA11,KDM6A,MED12,MST1R,MYOD1,NF1,NOTCH2,PDCD1,PTPRT,RAF1,RASA1,TGFBR2	0.271924219	1.176129036

path_1733	AKT1,CARM1,CD274,CDK6,CDKN1A,CXCR4,FGFR1,FGFR2,FLT3,FYN,HIST1H3C,IDH1,JUN,MTOR,NFKBIA,PIK3CD,RARA,REL,SRC,SRSF2	0.09433792	1.329538214
path_1734	ACVR1,ARAF,ARID1B,BARD1,BCL10,CARD11,CDH1,CSF1R,FUBP1,HIST1H3G,KEAP1,MGA,MYC,NBN,PAK1,PPP2R1A,PTEN,RFWD2,RHOA,SPOP	0.514443335	1.104890366
path_1735	AKT1,ARID1B,BRD4,CD274,CDK4,CHEK2,CIC,CTNNB1,DNMT1,DNMT3A,ETV6,GNA11,HGF,KDM5C,KEAP1,MEN1,NOTCH1,RASA1,RHEB,TEK	0.475328571	1.113502012
path_1736	AXIN2,CCND1,CIC,DICER1,EIF4E,EPHA3,FANCA,FGF4,IL10,INHA,LATS1,MALT1,MAX,MLL3,MYC,RET,ROS1,RPS6KB2,SDHA,SH2B3	0.142946671	1.296185442
path_1737	ARID1B,ASXL1,ATRX,CDK4,CDKN1B,DIS3,DNMT1,EPHA5,ERBB4,HIST1H3G,HIST1H3I,KDM6A,MAP3K13,MRE11A,PRDM1,RECQL4,REL,RPTOR,SRC,TP53BP1	0.378738151	1.155888564
path_1738	ACVR1,AR,ARID2,BCL10,CASP8,CBL,EZH2,FANCA,GATA2,HIST1H3G,INPP4B,MUTYH,PAK1,PALB2,PMS2,RASA1,RTEL1,SDHB,SH2D1A,SMAD2	0.499963796	1.101668883
path_1739	AKT1,ALOX12B,BCL2,DNMT3A,ERBB3,ERCC2,FGFR1,FOXA1,GATA2,HNF1A,INPP4A,INSR,MET,MST1,MYC,NOTCH1,NTRK2,PGR,RUNX1,TP53	0.164015117	1.21778679
path_1740	AR,ARID2,BCL2L11,BCOR,BRD4,CARM1,DNAJB1,FGFR4,FLT3,IGF2,MAP3K1,MITF,MRE11A,MYCL1,NOTCH3,PNRC1,PRDM1,SPOP,TBX3,TERT	0.062670148	1.30459469
path_1741	CDK12,CDKN2A,CXCR4,ETV6,FANCC,FGFR4,FOXP1,HIST1H3I,ICOSLG,IFNGR1,JAK3,KRAS,MAP3K1,MED12,POLE,RASA1,SPOP,STAT5B,TAP2,TET2	0.27608581	1.174426884
path_1742	AKT3,ARID2,BARD1,BCOR,CCND3,CD79B,CTCF,DDR2,FAT1,FGFR3,GSK3B,IRF4,MDC1,MITF,NTRK2,PNRC1,POLD1,POLE,RET,RICTOR	0.652263211	1.072425017
path_1743	ARAF,CENPA,CREBBP,CTNNB1,DROSHA,FGFR2,FOXA1,GPS2,HIST1H1C,HIST1H3H,IGF1R,IL7R,INHBA,KDM6A,MAP2K4,PARK2,PBRM1,RPTOR,RYBP,STAT3	0.613965133	1.102903142
path_1744	ASXL2,BBC3,CALR,DIS3,EGFR,FH,FOXP1,GATA1,GNA11,JUN,MLL,MTOR,MYC,NOTCH2,PPP2R1A,PTCH1,PTPRS,RUNX1,SUFU,TRAF7	0.603326301	1.098626914
path_1745	AKT1,BBC3,BRIP1,CARM1,CDKN1B,DDR2,DNMT3A,GPS2,H3F3C,HOXB13,KMT2A,MAP2K4,MUTYH,RASA1,RECQL4,SHQ1,SMO,TGFBR1,TMPRSS2,TP63	0.126669905	1.244706259
path_1746	ATM,BCL2,CCND1,CCNE1,DNMT3A,ERCC5,GNAS,HGF,MLL2,MST1,PARK2,PPM1D,RARA,REL,RTEL1,SH2D1A,SOCS1,SOX17,SRC,TAP1	0.477347161	1.132929716

path_1747	ATM,BMPR1A,BRIP1,EIF4A2,EZH2,FGFR1,IDH1,IKBKE,INSRR,KIT,MAP2K2,MUTYH,NKX2-1,NOTCH3,PIK3C2G,PIM1,PTEN,RAD51C,TERT,TET1	0.119632774	1.293227685
path_1748	CCND2,CTCF,CYSLTR2,ESR1,GNAS,HIST1H3C,HIST1H3H,JAK2,KMT2A,MAP3K13,MST1,NF1,NF2,RAD51B,RASA1,ROS1,SDHC,SMAD3,SRC,STK40	0.060175509	1.310874827
path_1749	ARID1B,BCL10,BRAF,CDH1,CDKN2A,CEBPA,CSF1R,CTNNB1,E2F3,FGF19,FLT3,IKBKE,NUF2,PDCD1,PIK3CA,RB1,RHEB,ROS1,STAT5B,TAP1	0.053238282	1.397971061
path_1750	BAP1,BARD1,BCOR,BRCA1,BRCA2,CEBPA,CREBBP,DOT1L,ERBB2,ERBB3,FGF19,IKZF1,MAP2K1,MAP3K1,MEF2B,NCOR1,NEGR1,NKX2-1,SH2B3,TAP2	0.780281429	1.048860877
path_1751	BBC3,CHEK1,CTLA4,EGFR,EIF4E,EPHA7,EZH2,FYN,HIST1H3D,MLL3,MRE11A,NF1,NKX2-1,NPM1,NRAS,PIM1,PMS2,SOX17,SPEN,TRAF2	0.904413053	1.019302139
path_1752	AXL,CCND1,CDK6,CDKN2A,CSF3R,FGF3,FGFR1,FGFR4,GNAQ,JAK3,KEAP1,LATS2,MAP3K1,MST1,MUTYH,RARA,RBM10,ROS1,SMARCA4,TCF7L2	0.211998423	1.202845437
path_1753	AXIN1,BCL2,BCL2L11,BMPR1A,CARD11,DICER1,EED,FUBP1,JAK1,KDM5A,KMT2D,MALT1,PALB2,PDGFRA,POLD1,RAD50,SH2D1A,SHQ1,SLX4,TRAF2	0.645578209	1.068762
path_1754	BRIP1,CDKN2A,CTLA4,DNMT3A,ELF3,FGFR3,FOXL2,GLI1,GNAQ,GRIN2A,INPP4A,JAK2,NOTCH2,PDGFRB,RAD51C,RASA1,SRC,TBX3,TCF7L2,TP63	0.357994233	1.162998914
path_1755	BAP1,CARM1,CD276,CTLA4,DNAJB1,ERG,FANCC,FGFR1,HNF1A,IGF1R,IKBKE,IL7R,IRF4,LATS2,MAX,MSH3,PIK3C3,PMS2,RARA,REL	0.082765934	1.278140411
path_1756	ASXL2,AXIN2,CDK4,DOT1L,ELF3,FGFR2,GNA11,IGF2,IRF4,KIT,KLF4,MDC1,MLL2,MLL3,NFE2L2,NKX3-1,PDCD1,PLCG2,POLE,PREX2	0.184655941	1.23362918
path_1757	ASXL2,CCND3,CDC73,CDK12,CENPA,ELF3,ERCC2,ERRFI1,FOXP1,HIST1H3C,IL7R,KDM5C,MST1,NFKBIA,NOTCH3,PARP1,PLCG2,POLE,PTPN11,RAD50	0.438330406	1.118149117
path_1758	ALOX12B,ARAF,BMPR1A,CDK8,DICER1,ERCC2,FGFR4,GPS2,HIST1H3G,JAK3,NKX3-1,NPM1,PARP1,PDCD1,PNRC1,PTPRT,RBM10,RPTOR,SMO,TEK	0.280936977	1.197119036
path_1759	AKT1,BTK,CD276,DICER1,EIF1AX,ERRFI1,ETV1,ETV6,HIST1H1C,HIST3H3,ICOSLG,JAK2,PARK2,PLCG2,RARA,RET,RPTOR,SMAD3,SPOP,TAP1	0.807196535	1.036266141
path_1760	AXIN1,CASP8,CD274,CDKN1B,CHEK1,FUBP1,HOXB13,MEN1,MSH3,NOTCH3,NOTCH4,PAK7,PAX5,PTPRD,RASA1,RYBP,SETD2,SLX4,SPOP,SRSF2	0.142767608	1.276610722

path_1761	ANKRD11,CARD11,CDKN1B,DDR2,DNMT3A,EGFR,EIF4A2,EIF4E,ERBB4,FANCA,IKBKE,KLF4,NEGR1,NTRK1,PALB2,PIK3C2G,PIK3CB,RECQL4,REL,ROS1	0.596563694	1.088621215
path_1762	BCOR,CYSLTR2,ERG,FAM175A,FLT4,GNAS,IDH1,JUN,LATS1,MAP2K2,MET,PDCD1,PIK3C3,PRDM1,SMAD3,SRSF2,TCF3,TGFBR1,TP63,TSHR	0.252418226	1.212026901
path_1763	AKT2,ARID5B,CD79B,CDK4,CTLA4,DNMT3A,ERBB2,GPS2,HIST1H3H,HLA-A,INHA,INPP4A,IRS2,NFE2L2,PALB2,SMO,SRC,TAP1,TCF3,TP53BP1	0.718550926	0.950201762
path_1764	ACVR1,AKT2,CREBBP,DDR2,ERBB3,ESR1,GNA11,HLA-A,IL7R,JUN,MRE11A,MST1R,PIK3C3,PTCH1,PTPRT,RHOA,RNF43,TBX3,TOP1,XPO1	0.336583556	1.180251943
path_1765	AKT1,ATM,AXL,BCL2L1,CIC,DOT1L,EZH2,FH,GATA1,JAK3,JUN,MST1R,MYCN,NTRK2,PAK7,PALB2,PDCD1,PIK3C2G,PIK3C3,PLK2	0.999908411	1.00001951
path_1766	ABL1,BCOR,BMPR1A,CDKN1A,CDKN2C,DNMT3B,ERBB2,HIST1H3B,IL7R,INHA,KMT2A,KMT2D,PHOX2B,PIK3R1,PTPRT,RHOA,SOX2,TEK,TOP1,ZFHX3	0.52959927	1.093267786
path_1767	ARID2,BRIP1,FOXL2,H3F3C,HIST1H3E,KLF4,KRAS,MAP3K13,MST1,MST1R,NSD1,PAK1,PIK3CB,PREX2,PTPRD,RYBP,SMARCA4,SOX2,SPOP,TGFBR2	0.407009085	1.124606325
path_1768	ARID1B,ATR,BARD1,BCL2L11,DDR2,ETV6,FANCC,IGF1R,IL7R,KDM6A,NF2,NRAS,NTRK1,PIK3C3,SDHB,SPEN,SRC,STAT5B,SUFU,TET1	0.405402931	1.150234765
path_1769	ARID1B,ARID5B,BRD4,CDKN1A,DNMT3B,E2F3,EGFR,ERBB2,IGF2,INPP4A,JAK1,MAP2K2,MDM4,MYOD1,PIK3C3,RHOA,RUNX1,SH2B3,SPOP,SUFU	0.089258283	1.361041279
path_1770	ABL1,ANKRD11,ARID1A,CBL,DAXX,ERBB2,ERCC4,FGF3,FGFR3,GRIN2A,HIST1H3B,HIST1H3D,HNF1A,MAP3K1,NOTCH2,NTRK1,PIK3CG,RB1,RET,SMARCB1	0.540997157	1.099947899
path_1771	ALOX12B,ARID1A,AXL,CARD11,CCND1,CDKN2A,DIS3,ERCC4,FANCC,FBXW7,HGF,INSRR,KNSTRN,MED12,NTRK1,PLK2,PMAIP1,RAC2,RPTOR,SMAD2	0.236430443	1.205041806
path_1772	CCND2,CDC73,CTCF,DAXX,DIS3,ERCC5,HIST1H3J,INHA,IRS1,KMT2A,KMT2D,MAP3K1,MCL1,MED12,MYOD1,PIK3CG,PLK2,PRDM1,SDHAF2,SPEN	0.107533174	1.254471608
path_1773	ABL1,ARID1B,B2M,BLM,BRCA2,CEBPA,CREBBP,CSF1R,EPHA5,HGF,KEAP1,KRAS,MAP3K1,MST1R,NFE2L2,PIK3R3,PTEN,PTPRT,STAG2,SYK	0.458325026	1.115330533
path_1774	AXIN1,BARD1,BRCA1,ERBB2,FGFR4,FH,FOXA1,GATA3,KIT,MET,MSH6,MYCL1,NTRK2,PTEN,SDHB,SMARCA4,SOX17,STK11,TET1,TRAF7	0.173116425	1.218832384

path_1775	ARID1B,BCL2L1,EED,ERRFI1,FAM175A,FGFR3,HIST1H3A,INPP4B,INSR,MAP2K2,MDM4,MITF,MPL,NCOR1,NUP93,PGR,RBM10,SF3B1,SMAD4,SMARCA4	0.306436537	1.173584989
path_1776	ARID1B,BBC3,BCL6,CUL3,EPHB1,ERBB3,FLT3,KDM5C,MAP2K4,MRE11A,MSH2,MYCL1,NEGR1,PARK2,PIM1,PTPRT,RAD50,RECQL4,TGFBR1,TMPRSS2	0.138017563	1.303531506
path_1777	CALR,CDK12,CDK4,FYN,HNF1A,HOXB13,JAK2,KDM5C,KLF4,MLL,NOTCH2,NTRK2,PMS1,POLD1,PPP2R1A,RICTOR,ROS1,RYBP,SF3B1,SH2B3	0.117975938	1.247734668
path_1778	ACVR1,ANKRD11,ATR,AURKA,BRIP1,CASP8,CCNE1,CDK12,CDKN2A,CXCR4,FAM175A,GPS2,INPP4B,JAK1,KDR,KRAS,MRE11A,MYOD1,NTRK2,SLX4	0.167910093	1.221226546
path_1779	APC,ARID1A,BCL2L1,CCND1,CCND2,CCND3,CDK4,DDR2,IFNGR1,MED12,MRE11A,NKX2-1,NKX3-1,PARP1,PIK3C2G,RPS6KB2,SLX4,SOX9,STK11,TET2	0.102095527	1.287867928
path_1780	ARAF,CDK4,CDK6,EPHA3,ERG,ETV1,HGF,HLA-A,IRS2,KDR,KNSTRN,MALT1,MLL,PDCD1,PTPRD,RET,SDHB,SETD2,SH2D1A,STAT5B	0.398967165	1.143036119
path_1781	ABL1,B2M,BMPR1A,BRCA2,CHEK1,CTLA4,FOXA1,KNSTRN,MAP2K2,MLL,MST1R,MYCN,NOTCH1,NTRK3,PTPRT,RAD51D,RAD54L,RTEL1,STAT3,TGFBR1	0.37095551	1.135131514
path_1782	ABL1,APC,BRCA1,BRIP1,CBL,CCND1,CEBPA,DICER1,FAM175A,FLT3,FOXL2,HOXB13,IGF1R,JAK1,MED12,NF2,PARK2,SH2B3,SOX2,STK40	0.074968774	1.403008093
path_1783	APC,CCND3,CDKN1A,CENPA,CSF1R,DICER1,EIF1AX,FAM175A,FGF3,FOXA1,HIST1H3D,MDM2,NUF2,RAD51,RET,RHEB,RPTOR,SF3B1,TET1,TOP1	0.091317798	1.276632707
path_1784	ACVR1,ASXL1,BCL2L11,BTK,CASP8,CDKN1A,CDKN2C,ETV6,GNAS,H3F3C,HIST1H3B,IL7R,JAK2,MLL3,MST1R,PARP1,PGR,PPP2R1A,SH2B3,SMARCA4	0.176101349	1.253334535
path_1785	AR,B2M,CBL,CIC,ELF3,FAM46C,GSK3B,IKZF1,KDM5A,KLF4,KNSTRN,MST1R,PAK7,PAX5,POLE,PRDM1,PTPRS,STK40,TCF7L2,TERT	0.064954681	1.298908652
path_1786	CDC73,CDK4,CDKN2A,E2F3,EED,EPHA5,ERCC4,FH,JAK3,MEF2B,MST1R,MYCN,PIK3CA,PMS1,RICTOR,SETD2,SPEN,STAT3,STAT5B,TRAF2	0.31648433	1.180686814
path_1787	AR,ATR,BMPR1A,CDKN1B,CTCF,EED,GRIN2A,IFNGR1,IL7R,KDM6A,MLL3,PAK1,PPM1D,RPS6KA4,SF3B1,SLX4,SMARCB1,STK40,TEK,TRAF2	0.24164957	1.180310786
path_1788	BRAF,CREBBP,CYSLTR2,FANCC,FAT1,FGFR1,FGFR3,GRIN2A,H3F3C,JAK1,JAK3,KDM5C,KNSTRN,MEF2B,NOTCH3,NRAS,PIM1,PTPRT,RAD51C,SH2D1A	0.66095133	1.071168259

path_1789	AKT1,ARID5B,CD274,DNMT3A,EIF1AX,ERRFI1,FOXO1,GSK3B,HIST1H3D,IGF2,IKZF1,KDM5A,MAP2K4,MET,NSD1,PIK3R2,PPP2R1A,RB1,RFWD2,SMAD3	0.065073911	1.40867515
path_1790	AXL,BMPR1A,BRCA1,CDKN1B,CDKN2C,EED,EIF4A2,FANCA,FOXP1,FYN,IKZF1,IL7R,JAK2,MALT1,PDGFRB,PIK3CB,POLD1,RAD51B,RAD51D,SDHC	0.450115533	0.897905081
path_1791	ARID5B,BRD4,CDH1,CTNNB1,EED,ERCC2,FANCC,HIST1H3A,HIST1H3H,IL7R,IRS2,MEF2B,MYCL1,NCOR1,NFE2L2,PIK3R3,RYBP,SH2D1A,TET1,TGFBR1	0.31647356	1.205790573
path_1792	AXIN1,CCNE1,CYSLTR2,EIF4E,ERBB3,ETV6,HIST1H3C,KDR,MAP2K2,MAP2K4,NUF2,PAK1,PIK3CA,PIK3CG,PTCH1,RASA1,SMAD4,SOX17,SPEN,SYK	0.180542229	1.283989743
path_1793	AKT3,BCOR,CBL,CREBBP,EP300,ERBB3,ESR1,FAT1,FOXO1,GATA1,INPP4A,KMT2D,KRAS,LATS1,MDC1,MLL2,MPL,PPP6C,RYBP,SMARCA4	0.248393178	1.207634166
path_1794	ANKRD11,ARID1A,ARID1B,CDH1,DNAJB1,EIF1AX,EIF4A2,ERBB3,ERRFI1,FAT1,IGF1R,JAK1,MEF2B,MLL3,PIK3R3,PNRC1,POLD1,RAD51C,SLX4,SYK	0.130237061	1.278017218
path_1795	APC,AR,CCNE1,CDH1,CIC,FAM175A,FANCC,FGFR3,FGFR4,H3F3C,KMT2D,LATS1,MAP3K13,NOTCH2,PIK3C2G,PIK3CB,PIK3CG,PTPRT,RNF43,STK40	0.063921052	1.350129147
path_1796	ARAF,ATR,AXL,ERRFI1,FANCA,FGF4,HIST1H3C,HIST1H3I,MAX,MST1R,PIK3CG,PIK3R1,PLCG2,SH2D1A,SMAD4,STAT3,SUFU,TRAF2,TSC1,WT1	0.882095588	0.970949924
path_1797	B2M,BRAF,BRIP1,CALR,CDC42,CTNNB1,DNMT1,ELF3,EP300,EZH2,FANCA,FAT1,FOXP1,IKZF1,NFE2L2,NTRK2,PDGFRB,RAC2,SMARCB1,TNFAIP3	0.1081849	1.297154968
path_1798	ABL1,AKT3,APC,BCL6,E2F3,EPHA3,ERBB3,FAM46C,HOXB13,KNSTRN,NKX2-1,NKX3-1,PNRC1,RAD54L,RASA1,RHEB,RPS6KB2,SF3B1,SHQ1,SPOP	0.459799173	1.149961839
path_1799	AMER1,AR,BBC3,CCND2,CIC,DROSHA,EIF4A2,ERG,FGFR3,GLI1,HIST1H3E,IGF2,IRS1,MLL3,PIK3R1,RAD51D,RBM10,RICTOR,SETD2,SMAD2	0.061397234	1.359570057
path_1800	AMER1,B2M,BCL10,BRIP1,CARD11,CDK6,EIF1AX,FBXW7,GRIN2A,IRS2,KDM6A,MAP2K4,NFKBIA,NOTCH2,PIK3CG,RAD21,RBM10,REL,RTEL1,SMARCD1	0.170812784	1.239152831
path_1801	AKT2,AMER1,AR,CALR,CD274,CDK12,CDKN1A,CTCF,CYSLTR2,MAP2K4,MTOR,PHOX2B,PIK3R2,PPP6C,RAD54L,RICTOR,SDHB,SMAD2,SMARCB1,STAG2	0.543541621	1.092725986
path_1802	B2M,CREBBP,CRLF2,CXCR4,DOT1L,E2F3,EPHA3,FBXW7,FOXP1,HIST1H3B,HIST1H3C,HIST1H3D,HIST3H3,KMT2D,MAX,MRE11A,MSH2,PHOX2B,PIK3C2G,RAD51C	0.05536979	1.388911803

path_1803	AMER1,ARID1A,BCL6,BCOR,CD276,CDK12,CYSLTR2,DDR2,FANCC,FAT1,HOXB13,IKZF1,JAK3,MDM2,PLK2,PTCH1,RAD51C,RAD51D,RET,SOX9	0.350414452	1.162306947
path_1804	ASXL1,AXL,BARD1,CARD11,CENPA,DOT1L,FBXW7,FH,FLT1,GATA3,IFNGR1,LATS2,MCL1,MDC1,MLL2,MST1,MUTYH,PAK7,PPM1D,RBM10	0.670369755	1.06524103
path_1805	ACVR1,B2M,CCND1,CDK8,CDKN2C,FUBP1,GSK3B,HIST1H1C,IL7R,IRS2,JUN,KMT2A,KRAS,MAP2K1,MEN1,MSH6,NUP93,PIM1,PREX2,ROS1	0.396512657	1.150297488
path_1806	CCNE1,CDK12,DDR2,ERCC2,FBXW7,ICOSLG,KMT2D,MAP2K1,MUTYH,NCOA3,NPM1,PDCD1,PDGFRB,PIK3R2,PLCG2,PTEN,RAD51C,RARA,ROS1,RPTOR	0.181018019	1.208293687
path_1807	ATM,BCOR,BRCA2,CCND3,CDC73,DICER1,ERBB4,HIST1H3H,HOXB13,ICOSLG,IRS1,KEAP1,KNSTRN,MAP3K13,MEN1,NTRK2,PBRM1,PIK3R2,PREX2,TCF3	0.579206718	0.92262807
path_1808	ATM,CIC,EPHA5,EPHA7,FAT1,GSK3B,IGF2,KEAP1,KNSTRN,LATS2,MAP2K4,MSH2,NSD1,PARK2,PBRM1,PDGFRB,PLK2,STAT3,TAP2,TCF7L2	0.560932408	0.921145534
path_1809	ALOX12B,BCL2L1,CDK4,DNMT1,DNMT3A,EPHA7,ETV1,FGF3,GATA3,IL7R,INSR,KNSTRN,MLL3,PDCD1,PIM1,PTPRS,RHOA,RPTOR,SMARCA4,SRC	0.101796695	1.307710387
path_1810	ATM,ATR,BBC3,CENPA,EIF1AX,EPHA3,GATA3,LATS1,NCOR1,PPM1D,PPP2R1A,RAD50,SH2B3,SLX4,SMAD3,SMARCA4,SPEN,TMPRSS2,TP63,TSC2	0.090399721	1.2913067
path_1811	BCL2,BCL2L11,CENPA,CRLF2,CTNNB1,EIF1AX,FAM175A,FLT4,FYN,GATA3,HIST1H1C,HIST1H3I,MLL3,MPL,NUP93,PRDM1,RAD54L,RB1,SDHAF2,TGFBR1	0.651934213	1.08254481
path_1812	CARM1,CCND2,CCNE1,CDC73,CDKN2C,CYSLTR2,EP300,EPHA7,FH,INHA,JAK3,MED12,NRAS,PDCD1,PIK3R1,RAD51B,RPS6KA4,TCF7L2,TGFBR1,TSC1	0.460029656	1.111258276
path_1813	ALOX12B,ATR,CDK6,CTCF,DNAJB1,EPHA5,ERCC4,FANCA,FAT1,FOXA1,FOXP1,HIST1H3G,IFNGR1,MCL1,PRDM1,RAD50,RUNX1,SDHAF2,SOX2,TP53BP1	0.282092693	1.163448434
path_1814	ASXL2,BAP1,BARD1,BTK,CCNE1,CDK12,EP300,FUBP1,HNF1A,JAK3,KDM6A,KMT2A,LATS2,NBN,NOTCH1,NRAS,PDCD1,RECQL4,RHOA,SF3B1	0.192926778	1.244955074
path_1815	BCL2L1,BTK,CXCR4,EGFR,ERBB4,ETV6,EZH2,FUBP1,HOXB13,IKBKE,KDM6A,KMT2D,MITF,NOTCH2,NTRK1,PDGFRA,PIK3CA,RTEL1,SHQ1,TP53BP1	0.863551186	1.029844095
path_1816	AMER1,ARID1B,CSF3R,DNMT3A,EPHA7,FOXA1,GNAQ,IGF2,INHBA,MDM4,NRAS,NSD1,PALB2,PTCH1,PTEN,RECQL4,SHQ1,STAT5B,TEK,TERT	0.192412243	1.260065333

path_1817	ATM,CALR,CHEK2,DNMT1,EGFR,ERRFI1,H3F3C,INHA,LATS1,NCOA3,NRAS,NTRK3,PNRC1,POLE,RAD51B,RHOA,SH2B3,SLX4,SMAD2,SOX2	0.550358056	1.107545402
path_1818	ASXL1,BCL2L11,CCND1,CD276,DOT1L,EPHA7,ERG,FGF4,FLT1,FOXP1,HIST1H3B,HIST1H3D,INHA,KDM5C,NFE2L2,NPM1,NRAS,PTEN,PTPRD,RB1	0.081241738	1.29374465
path_1819	BTK,CDKN2C,CTLA4,DROSHA,ERBB3,ESR1,FGF3,FGFR2,GLI1,GNAS,GRIN2A,KDR,LATS1,MCL1,MSH6,NRAS,SH2B3,SMARCA4,SPOP,STAT3	0.249526715	1.219176715
path_1820	ASXL1,ATM,BAP1,CCND2,CXCR4,CYSLTR2,DROSHA,EIF4A2,ETV1,GNAS,INHBA,MAP2K1,NEGR1,NF1,PDCD1,PDGFRB,PMAIP1,RAD50,RYBP,SDHB	0.888213542	1.02321323
path_1821	AKT3,ARID1B,ATM,CTCF,CTNNB1,DICER1,FLT1,GATA1,IGF2,IL10,IRS2,JUN,MCL1,MLL2,NBN,NF1,NUP93,RAD21,RET,RFWD2	0.348744127	0.86625106
path_1822	ATRX,CCND3,CD79B,CTNNB1,EPHB1,ERCC2,FOXL2,FUBP1,KLF4,NKX2-1,PARP1,PIM1,PTPN11,RAD51C,RPS6KA4,SH2B3,SMAD3,SOX17,TCF3,TEK	0.304759878	1.15919217
path_1823	BTK,CD79B,CDH1,CTLA4,EPHA5,ERBB4,FLT1,FYN,IGF2,JAK1,KDM5A,MDM2,MEN1,NFE2L2,NSD1,PARK2,PIK3R2,RHEB,SF3B1,SMO	0.121288561	1.284587908
path_1824	ASXL1,AURKA,AXL,BRCA1,CSF1R,CXCR4,ERRFI1,ESR1,ETV6,HIST1H3A,HIST1H3B,IL10,MAP3K13,MRE11A,PARK2,PIK3R2,PIK3R3,PLCG2,SOX17,SYK	0.792894273	1.03777179
path_1825	AKT2,ALOX12B,ANKRD11,CRLF2,CTNNB1,ERCC5,FBXW7,FGFR1,FOXO1,KLF4,KMT2A,MITF,MSH3,NFE2L2,NKX2-1,PPP2R1A,RYBP,SF3B1,SMARCD1,SOX9	0.129369457	1.24402859
path_1826	ARID1B,BRIP1,CDC42,CEBPA,CTNNB1,EED,FBXW7,FH,IDH1,INPP4A,MSH3,MSH6,PIK3R2,POLE,RAD50,RAD54L,RAF1,RARA,SH2B3,SMAD4	0.398868932	1.126649399
path_1827	AKT1,AMER1,ASXL2,ATM,BBC3,FLT1,FOXP1,GNAS,HGF,HIST1H3H,IL10,LATS1,MCL1,MLL2,MPL,NF2,REL,SMAD2,SUFU,TBX3	0.16074518	1.244928819
path_1828	ARAF,BBC3,CALR,CCND3,CENPA,ERBB3,GLI1,GNA11,GNAS,HIST1H3B,IFNGR1,INPP4B,MALT1,MITF,PAX5,PDGFRA,PTPRD,PTPRT,RAD54L,REL	0.397951367	1.148283371
path_1829	ARAF,ASXL1,AXIN1,BLM,CCNE1,CHEK1,CTNNB1,EPHA3,ERG,EZH2,MRE11A,MUTYH,MYCN,NPM1,RAD21,REL,SDHA,SMARCA4,SMO,TMPRSS2	0.146897511	1.278644166
path_1830	BMPR1A,CALR,CXCR4,DROSHA,E2F3,EIF4E,GNAQ,H3F3C,INHBA,MITF,MSH2,MSH6,NEGR1,NFE2L2,NRAS,PIK3CD,PIK3R3,PNRC1,SMARCB1,TAP1	0.113905045	1.258131536
path_1831	ALOX12B,ARID5B,EIF4A2,ERCC2,ESR1,FGFR1,FLT1,HIST1H3G,HIST1H3I,KDR,MAP3K13,NF1,NTRK2,PALB2,RAD21,RARA,RBM10,RPS6KB2,SH2B3,SOCS1	0.841387726	1.03274202

path_1832	ABL1,CDC73,CREBBP,CRLF2,CTCF,CYSLTR2,FGF4,FOXL2,GNAQ,HIST1H3A,ICOSLG,MALT1,MCL1,MST1,PLCG2,PPP2R1A,PTPRS,RB1,RNF43,STK11	0.275431446	1.210802225
path_1833	ARID1A,CALR,CDC42,CDKN2C,CXCR4,DROSHA,FGFR1,FH,GNAS,HIST1H3E,IRS2,JAK1,JAK2,MCL1,MYOD1,NKX3-1,PTPRD,RAD51D,RHEB,RYBP	0.363478032	1.136788522
path_1834	ALK,ANKRD11,ARID1A,CDKN1B,CENPA,CHEK2,EPHA7,EPHB1,FAM175A,GATA3,HIST1H3C,HIST3H3,HNF1A,IDH1,KMT2D,MDM2,NF2,NKX2-1,PIK3R2,RB1	0.20925314	1.224418205
path_1835	BARD1,BCL2L11,BRCA2,CCNE1,CD274,CD276,EPHB1,ERBB4,ERCC4,ETV1,FAM46C,HIST1H3B,HIST1H3E,HLA-A,KDM5A,PDGFRA,PMS1,RFWD2,RHEB,SH2B3	0.236076408	1.184592423
path_1836	AKT2,APC,DROSHA,INSRR,IRS1,IRS2,KMT2A,MAP2K2,MPL,NCOA3,NRAS,PAX5,PIK3R1,RAD51B,RBM10,RNF43,STAT5B,STK40,SUFU,TET2	0.850583224	1.034695045
path_1837	AKT2,AMER1,ARAF,BRD4,CASP8,CCNE1,FBXW7,MITF,MYCL1,MYCN,PAK1,PGR,PIK3CD,RNF43,RPS6KB2,SETD2,SRC,TAP2,TBX3,TCF7L2	0.115882892	1.24997531
path_1838	ARID1B,ATR,AXIN1,CALR,CASP8,CIC,DNMT1,EIF4A2,FGFR2,GLI1,MDM4,MLL3,NEGR1,NFKBIA,PARP1,PIM1,SOX17,STK11,TCF7L2,TGFBR2	0.343118062	1.166423892
path_1839	AXIN1,BRCA1,CDC73,CEBPA,CENPA,CYSLTR2,EGFR,HIST1H3G,MLL2,MPL,NCOR1,NKX3-1,NOTCH2,NOTCH4,PBRM1,PIK3CD,PMS1,RAD51D,SDHB,TMPRSS2	0.662262302	0.933473777
path_1840	ARID1A,BCL2L11,BLM,CTLA4,FAT1,FGF4,GPS2,GRIN2A,HIST1H3E,MITF,MLL,MSH3,NPM1,PHOX2B,PNRC1,PTPRS,RARA,RPTOR,SLX4,SOX17	0.195783023	1.252038703
path_1841	ACVR1,AXL,BARD1,BCOR,BTK,CHEK2,EPHA5,FAM175A,GNA11,GNAQ,GNAS,MAX,PHOX2B,PIK3R1,PLK2,PMAIP1,RBM10,SMAD4,SMARCA4,SOS1	0.514540213	0.907617937
path_1842	ABL1,AKT1,ANKRD11,ARAF,ASXL2,CD79B,DNMT3B,DROSHA,EED,GPS2,HIST1H3I,KNSTRN,PAK7,PARK2,PAX5,POLD1,RAD51D,SDHB,SMARCB1,SOX17	0.42051799	1.121417325
path_1843	ARID5B,BCL2,BCOR,CENPA,E2F3,EIF4A2,FAM46C,FGF19,FGFR2,FOXO1,HIST1H3A,KEAP1,MUTYH,NKX3-1,NTRK2,POLD1,RAD50,RAD51B,RET,SF3B1	0.272967626	1.195564971
path_1844	AXL,CTCF,DAXX,FAM46C,FYN,HNF1A,IRF4,JAK1,KNSTRN,NOTCH1,PDCD1,PIK3CG,PIK3R2,RUNX1,RYBP,SETD2,SOX9,STAT5B,STK11,SYK	0.885481608	0.977596052
path_1845	AXIN2,AXL,BCL6,EIF4A2,ETV6,FGFR2,FGFR4,GATA2,INSR,IRF4,JAK1,MYCL1,NOTCH1,NOTCH3,PARK2,PIK3C3,PIK3R2,PNRC1,PPP2R1A,SMARCB1	0.170985422	1.287057705

path_1846	ATR,BCL2,BRAF,BTK,CCND1,CEBPA,CHEK1,DNMT3B,EPHA5,ERG,GATA3,HIST1H3J,ICOSLG,INSR,MST1R,PIM1,PMS2,RAD51B,RECQL4,RPS6KB2	0.233636593	1.23203779
path_1847	AXL,CD276,CRLF2,EIF4A2,ERBB3,ERCC2,FGFR4,FOXA1,GSK3B,HIST1H3D,HIST1H3H,HIST1H3I,IDH1,NUP93,PIK3CG,PTCH1,PTEN,RAD21,SHQ1,TEK	0.12143039	1.253802064
path_1848	CCND3,CDH1,CEBPA,CENPA,CSF3R,DIS3,DNMT1,DROSHA,EIF4A2,EPHA5,ERBB2,FGF19,KDM5A,MALT1,NTRK2,NUF2,PALB2,RAC2,RHOA,SMO	0.071249015	1.437629352
path_1849	ARID1B,BLM,CDKN2C,DIS3,ERRFI1,GSK3B,H3F3C,HIST1H1C,IDH1,INHA,MAP2K4,MAP3K13,MEF2B,MST1,POLE,PTCH1,PTPRD,SDHB,SMARCA4,SYK	0.441027665	1.126658122
path_1850	ACVR1,APC,BMPR1A,CDC42,CSF3R,CTCF,DICER1,GATA3,GLI1,HIST1H1C,HIST1H3H,HIST1H3I,HIST3H3,HNF1A,KIT,KMT2A,MCL1,POLE,SDHAF2,SMAD2	0.976398307	1.00419561
path_1851	ARID1A,BMPR1A,ETV6,FLT4,JAK3,KLF4,KMT2A,KNSTRN,MEF2B,MGA,MYCL1,NUF2,PBRM1,POLE,RHEB,RNF43,RPS6KA4,SDHAF2,SMARCA4,SRC	0.085254201	1.309681112
path_1852	ALOX12B,BTK,E2F3,ERBB3,ERG,ESR1,EZH2,KDM5A,KMT2A,MAP3K1,MEN1,NBN,NF1,PBRM1,PIM1,PMS2,PNRC1,RPS6KA4,TP63,TRAF2	0.353451984	1.185064376
path_1853	ASXL1,AXL,BARD1,CTCF,E2F3,EIF1AX,IDH1,IRS1,JAK1,MALT1,MDM2,MEN1,NSD1,NUF2,PLK2,PTCH1,RAD51,RASA1,SDHC,SYK	0.868594632	0.976772265
path_1854	AKT1,ALK,ANKRD11,ARID1B,ATRX,BARD1,CARM1,CCND3,CENPA,CIC,DIS3,DNAJB1,GNA11,INPP4B,JAK3,LATS2,MYCL1,NOTCH2,RAC2,RAD54L	0.116196932	1.344852319
path_1855	ABL1,APC,BCL10,BCL2,CARD11,CTNNB1,DOT1L,EIF4A2,HIST1H3G,IGF2,INSRR,MALT1,MDM4,MLL3,NOTCH2,PHOX2B,PIK3CD,RAF1,SOCS1,TET1	0.101018457	1.336234852
path_1856	APC,BRCA1,CCND2,DROSHA,ESR1,ETV6,FGF4,FGFR4,FLT1,HIST1H3D,JAK3,NTRK2,NUF2,PBRM1,PHOX2B,PIK3CD,PIK3R3,RAD21,RPS6KB2,SMO	0.264451823	1.228952898
path_1857	ALOX12B,ATR,CASP8,CENPA,ETV1,FBXW7,FH,FLT4,GATA3,GNA11,MAP3K1,MDC1,PALB2,PTPN11,RAF1,SMAD4,SOCS1,SOX2,STAT5B,SUFU	0.11000891	1.351042027
path_1858	ASXL2,CENPA,CTNNB1,CUL3,FLT4,FYN,IL10,MRE11A,MST1R,NKX2-1,NPM1,PDGFRB,PIK3C3,PIK3CA,PPP6C,RAD51B,SDHB,SLX4,SMARCB1,TCF7L2	0.056056509	1.420596653
path_1859	ABL1,ASXL1,DNAJB1,FAM46C,GNA11,GSK3B,INHA,INSR,MDC1,MUTYH,NSD1,RAD50,RAD54L,RARA,RET,RHOA,SMAD3,SOX17,SOX9,TET2	0.595289845	0.910790032

path_1860	ANKRD11,CALR,CASP8,DNMT1,ERBB3,ETV6,FAM46C,FOXL2,GNAS,HIST3H3,HLA-A,ICOSLG,IL7R,LATS1,MUTYH,NOTCH1,NTRK1,SPEN,STAT5B,TP63	0.052435042	1.314943551
path_1861	AKT2,B2M,BAP1,BRCA1,ERBB3,ETV6,FH,FUBP1,GPS2,HIST1H3B,IL10,MAP2K4,NOTCH1,NTRK3,PAX5,PIK3C2G,PIK3CA,RAD51D,RHEB,SDHC	0.138719908	1.336857035
path_1862	ACVR1,ARID1A,ASXL2,ATM,BCL2L1,CBL,DNMT1,DROSHA,EIF1AX,GNA11,HIST1H3B,KLF4,MDC1,MLL,MLL3,MPL,NCOA3,NF2,PDCD1,SDHB	0.118192002	1.28280131
path_1863	ARID1B,ATRX,CARD11,CSF3R,DNMT1,GRIN2A,HIST1H3C,HIST1H3I,INSRR,LATS2,MYCL1,NKX3-1,NOTCH4,PALB2,PDGFRA,SF3B1,SH2B3,SOX9,TRAF7,TSC2	0.315714375	1.183868047
path_1864	ALOX12B,BARD1,BLM,CBL,CHEK2,CTNNB1,EGFR,ERBB2,HIST1H3A,JAK1,KNSTRN,MET,PTPN11,RAC2,RAD51,RPTOR,RYBP,SMARCB1,SPEN,TP53	0.38171433	1.165870952
path_1865	ALK,BARD1,CDC42,CTNNB1,EED,EGFR,ETV6,FAT1,FGFR4,FLT3,IGF2,NCOR1,NOTCH2,PARP1,PMAIP1,RAD51,RARA,RHOA,SDHA,TAP2	0.067470158	1.346816863
path_1866	AXL,BCL10,BCL2,CD276,CSF3R,CTLA4,CUL3,EPHA5,ERRFI1,ESR1,FLT3,GPS2,KEAP1,KNSTRN,MAP2K2,MAX,MED12,MLL2,NPM1,PPP6C	0.343391179	1.150595055
path_1867	AXL,BBC3,BCL10,BRD4,CALR,CD79B,CTLA4,ERCC2,ERCC5,GLI1,GNAQ,IKZF1,MAP2K1,MCL1,MDM2,MLL2,PALB2,RPS6KA4,SMO,STAT3	0.982610051	1.003100142
path_1868	AR,CDK8,CEBPA,FOXP1,FUBP1,HOXB13,IGF2,MAP2K2,RPTOR,RYBP,SDHA,SDHAF2,SOX17,SPOP,STAG2,STAT5B,TGFBR2,TP63,XPO1,ZFHX3	0.785064997	1.03975139
path_1869	ARID1A,B2M,BCL6,BRD4,CDC73,CDH1,CDK8,DICER1,ETV6,EZH2,GRIN2A,ICOSLG,IGF1R,IRF4,MST1,NEGR1,NOTCH3,NRAS,SOX2,TAP1	0.369946199	1.135840518
path_1870	ANKRD11,ASXL2,CCND1,CDC73,ERBB3,FANCC,FOXP1,HIST1H3A,INHA,KDM5A,KDM6A,KMT2D,NTRK3,NUF2,PARK2,PIK3C3,PIK3CB,PIK3R3,RECQL4,RET	0.104134491	1.259769359
path_1871	CCND3,CDK4,CSF1R,EPHA5,FOXO1,IGF2,INSR,MEF2B,MEN1,MITF,NCOR1,PDGFRB,PIK3CB,PPP2R1A,RAD50,RAD51,RPS6KB2,SH2D1A,SLX4,STAG2	0.930322926	0.987729191
path_1872	AKT2,ARID2,AXL,B2M,BCOR,EIF4A2,FOXA1,HIST1H3A,HIST1H3H,MALT1,MDC1,MDM4,MLL,NFKBIA,NTRK1,SDHA,SMAD4,SMO,SOCS1,TERT	0.18387821	1.254988769
path_1873	ALOX12B,BARD1,BCL6,CDKN1B,CTCF,DICER1,ERG,ETV1,GNA11,GNAS,IKZF1,MSH6,MST1,MYCL1,PDGFRB,PIK3CD,PTPRS,RICTOR,ROS1,RTEL1	0.112202616	1.261272526

path_1874	AXIN2,AXL,BCOR,EPHB1,ERBB2,HIST1H1C,HIST1H3J,HIST3H3,INSRR,JAK1,JAK3,KLF4,MAP2K4,MRE11A,MSH6,NCOR1,PMS2,PTCH1,RAC2,RAD54L	0.392876575	1.128604592
path_1875	AKT1,BRIP1,CASP8,CRLF2,DNMT1,EED,HIST1H3E,INPP4A,INSR,KDM5A,MAP2K2,NUP93,PALB2,PIK3C2G,PMAIP1,PTCH1,PTEN,RAD50,SH2D1A,STK40	0.410683218	1.123123125
path_1876	AKT3,E2F3,EPHB1,ESR1,FAM175A,GNAQ,GNAS,JAK2,KRAS,MRE11A,NCOA3,NFKBIA,PDGFRA,PLCG2,PPP6C,REL,RPTOR,SOX9,SPEN,STK11	0.447183891	1.11327919
path_1877	CALR,CD274,CDK4,CHEK2,CRLF2,DNMT3B,EPHB1,FOXP1,GLI1,GRIN2A,HIST1H3J,ICOSLG,MAP3K1,MEN1,MPL,PDGFRB,RUNX1,SOX17,SPOP,XPO1	0.83375111	1.03067637
path_1878	AKT3,ERBB2,ETV6,GNAQ,GPS2,INPP4A,KIT,MRE11A,NCOA3,NTRK2,PMS1,PREX2,PTPN11,RYBP,SDHAF2,SHQ1,SMARCA4,SOS1,SOX9,XPO1	0.279827396	1.164878432
path_1879	ARAF,ARID5B,CREBBP,CSF1R,CSF3R,CUL3,EPHA3,EPHA7,ERBB4,ETV6,FOXP1,KRAS,MAP2K4,NCOR1,NTRK1,PGR,RARA,RYBP,SMO,SOX9	0.10400376	1.259474664
path_1880	ASXL2,BCOR,CCNE1,CDH1,DNMT3A,ELF3,ERCC4,ESR1,FAM175A,FGFR4,HIST1H1C,INPP4B,IRF4,LATS1,PGR,SOX17,SRSF2,STAG2,STAT3,STK11	0.176886141	1.225447261
path_1881	AKT1,CDKN2C,ERCC4,ESR1,HIST1H3A,HLA-A,KIT,KMT2A,KNSTRN,MAP2K4,MAX,MEN1,MLL3,MSH3,NKX2-1,PDGFRB,PNRC1,PTPRD,RHOA,SRSF2	0.595023877	1.088315712
path_1882	ATM,BBC3,CDK12,CDKN1B,EGFR,ERRFI1,FLT4,FOXL2,IFNGR1,JAK2,KNSTRN,MEN1,MLL3,PBRM1,PTEN,PTPRD,RAC2,RYBP,SDHC,SOS1	0.468822653	1.113344757
path_1883	BRCA2,CD274,CDK4,DNMT1,EGFR,EPHB1,ERCC2,FANCC,GNAQ,HIST1H3B,HIST1H3E,HIST1H3J,INPP4A,KIT,MEF2B,MSH3,NFKBIA,PDCD1,POLE,SMARCB1	0.146203467	1.324770271
path_1884	ABL1,AKT1,BRCA2,CALR,CBL,CUL3,EIF4E,ERCC2,GATA3,INSR,MDC1,MEF2B,MYC,NTRK2,PALB2,PDGFRA,PIM1,PLK2,RAD21,SOX9	0.680488818	0.928207229
path_1885	AKT1,CDK6,CSF1R,DNAJB1,DNMT3B,ERBB4,FANCA,FYN,GATA2,HIST1H3C,HIST1H3E,IRS2,LATS2,MDC1,MRE11A,MYOD1,PTPRD,RAD51,RASA1,ROS1	0.18266347	1.244411715
path_1886	BTK,CDK8,CDKN1B,CYSLTR2,FGFR3,FGFR4,FLT4,GLI1,HOXB13,JAK1,MPL,MSH6,PAK1,PPP2R1A,RECQL4,REL,RPTOR,SMARCB1,SOX2,TP53	0.521518529	1.101573483
path_1887	BCOR,CDK8,FAM175A,FANCC,FGF19,FOXL2,HIST1H3I,IRF4,MYOD1,PAK7,PARP1,PIK3CD,PPP6C,RAD21,RNF43,SDHC,SUFU,TCF3,TNFAIP3,U2AF1	0.618098996	1.074537916

path_1888	CCND1,CHEK2,CXCR4,DOT1L,EPHA7,ERBB4,FGFR3,GRIN2A,HGF,HIST3H3,MDC1,NOTCH3,PAX5,PBRM1,PIK3C3,PPM1D,PREX2,RICTOR,SDHA,STK40	0.098581476	1.283096692
path_1889	ANKRD11,ARID5B,BTK,FANCA,FGFR2,HIST1H1C,HIST1H3A,HIST1H3H,INSRR,KDR,MAP2K2,MAX,MCL1,MDM2,MST1R,NF1,PARP1,POLE,RAC2,STAG2	0.154984697	1.267085002
path_1890	ALOX12B,ARID1A,ASXL1,BAP1,BCL10,BCL2L11,BLM,CDK12,HIST1H3E,ICOSLG,NEGR1,NFE2L2,NKX2-1,PTEN,PTPRT,RAC2,RAD50,SOX9,SRC,TET1	0.104924107	1.296251615
path_1891	AXL,CBL,CCND1,CD274,CD79B,CDK8,CSF1R,ERG,INSRR,KLF4,MAP3K13,MRE11A,MSH6,PPP6C,RAD51B,RAD54L,RNF43,SH2B3,SOS1,TET2	0.481548644	0.899872592
path_1892	ARID5B,BARD1,CDK12,CRLF2,CSF3R,HIST1H3H,IKBKE,JAK1,KIT,KMT2D,MAP2K2,MAP2K4,MLL2,MPL,MYC,PTCH1,RAD50,RPTOR,SLX4,SMAD2	0.799990438	0.964815982
path_1893	ALOX12B,ARID2,ATM,ATR,BTK,CARM1,CHEK2,EZH2,FLT4,IKBKE,INHA,MLL2,NOTCH3,PLCG2,PLK2,PMS2,RHOA,SMO,TGFBR2,TRAF2	0.987566643	1.002418114
path_1894	ARID2,ASXL1,AXIN1,BCL2L1,BRCA2,EIF4A2,ERCC4,FUBP1,IL7R,KDM5C,LATS1,NF2,NTRK2,PDGFRA,PIM1,PLCG2,RARA,RHOA,RTEL1,SDHAF2	0.145519279	1.278944943
path_1895	CCNE1,CSF3R,FGFR4,GRIN2A,JAK1,KMT2A,MLL3,NEGR1,NKX3-1,NOTCH3,PARK2,PIK3R2,PNRC1,PPM1D,PREX2,PTCH1,SDHAF2,STAT5B,TGFBR2,TP53	0.328527931	1.149009914
path_1896	BCL10,CARD11,CBL,CCND2,CD276,CDK4,EPHA3,ERCC4,ETV6,GATA1,IDH1,KDM5A,MST1,MYCN,NSD1,NUP93,PAK7,PIK3CG,RASA1,ROS1	0.063685006	1.361720066
path_1897	AXIN2,BCL2L11,CCND1,CD274,CENPA,EIF4A2,FGFR1,FOXO1,FUBP1,GLI1,HGF,HOXB13,IFNGR1,KEAP1,PHOX2B,PIK3C2G,PRDM1,PTPRD,RET,SF3B1	0.443472682	1.120011044
path_1898	ALK,APC,ARID1B,BARD1,BCOR,CDKN2C,FGF4,HIST1H3A,HIST1H3G,HIST3H3,HOXB13,INHA,MITF,NEGR1,NTRK2,PALB2,PPP2R1A,PPP6C,SMARCA4,TGFBR2	0.84644795	1.032762053
path_1899	DAXX,EED,ERCC4,FBXW7,GNA11,HIST1H3C,IFNGR1,IL7R,IRS1,KMT2A,NBN,NF2,PAK7,PGR,PMS2,POLD1,PPM1D,PTEN,RHOA,SOCS1	0.413158702	1.12255325
path_1900	BAP1,BARD1,BRD4,CCND1,CDK4,CREBBP,DOT1L,FOXO1,IFNGR1,INHBA,KLF4,MDC1,MET,MITF,NBN,PMS1,RAD51,SH2D1A,SMAD4,TRAF2	0.116634332	1.307422982
path_1901	AXIN2,CDC42,CDC73,CIC,CSF1R,FANCC,FAT1,FOXP1,INPP4B,JAK2,KDR,NKX3-1,NSD1,NUF2,PLCG2,PLK2,PREX2,RYBP,SMARCA4,SMARCD1	0.145137846	1.268490913

path_1902	ARID2,CARD11,CDK4,CDK6,ETV6,EZH2,FGF19,FGFR2,FLT1,GPS2,HIST1H3C,HLA-A,NCOR1,PARP1,PDCD1,PMAIP1,POLD1,RPS6KA4,SHQ1,SMAD2	0.403395539	1.125362473
path_1903	ARID1A,BCL2L1,BCL6,CDK8,CTLA4,DNMT3A,GATA1,GATA3,HIST1H3J,IKZF1,NOTCH3,NOTCH4,NTRK2,PAK1,PDGFRA,PRDM1,RAD50,SDHAF2,SUFU,TBX3	0.802226363	1.043406409
path_1904	BCOR,EIF4A2,ERRFI1,FGFR2,FOXO1,IRF4,KNSTRN,MDM2,MEF2B,MPL,MTOR,NSD1,PPP2R1A,RARA,RHEB,ROS1,SH2D1A,SHQ1,SRSF2,YAP1	0.374189939	1.136284742
path_1905	BAP1,BLM,BRCA1,CARD11,CCND1,CRLF2,DIS3,DNAJB1,FAM175A,FANCA,GLI1,KDM5A,MRE11A,MSH2,NOTCH4,PMS2,RAD50,RARA,RNF43,TCF3	0.776857369	0.960770699
path_1906	ARID5B,BLM,BTK,CDH1,CDKN1A,DNMT3B,E2F3,ERBB2,GNAQ,GSK3B,IRF4,JAK2,MYCL1,NFE2L2,PARP1,PDGFRB,PIK3R3,PPP2R1A,SUFU,WT1	0.146095345	1.300313861
path_1907	ACVR1,BCOR,BRIP1,CDKN1A,EED,EPHA7,ERCC4,FGF19,FGF3,HLA-A,KDM5C,MAP3K13,NF2,NRAS,PIK3C3,PLCG2,PPM1D,RB1,SOX17,SPOP	0.181592333	1.213554323
path_1908	AURKA,BCL10,BCL2,BCL2L11,CDKN2C,CTCF,CTLA4,DIS3,EIF1AX,ERBB3,FAT1,GATA2,GPS2,HIST1H3E,IFNGR1,INSR,MET,MYCL1,SYK,TAP1	0.681508023	1.059618042
path_1909	BRCA1,CREBBP,ESR1,ETV6,FH,GATA2,HIST3H3,IGF2,IL7R,INSRR,KNSTRN,MAP2K4,MYCN,PDGFRA,REL,RPTOR,RUNX1,SPOP,TP53BP1,TRAF2	0.669290747	1.062870572
path_1910	BCOR,CRLF2,DAXX,EIF4A2,FANCA,FOXO1,IGF2,KMT2A,KNSTRN,MLL2,PDCD1,PHOX2B,PIK3R1,PLCG2,PLK2,RAD51B,SHQ1,SOCS1,SUFU,TOP1	0.895365942	1.018958823
path_1911	ALK,ATM,AXIN2,CDK8,EGFR,ERCC2,ERCC5,HIST1H1C,HOXB13,IDH1,JAK2,MCL1,NTRK3,PIK3C2G,PPP6C,PTEN,PTPN11,RECQL4,RPTOR,SUFU	0.524426172	1.107510364
path_1912	CDK6,EGFR,EPHA3,ETV1,FGFR3,FLT1,FOXP1,GATA2,GPS2,HIST3H3,IL10,JAK3,MPL,MUTYH,NEGR1,PIK3C3,PIK3CD,PIK3R3,PPP6C,RET	0.590008782	1.091565834
path_1913	AKT1,CARD11,CBL,CDK8,CDKN2C,CENPA,EP300,EPHA5,EPHB1,FLT1,GATA3,LATS1,MPL,MUTYH,NEGR1,PMAIP1,PRDM1,SDHA,SDHC,SOX17	0.361155473	1.169048002
path_1914	ASXL2,ATR,CENPA,CSF1R,DIS3,EIF4A2,ELF3,EPHB1,FGF19,IDH1,NTRK2,PIK3CD,PMAIP1,RAD51C,RPS6KB2,SETD2,SMAD2,SMAD4,SOS1,SOX2	0.375040387	1.13522915
path_1915	DNMT3A,DOT1L,FGFR3,FUBP1,INHA,INSR,IRS2,MAP3K1,MDM4,MLL2,MSH2,NF1,NRAS,NUF2,RAC2,RBM10,RECQL4,SDHA,SF3B1,SHQ1	0.668736491	1.067352727

path_1916	ATM,ATRX,CDC73,EIF1AX,EIF4A2,FAM175A,FLT3,HIST1H3D,IKZF1,JAK3,MCL1,MLL,MSH6,NTRK1,PDCD1,POLD1,PTCH1,RAD51,SF3B1,SOS1	0.063866043	1.390976195
path_1917	ACVR1,CARM1,CUL3,DNAJB1,DNMT3B,ERRFI1,FGF4,HOXB13,IL10,INHBA,MSH2,MST1R,NBN,PDGFRA,PIK3C2G,PMS1,PRDM1,RAC2,RAF1,SOCS1	0.255055327	1.177632535
path_1918	AKT1,ARID2,B2M,BRIP1,CDH1,CDK8,CHEK2,CTLA4,E2F3,HIST1H3A,HIST1H3E,MAP3K1,MAX,MITF,NEGR1,NTRK1,PMS1,RAD51B,RPS6KA4,SPOP	0.109341364	1.258915996
path_1919	AXIN2,CDK12,CDK6,CDKN1A,FANCA,FGF19,FGFR4,FLT3,IL7R,MEF2B,MEN1,NEGR1,NF2,NOTCH4,PDGFRB,PNRC1,PPP6C,PTPN11,RB1,SMAD2	0.19799197	1.253064969
path_1920	ABL1,AXIN1,BRD4,CCND3,CDK4,EIF4A2,ERCC5,FANCA,FH,IKBKE,MAP2K2,MDM4,NCOR1,PLCG2,POLE,PPM1D,RARA,RNF43,RPTOR,STK11	0.198765183	1.255646885
path_1921	APC,ASXL2,BCL2L1,BRAF,CDK6,CXCR4,FBXW7,FOXL2,FOXP1,HIST1H3B,HIST1H3G,LATS1,MAP2K4,MEN1,MSH3,PALB2,PARK2,PIK3C2G,RHOA,SDHA	0.432658913	1.117008979
path_1922	ALK,ARAF,AXIN2,BARD1,BBC3,BCL2L11,CDH1,CDKN1B,CEBPA,CXCR4,EIF4A2,ERCC4,HOXB13,IRS1,JAK3,MAP3K13,PNRC1,STAG2,TP53BP1,WT1	0.829405131	1.030997463
path_1923	CDKN2C,CREBBP,EP300,FAT1,GRIN2A,HIST1H3I,ICOSLG,INPP4A,KNSTRN,MDM2,MLL3,MSH6,NOTCH3,NTRK1,PREX2,RAD51B,RPTOR,SHQ1,STK40,TGFBR1	0.632950442	1.078226708
path_1924	ARID5B,BCL2,BRIP1,CARD11,CBL,CDKN1A,CDKN1B,DNMT3B,HIST1H3D,IKZF1,KLF4,LATS1,MAP2K1,MAP2K4,MAP3K1,NCOA3,NKX3-1,POLD1,PRDM1,SMAD4	0.231005932	1.241389028
path_1925	ATRX,BCL2,CCND1,CIC,CYSLTR2,EPHA5,ETV1,JAK3,MET,MPL,MTOR,MYC,PDGFRA,PIK3CB,RNF43,ROS1,RUNX1,SOX2,STK40,TP53	0.108280561	1.253680146
path_1926	AKT3,ASXL1,CDC73,DNAJB1,DROSHA,FAM46C,HIST1H3J,INSR,KMT2A,MDC1,MST1,MUTYH,NOTCH2,PAK1,PDGFRB,PIM1,PRDM1,RB1,RBM10,SDHAF2	0.979745669	1.00443755
path_1927	AKT3,ANKRD11,ASXL1,EIF4E,ERG,FH,FLT1,FOXA1,HIST1H1C,HIST1H3D,ICOSLG,IFNGR1,MYCL1,PPM1D,PTCH1,PTPRS,RICTOR,RTEL1,SMAD4,STAG2	0.423268476	1.119699053
path_1928	CD276,CHEK1,ERBB3,FAT1,FBXW7,GNA11,HNF1A,INSR,INSRR,KNSTRN,MAP3K13,MLL2,MUTYH,PARK2,PBRM1,PIK3CD,RAD51C,RYBP,SETD2,TP63	0.193555302	1.236394561
path_1929	ABL1,AXIN1,CDK12,CTNNB1,CUL3,DDR2,DICER1,DNMT1,ELF3,FGFR4,GATA1,GPS2,INSRR,IRF4,MAP2K4,MSH6,NKX3-1,RAC2,RAD50,RAD51B	0.413215577	1.182880243

path_1930	AKT2,CARD11,CDK12,GLI1,ICOSLG,IRS1,NCOR1,NEGR1,NKX3-1,NTRK3,PAK1,PAX5,PPM1D,PTCH1,RASA1,RYBP,SMAD4,SOX17,SRC,TET1	0.079652692	1.342255331
path_1931	AMER1,ARID5B,AXIN2,CENPA,FUBP1,HIST1H3C,IL10,KEAP1,MAP2K1,MDM2,MYCL1,NOTCH1,PAK1,PBRM1,PHOX2B,PMS1,ROS1,SH2D1A,STAT5B,TCF7L2	0.3619778	1.152680485
path_1932	ACVR1,AXL,BRD4,DAXX,ERCC5,FAT1,KEAP1,KRAS,MSH2,NFKBIA,NTRK2,PAK7,PIK3CD,PREX2,PTEN,PTPRD,RAD21,SMO,STK40,TEK	0.930403694	0.986465859
path_1933	AKT2,BCL2L11,CXCR4,DIS3,ERG,ERRFI1,ETV6,FGF3,FH,IGF1R,KIT,MDM4,NBN,NCOA3,NFKBIA,PAX5,PHOX2B,PIK3CB,PTPRD,SMAD4	0.395516432	1.127683324
path_1934	AMER1,CDC73,CEBPA,CTLA4,GNAQ,HIST1H3D,HOXB13,KEAP1,KMT2D,MEN1,MET,NCOR1,NTRK3,PALB2,PIK3CA,RB1,SOX9,STK11,TCF3,TP53BP1	0.409894881	1.123582389
path_1935	ACVR1,AMER1,BTK,CTCF,EGFR,ERBB2,FAM175A,FYN,GPS2,HIST1H1C,ICOSLG,MYCL1,NOTCH4,PPM1D,PTPN11,SDHA,SYK,TBX3,TERT,TSC1	0.535304272	1.108342531
path_1936	AR,ASXL2,DIS3,EP300,EPHA5,ETV1,FAM46C,FLT1,GRIN2A,INHBA,NOTCH2,PDGFRB,POLD1,POLE,RAF1,REL,SMAD4,SOS1,SOX2,TAP2	0.343110308	1.162919545
path_1937	ALK,AXL,CD79B,CDK4,CHEK2,FGF3,FGF4,GNA11,HIST1H3I,KMT2A,MAP2K2,MAP3K13,MCL1,NFE2L2,PIK3R1,RAF1,RET,RFWD2,SMO,SOCS1	0.75254943	1.04559838
path_1938	BRAF,CCND3,DNMT1,EPHA3,FGF19,FH,FOXL2,HIST1H3G,HIST3H3,INHA,INSR,NEGR1,NFKBIA,PARP1,PBRM1,RAD50,SMAD4,TGFBR2,TNFAIP3,TP63	0.608487492	1.090880653
path_1939	ATRX,CEBPA,EIF1AX,ERBB3,ERCC5,FANCA,HGF,KDM5C,MEF2B,MSH6,NBN,NUF2,NUP93,PALB2,PIK3CG,PPP6C,PTEN,SLX4,SOX9,TET2	0.200614521	1.231909694
path_1940	AKT1,BTK,CXCR4,DOT1L,E2F3,EIF1AX,FLT1,GNAS,HLA-A,IGF1R,KIT,KNSTRN,NKX3-1,NTRK2,PDGFRB,PIK3CA,PIK3CG,PRDM1,RHEB,ROS1	0.156936616	1.257281162
path_1941	AKT1,CASP8,CBL,CD79B,CDC42,CDKN2A,CHEK2,DNMT3A,FGFR2,IL10,NTRK2,PDCD1,RASA1,RECQL4,SMARCD1,SOX9,SPEN,SRC,STAT5B,TET1	0.153749066	1.222093675
path_1942	AKT2,ASXL2,B2M,BAP1,BARD1,CD276,CENPA,DICER1,ETV1,IRS1,NFKBIA,NOTCH4,NTRK3,POLE,ROS1,RYBP,SMARCB1,STK40,TP53BP1,U2AF1	0.062004934	1.302375318
path_1943	CARM1,CEBPA,CXCR4,DAXX,DNMT3A,ETV1,HIST3H3,HLA-A,IRS2,MITF,MTOR,MYOD1,PDGFRA,PMAIP1,RAD50,RAF1,RB1,RFWD2,RYBP,TET2	0.561242868	1.085582852

path_1944	BTK,CASP8,CCND1,CCNE1,CDC42,CDK6,CDKN1B,ERBB2,KDM5C,KLF4,MAP2K2,NUF2,PAX5,POLD1,PTEN,RTEL1,SDHB,SHQ1,TP53BP1,TRAF2	0.96706653	0.994097728
path_1945	AMER1,AR,CTCF,CTLA4,DNMT3B,EPHA3,FANCA,GNAQ,GSK3B,IL7R,IRS1,KIT,PDCD1,PIK3CG,PRDM1,RASA1,RB1,RNF43,SLX4,SRSF2	0.192415657	1.221351665
path_1946	AXIN2,CDC73,CTNNB1,DIS3,DROSHA,FGFR1,FLT3,FOXA1,GRIN2A,HIST1H3C,HIST1H3E,IRF4,MAP3K13,MED12,PIK3CA,PIK3CD,POLD1,PPP6C,RAD54L,SUFU	0.983981587	0.997155506
path_1947	AKT1,AR,B2M,CHEK1,CUL3,CXCR4,FGFR1,FLT3,FOXL2,NEGR1,NOTCH2,PAK7,PBRM1,POLD1,RICTOR,SHQ1,SMAD4,SOX2,TAP1,TMPRSS2	0.746192435	0.944722633
path_1948	ARID5B,ASXL2,B2M,BRCA1,CBL,CREBBP,CYSLTR2,FGFR2,FGFR3,FLT4,GNAQ,HGF,HIST1H3D,MAP2K4,MET,MSH2,PIK3CG,RAC2,REL,SOS1	0.223536378	1.225414624
path_1949	AKT3,BARD1,BCL2,CCNE1,CHEK2,DNAJB1,FLT4,KIT,MEN1,MET,MSH2,PAK7,PDGFRA,PLK2,PTPRD,RBM10,RYBP,SMAD3,SPEN,SRC	0.509712202	1.102252729
path_1950	ACVR1,ANKRD11,BRAF,CENPA,DNMT3A,DOT1L,FAT1,GNA11,GRIN2A,IDH1,INHA,MAP3K13,NF1,PIK3R3,RAF1,RARA,ROS1,SDHB,SETD2,SMARCB1	0.399028216	1.141181944
path_1951	AKT2,AMER1,BCL2L1,BTK,ELF3,ETV1,FGF19,GNAS,MLL2,MTOR,NCOA3,NCOR1,NRAS,PMS1,PREX2,PTPN11,SMARCB1,SOCS1,STK40,TET2	0.357227689	1.138853927
path_1952	ARID1B,BLM,BRIP1,CTLA4,DNMT3A,EGFR,ERCC5,EZH2,FLT3,FOXO1,HIST1H3J,MSH3,MYCL1,NFE2L2,PGR,PMS2,PTEN,RAD54L,RBM10,RICTOR	0.495758246	1.109802528
path_1953	BRAF,CHEK1,EIF1AX,EPHA5,EPHB1,ERCC5,FLT3,FOXO1,INPP4A,IRF4,KDM5A,KLF4,NOTCH1,PMAIP1,RAF1,RASA1,RECQL4,SRSF2,TNFAIP3,TP63	0.617529348	1.073207685
path_1954	AKT2,ALK,CREBBP,E2F3,ERCC2,H3F3C,HIST1H3C,JAK2,MAP2K4,MAP3K1,MAP3K13,MED12,NCOR1,NOTCH4,NPM1,NTRK3,PTPRT,RAD51,RB1,TBX3	0.195797256	1.214291845
path_1955	APC,ARID1A,BCL6,CCND3,CDC42,CDH1,DNMT3B,ETV6,FLT3,GNAS,HIST1H1C,HIST1H3C,HIST1H3E,INPP4A,PAK1,PBRM1,PIK3CA,PIK3CD,RAD51,RAD54L	0.742862809	1.058715586
path_1956	BCL2L1,CENPA,CHEK1,CREBBP,EP300,EPHA7,EPHB1,FLT3,GNA11,H3F3C,HIST1H3D,HIST1H3H,INPP4A,JAK1,JAK2,KIT,MAX,NFE2L2,PDGFRA,RAF1	0.086260765	1.346450264
path_1957	AXIN2,CD79B,CDC42,CDK6,EPHB1,ERBB2,ERG,GLI1,INHBA,KDM5C,LATS1,MAX,MRE11A,NBN,PIK3CD,RUNX1,SDHAF2,TNFAIP3,TP53BP1,TRAF2	0.663421695	1.063838077

path_1958	AR,CCND3,CHEK1,CTNNB1,DNMT1,E2F3,ELF3,FANCA,GRIN2A,HNF1A,IKBKE,KDR,MYC,NF1,NKX3-1,NRAS,PDGFRA,PHOX2B,PLK2,TERT	0.523414742	1.108533915
path_1959	BCL6,CCND1,CTNNB1,ERBB2,ETV6,FGFR4,GLI1,HIST1H3C,HOXB13,IKZF1,MDM4,PIK3CB,PIM1,RAD51C,RAD54L,RASA1,SH2D1A,SRSF2,TCF3,U2AF1	0.553290758	1.088527934
path_1960	ALOX12B,ANKRD11,BAP1,CCND3,CSF1R,ERBB4,FAM46C,H3F3C,ICOSLG,IFNGR1,KIT,KLF4,MYC,NCOR1,PTEN,PTPN11,PTPRD,RASA1,RB1,SLX4	0.470349199	1.119113792
path_1961	AURKA,CEBPA,CXCR4,DIS3,EIF4E,ERCC2,HIST1H3C,HOXB13,MITF,MLL2,MLL3,NCOA3,RAD51,RAD51D,RYBP,SPOP,TAP1,TBX3,U2AF1,WT1	0.085433027	1.383425352
path_1962	CARD11,CASP8,CCND3,CTLA4,DICER1,ERRFI1,FYN,GATA2,INHA,KDM5A,KEAP1,MALT1,MTOR,NTRK1,PALB2,PTPN11,RAD51,RYBP,SMAD4,SMO	0.227868689	1.209922765
path_1963	APC,ASXL1,ATR,AXL,CDK4,DNAJB1,HIST1H3B,HIST1H3D,MLL2,NEGR1,NF2,NFE2L2,NOTCH2,PIK3CA,PTPRS,RARA,RHEB,SMAD2,SOS1,SOX17	0.158857185	1.266703815
path_1964	BCL2L11,CDC42,CDK6,CUL3,EIF4A2,EPHA7,ERBB3,ETV6,GPS2,ICOSLG,KRAS,MAP2K1,MYOD1,PIM1,PMS2,POLD1,SLX4,SMAD3,SPOP,SYK	0.311267719	1.176941667
path_1965	AR,BARD1,BCL10,BCL2,BCL6,CDC73,CEBPA,E2F3,EIF1AX,EPHA5,ERBB4,FGFR3,FLT3,HNF1A,IKZF1,IL10,INSRR,PAX5,RECQL4,SYK	0.137877345	1.323180914
path_1966	ABL1,AXIN1,CDC73,DAXX,EIF1AX,GATA1,IKZF1,MAP3K13,MED12,MITF,MUTYH,MYCL1,NCOA3,NCOR1,NRAS,NTRK2,PIK3CA,RPS6KA4,SH2B3,STAG2	0.131290927	0.810330478
path_1967	ACVR1,CD276,CIC,DIS3,EP300,FGFR1,FGFR2,HGF,IGF1R,IL10,INSR,INSRR,MCL1,MSH2,NFKBIA,NOTCH2,PBRM1,PDCD1,RAC2,SOX9	0.341361675	1.144404463
path_1968	ARID1B,ATM,DNAJB1,DNMT3B,E2F3,ERBB4,ESR1,FGFR4,HIST1H3C,ICOSLG,IKBKE,MAP2K4,PARK2,PRDM1,PTPRT,RAD51C,RTEL1,SHQ1,SMARCB1,SOX9	0.511287956	1.110424235
path_1969	AMER1,BRIP1,CTNNB1,CYSLTR2,DNAJB1,FLT1,FLT4,FOXL2,HIST1H1C,HIST1H3D,HIST1H3J,MRE11A,MUTYH,NTRK1,PLK2,POLD1,PREX2,RHEB,SOX9,TBX3	0.139054768	1.290095081
path_1970	AXIN1,CDK8,CDKN1B,EZH2,FH,GLI1,HIST1H1C,HIST1H3B,KEAP1,MCL1,MEN1,MET,MYC,NBN,NTRK2,PGR,RAD50,SMAD2,SMARCB1,SPEN	0.258963583	1.192774335
path_1971	ALK,BBC3,BRCA2,BRIP1,CDKN1A,CSF1R,DOT1L,EIF4A2,FGFR2,H3F3C,JAK3,KIT,KLF4,NKX2-1,NRAS,PDCD1,PLCG2,PPM1D,RFWD2,SOS1	0.067718702	1.344426652
path_1972	ABL1,AKT3,AXIN1,CDC42,DROSHA,ELF3,ERBB4,FH,FOXL2,FOXO1,HIST1H1C,KDM5A,LATS2,MDM4,MYOD1,NTRK1,PAK7,PRDM1,SDHB,SMARCD1	0.753438702	1.059120455

path_1973	ALK,ANKRD11,CALR,CENPA,ETV1,FGF4,FGFR3,FOXA1,GATA2,GNA11,KMT2D,MLL,MYC,NF1,PBRM1,PDGFRB,RAD51B,SDHB,SOS1,SUFU	0.229075867	1.235056924
path_1974	ARID1B,ATRX,BMPR1A,BRAF,EGFR,EPHA5,EPHA7,FANCA,GLI1,HIST1H3E,HIST3H3,HLA-A,ICOSLG,IRF4,JAK1,JUN,KMT2A,MST1,NPM1,PLCG2	0.370019929	1.14826817
path_1975	CD79B,CEBPA,DIS3,EGFR,FLT4,HIST1H1C,HLA-A,MAP2K1,MDC1,MGA,MLL,MLL3,MSH2,NFE2L2,NOTCH4,PTPRD,PTPRT,RHEB,SOCS1,SPOP	0.285011236	1.164840583
path_1976	ALOX12B,CASP8,CDKN2A,CSF3R,DICER1,FANCA,GNAS,IDH1,KDM5A,MALT1,MEN1,NOTCH4,NTRK2,PALB2,PHOX2B,PIK3CD,RARA,SPOP,TERT,TGFBR1	0.614088146	0.911112762
path_1977	AMER1,CDK12,DNAJB1,DNMT3A,EIF4E,FOXL2,GRIN2A,HIST1H3C,HIST1H3I,KIT,LATS2,MDM2,MSH2,NSD1,PLCG2,PMAIP1,PNRC1,POLD1,RHOA,STAT5B	0.865067425	0.976271373
path_1978	AKT3,AR,CARM1,CDK6,CYSLTR2,EPHA5,ERG,FANCC,FGF4,GATA2,GSK3B,HIST1H1C,MRE11A,NF2,PAK7,PMS1,PPP6C,RECQL4,SH2B3,TERT	0.064359923	1.359541328
path_1979	BCL2L1,BTK,CARD11,CDC42,CDKN2A,EED,EIF4A2,EIF4E,GNAQ,GRIN2A,HGF,ICOSLG,INHBA,LATS1,NF2,NOTCH1,PARK2,PLCG2,PMS2,RARA	0.098399678	1.300832611
path_1980	ARID1A,ASXL1,CDK12,CHEK1,EPHA5,ERG,ESR1,GATA3,H3F3C,HOXB13,KLF4,LATS2,MCL1,MITF,PARK2,PHOX2B,PIK3CD,POLE,SOCS1,SRC	0.087780699	1.341287336
path_1981	B2M,BMPR1A,CBL,CDKN1A,CIC,CSF1R,FAM46C,GNA11,IKBKE,IL10,KNSTRN,MED12,MLL,NKX2-1,NUF2,PDGFRA,PMS1,SMARCD1,STK11,TOP1	0.216857116	1.234937146
path_1982	BCL2L1,BRAF,BTK,CD274,CHEK2,CTNNB1,CXCR4,ERBB2,FGFR2,FLT4,FOXA1,INSRR,KDM6A,MGA,PDGFRA,PDGFRB,PMS2,SOX17,SRSF2,TCF3	0.451215543	1.138982176
path_1983	ARID5B,AXL,BCOR,BLM,CDK12,CDK8,DIS3,EP300,EPHA3,IKBKE,IL7R,LATS1,MSH6,PLCG2,PLK2,RAD21,RARA,RPS6KA4,STK40,TRAF2	0.262829403	1.214928629
path_1984	BRIP1,CCND2,DDR2,EIF4A2,ERG,EZH2,FAM175A,FAT1,H3F3C,INHBA,KRAS,MAP3K13,MED12,MGA,MST1,NCOA3,RAD51,RUNX1,SDHAF2,SLX4	0.122762689	1.246133733
path_1985	B2M,BLM,CD276,CYSLTR2,EP300,ERBB2,ESR1,FAM175A,FGF3,FLT4,IFNGR1,MEF2B,NF2,NOTCH4,PPP2R1A,RAD51C,ROS1,TCF3,TET2,TNFAIP3	0.181441319	1.242109503

path_1986	ACVR1,BMPR1A,CHEK2,CTCF,DNAJB1,ERCC4,FANCA,FLT3,GATA2,HIST1H3B,JUN,MALT1,PHOX2B,PMAIP1,SDHA,SOX17,SRC,STAT3,TCF3,TEK	0.916932577	0.984365716
path_1987	ASXL2,ATM,BLM,CDKN1A,CDKN2A,DNAJB1,EP300,EPHA3,FANCC,HIST1H3I,IRS2,JAK1,LATS1,MEN1,MST1R,NEGR1,NRAS,PTEN,SMO,TAP1	0.063524339	1.327270325
path_1988	ATM,CCND2,CENPA,CREBBP,FGFR2,HGF,HIST1H3I,ICOSLG,MDM2,MED12,MET,MGA,MLL2,NFE2L2,NTRK1,PIK3R1,PPP6C,SMARCB1,SOX2,STAG2	0.885584859	1.021891004
path_1989	ALK,CHEK1,DDR2,DOT1L,ERBB2,ERBB4,FANCC,GATA1,HIST1H3E,HIST3H3,INPP4A,JAK1,NPM1,RAD51B,RYBP,SOX2,SOX9,SPEN,SPOP,STAT5B	0.064673735	1.297451304
path_1990	ARID1B,ARID2,ATR,BCL6,CCNE1,CDK12,DROSHA,GLI1,HIST1H3G,IL7R,INHBA,KEAP1,MAP3K13,MSH2,PHOX2B,POLD1,PTPRS,RAD54L,RNF43,RPS6KA4	0.846696869	0.970307246
path_1991	CDK8,CDKN2A,CSF1R,EPHA3,ERCC4,FH,FOXL2,HIST1H3J,MAP2K4,PARK2,PDCD1,PMAIP1,RAC2,RB1,RHOA,RTEL1,SOS1,SRSF2,STK40,WT1	0.359961692	1.176349858
path_1992	ABL1,AXIN2,BCOR,BRCA1,EIF4A2,INSRR,IRS1,JAK3,KNSTRN,MITF,MST1R,MYCN,NBN,PARP1,PDCD1,PIK3CG,PRDM1,RYBP,TSC2,XPO1	0.201092699	1.269437995
path_1993	ACVR1,AMER1,AURKA,BCL2,BRCA2,CBL,CDK8,ERBB3,FAM175A,GNA11,KDM5C,MST1,NUP93,PARP1,PTPRD,RAD21,RPTOR,SMAD4,SMARCD1,SRC	0.498935665	1.119600535
path_1994	ARID2,ASXL1,AXL,CTLA4,DNMT3A,HIST1H3D,HIST1H3H,IDH1,KNSTRN,MAP3K13,MITF,MYOD1,PIK3R1,PNRC1,RAD51,RAD54L,RHOA,SOS1,STAT5B,TET2	0.508304086	0.910857642
path_1995	ALOX12B,AMER1,CDKN1A,CREBBP,ERG,ERRFI1,FGF19,FGFR1,FGFR4,FOXP1,H3F3C,MCL1,MPL,MST1,PAK7,PTEN,RAD51,RAF1,RTEL1,SOS1	0.092606663	1.267446206
path_1996	ARID1B,ARID5B,CCND1,EED,EPHB1,ERCC2,ERCC4,FGFR1,GATA1,GNA11,INSRR,MSH2,MST1,MYCN,NCOR1,NOTCH4,POLE,PPM1D,RAD51C,ROS1	0.328676639	1.18033355
path_1997	AKT3,ANKRD11,CCND2,CTNNB1,FLT1,GSK3B,HGF,HIST1H3J,IL7R,INSRR,IRS1,MAP2K2,PIK3CB,RAF1,RHOA,RICTOR,RPS6KB2,RTEL1,SOX2,SOX9	0.842454848	1.02841185
path_1998	BCL10,BCOR,BRD4,CD79B,CDK12,CDKN1A,FUBP1,GLI1,GNA11,IKBKE,INSRR,MLL2,MST1,MUTYH,PDGFRB,PIK3CB,PIK3CG,PMS2,RAD51C,RFWD2	0.788647034	0.953972434
path_1999	AKT3,ALOX12B,BBC3,EIF4A2,FOXA1,GATA1,GATA2,H3F3C,KLF4,MAP3K13,MDM4,MITF,PDCD1,PGR,PPP2R1A,RAD51C,RAF1,SDHB,SMARCA4,SRSF2	0.737979359	1.04842535

path_2000	ATR,BCL6,BRCA2,CIC,EGFR,EPHA5,FANCC,FLT3,HIST1H3B,IL7R,IRS2,MAP2K2,MEF2B,NBN,NKX2-1,NSD1,PAK7,PARP1,PIK3R3,SPEN	0.265080877	1.180572081
