# Supplementary material for: Prognosis of Non-small-cell Lung Cancer Patients With Lipid Metabolism Pathway Alternations to Immunotherapy
Source: Front Genet. 2021 Jul 14;12:646362. doi: 10.3389/fgene.2021.646362 (PMC8317604; doi:10.3389/fgene.2021.646362)
Supplement: Supplementary file 8 [file Data_Sheet_4.docx]

**DNA extraction**

Extraction of tumor DNA from Formalin-fixed paraffin-embedded (FFPE) sample was with QIAamp DNA FFPE tissue kit (Qiagen), and extraction of PBL DNA was with the RelaxGene Blood DNA system (Tiangen Biotech Co., Ltd., Beijing, China) according to the manufacturer’s protocol. All of the DNA samples were quantified both using the Qubit 2.0 fluorometer and the Qubit dsDNA HS Assay kit (Thermo Fisher Scientific, Inc., Waltham, MA, USA) according to the manufacturer’s protocol.

**Library construction and sequencing**

100ng of DNA from each sample was sheared by the dsDNA Fragmentase (New England BioLabs, Inc., Ipswich, MA, USA), and then performed size selection (150-250 bp) using Ampure XP beads (Beckman Coulter, Inc., Brea, CA, USA). Library construction was performed using the KAPA Library Preparation kit (Kapa Biosystems, Inc., Wilmington, MA, USA) according to the manufacturer’s protocol. The concentration of the library were assessed using the e Qubit dsDNA HS Assay kit, and fragment length was determined on a 4200 Bioanalyzer (Agilent Technologies, Inc., Santa Clara, CA, USA). Target enrichment was carried out using the Agilent

SureSelect XT HS kit (Agilent Technologies) according to the manufacturer's Protocol. DNA sequencing was then performed on the Illumina Novaseq 6000 system.

**Data analysis and variant calling**

Raw sequences were pre-processed by fastp version 0.18.0 (https://github.com/OpenGene/fastp) [1], and clean reads were aligned to the hg19 genome (GRch37) using Burrows-Wheeler Aligner maximal exact matches algorithm [2]. The Gencore version 0.12.0(<https://github.com/OpenGene/gencore>) [3] was used for removing duplicate reads. Pileup files with mapping quality ≥60 were generated using Samtools version 0.1.19(http://www.htslib.org/) [4]. Somatic variants were called using VarScan2 version 2.3.8 (http://varscan.sourceforge.net/) [5] [the minimum read depth 20; the variant allele frequency (VAF) threshold ≥0.01; somatic‑P‑value ≤0.01; strand‑filter ≥1; others, default parameters]. CNV kit with version 0.9.3(<https://github.com/etal/cnvkit>) [6] were used for copy number variation detection, and GeneFuse version v0.6.1 (<https://github.com/OpenGene/GeneFuse>) [7] for structural variation detection.

References

1. Shifu Chen, Yanqing Zhou, Yaru Chen, Jia Gu; fastp: an ultra-fast all-in-one FASTQ preprocessor, Bioinformatics, Volume 34, Issue 17, 1 September 2018, Pages i884–i890, https://doi.org/10.1093/bioinformatics/bty560

2. Li H and Durbin R: Fast and accurate long‑read alignment with Burrows‑Wheeler transform. Bioinformatics 26: 589‑595, 2010.

3. gencore: an efficient tool to generate consensus reads for error suppressing and duplicate removing of NGS data. Shifu Chen, Yanqing Zhou, Yaru Chen, Tanxiao Huang, Wenting Liao, Yun Xu, Zhihua Liu, Jia Gu bioRxiv 501502; doi: https://doi.org/10.1101/501502.

4. Li H, Handsaker B, Wysoker A, Fennell T, Ruan J, Homer N, Marth G, Abecasis G and Durbin R; 1000 Genome Project Data Processing Subgroup: The sequence alignment/map (SAM) format and SAMtools. Bioinformatics 25 2078‑2079, 2009.

5. Koboldt DC, Zhang Q, Larson DE, Shen D, McLellan MD, Lin L, Miller C, Mardis ER, Ding L and Wilson RK: VarScan 2: Somatic mutation and copy number alteration discovery in cancer by exome sequencing. Genome Res 22: 568‑576, 2012.

6. Talevich, E., Shain, A.H., Botton, T., & Bastian, B.C. (2014). CNVkit: Genome-wide copy number detection and visualization from targeted sequencing. PLOS Computational Biology 12(4):e1004873.

7. Shifu Chen, Ming Liu, Tanxiao Huang, Wenting Liao, Mingyan Xu and Jia Gu. GeneFuse: detection and visualization of target gene fusions from DNA sequencing data. International Journal of Biological Sciences, 2018; 14(8): 843-848. doi: 10.7150/ijbs.24626.
